# Repeated evolution of camouflage in speciose desert rodents

**DOI:** 10.1038/s41598-017-03444-y

**Published:** 2017-06-14

**Authors:** Zbyszek Boratyński, José C. Brito, João C. Campos, José L. Cunha, Laurent Granjon, Tapio Mappes, Arame Ndiaye, Barbara Rzebik-Kowalska, Nina Serén

**Affiliations:** 1 CIBIO-InBIO Associate Laboratory, Research Center in Biodiversity and Genetic Resources, University of Porto, Vairão, 4485-661 Portugal; 2 Department of Biology, Faculty of Science, University of Porto, Rua Campo Alegre, 4169-007 Porto Portugal; 3 IRD, UMR CBGP, Campus International de Baillarguet, CS 30016, 34988 Montferrier-sur-Lez cedex, France; 4 Department of Biological and Environmental Science, University of Jyväskylä, Jyväskylä, P.O. Box 35, 40014 Finland; 5 Department of Animal Biology, Faculty of Sciences and Technologies, University Cheikh Anta Diop, BP 5005 Dakar, Senegal; 6 Institute of Systematics and Evolution of Animals, Polish Academy of Sciences, Sławkowska 17, 31-016 Kraków, Poland

## Abstract

There are two main factors explaining variation among species and the evolution of characters along phylogeny: adaptive change, including phenotypic and genetic responses to selective pressures, and phylogenetic inertia, or the resemblance between species due to shared phylogenetic history. Phenotype-habitat colour match, a classic Darwinian example of the evolution of camouflage (crypsis), offers the opportunity to test the importance of historical versus ecological mechanisms in shaping phenotypes among phylogenetically closely related taxa. To assess it, we investigated fur (phenotypic data) and habitat (remote sensing data) colourations, along with phylogenetic information, in the species-rich *Gerbillus* genus. Overall, we found a strong phenotype-habitat match, once the phylogenetic signal is taken into account. We found that camouflage has been acquired and lost repeatedly in the course of the evolutionary history of *Gerbillus*. Our results suggest that fur colouration and its covariation with habitat is a relatively labile character in mammals, potentially responding quickly to selection. Relatively unconstrained and substantial genetic basis, as well as structural and functional independence from other fitness traits of mammalian colouration might be responsible for that observation.

## Introduction

Two main factors are invoked to shape the evolution of characters in the course of a phylogenetic history: adaptive change and phylogenetic inertia. On the one hand, adaptive variation in phenotype and its relationship with fitness, causes covariation between phenotypic and environmental features in response to selection^1, 2^. On the other hand, species’ phenotypes are constrained by their evolutionary history due to shared ancestry. Phylogenetic inertia states that more closely related species will be phenotypically more similar to each others, as opposed to species that are more phylogenetically distant^3–5^. In addition to phylogenetic resemblance caused solely by stochastic effects in generating phylogenetic signal (e.g. genetic drift), phylogenetic inertia states that lack of genetic variation, developmental and structural constraints, and shared adaptive landscape can impede diversification^3, 6, 7^. However, if enough genetic variation is present and if traits are relatively evolutionarily independent, the functional characters can diversify in response to selection varying across habitats, causing phenotypic divergence along the phylogenetic tree^8, 9^. When habitat variation and the associated selective pressures are strong enough, adaptive processes can reduce or even overcome phylogenetic inertia^8, 10^.

The most classical examples of adaptive evolution are related to the emergence of colour polymorphism, particularly with the evolution of camouflage colouration in animals^11–14^. Simply, camouflage (or crypsis) is a functional trait characterized by the match between the organisms’ colour/pattern and the associated background, in order to avoid being detected by predators^13^. A handful of studies, inspired by Darwin’s seminal example of camouflage in a subfamily of grouse birds^15^, directly tested the selection on camouflage phenotype, mainly in response to diurnal and nocturnal avian^16–20^ or mammalian predators^21^. Others suggested that evolution related to emergence of camouflage can result in pronounced phenotypic variation associated to restricted habitat use and/or habitat specialization, and showed substantial genetic variation in colourations allowing response to selective pressures^2, 22–24^. Evolution of animal-habitat match in colouration has been described and experimentally tested in open habitat types, where prey adapt to the substrate to increase survival^12, 18, 25^. The environment, however, is usually spatially structured resulting in substantial variation in habitat distribution and associated differential selective advantages of particular phenotypes^11, 12, 26^. This raises the question of how (micro) habitat variation shaped evolutionary history of camouflage in diverse, closely related and sympatric species.

Phylogenetic inertia and stabilizing selection can both constrain a trait’s evolution and generate a similar pattern of phenotype-habitat linkage across a phylogeny^27^. In contrast, in structured landscapes spatially variable selection should generate a pattern of relatively phylogenetically independent phenotypic diversity among related species^3, 10^. In such cases, phenotypic variation should primarily be explained by variation in the habitat occupied by the species or population, and the phylogenetic signal describing relatedness among the species should be less important. To partition the variation of putatively adaptive traits into evolutionary history and functional capacity^28, 29^, we used phenotypic data of animal colouration^30^, remotely sensed habitat colouration^25^, and phylogenetic information^8^ using the speciose *Gerbillus* rodent genus as model^31, 32^. *Gerbillus* species are known for their adaptation to open, dry to semi-dry habitats, as well as for their spatial and temporal partitioning of microhabitats, an outcome of a balance between the resource competition and predation risk of foraging in open, but often productive, habitats^33–35^. The predation level and selection for camouflage can vary between, and even within, species according to the type of explored microhabitat^36^, even in strictly nocturnal rodents^17^. But as *Gerbillus* is of recent origin and includes many closely related species^32^ living often in sympatry in the very same ecoregion^37, 38^, they can also experience similar selective pressures. We used animal colouration as it is usually a strong predictor of fitness in species from open habitat (including nocturnal ones)^2, 17, 18, 39, 40^. We predict that the significant differences between species in habitat and phenotype, and phenotype-habitat match would signify the importance of habitat structure and habitat specific selection in shaping phenotype. Alternatively, consistent variation across the phylogeny and phenotypic similarity between closely related species, would suggest the importance of phylogenetic inertia or/and similar selection pressures within clades. In general, the colouration in animals is well studied, but only a handful of studies has aimed to test the relative importance of phylogenetic and ecological signals among closely related taxa^41, 42^. Here we took opportunity to test it in a speciose rodent genus that inhabits open although micro-geographically heterogenous habitats, thus exposed to variable and probably occasionally strong selection promoting the evolution of camouflage.

## Results

### Repeatability, Correlations and Variation

**Figure 1:**
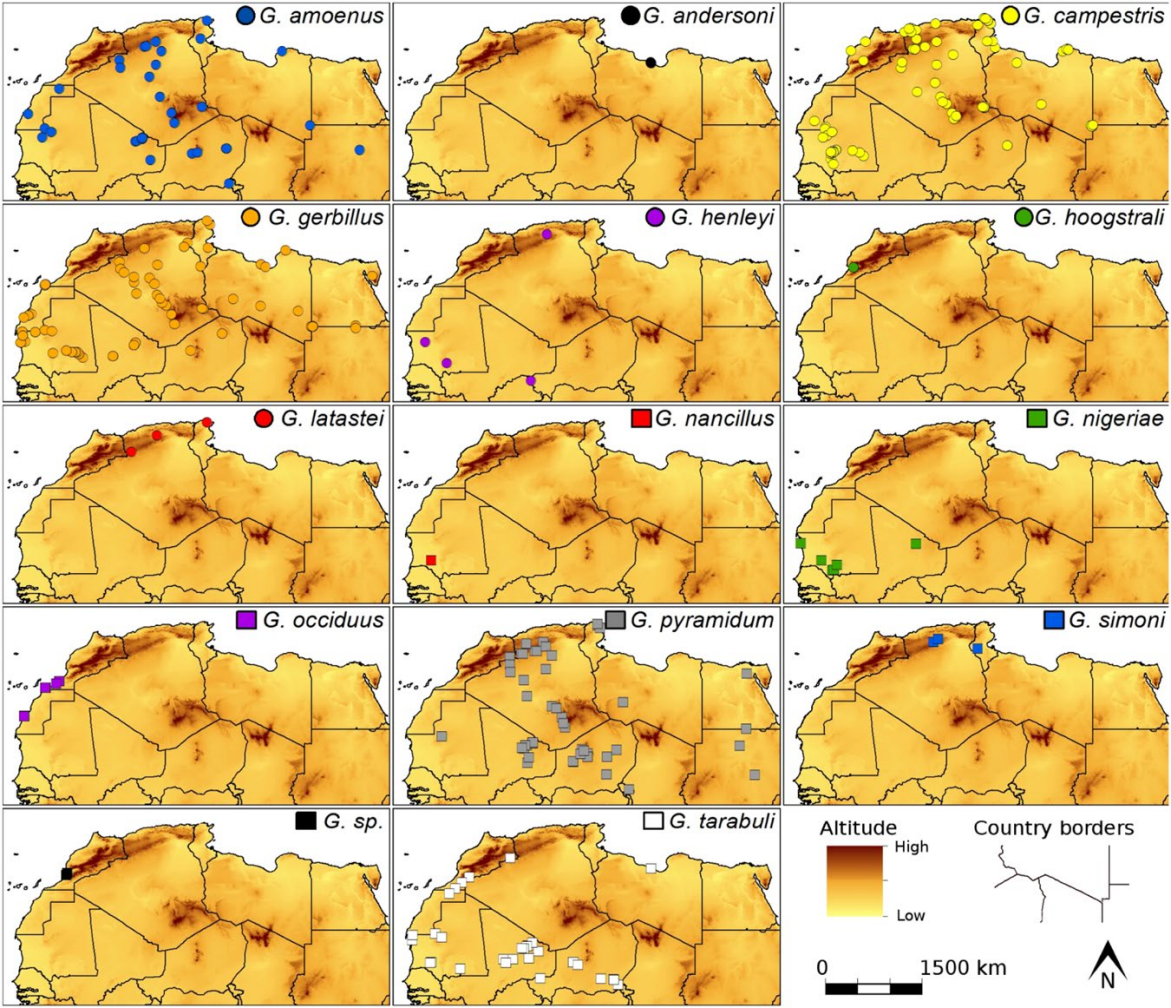
Maps of distribution of specimens included in this study (generated in: ArcGIS ver. 10.1, www.arcgis.com; see detailed information in Supplementary Material: Data Description).

**Figure 2:**
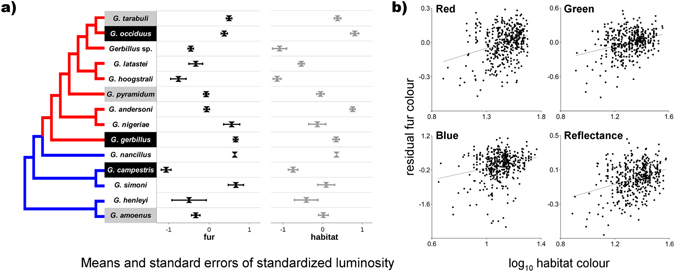
(**a)** Phylogenetic hypothesis (tree) derived from molecular data for the *Gerbillus* species used in this study. The signs of scores for phylogenetic eigenvector (EV1) derived from phylogenetic vector regression (PVR) are depicted on the phylogenetic tree with contrasting pair of colours, of red (negative) and blue (positive scores). Names of species for which signs, or lack of signs, of camouflage adaptation were detected (significant/insignificant relationship between fur and habitat colourations: Table 2) are indicated with black or grey backgrounds, respectively (white background indicate lack of data for comprehensive test). Right panels present means (and standard errors) of log-transformed and standardized fur (black marks, accounted for variation between field/collections specimens) and habitat (grey marks) total reflectance. (**b)** The relationship between the residual animal fur and habitat colours for red (R^2^ = 0.08, f(x) = 0.30x − 0.44), green (R^2^ = 0.10, f(x) = 0.58x − 0.76) and blue (R^2^ = 0.06, f(x) = 1.21x − 1.33) colour spectrum, and total reflectance (R^2^ = 0.10, f(x) = 0.46x − 0.63). Residuals were calculated from linear models including animal fur colours as dependent variables and independent variables of: geographic location affiliation (latitude and longitude), museum collection affiliation (seven levels), field affiliation (two levels) and selected in PVR analysis phylogenetic eigenvectors.

Analyses on samples (n = 460) belonging to fourteen North African *Gerbillus* species (Fig. 1) showed that average levels of total reflectance and RGB colours varied significantly among species, both for fur (colour values accounting for field/collection specimen origin, species effect in ANOVA: df = 13, F > 7.02, p < 0.0001) and for habitat (species effect in ANOVA: df = 13, F > 12.44, p < 0.0001) colouration (Fig. 2a). The estimated variations were highly and significantly repeatable (intra-class correlation coefficient) for reflectance and RGB colours of animal fur (df = 39, τ ≥ 0.99, p < 0.0001) and habitat (df = 39, τ ≥ 0.90, p < 0.0001; Supplementary information: Table S1). The estimated Pearson’s product-moment correlations between residual (accounting for variation in: geographic location, field/collection origin, and phylogenetic eigenvectors) reflectance (r = 0.33) and RGB (red: r = 0.31, green: r = 0.32, blue: r = 0.26, p < 0.0001) colours of animal fur and habitat were also significant (Fig. 2b), but simpler partial correlations (accounting for collection origin) varied between species (Table S2).

### Phylogenetic Vector Regression

**Figure 3:**
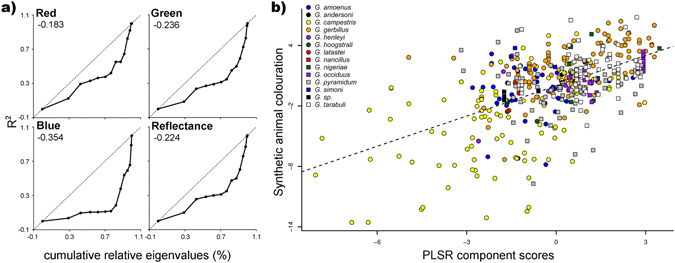
(**a)** Closely related species are less similar in colouration than expected, as indicated by negative mean deviation from phylogenetic signal-representation (PSR) curves. PSR curves for animal fur colouration (RGB and total reflectance) were derived from phylogenetic vector regression (PVR) analysis by sequentially including 12 eigenvectors. R^2^ describes the amount of the variance explained by the phylogenetic eigenvectors. Dashed lines represent patterns under Brownian motion evolution. Mean differences between R^2^ and eigenvalues, a measure of the phylogenetic signal, are indicated. (**b)** Animal colouration followed the colouration of the environment inhabited by individuals. Habitat carries a positive, and latitude a negative, load to the relationship between the partial least-squares regression (PLSR) component scores and synthetic (including variation in RGB colour spectrum and total reflectance) variable of animal fur colouration. PLSR component scores represent the position of sampling units (individual specimens) along an axis composed of the predictor variables of habitat colouration (RGB and total reflectance), geographic coordinates, museum/field affiliation and phylogenetic signal (eigenvector scores from phylogenetic vector analyses, PVR).

Phylogenetic hypothesis (Fig. 2a) was used to test for phylogenetic signal in trait evolution. Phylogenetic signal-representation (PSR) curves, representing the amount of divergence in traits along the eigenvectors against their cumulative eigenvalues, showed deviation from a linear relationship. Mean deviations from PSR curve were always negative for all traits indicating that closely related species are less similar in colouration than expected under Brownian motion (Fig. 3a). Only one phylogenetic eigenvector was selected per colour trait using Moran’s I autocorrelation method (for Moran’s I < 0.06). The first phylogenetic eigenvector was selected for all colouration traits discriminating *G*. *amoenus*, *G*. *henleyi*, *G*. *simoni*, *G*. *campestris* and *G*. *nancillus* (positive scores) from other *Gerbillus* species (negative scores; Fig. 2a). The species-specific phylogenetic eigenvector scores were assigned to the specimens of a given species and included as predictor variables in the partial least squares regression (PLSR) models.

### Partial Least Squares Regression

The PLSR model for multiple response variables (RGB colours and total reflectance) resulted in a significant component that explained 45.2% of the variance in the dataset. Pearson’s product-moment correlation between response variables and PLSR components was high and significant (r = 0.56, t = 14.7, df = 458, p < 2.2e-16; Fig. 3b). The weights of the habitat colouration indexes (RGB and total reflectance) were positive and relatively high, indicating that the synthetic response variable describing animal colouration (derived from RGB colours and total reflectance) followed the colouration of the respective habitat of individuals (Table 1). Another important predictor affecting animal colourations was latitude, indicating that specimens from lower latitudes were brighter in colouration. The phylogenetic eigenvector (EV1) and origin of the specimen (field versus museum collected) were other important predictors, explaining more than 5% variance of animal colouration. Specimens of the same species coming from different collections behave similarly (non-significant collection affiliation effect) indicating that fur characteristics were not differentially affected by taxidermic treatments and preparation, but field collected individuals were in average brighter than museum specimens (a condition therefore controlled for in all models; Table 1). All important predictors (all colouration traits, latitude, field origin and phylogenetic eigenvector) were also statistically significant (Table 1). The results derived from the dataset reduced to barcoded specimens were similar, with geographic location being relatively more important (explaining more), and phylogenetic signal relatively less important (explaining less of the variation in dependent variables; Table S3). The PLSR analyses that included additional phylogenetic eigenvectors than those statistically selected by Moran’s I test gave qualitatively similar results (Table S3). Similar results were also obtained in analyses on residual values (from linear regression) of fur colouration corrected for phylogenetic eigenvectors, prior to PLSR analyses (Table S3). Likewise alternative phylogenetic position of *G*. *nancillus* did not affect the main results (Table S4).

**Table 1:** Partial least squares regression (PLSR) analyses results for dorsal fur colour (synthetic colouration includes RGB colours and total reflectance variables) and habitat colouration, geographic coordinates, field/collection affiliations and phylogenetic eigenvectors for *Gerbillus* rodents from Sahara-Sahel region.

	synthetic colouration		log_10_ red		log_10_ green		log_10_ blue		log_10_ total reflectance	
	W	β (±SE)	W	β (±SE)	W	β (±SE)	W	β (±SE)	W	β (±SE)
log_10_ red habitat	**0**.41***	0.12 (±0.008)	**0**.40***	0.13 (±0.007)	**0**.41***	0.13 (±0.008)	**0**.42**	0.10 (±0.010)	**0**.41***	0.13 (±0.007)
log_10_ green habitat	**0**.39***	0.11 (±0.008)	**0**.39***	0.13 (±0.006)	**0**.39***	0.12 (±0.007)	**0**.40**	0.09 (±0.009)	**0**.39***	0.12 (±0.007)
log_10_ blue habitat	**0**.37***	0.11 (±0.009)	**0**.37***	0.12 (±0.007)	**0**.37***	0.12 (±0.008)	**0**.38**	0.09 (±0.010)	**0**.37***	0.12 (±0.008)
log_10_ reflectance habitat	**0**.41***	0.12 (±0.008)	**0**.40***	0.13 (±0.007)	**0**.40***	0.13 (±0.007)	**0**.42**	0.09 (±0.009)	**0**.40***	0.13 (±0.007)
latitude	−**0**.34**	−0.10 (±0.010)	−**0**.38***	−0.12 (±0.009)	−**0**.35**	−0.11 (±0.009)	−**0**.32**	−0.07 (±0.011)	−**0**.36***	−0.11 (±0.009)
longitude	−0.19*	−0.06 (±0.012)	−0.20**	−0.07 (±0.012)	−0.20**	−0.06 (±0.012)	−0.17*	−0.04 (±0.012)	−0.21**	−0.07 (±0.012)
collection affiliation	0.02	0.01 (±0.011)	0.08	0.03 (±0.011)	0.01	0.01 (±0.012)	0.01	0.01 (±0.012)	0.02	0.01 (±0.012)
field collected	**0**.35**	0.10 (±0.012)	**0**.28**	0.09 (±0.010)	**0**.36**	0.11 (±0.011)	**0**.37**	0.08 (±0.014)	**0**.33**	0.11 (±0.011)
EV1	−**0**.31**	−0.09 (±0.008)	−**0**.35**	−0.12 (±0.008)	−**0**.31**	−0.10 (±0.008)	−**0**.27*	−0.06 (±0.010)	−**0**.32***	−0.10 (±0.008)
explained variance (%)		45.2		45.5		44.9		45.2		45.1

Significance levels of regression coefficients of predictors: ***<0.001, **<0.01, *<0.05. EV1 – eigenvector 1 from phylogenetic vector regression (PVR) models. Explained variance (%) in the response variables depicted by the PLSR models. W, the weights of predictors’ contribution of variables to PLSR models, explaining more than 5% of the total variance are indicated in bold.

The PLSR models with single response variables (red, green, blue or total reflectance) also resulted in significant components that explained a substantial proportion (>44%) of the variance in response variables (Table 1). Pearson’s product-moment correlations between response variables and PLSR components were high and significant in all analyses (red: r = 0.65, t = 18.3; green: r = 0.61, t = 16.4; blue: r = 0.44, t = 10.5; and total reflectance: r = 0.63, t = 17.3, df = 458, p < 2.2e-16). The weights for the PLSR components, similarly to the multiple response variable model results, pointed on the importance of habitat colour variables and latitudinal geographic location, as well as phylogenetic signal (Table 1).

The PLSR models for multiple response variables calculated separately for (six) species with more than 20 records showed apparent variation among species in the significance of habitat effects (Table 2). In three species (*G*. *campestris*, *G*. *gerbillus* and *G*. *occiduus*) strong and significant effects of habitat colouration were detected (explaining 8–17% of variation). But apparent lack of camouflage was detected in three other species. In two of them (*G*. *amoenus* and *G*. *pyramidum*), the lack of any significant PLSR components indicated no significant variables in the models, while in the third (*G*. *tarabuli*), a significant PLSR component indicated that habitat colouration was unrelated with animal fur colouration (Table 2).

**Table 2:** Partial least squares regression (PLSR) analyses results for the dorsal fur colour (synthetic colouration include RGB colours and total reflectance) and habitat colouration, geographic coordinates, field and collection affiliations for *Gerbillus*.

	*G. amoenus* ^*#*^		*G. campestris*		*G. gerbillus*		*G. occiduus*		*G. pyramidum* ^*#*^		*G. tarabuli*	
	W	Beta (±SE)	W	Beta (±SE)	W	Beta (±SE)	W	Beta (±SE)	W	Beta (±SE)	W	Beta (±SE)
log_10_ red habitat	−0.29	−0.12 (±0.084)	**0**.39*	0.09 (±0.016)	**0**.34*	0.09 (±0.017)	**0**.41**	0.14 (±0.016)	−0.35	−0.10 (±0.047)	0.02	0.01 (±0.049)
log_10_ green habitat	−0.08	−0.03 (±0.067)	**0**.37*	0.09 (±0.016)	**0**.32*	0.08 (±0.015)	**0**.38**	0.13 (±0.016)	−0.18	−0.05 (±0.051)	−0.03	−0.01 (±0.045)
log_10_ blue habitat	0.07	0.03 (±0.076)	**0**.34*	0.08 (±0.018)	**0**.28*	0.08 (±0.016)	**0**.39**	0.13 (±0.016)	−0.24	−0.07 (±0.049)	−0.03	−0.01 (±0.042)
log_10_ reflectance h.	−0.16	−0.07 (±0.070)	**0**.38*	0.09 (±0.016)	**0**.33*	0.09 (±0.015)	**0**.40**	0.14 (±0.016)	−0.26	−0.08 (±0.046)	−0.01	0.00 (±0.046)
latitude	−0.62	−0.25 (±0.111)	−**0**.42*	−0.10 (±0.019)	−0.22	−0.06 (±0.021)	−**0**.43**	−0.15 (±0.016)	−0.21	−0.06 (±0.084)	0.17	0.06 (±0.046)
longitude	−0.23	−0.09 (±0.085)	−0.16	−0.04 (±0.021)	−**0**.29*	−0.08 (±0.018)	−**0**.43**	−0.15 (±0.016)	−0.35	−0.10 (±0.074)	−**0**.48*	−0.16 (±0.047)
collection affiliation	0.11	0.05 (±0.077)	−0.11	−0.03 (±0.018)	−**0**.37*	−0.10 (±0.023)	—	—	0.36	0.10 (±0.113)	**−0**.56*	−0.19 (±0.046)
field collected	0.65*	0.26 (±0.074)	**0**.49**	0.12 (±0.017)	**0**.57**	0.15 (±0.022)	—	—	0.65	0.19 (±0.083)	**0**.65*	0.22 (±0.050)
explained variance (%)		24.5		58.0		53.4		96.8		38.7		43.14
N		37		99		122		24		72		70

Results are presented separate analyses for species with N > 20. Significance levels of regression coefficients of predictors: **<0.01, *<0.05. Explained variance (%) in the response variable by the PLSR model. W, weights of predictors’ contribution of variables to PLSR models, explaining more than 5% of the total variance are indicated in bold. ^#^No important PLSR component was detected for *G*. *amoenus* and *G*. *pyramidum* models. − indicates that the variable was not included in the model.

## Discussion

Our results showed significant variation in both habitat and animal colouration between closely related *Gerbillus* species (Fig. 2a). This first indicates that species differentiated their occupied niches, as defined here by habitat variation (exemplified by substrate colouration), and second that species evolved toward variable phenotypes, in terms of their coat colouration. We showed that habitat and phenotype colourations significantly covary across phylogenetic tree (Table 1; Fig. 2b), and within three out of six species with comprehensive datasets (Fig. 1, Table 2). The significant level of covariation between repeatable measures of variation in habitats and phenotypes is a strong indication of the existence of selective pressures related to camouflage in *Gerbillus*. The covariation is not masked by other factors, such as geographic origin of specimens, the phylogenetic relationship among species, or by differences between specimens originating from variable collection types. Moreover, the results from phylogenetic analyses, showing negative deviation from Brownian motion type of evolution (Fig. 3), also suggest adaptation in the studied phenotypic traits, at least in some of the lineages presented on the tree (Fig. 2a).

Phenotypic and habitat covariation showed that the magnitude of camouflage adaptation varies among species, and that it was lost/repeatedly acquired in three of the lineages (Fig. 2a). Spatial structure of habitat might have caused shifts in adaptive optima^26^. This may have caused different strengths of selection on fur colouration, and lead to variations in the covariation between phenotype and habitat (Table 2). Species occupying broader ranges usually inhabit a wider array of habitat types^43^, and local adaptation could be prevented if gene flow is significant between variable habitats^44^. Also, generalists species might find refuges from selective pressures, making them less susceptible to predation^34^. Indeed, in our dataset species with an apparent lack of phenotype-habitat covariation (*G*. *amoenus*, *G*. *pyramidum* and *G*. *tarabuli;* Table 2) tend to occupy a wide range of habitats^38^. Interspecific competition could have strengthened differences in the level of camouflage between gerbils, in cases where generalists are also dominant and if subordinates are exposed to increased foraging costs. According to the centrifugal community hypothesis proposed for *Gerbillus* ^45^ the two competing species share preference for the same primary habitat but differ in preference for less optimal, marginal habitats^46^. For most of *Gerbillus* species optimal habitats lay along a gradient from open to shrubby areas, defining a balance between resource availability (seed production/renewal and foraging efficiency in open areas) and safety from avian predators (in shrubs)^36^. If subordinate species are confined to more open habitats (also consistent with shared preference concept) they will suffer stronger selection from predation, reinforcing evolution of camouflage^17^. Unfortunately, the dominance hierarchy among *Gerbillus* species is not well established^36^. Thus, if open-space adapted species (*G*. *gerbillus* and, probably to a lesser extent, *G*. *occiduus* and *G*. *campestris*) suffer stronger selection for camouflage in the presence of dominant species is unknown. Indeed, benefits from camouflage adaptations could emerge simple because open-space species live in environments where hides are scarce.

Specimens tended to be brighter in colouration towards the equator (Table 1), a trend likely reflecting a climate-related and topographic environmental variability among sampling locations (although all were relatively dry and open habitats), that could have also reduced local adaptation. The so called Gloger’s rule, first described in birds, refer to not mutually exclusive mechanisms explaining geographic pattern in colouration, where darker animals are found in more humid environment^47^. It has been proposed that concealment (i.e. camouflage), protection against bacteria by structurally stronger feathers enriched with eumelanin (a darker form of melanin more resistant to physical damage) and thermoregulation can all account for latitudinal colour differences. A latitudinal pattern related to camouflage, also suggesting interplay between habitat and climatic variables, was shown in mice that increased fur brightness toward drier and more open geographic regions^48^. Since we have directly controlled for concealment/camouflage, and given that *Gerbillus* are nocturnal and non-basking mammals, the strengthening function of enriched with eumelanin hairs, and its interplay with isolation and camouflage functions^49^, could likely lay behind the observed geographic trends.

The adaptive variation among species in fur colour (Fig. 3b) suggests relatively easy evolutionary responses, an expected effect if i) selection is variable and occasionally strong, ii) a substantial proportion of phenotypic variation has a genetic background, and iii) fitness traits are not involved in strong trade-offs with colouration^50, 51^. The significant effect of phylogenetic information suggests that part of the differences observed between species is due to phylogenetic inertia (either direct on coat or indirect on habitat) related to genetic conservatism or convergent selection. The results indicate phylogenetic constraints for *G*. *amoenus*, *G*. *henleyi*, *G*. *simoni*, *G*. *campestris* and *G*. *nancillus* to increase fur reflectance (brightening), whereas decreasing reflectance (darkening) is constrained in the remaining species (Fig. 2a; Table 1). Significant constraints on colouration suggest that part of the colour inheritance is due to genetic mechanisms related to the expression of few colour genes (e.g. the well-known: Melanocortin-1 receptor and its antagonists Agouti signaling protein)^52, 53^. But camouflage is present in both brightening-constrained (*G*. *campestris*) and darkening-constrained (*G*. *gerbillus* and *G*. *occiduus*) species and in both relatively bright (*G*. *gerbillus* and *G*. *occiduus*) and dark (*G*. *campestris*) species (Fig. 2a). This and the continuous character of variation of coat colour (Fig. 2b) suggest that beside a few important genes, multigenetic (and epigenetic) regulatory mechanisms can be responsible for observed variation, that could facilitate adaptive evolution^24, 54^. The relative importance of environment and phylogeny suggests that environmental mechanisms are more essential in shaping colouration, at least in some parts of the *Gerbillus* phylogeny. The phylogenetic inertia explained around 9.6% of variation in fur colouration, compared to the 16.8% of inter-specific variation explained by habitat structure. Similar pattern is expected in traits with high genetic variation and little linkage between fitness-related characters^3^, suggesting that selection pressure on colour is independent from other selective forces e.g. those related to sexual selection^55^.

In conclusion, our results illustrated a broad scale of cryptic coloration in the *Gerbillus* genus, with a variable level of camouflage between species. The results showed that dorsal fur colouration and camouflage might have evolved repeatedly to match the colouration of the local habitat. This suggests that local selection is sometimes strong, and that the molecular mechanisms of coat colouration in *Gerbillus* are labile and unconstrained^56^. Pronounced and mosaic environmental variation in the Sahara-Sahel region occupied by *Gerbillus* species, even with apparent lack of geographic barriers, could promote local adaptation^26^. In philopatric organisms, selection against intermediate (hybrid) individuals, mismatching either of the neighbouring local habitats, could prompt emergence of reproductive isolation by evolution of assortative mating^57^. Therefore, the observed variation in habitat, and its covariation with phenotype, could promote speciation^58^. It may have been one of the processes at work that allowed *Gerbillus* to diversify into one of the most species-rich genera of mammals, with an estimated 60 species^37^. It is also worth noticing that this strong variation in *Gerbillus* species could represent an advantage that help them to adapt to the rapid habitat shifts^59^ predicted for the Sahara-Sahel region in response to ongoing climatic changes^60–62^.

## Materials and Methods

### Specimens and Species

*Gerbillus* species are arid and semi-arid habitat specialists, distributed from North and West Africa to western India through the Middle East^63^. We studied samples (n = 460; Fig. 1) belonging to fourteen North African *Gerbillus* species collected during field expeditions (n = 100) or housed in various museum collections (n = 360) at: CBGP - Center of Biology for the Management of Populations, Montferrier, France; NHM - Natural History Museum, Vienna, Austria; RMCA - Royal Museum for Central Africa, Tervuren, Belgium; MNA - Museum of Natural Sciences, Brussels, Belgium; ISEA - Institute of Systematics and Evolution of Animals, Polish Academy of Sciences, Kraków, Poland (Supplementary information: Data Description). We used only museum specimens that were preserved and stored without any chemical treatment as dried skins, and for which geographic coordinates or location description existed. Ear tissue samples were collected from live animals (n = 93) caught either with Sherman traps (Extra-Large Kangaroo Rat; placed every night in trap-line of 10–30 traps and collected every morning), with butterfly nets while walking at night, or from well preserved carcasses (n = 7). Species affiliations of all specimens were identified using external measurements (hind feet length, body mass, body and tail lengths, ear size) and other morphological features (presence of hair on foot soles, body colouration, and proportion of body parts)^38^. Samples were georeferenced using the global positioning system. To confirm species identification, DNA from 60 individuals was extracted and a species specific marker, cytochrome *b*, was amplified and sequenced with described procedures^31, 64^. Available sequences from 161 specimens from the CBGP collection were used to confirm species affiliations (Supplementary information: Data Description and Alignment.fasta). All methods were performed in accordance with the relevant guidelines and regulations, and all study protocols (e.g.: animal capturing, handling and tissue sampling) were approved by the Direction de la Lutte Contre la Désertification et la Protection de la Nature of Morocco (decision: 42/2014) and by the Ministère de l’Environnement et du Développement Durable of Mauritania (decision: 227/08.11.2012).

### Animal Colouration

Digital photography was used to estimate animal fur colouration^25, 30, 65^. Field and museum samples were photographed alongside a ColorChecker (X-Rite, Michigan, USA) with Canon EOS400D digital camera, 18–55 mm (set to 55 mm) 1:3.5–56 lens. Photographs were black and white-balance corrected (with GIMP 2.8 program) using the white and black references in the ColorChecker for light condition standardization. High-resolution images (TIFF format) were analysed with Hyper-Utility 2 program (www.fujifilm.com) to quantify fur colouration from a square-shaped area on the back of animal’s head (between the ears, pixel size: 40–150). To test for repeatability of colouration estimates (representativeness of dorsal colour), a total of 40 samples were randomly selected with “Sampling Design Tool” (in ArcGIS 10.1^66^) on which selected areas for colour estimation were moved to different locations, and calculations were repeated. Red, green and blue reflectance and total reflectance were measured as Red-Green-Blue (RGB, 8 bit, 0–255) standard values.

### Habitat Colouration

In order to estimate habitat colouration of the sample locations, remote sensing techniques were applied on NASA MODIS Terra satellite images. MODIS images (product name: MOD09A1 - Surface Reflectance 8-Day L3 Global) with a pixel resolution of 500^ × ^500 m were downloaded through USGS MODIS Reprojection Tool Web Interface (lpdaac.usgs.gov/data_access/mrtweb), covering the entire North Africa (total of 18 quadrants). Satellite images corresponded to the peak of the dry season in the study area (May^67, 68^), in order to represent substrate colouration without the colour noise from water features and developed vegetation. Overall, 45 images per location (three per month of May) between 2000 and 2014 were obtained. Only images without scanning problems and with less than 10% of total cloud cover were used to calculate average substrate reflectance parameters, resulting in a total of more than 12 images per quadrant. The image bands used were “sur_refl_b01_1”, “sur_refl_b04_1” and “sur_refl_b0351”, which correspond to the visible reflectance of red, green and blue colours, respectively. The “Cell Statistics” tool of ArcGIS was used to calculate the mean values of red, green and blue reflectance for each pixel per quadrant. The quadrants were merged to form a single image with the temporal reflectance mean (2000–2014) of the region using “Mosaic” tool of ArcGIS. The landscape reflectance was measured as the average pixel value for 1 km radius around the sample origin, a representative area to estimate variance in habitat given the mobility capacity of sampled individuals/species that usually do not move more than few hundred meters from their burrow^69, 70^. Locations of samples collected near infrastructures and near water courses were manually moved (few hundred meters) to avoid unnatural habitats from being included in analyses. The total reflectance (L) was calculated as a weighted average, as ITU-R BT.601 standard coefficient [International Telecommunications Union 2011: www.itu.int; using the formula: L = (0.299 R + 0.587 G + 0.114 B), where R, G and B represent the reflectance of red, green and blue, respectively]. For repeatability estimates, i.e. consistency of habitat colouration over time, images were divided in two groups: 2000–2006 and 2007–2014.

### Statistical Analysis

Repeatability of fur and habitat colouration was estimated with the intraclass correlation coefficient (τ) based on between and within group mean squares extracted from ANOVA/ANCOVA analysis^71–73^. Correlations between fur and habitat colouration were tested with partial Pearson’s product-moment correlations controlling for the sample origin (museum *vs*. field collections) in R 3.1.3 (http://www.r-project.org/) with “ppcor” package. Analysis of variance was used to test the significance of differences between species in average habitat and fur colourations, controlling for variation between collection/field types of specimens.

Phylogenetic vector regression (PVR) is an optimal method to detect phylogenetic inertia on datasets containing a relatively small numbers of species (e.g. 10, our study includes 14 species) and when phylogenetic inertia is expected to be relatively low^4, 74, 75^. It has been shown that in such cases PVR returns good statistical performance irrespective of evolutionary models used and suffers very low type I and II errors, in comparison to alternative methods (autoregressive method, Felsenstein’s independent contrasts and phylogenetic generalized least squares)^76, 77^. Moreover PVR does not assume any evolutionary model for a given data, which is advantageous in poorly studied organisms^76, 78^ like in *Gerbillus* rodents. Finally, PVR decomposes phylogenetic distance matrices into the phylogenetic eigenvectors that can be included in statistical modelling of predictor variables^4, 75^. The phylogenetic eigenvectors for *Gerbillus* species were generated while including the species’ mean phenotypic values (fur reflectance and RGB colours) in R 3.1.3 “PVR” package. The phylogenetic relationship between species (Fig. 2) was derived from published phylogenies^31, 32, 79, 80^. As phylogenetic position of *G*. *nancillus* (subgenus *Monodia*) is not resolved with statistical support, alternative phylogenetic hypotheses were tested (Supplementary information: Table S4). The tree topology and branch lengths were used to test the concordance of mode of evolution in the studied traits and Brownian motion evolutionary model by estimating phylogenetic signal-representation (PSR) curves^81^, and to select phylogenetic eigenvectors for statistical modelling^75^. In cases when traits follow non-linear model of evolution, only part of the eigenvectors can be used to describe the phylogenetic relatedness in comparative analyses^81^. From the generated phylogenetic eigenvectors, those that reduce the largest amount of autocorrelation in the residuals, below statistical significance threshold of the Moran’s I test, shall only be included^81^. The phylogenetic inertia is tested here by estimating sign and strength of phylogenetic signal, an area under the phylogenetic signal-representation (PSR) curve. The PSR curve is built from sequential PVR models with increasing number of phylogenetic eigenvectors included, and by plotting eigenvectors R^2^ against their accumulated eigenvalues. The area under PVR curve describes deviations from Brownian motion, i.e. nonlinear curves reveal if traits evolved at a slower or higher rate than expected. We constructed PVR curves for studied traits (animal colouration)^3, 4, 29^ potentially related to adaptation (estimated by co-variation with habitat colouration) invoking camouflage (animal-habitat colour matching).

Due to multicollinearity in our dataset, the relationship between the response variables (animal fur reflectance and RGB colours), habitat variables (substrate reflectance and RGB colours) and phylogenetic signal (represented by phylogenetic eigenvectors) was tested on comprehensive sample size (n = 460) with partial least squares regression (PLSR), a method extremely resilient to the correlations between predictor variables^82^. The high and significant correlations between predictor variables (for habitat RGB colours and total reflectance: r > 0.87, df = 459, p < 0.0001; for habitat and latitude: r < −0.28, df = 459, p < 0.0001; for habitat and some of eigenvectors: r = −0.27, df = 459, p < 0.0001) makes PLSR the most appropriate method for our analyses. The PLSR models included independent variables of: habitat colouration (red, green, blue and total reflectance), geographic location affiliation (latitude and longitude), museum collection affiliation (seven levels), field affiliation (two levels) and selected in PVR analysis phylogenetic eigenvectors. As dependent variables fur colouration traits were included (red, green blue and total reflectance), either in four separate analysis for each colour band and total reflectance, or in one multiple response variables model. In the case of the multiple response variable model a synthetic response variable is predicted from a linear combination of the original response traits (here red, green blue colours and total reflectance). PLSR calculates components from predictor variables in a way to maximize explained variation in response traits, and that components are used to test for associations between variables. The cross-validation test of the parameter Q^2^ was carried out to determine if PLSR components carry significant signals explaining variation in response variables of animal colouration. Correlation between PLSR scores for response variables and PLSR component scores was estimated to test the significance of the explained variance in response variables. The relative contribution of each variable to the PLSR components by means of the square of its weight were estimated, considering that a predictor was important if it accounted for more than 5% of the variance in the response. The statistical significance of regression coefficients from the PLSR models of the predictors was performed by bootstrapping with 1000 replications. PLSR analyses were performed in R 3.1.3 with “plsRglm” and “pls” packages. Analyses were conducted on log transformed continuous variables (i.e. fur and habitat colouration traits).

## Electronic supplementary material

Supplementary information
