# Supplementary material for: Repeated evolution of camouflage in speciose desert rodents
Source: Sci Rep. 2017 Jun 14;7:3522. doi: 10.1038/s41598-017-03444-y (PMC5471182; doi:10.1038/s41598-017-03444-y)
Supplement: Supplementary file 1 — Supplementary information [file 41598_2017_3444_MOESM1_ESM.pdf]

Supplementary information

Repeated evolution of camouflage in speciose desert rodents

Boratyński Zbyszek[1*], Brito C. José[1,2], Campos C. João[1,2], Cunha L. José[1], Granjon Laurent[3], Mappes Tapio[4], Ndiaye Arame[5], Rzebik-Kowalska Barbara[6], Serén Nina[1]

[1] CIBIO/InBio, Research Center in Biodiversity and Genetic Resources, University of Porto, Vairão, 4485-661 Vairão, Portugal

[2] Department of Biology, Faculty of Science, University of Porto, Rua Campo Alegre, 4169-007 Porto, Portugal

[3] IRD, UMR CBGP (INRA/IRD/CIRAD/MontpellierSupAgro), Campus International de Baillarguet, CS 30016, 34988 Montferrier-sur-Lez cedex, France

[4] Department of Biological and Environmental Science, University of Jyväskylä, P.O. Box 35, 40014, Finland

[5] Department of Animal Biology, Faculty of Sciences and Technologies, University Cheikh Anta Diop, BP 5005 Dakar, Senegal

[6] Institute of Systematics and Evolution of Animals, Polish Academy of Sciences, Sławkowska 17, 31-016, Kraków, Poland

* Authors are in alphabetical order, after corresponding author: boratyns@cibio.up.pt

Contents:

Table S1 --------- page 2

Table S2 --------- page 2

Table S3 --------- page 3

Table S4 -------------- page 4

Data Description ---- page 5

Alignment.fasta ----- page 16

Table S1. Repeatability (intraclass correlation coefficients) for fur and habitat colourations derived from repeated measurements and of total reflectance and RGB (red, green and blue) reflectance.

	animal fur			habitat (1 km radius)		
	df	τ	p	df	τ	p
$\log_{10}$ reflectance	39	1.00	<0.0001	39	0.98	<0.0001
$\log_{10}$ red	39	0.99	<0.0001	39	0.98	<0.0001
$\log_{10}$ green	39	1.00	<0.0001	39	0.97	<0.0001
$\log_{10}$ blue	39	0.99	<0.0001	39	0.90	<0.0001

Table S2. Correlations (Pearson's partial correlation accounted for collection type) between fur and habitat total reflectance calculated separately for 9 species with N > 4.

species	N	r	p
G. amoenus	37	-0.18	0.276
G. campestris	99	**0.40**	<0.0001
G. gerbillus	122	**0.26**	0.005
G. hoogstrali	6	-0.55	0.254
G. nigeriae	9	0.37	0.363
G. occiduus	24	**0.85**	<0.0001
G. pyramidum	72	-0.18	0.126
Gerbillus sp.	5	**0.87**	0.054
G. tarabuli	70	0.18	0.137

Table S3. Partial least squares regression (PLSR) analyses results for the dorsal fur colour (synthetic colouration include RGB colours and total reflectance) and habitat colouration, geographic coordinates, field/collection affiliations and phylogenetic eigenvectors for *Gerbillus*. Results are presented for barcoded specimens only (left column; N = 221), for analyses conducted on residual dorsal fur colour (synthetic colouration include residual values of RGB colours and total reflectance calculated from linear regression models that included phylogenetic eigenvectors) and for analysis including 10 eigenvectors. Significance levels of regression coefficients of predictors: *** < 0.001, ** < 0.01, * <0.05. Explained variance (%) in the response variable by the PLSR model. W, weights of predictors' contribution of variables to PLSR models, explaining more than 5% of the total variance are indicated in bold.

	on barcoded samples		on residuals colours		with 10 eingenvectors				
	W	Beta(±SE)	W	Beta(±SE)	W	Beta(±SE)		W	Beta(±SE)
log_{10} red habitat	**0.34****	0.13(±0.023)	**0.41****	0.11(±0.010)	**0.32*****	0.09(±0.007)	EV2	-0.14**	-0.04(±0.009)
Log_{10} green habitat	**0.25****	0.10(±0.022)	**0.37****	0.10(±0.009)	**0.30*****	0.09(±0.006)	EV3	**-0.22*****	-0.07(±0.007)
log_{10} blue habitat	**0.25***	0.10(±0.025)	**0.35****	0.09(±0.010)	**0.29*****	0.09(±0.007)	EV4	**-0.35*****	-0.11(±0.007)
log_{10} reflectance h.	**0.29****	0.11(±0.022)	**0.39****	0.11(±0.009)	**0.31*****	0.09(±0.006)	EV5	-0.19***	-0.06(±0.008)
latitude	**-0.25***	-0.10(±0.031)	**-0.36****	-0.10(±0.012)	**-0.26*****	-0.08(±0.008)	EV6	0.18***	0.05(±0.006)
longitude	**-0.41****	-0.16(±0.036)	-0.21*	-0.06(±0.014)	-0.15**	-0.04(±0.009)	EV7	0.10*	0.03(±0.009)
collection affiliation	**-0.36***	-0.14(±0.042)	-0.12	-0.03(±0.015)	0.02	0.01(±0.009)	EV8	**-0.36*****	-0.11(±0.007)
field collected	**0.53****	0.21(±0.039)	**0.48****	0.13(±0.015)	**0.27*****	0.08(±0.009)	EV9	-0.04	-0.01(±0.006)
EV1	-0.20	-0.08(±0.028)	-	-	**-0.24*****	-0.07(±0.007)	EV10	-0.02	-0.01(±0.005)
Explained variance (%)		30.3		46.7		28.2			

Table S4. Partial least squares regression (PLSR) analyses results for the dorsal fur colour (synthetic colouration include RGB colours and total reflectance) and habitat colouration, geographic coordinates, field/collection affiliations and phylogenetic eigenvectors for *Gerbillus*. Results are presented for analyses including eigenvectors derived for alternative phylogenetic positions of *G. nancillus* (subgenus *Monodia*) relative to other subgenera of *Gerbillus.* Significance levels of regression coefficients of predictors: *** < 0.001, ** < 0.01, * <0.05. Explained variance (%) in the response variable by the PLSR model. W, weights of predictors' contribution of variables to PLSR models, explaining more than 5% of the total variance are indicated in bold.

	Monodia-Dipodillus(Gerbillus)		Monodia(Dipodillus-Gerbillus)	
	W	Beta(±SE)	W	Beta(±SE)
log_{10} red habitat	**0.41*** **	0.12(±0.008)	**0.42*** **	0.12(±0.009)
Log_{10} green habitat	**0.39*** **	0.11(±0.008)	**0.40*** **	0.11(±0.008)
log_{10} blue habitat	**0.37*** **	0.11(±0.009)	**0.38*** **	0.11(±0.009)
log_{10} reflectance h.	**0.41*** **	0.12(±0.008)	**0.41*** **	0.12(±0.008)
latitude	**-0.34** **	-0.10(±0.010)	**-0.35** **	-0.10(±0.010)
longitude	-0.19*	-0.06(±0.012)	-0.19*	-0.06(±0.012)
collection affiliation	0.02	0.01(±0.011)	0.02	0.01(±0.012)
field collected	**0.35** **	0.10(±0.012)	**0.35** **	0.10(±0.012)
EV1	**-0.31** **	-0.09(±0.008)	**-0.28** **	-0.08(±0.008)
Explained variance (%)		45.3		44.9

Data Description. Geographic sampling location, sample and GenBank affiliation numbers. CBGP - Center for Biology and Management of Populations, Montferrier, France; NHM - Natural History Museum, Vienna, Austria; RMCA - Royal Museum for Central Africa, Tervuren, Belgium; MNA - Museum of Natural Sciences, Brussels, Belgium; ISEA - Institute of Systematics and Evolution of Animals, Polish Academy of Sciences, Kraków, Poland.

ID	latitude	longitude	country	collection	species	Barcoded	sequences in
15189	30.82	14.28	Libya	NHM	campestris	1	alignment.fasta
25768	24.911	17.759	Libya	NHM	campestris	1	alignment.fasta
8249	22.12	31.32	Sudan	NHM	pyramidum	1	alignment.fasta
8248	22.12	31.32	Sudan	NHM	gerbillus	1	alignment.fasta
25754	25.9	13.85	Libya	NHM	gerbillus	1	alignment.fasta
37777	21.835	25.019	Sudan	RMCA	campestris	1	alignment.fasta
37799	22.003604	25.144722	Egypt	RMCA	gerbillus	1	alignment.fasta
5044	29.1625	33.626389	Egypt	RMCA	gerbillus	1	alignment.fasta
37796	22.003604	25.144722	Egypt	RMCA	gerbillus	1	alignment.fasta
5035	18.476	31.832	Sudan	RMCA	amoenus	1	alignment.fasta
5050	32.752	-0.599	Algeria	RMCA	latastei	1	alignment.fasta
91-090-M-288	32.5	20.83	Libya	RMCA	campestris	1	alignment.fasta
17927	30.78	17.83	Libya	MNA	tarabuli	1	alignment.fasta
17240	19.098	12.916	Niger	MNA	campestris	1	alignment.fasta
21983	33.001	11.248	Tunisia	MNA	campestris	1	alignment.fasta
17002	21.963	24.82	Libya	MNA	amoenus	1	alignment.fasta
22135	36.8	10.1833	Tunisia	MNA	amoenus	1	alignment.fasta
M/9701/90	34.433336	2.516674	Algeria	ISEA	simoni	1	alignment.fasta
M/9041/89	36.426503	2.757637	Algeria	ISEA	campestris	1	alignment.fasta
M/9069/89	24.483	9.699	Algeria	ISEA	campestris	1	alignment.fasta
M/9120/89	26.00001	3.50001	Algeria	ISEA	campestris	1	alignment.fasta
M/9192/89	28.250021	-0.200006	Algeria	ISEA	gerbillus	1	alignment.fasta
M/9703/90	35.089572	3.029872	Algeria	ISEA	henleyi	1	alignment.fasta
M/9816/90	33.8	2.9	Algeria	ISEA	amoenus	1	alignment.fasta
M/10016/90	35.089572	3.029872	Algeria	ISEA	latastei	1	alignment.fasta
M/1011/90	22.335	5.551	Algeria	ISEA	pyramidum	1	alignment.fasta
ZBSC0055	17.162042	-12.128122	Mauritania	field	campestris	1	alignment.fasta
ZBSC0058	17.422828	-13.435163	Mauritania	field	nancillus	1	alignment.fasta
ZBSC0059	17.422828	-13.435163	Mauritania	field	tarabuli	1	alignment.fasta
ZBSC0060	17.422828	-13.435163	Mauritania	field	nigeriae	1	alignment.fasta
ZBSC0061	17.422828	-13.435163	Mauritania	field	tarabuli	1	alignment.fasta
ZBSC0062	17.422828	-13.435163	Mauritania	field	tarabuli	1	alignment.fasta
ZBSC0067	19.808792	-14.288472	Mauritania	field	henleyi	1	alignment.fasta
ZBSC0068	20.55489	-16.155358	Mauritania	field	tarabuli	1	alignment.fasta

ZBSC0071	20.611237	-16.01271	Mauritania	field	gerbillus	1	alignment.fasta
ZBSC0073	19.851582	-15.646828	Mauritania	field	gerbillus	1	alignment.fasta
ZBSC0200	21.2802929	-16.0917824	Mauritania	field	gerbillus	1	alignment.fasta
ZBSC0201	21.2802929	-16.0917824	Mauritania	field	gerbillus	1	alignment.fasta
ZBSC0202	21.2802929	-16.0917824	Mauritania	field	gerbillus	1	alignment.fasta
ZBSC0203	21.2802929	-16.0917824	Mauritania	field	gerbillus	1	alignment.fasta
ZBSC0204	21.2802929	-16.0917824	Mauritania	field	tarabuli	1	alignment.fasta
ZBSC0206	21.1974857	-14.2221218	Mauritania	field	gerbillus	1	alignment.fasta
ZBSC0207	21.1974857	-14.2221218	Mauritania	field	gerbillus	1	alignment.fasta
ZBSC0208	21.1974857	-14.2221218	Mauritania	field	gerbillus	1	alignment.fasta
ZBSC0209	21.1974857	-14.2221218	Mauritania	field	gerbillus	1	alignment.fasta
ZBSC0212	21.5194373	-12.8528308	Mauritania	field	gerbillus	1	alignment.fasta
ZBSC0213	21.5194373	-12.8528308	Mauritania	field	amoenus	1	alignment.fasta
ZBSC0215	21.5194373	-12.8528308	Mauritania	field	tarabuli	1	alignment.fasta
ZBSC0216	21.7751568	-12.8794379	Mauritania	field	campestris	1	alignment.fasta
ZBSC0217	21.4380974	-12.98	Mauritania	field	campestris	1	alignment.fasta
ZBSC0225	20.5507976	-12.676174	Mauritania	field	campestris	1	alignment.fasta
ZBSC0227	21.3728067	-11.9104643	Mauritania	field	gerbillus	1	alignment.fasta
ZBSC0228	21.0179944	-11.9248855	Mauritania	field	amoenus	1	alignment.fasta
ZBSC0229	21.0179944	-11.9248855	Mauritania	field	amoenus	1	alignment.fasta
ZBSC0230	21.0179944	-11.9248855	Mauritania	field	tarabuli	1	alignment.fasta
ZBSC0231	21.0179944	-11.9248855	Mauritania	field	campestris		
ZBSC0232	21.0179944	-11.9248855	Mauritania	field	tarabuli	1	alignment.fasta
ZBSC0233	21.0179944	-11.9248855	Mauritania	field	campestris	1	alignment.fasta
ZBSC0234	21.0179944	-11.9248855	Mauritania	field	pyramidum	1	alignment.fasta
ZBSC0235	21.0179944	-11.9248855	Mauritania	field	pyramidum	1	alignment.fasta
ZBSC0238	20.2650502	-13.2075922	Mauritania	field	campestris	1	alignment.fasta
ZBSC0239	20.2650502	-13.2075922	Mauritania	field	amoenus	1	alignment.fasta
ZBSC0248	19.851327	-16.357723	Mauritania	field	nigeriae	1	alignment.fasta
ZBSC0249	19.7400604	-16.2746175	Mauritania	field	gerbillus	1	alignment.fasta
ZBSC0250	19.7400604	-16.2746175	Mauritania	field	gerbillus	1	alignment.fasta
ZBSC0251	19.7400604	-16.2746175	Mauritania	field	gerbillus	1	alignment.fasta
ZBSC0254	17.3639094	-13.5720984	Mauritania	field	tarabuli	1	alignment.fasta
ZBSC0259	17.8880876	-12.1150911	Mauritania	field	campestris	1	alignment.fasta
ZBSC0264	18.0535711	-11.9440143	Mauritania	field	campestris	1	alignment.fasta
ZBSC0282	29.5406159	-8.0248397	Morocco	field	tarabuli	1	alignment.fasta
ZBSC0284	28.7582909	-9.1267523	Morocco	field	tarabuli	1	alignment.fasta
ZBSC0285	27.921633	-10.0007429	Morocco	field	tarabuli	1	alignment.fasta
ZBSC0287	27.1529816	-10.847235	WesternSahara	field	amoenus	1	alignment.fasta
ZBSC0288	27.153484	-10.847211	WesternSahara	field	tarabuli	1	alignment.fasta
ZBSC0297	23.7841931	-14.7938761	WesternSahara	field	gerbillus	1	alignment.fasta
ZBSC0298	23.579987	-15.2325639	WesternSahara	field	gerbillus	1	alignment.fasta
ZBSC0299	23.579987	-15.2325639	WesternSahara	field	amoenus	1	alignment.fasta
ZBSC0300	23.579987	-15.2325639	WesternSahara	field	gerbillus	1	alignment.fasta

ZBSC0301	23.256867	-15.887359	WesternSahara	field	gerbillus	1	alignment.fasta
ZBSC0321	15.9994396	-11.8726127	Mauritania	field	nigeriae	1	alignment.fasta
ZBSC0322	15.9994396	-11.8726127	Mauritania	field	nigeriae	1	alignment.fasta
ZBSC0338	16.0532808	-11.6699325	Mauritania	field	nigeriae	1	alignment.fasta
ZBSC0339	16.0532808	-11.6699325	Mauritania	field	nigeriae	1	alignment.fasta
ZBSC0344	16.0532808	-11.6699325	Mauritania	field	nigeriae	1	alignment.fasta
ZBSC0348	16.4442935	-11.7780408	Mauritania	field	campestris	1	alignment.fasta
ZBSC0369	16.7629369	-11.2219761	Mauritania	field	henleyi	1	alignment.fasta
ZBSC0370	16.7629369	-11.2219761	Mauritania	field	nigeriae	1	alignment.fasta
ZBSC0402	27.8446312	-12.9141547	Morocco	field	gerbillus		
ZBSC0416	22.6110729	-14.4709096	WesternSahara	field	campestris		
ZBSC0486	16.4841416	-9.2895905	Mauritania	field	tarabuli		
ZBSC0489	17.5891599	-7.4459693	Mauritania	field	campestris		
ZBSC0490	17.5891599	-7.4459693	Mauritania	field	gerbillus		
ZBSC0491	18.0995765	-8.0107303	Mauritania	field	gerbillus		
ZBSC0492	18.0995765	-8.0107303	Mauritania	field	gerbillus		
ZBSC0493	18.0995765	-8.0107303	Mauritania	field	gerbillus		
ZBSC0494	18.0995765	-8.0107303	Mauritania	field	campestris		
ZBSC0496	18.3807983	-8.2721973	Mauritania	field	gerbillus		
ZBSC0497	18.3837159	-8.5216775	Mauritania	field	gerbillus		
ZBSC0498	18.3837159	-8.5216775	Mauritania	field	gerbillus		
ZBSC0499	18.3837159	-8.5216775	Mauritania	field	gerbillus		
ZBSC0500	18.3837159	-8.5216775	Mauritania	field	gerbillus		
ZBSC0503	18.3674677	-9.0485299	Mauritania	field	gerbillus		
ZBSC0504	18.3674677	-9.0485299	Mauritania	field	gerbillus		
ZBSC0505	18.3674677	-9.0485299	Mauritania	field	gerbillus		
ZBSC0507	18.3674677	-9.0485299	Mauritania	field	campestris		
ZBSC0513	18.5787191	-9.8186112	Mauritania	field	gerbillus		
ZBSC0514	18.5787191	-9.8186112	Mauritania	field	gerbillus		
ZBSC0529	18.4428214	-11.3873945	Mauritania	field	campestris		
ZBSC0530	18.4428214	-11.3873945	Mauritania	field	campestris		
ZBSC0534	18.4428214	-11.3873945	Mauritania	field	campestris		
ZBSC0535	18.4428214	-11.3873945	Mauritania	field	campestris		
ZBSC0541	18.3568223	-11.8161066	Mauritania	field	campestris		
ZBSC0548	18.3568223	-11.8161066	Mauritania	field	campestris		
ZBSC0549	18.3568223	-11.8161066	Mauritania	field	campestris		
ZBSC0553	18.2062343	-11.7303343	Mauritania	field	campestris		
ZBSC0554	18.2062343	-11.7303343	Mauritania	field	campestris		
ZBSC0555	18.2062343	-11.7303343	Mauritania	field	campestris		
ZBSC0556	18.2062343	-11.7303343	Mauritania	field	gerbillus		
ZBSC0564	18.1505083	-12.0657156	Mauritania	field	campestris		
ZBSC0565	18.1505083	-12.0657156	Mauritania	field	campestris		
ZBSC0566	18.1505083	-12.0657156	Mauritania	field	campestris		
ZBSC0567	18.1505083	-12.0657156	Mauritania	field	campestris		

ZBSC0571	20.7304184	-16.0248282	Mauritania	field	gerbillus
ZBSC0572	20.7304184	-16.0248282	Mauritania	field	gerbillus
ZBSC0592	32.2539635	-2.1878765	Morocco	field	tarabuli
ZBSC0593	32.2573519	-2.2407148	Morocco	field	pyramidum
M/10001/90	23.700076	5.133385	Algeria	ISEA	pyramidum
M/10005/90	22.89755	5.37775	Algeria	ISEA	pyramidum
M/10013/90	25.3	3.7336	Algeria	ISEA	pyramidum
M/10014/90	34.433336	2.516674	Algeria	ISEA	pyramidum
M/10015/90	33.107492	1.267227	Algeria	ISEA	pyramidum
M/9038/89	35.7706	-0.8032	Algeria	ISEA	campestris
M/9040/89	35.150019	-1.450013	Algeria	ISEA	campestris
M/9044/89	35.483347	-0.51668	Algeria	ISEA	campestris
M/9045/89	30.138	-2.164	Algeria	ISEA	campestris
M/9048/89	33.107492	1.267227	Algeria	ISEA	campestris
M/9049/89	24.650017	9.633348	Algeria	ISEA	campestris
M/9051/89	24.648	9.629	Algeria	ISEA	campestris
M/9057/89	24.673	9.729	Algeria	ISEA	campestris
M/9058/89	24.471316	9.682003	Algeria	ISEA	campestris
M/9067/89	24.483369	9.700033	Algeria	ISEA	campestris
M/9068/89	24.483	9.699	Algeria	ISEA	campestris
M/9072/89	24.566669	9.483336	Algeria	ISEA	campestris
M/9073/89	32.732	-0.166	Algeria	ISEA	campestris
M/9079/89	35.1666	-1.6501	Algeria	ISEA	campestris
M/9083/89	32.766689	-0.416679	Algeria	ISEA	campestris
M/9087/89	26.716677	0.166699	Algeria	ISEA	campestris
M/9091/89	35.64	-0.77	Algeria	ISEA	campestris
M/9093/89	32.732	-0.166	Algeria	ISEA	campestris
M/9094/89	35.593053	-0.414676	Algeria	ISEA	campestris
M/9095/89	35.450003	-0.016669	Algeria	ISEA	campestris
M/9100/89	30.916676	-2.033343	Algeria	ISEA	campestris
M/9107/89	33.800095	2.900253	Algeria	ISEA	campestris
M/9110/89	30.591184	2.896947	Algeria	ISEA	campestris
M/9111/89	28.016	2.667	Algeria	ISEA	campestris
M/9113/89	25.000001	4.33333	Algeria	ISEA	campestris
M/9114/89	23.700076	5.133385	Algeria	ISEA	campestris
M/9116/89	23.131294	5.743068	Algeria	ISEA	campestris
M/9118/89	22.89755	5.37775	Algeria	ISEA	campestris
M/9119/89	25.3	3.7336	Algeria	ISEA	campestris
M/9123/89	34.166677	0.083349	Algeria	ISEA	campestris
M/9125/89	34.166677	0.083349	Algeria	ISEA	campestris
M/9127/89	35.216667	-0.750003	Algeria	ISEA	campestris
M/9129/89	35.666	-0.999	Algeria	ISEA	campestris
M/9134/89	30.138	-2.164	Algeria	ISEA	gerbillus
M/9136/89	29	-0.25	Algeria	ISEA	gerbillus

M/9158/89	31.183333	-2.25	Algeria	ISEA	gerbillus
M/9163/89	30.138	-2.164	Algeria	ISEA	gerbillus
M/9169/89	24.471316	9.682003	Algeria	ISEA	gerbillus
M/9171/89	24.483	9.699	Algeria	ISEA	gerbillus
M/9174/89	24.566669	9.483336	Algeria	ISEA	gerbillus
M/9175/89	24.566669	9.483336	Algeria	ISEA	gerbillus
M/9191/89	29.666	-1.668	Algeria	ISEA	gerbillus
M/9195/89	26.716677	0.166699	Algeria	ISEA	gerbillus
M/9200/89	28.88999	1.97755	Algeria	ISEA	gerbillus
M/9205/89	30.716667	3.133333	Algeria	ISEA	gerbillus
M/9207/89	33.107492	1.267227	Algeria	ISEA	gerbillus
M/9209/89	33.107492	1.267227	Algeria	ISEA	gerbillus
M/9217/88	28.016672	2.666676	Algeria	ISEA	gerbillus
M/9218/89	26.5002	3.05002	Algeria	ISEA	gerbillus
M/9219/89	25.000001	4.33333	Algeria	ISEA	gerbillus
M/9227/89	23.700076	5.133385	Algeria	ISEA	gerbillus
M/9229/89	22.333348	5.550009	Algeria	ISEA	gerbillus
M/9235/89	25.3	3.7336	Algeria	ISEA	gerbillus
M/9237/89	26.00001	3.50001	Algeria	ISEA	gerbillus
M/9699/90	34.8334	3.1167	Algeria	ISEA	simoni
M/9804/90	33.107492	1.267227	Algeria	ISEA	amoenus
M/9805/90	31.183333	-2.25	Algeria	ISEA	amoenus
M/9806/90	30.138	-2.164	Algeria	ISEA	amoenus
M/9810/90	24.566669	9.483336	Algeria	ISEA	amoenus
M/9812/90	33.166731	1.583348	Algeria	ISEA	amoenus
M/9813/90	28.88999	1.97755	Algeria	ISEA	amoenus
M/9817/90	32.49058	3.715209	Algeria	ISEA	amoenus
M/9821/90	30.591184	2.896947	Algeria	ISEA	amoenus
M/9822/90	23.700076	5.133385	Algeria	ISEA	amoenus
M/9823/90	22.333348	5.550009	Algeria	ISEA	amoenus
M/9824/90	26.00001	3.50001	Algeria	ISEA	amoenus
M/9945/90	32.747	-0.591	Algeria	ISEA	pyramidum
M/9953/90	29	-0.25	Algeria	ISEA	pyramidum
M/9966/90	31.183333	-2.25	Algeria	ISEA	pyramidum
M/9968/90	30.138	-2.164	Algeria	ISEA	pyramidum
M/9980/90	33.166676	1.583343	Algeria	ISEA	pyramidum
M/9981/90	32.766689	-0.416679	Algeria	ISEA	pyramidum
M/9985/90	26.716677	0.166699	Algeria	ISEA	pyramidum
M/9986/90	33.8	2.9	Algeria	ISEA	pyramidum
M/9987/90	34.166677	0.083349	Algeria	ISEA	pyramidum
M/9991/90	32.49058	3.715209	Algeria	ISEA	pyramidum
M/9994/90	30.591184	2.896947	Algeria	ISEA	pyramidum
M/9996/90	25.000001	4.33333	Algeria	ISEA	pyramidum
17003	21.963	24.82	Libya	MNA	campestris

17004	21.831	24.985	Sudan	MNA	campestris
17005	21.831	24.985	Sudan	MNA	campestris
17021	21.96	25.13	Libya	MNA	gerbillus
17024	22.037246	25.09651	Egypt	MNA	gerbillus
17025	22.011	24.96	Libya	MNA	gerbillus
17026	22.011	24.96	Libya	MNA	gerbillus
17230	21.016	12.301	Niger	MNA	gerbillus
17231	33.883	7.366	Algeria	MNA	gerbillus
17232	33.333	6.8833	Algeria	MNA	gerbillus
17233	33.333	6.8833	Algeria	MNA	gerbillus
17234	21.016	12.301	Niger	MNA	gerbillus
17238	19.098	12.916	Niger	MNA	pyramidum
17239	32.0001	5.2666	Algeria	MNA	campestris
17241	19.098	12.916	Niger	MNA	campestris
17243	18.67	12.9	Niger	MNA	amoenus
17908	30.78	17.83	Libya	MNA	gerbillus
17909	30.78	17.83	Libya	MNA	gerbillus
17948	30.78	17.83	Libya	MNA	tarabuli
17951	30.78	17.83	Libya	MNA	tarabuli
17959	30.78	17.83	Libya	MNA	tarabuli
17966	30.78	17.83	Libya	MNA	tarabuli
17967	30.78	17.83	Libya	MNA	tarabuli
17996	30.78	17.83	Libya	MNA	andersoni
17997	30.93	17.76	Libya	MNA	andersoni
18079	30.78	17.83	Libya	MNA	gerbillus
18080	30.78	17.83	Libya	MNA	andersoni
18612	18	8	Niger	MNA	amoenus
18613	18.12	8.78	Niger	MNA	pyramidum
18620	18.12	8.78	Niger	MNA	amoenus
18622	18.583	8.783	Niger	MNA	pyramidum
18623	18.12	8.78	Niger	MNA	pyramidum
21967	36.4	10.616	Tunisia	MNA	campestris
21968	36.8	10.1833	Tunisia	MNA	campestris
21969	37.2	9.7	Tunisia	MNA	campestris
21970	37.2	9.7	Tunisia	MNA	campestris
21972	36.4	10.616	Tunisia	MNA	campestris
21978	36.8	10.1833	Tunisia	MNA	campestris
21980	36.18	8.72	Tunisia	MNA	campestris
22022	36.94	10.19	Tunisia	MNA	latastei
22023	36.94	10.19	Tunisia	MNA	campestris
22126	36.8	10.1833	Tunisia	MNA	campestris
22130	36.8	10.1833	Tunisia	MNA	campestris
22131	33.88	10.85	Tunisia	MNA	campestris
22132	36.8	10.1833	Tunisia	MNA	campestris

22133	36.8	10.1833	Tunisia	MNA	campestris		
22134	36.8	10.1833	Tunisia	MNA	pyramidum		
22136	36.4333	10.583	Tunisia	MNA	gerbillus		
5043	29.9	31.4	Egypt	RMCA	pyramidum		
5045	29.1625	33.626389	Egypt	RMCA	gerbillus		
5048	15.52	32.596	Sudan	RMCA	pyramidum		
5049	19.66	30.4391	Sudan	RMCA	pyramidum		
37778	22.037246	25.09651	Egypt	RMCA	campestris		
37800	22.003604	25.144722	Egypt	RMCA	gerbillus		
37801	22.003604	25.144722	Egypt	RMCA	gerbillus		
37802	22.003604	25.144722	Egypt	RMCA	gerbillus		
37804	22.01231	25.1577	Egypt	RMCA	gerbillus		
37806	22.01231	25.1577	Egypt	RMCA	gerbillus		
37808	22.038234	25.201083	Egypt	RMCA	gerbillus		
37810	22.038234	25.201083	Egypt	RMCA	gerbillus		
37815	22.025772	25.179592	Egypt	RMCA	gerbillus		
91-090-M-215	36.44	10.58	Tunisia	RMCA	pyramidum		
91-090-M-216	37.0001	10.1666	Tunisia	RMCA	pyramidum		
91-090-M-217	33.16	10.33	Tunisia	RMCA	gerbillus		
91-090-M-228	30.5	9.25	Tunisia	RMCA	gerbillus		
91-090-M-229	37	10.166667	Tunisia	RMCA	pyramidum		
91-090-M-256	37.083	9.85	Tunisia	RMCA	campestris		
91-090-M-257	37	10.166667	Tunisia	RMCA	gerbillus		
91-090-M-259	34.25	9.233333	Tunisia	RMCA	amoenus		
91-090-M-262	33.883333	10.116667	Tunisia	RMCA	campestris		
91-090-M-263	33.466667	8.716667	Tunisia	RMCA	simoni		
91-090-M-289	32.5	20.83	Libya	RMCA	amoenus		
8247	21.766667	31.283333	Sudan	NHM	gerbillus		
8250	22.12	31.32	Sudan	NHM	pyramidum		
12064	24.2	23.1	Libya	NHM	gerbillus		
25748	23.3	5.684	Algeria	NHM	campestris		
25750	25.9	13.85	Libya	NHM	pyramidum		
25751	25.9	13.85	Libya	NHM	pyramidum		
25759	24.911	17.759	Libya	NHM	gerbillus		
27347	32.817	21.517	Libya	NHM	campestris		
28086	31.15	-7.45	Morocco	NHM	campestris		
28088	32.54	-9.27	Morocco	NHM	campestris		
28090	33.8	-7.166667	Morocco	NHM	campestris		
30219	32.766667	21.333333	Libya	NHM	gerbillus		
30221	30.45	18.483333	Libya	NHM	gerbillus		
30255	32.767	21.334	Libya	NHM	campestris		
30262	32.684	21.6	Libya	NHM	campestris		
M-ANE1	17.997833	0.460533	Mali	CBGP	tarabuli	1	alignment.fasta
M-ANE2	17.997833	0.460533	Mali	CBGP	tarabuli		

M-AZA13	17.863067	-1.830683	Mali	CBGP	tarabuli	1	alignment.fasta
M-AZA14	17.863067	-1.830683	Mali	CBGP	tarabuli	1	alignment.fasta
M-CHA5	19.721183	-0.028667	Mali	CBGP	pyramidum	1	alignment.fasta
M-INA10	19.355683	-0.241783	Mali	CBGP	gerbillus		
M-INA16	19.355683	-0.241783	Mali	CBGP	tarabuli	1	alignment.fasta
M-INA17	19.355683	-0.241783	Mali	CBGP	gerbillus		
M-INA18	19.355683	-0.241783	Mali	CBGP	tarabuli		
M-INA19	19.333333	-0.241667	Mali	CBGP	pyramidum	1	alignment.fasta
M-INA2	19.337817	-0.240333	Mali	CBGP	gerbillus	1	alignment.fasta
M-INA20	19.333333	-0.241667	Mali	CBGP	pyramidum		
M-INA22	19.333333	-0.241667	Mali	CBGP	pyramidum	1	alignment.fasta
M-INA8	19.330483	-0.233617	Mali	CBGP	gerbillus		
M-TAD10	17.373017	-2.80455	Mali	CBGP	tarabuli	1	alignment.fasta
M-TAD3	17.373017	-2.80455	Mali	CBGP	tarabuli		
M-TAD7	17.373017	-2.80455	Mali	CBGP	tarabuli	1	alignment.fasta
M-TAD8	17.373017	-2.80455	Mali	CBGP	tarabuli	1	alignment.fasta
M-TES11	20.015233	0.9403	Mali	CBGP	amoenus	1	alignment.fasta
M-TES19	20.015233	0.9403	Mali	CBGP	pyramidum		
M-TES23	20.256133	0.989633	Mali	CBGP	amoenus	1	alignment.fasta
M-TES28	20.194233	0.97225	Mali	CBGP	pyramidum	1	alignment.fasta
M-TES30	20.187633	0.975417	Mali	CBGP	pyramidum	1	alignment.fasta
M-TOU1	17.82325	-3.197433	Mali	CBGP	gerbillus	1	alignment.fasta
M-TOU11	17.82325	-3.197433	Mali	CBGP	gerbillus	1	alignment.fasta
M-TOU2	17.82325	-3.197433	Mali	CBGP	gerbillus		
M-TOU3	17.82325	-3.197433	Mali	CBGP	gerbillus	1	alignment.fasta
M-TOU6	17.82325	-3.197433	Mali	CBGP	gerbillus		
M-TOU7	17.82325	-3.197433	Mali	CBGP	gerbillus	1	alignment.fasta
M5185	17.227167	0.241	Mali	CBGP	pyramidum		
M5309	17.0264	2.117333	Mali	CBGP	amoenus	1	alignment.fasta
M5314	19.019267	1.799133	Mali	CBGP	tarabuli	1	alignment.fasta
M5383	15.122717	2.089067	Mali	CBGP	tarabuli		
M5925	19.709717	0.0034	Mali	CBGP	amoenus	1	alignment.fasta
M5927	20.187633	0.97225	Mali	CBGP	pyramidum	1	alignment.fasta
M5928	19.330483	-0.233617	Mali	CBGP	gerbillus	1	KM236125
M5929	17.863067	-1.830683	Mali	CBGP	tarabuli	1	alignment.fasta
M5930	19.330483	-0.233617	Mali	CBGP	tarabuli	1	alignment.fasta
M5931	19.721183	-0.028667	Mali	CBGP	pyramidum	1	alignment.fasta
M5932	17.82325	-3.197433	Mali	CBGP	tarabuli	1	alignment.fasta
M5933	19.333333	-0.241667	Mali	CBGP	pyramidum	1	alignment.fasta
M5934	19.330483	-0.233617	Mali	CBGP	gerbillus	1	alignment.fasta
M5935	17.373017	-2.80455	Mali	CBGP	tarabuli	1	alignment.fasta
M5936	20.187633	0.975417	Mali	CBGP	pyramidum	1	alignment.fasta
M5937	19.7279	0.191183	Mali	CBGP	pyramidum	1	alignment.fasta
M5938	17.82325	-3.197433	Mali	CBGP	gerbillus	1	alignment.fasta

M5939	19.333333	-0.241667	Mali	CBGP	gerbillus	1	alignment.fasta
M5942	20.015233	0.9403	Mali	CBGP	pyramidum	1	alignment.fasta
M5943	17.373017	-2.80455	Mali	CBGP	tarabuli	1	alignment.fasta
M5944	17.863067	-1.830683	Mali	CBGP	tarabuli	1	alignment.fasta
M5945	17.997833	0.460533	Mali	CBGP	tarabuli	1	alignment.fasta
M5946	20.194233	0.97225	Mali	CBGP	pyramidum	1	alignment.fasta
M5947	19.330483	-0.233617	Mali	CBGP	gerbillus	1	alignment.fasta
M5948	19.783567	0.328067	Mali	CBGP	tarabuli	1	alignment.fasta
M5949	20.187633	0.975417	Mali	CBGP	tarabuli	1	alignment.fasta
M5950	17.373017	-2.80455	Mali	CBGP	tarabuli	1	alignment.fasta
M5951	17.82325	-3.197433	Mali	CBGP	gerbillus	1	alignment.fasta
M5952	20.015233	0.9403	Mali	CBGP	pyramidum	1	alignment.fasta
M5953	17.82325	-3.197433	Mali	CBGP	gerbillus	1	alignment.fasta
M5957	19.709717	0.0034	Mali	CBGP	gerbillus	1	alignment.fasta
M5958	17.82325	-3.197433	Mali	CBGP	gerbillus	1	alignment.fasta
M5960	19.722783	0.1816	Mali	CBGP	amoenus	1	alignment.fasta
M5961	17.373017	-2.80455	Mali	CBGP	tarabuli	1	alignment.fasta
M5962	19.7116	0.1873	Mali	CBGP	tarabuli	1	alignment.fasta
M5963	20.194233	0.97225	Mali	CBGP	pyramidum	1	alignment.fasta
M5964	20.015233	0.9403	Mali	CBGP	pyramidum	1	alignment.fasta
M5965	20.015233	0.9403	Mali	CBGP	amoenus	1	alignment.fasta
M5966	20.191667	1	Mali	CBGP	amoenus	1	alignment.fasta
M5967	19.337817	-0.240333	Mali	CBGP	tarabuli	1	alignment.fasta
M5968	19.72945	-0.0246	Mali	CBGP	pyramidum	1	alignment.fasta
M5969	17.373017	-2.80455	Mali	CBGP	tarabuli	1	alignment.fasta
M5970	19.716	-0.0211	Mali	CBGP	gerbillus	1	alignment.fasta
M5971	19.355683	-0.241783	Mali	CBGP	tarabuli	1	alignment.fasta
M5972	19.721183	-0.028667	Mali	CBGP	nigeriae	1	alignment.fasta
M5973	20.015233	0.9403	Mali	CBGP	amoenus	1	alignment.fasta
M5974	20.187633	0.975417	Mali	CBGP	pyramidum		
M5975	20.191667	1	Mali	CBGP	pyramidum	1	alignment.fasta
M5976	19.7462	-0.034017	Mali	CBGP	gerbillus	1	alignment.fasta
M5977	19.355683	-0.241783	Mali	CBGP	tarabuli	1	alignment.fasta
M5978	19.337817	-0.240333	Mali	CBGP	pyramidum	1	alignment.fasta
M5979	19.355683	-0.241783	Mali	CBGP	tarabuli	1	alignment.fasta
M5980	20.191667	1	Mali	CBGP	amoenus	1	alignment.fasta
M5982	19.72945	-0.0246	Mali	CBGP	pyramidum	1	JN652809
M5983	17.373017	-2.80455	Mali	CBGP	tarabuli	1	alignment.fasta
M5984	17.863067	-1.830683	Mali	CBGP	tarabuli	1	alignment.fasta
M5985	19.355683	-0.241783	Mali	CBGP	gerbillus	1	alignment.fasta
M5986	17.373017	-2.80455	Mali	CBGP	tarabuli		
M6108	18.030317	0.47515	Senegal	CBGP	pyramidum	1	alignment.fasta
M6133	19.412817	-0.57765	Senegal	CBGP	pyramidum	1	alignment.fasta
M6134	18.030317	0.47515	Senegal	CBGP	tarabuli	1	alignment.fasta

M6135	19.412817	-0.57765	Senegal	CBGP	tarabuli	1	alignment.fasta
MAR-08-LG102	28.841467	-10.84575	Morocco	CBGP	occiduus	1	alignment.fasta
MAR-08-LG103	28.841467	-10.84575	Morocco	CBGP	occiduus	1	JN021421
MAR-08-LG104	28.8324	-10.83505	Morocco	CBGP	occiduus	1	JN021422
MAR-08-LG106	28.839983	-10.836483	Morocco	CBGP	occiduus	1	JN021424
MAR-08-LG107	28.839983	-10.836483	Morocco	CBGP	occiduus	1	JN021423
MAR-08-LG108	28.837367	-10.840683	Morocco	CBGP	occiduus	1	JN021425
MAR-08-LG109	28.837367	-10.840683	Morocco	CBGP	occiduus	1	JN021426
MAR-08-LG110	28.481617	-11.237467	Morocco	CBGP	occiduus	1	JN021432
MAR-08-LG112	27.956017	-12.760717	Morocco	CBGP	gerbillus	1	JN021403
MAR-08-LG114	27.956017	-12.760717	Morocco	CBGP	occiduus	1	JN021435
MAR-08-LG115	27.956017	-12.760717	Morocco	CBGP	occiduus	1	JN021440
MAR-08-LG117	27.958933	-12.832867	Morocco	CBGP	occiduus	1	JN021437
MAR-08-LG118	27.958933	-12.832867	Morocco	CBGP	occiduus		
MAR-08-LG120	27.96495	-12.778783	Morocco	CBGP	gerbillus	1	JN021405
MAR-08-LG122	27.96495	-12.778783	Morocco	CBGP	gerbillus	1	JN021407
MAR-08-LG124	27.96495	-12.778783	Morocco	CBGP	gerbillus	1	JN021409
MAR-08-LG126	27.96495	-12.778783	Morocco	CBGP	gerbillus	1	JN021411
MAR-08-LG127	23.918067	-15.763333	Morocco	CBGP	occiduus		
MAR-08-LG128	23.918067	-15.763333	Morocco	CBGP	occiduus		
MAR-08-LG129	23.918067	-15.763333	Morocco	CBGP	occiduus	1	JN021427
MAR-08-LG130	23.918067	-15.763333	Morocco	CBGP	occiduus		
MAR-08-LG131	23.918067	-15.763333	Morocco	CBGP	occiduus	1	JN021428
MAR-08-LG132	23.885067	-15.830083	Morocco	CBGP	occiduus	1	JN021429
MAR-08-LG133	23.885067	-15.830083	Morocco	CBGP	occiduus	1	JN021433
MAR-08-LG134	23.885067	-15.830083	Morocco	CBGP	occiduus	1	JN021434
MAR-08-LG135	23.885067	-15.830083	Morocco	CBGP	occiduus		
MAR-08-LG136	23.885067	-15.830083	Morocco	CBGP	occiduus		
MAR-08-LG137	23.885067	-15.830083	Morocco	CBGP	occiduus	1	alignment.fasta
MAR-08-LG138	23.885067	-15.830083	Morocco	CBGP	occiduus	1	JN021439
MAR-08-LG139	23.885067	-15.830083	Morocco	CBGP	occiduus	1	JN021430
MAR-08-LG140	23.885067	-15.830083	Morocco	CBGP	occiduus	1	JN021431
MAR-08-LG65	30.3637	-8.9254	Morocco	CBGP	hoogstrali	1	JN021412
MAR-08-LG66	30.3637	-8.9254	Morocco	CBGP	hoogstrali	1	JN021413
MAR-08-LG67	30.3637	-8.9254	Morocco	CBGP	hoogstrali	1	JN021417
MAR-08-LG72	30.3637	-8.9254	Morocco	CBGP	hoogstrali	1	JN021414
MAR-08-LG73	30.3637	-8.9254	Morocco	CBGP	hoogstrali	1	JN021419
MAR-08-LG75	30.3637	-8.9254	Morocco	CBGP	hoogstrali	1	JN021420
MAR-08-LG77	30.066667	-9.658333	Morocco	CBGP	sp	1	JN021441
MAR-08-LG79	30.066667	-9.658333	Morocco	CBGP	sp	1	JN021446
MAR-08-LG91	29.821467	-9.8232	Morocco	CBGP	sp	1	JN021448
MAR-08-LG93	29.821467	-9.8232	Morocco	CBGP	sp	1	JN021442
MAR-08-LG96	29.821467	-9.8232	Morocco	CBGP	sp	1	JN021451
N3008	19.166667	7.966667	Niger	CBGP	pyramidum	1	alignment.fasta

N3013	17.45	6.7	Niger	CBGP	tarabuli	1	alignment.fasta
N3015	17.416667	6.783333	Niger	CBGP	pyramidum	1	alignment.fasta
N3016	17.45	6.7	Niger	CBGP	tarabuli	1	alignment.fasta
N3019	17.45	6.7	Niger	CBGP	tarabuli		
N3029	17.416667	6.783333	Niger	CBGP	tarabuli	1	alignment.fasta
N3035	18.55	7.783333	Niger	CBGP	gerbillus	1	alignment.fasta
N3036	17.45	6.7	Niger	CBGP	pyramidum	1	alignment.fasta
N3038	18.55	7.783333	Niger	CBGP	pyramidum		
N3039	18.933333	8.25	Niger	CBGP	pyramidum	1	alignment.fasta
N3040	17.083333	7.45	Niger	CBGP	tarabuli	1	alignment.fasta
N3073	13.467417	14.7127	Chad	CBGP	pyramidum	1	alignment.fasta
N3133	14.9	12.516667	Niger	CBGP	tarabuli	1	alignment.fasta
N3134	14.9	12.516667	Niger	CBGP	tarabuli	1	alignment.fasta
N3139	14.183333	13.183333	Niger	CBGP	tarabuli	1	alignment.fasta
N3148	15.016667	12.466667	Niger	CBGP	tarabuli		
N3158	15.016667	12.466667	Niger	CBGP	tarabuli	1	alignment.fasta
N3168	13.683333	13.3	Niger	CBGP	amoenus	1	alignment.fasta
N3180	14.25	13.15	Niger	CBGP	tarabuli	1	alignment.fasta
N3195	14.9	12.516667	Niger	CBGP	tarabuli	1	alignment.fasta
N3196	14.9	12.516667	Niger	CBGP	tarabuli		
N3197	14.9	12.516667	Niger	CBGP	tarabuli		
N3217	18.116667	11.583333	Niger	CBGP	pyramidum		
N3221	18.116667	11.583333	Niger	CBGP	pyramidum		
N3223	18.116667	11.583333	Niger	CBGP	pyramidum	1	alignment.fasta
N3307	14.266667	0.733333	Niger	CBGP	henleyi		
N3312	18.683333	12.916667	Niger	CBGP	amoenus	1	alignment.fasta
N3313	18.68333	12.916667	Niger	CBGP	amoenus	1	alignment.fasta
N3324	15.06666	10.7	Niger	CBGP	tarabuli		
N3327	15.63333	11.516667	Niger	CBGP	pyramidum	1	alignment.fasta
N3329	15.63333	11.516667	Niger	CBGP	pyramidum	1	alignment.fasta

Alignment.fasta. Sequences used for barcodding specimens.

>ZBSC0055

--AACCACTCATTCATTGAC

CTCCCCACTCCCCCCAACATTTCATCCTGATGAAACTTTGGCTCACTCTTAGGAATTTGC

CTAGTAATTCAAATCGCCACAGGACTCTTTTTAGCCATACATTACACAGCAGATACAACA

ACAGCATTTTCATCAGTATCTCACATCTGCCGAGACGTAAATTACGGATGACTAATCCGT

TATATACATGCAAACGGAGCCTCAATATTCTTTATCTGCTTATTCATCCACATTGGACGA

GGAATCTACTATGGATCCTACATCTTCCACGAAACATGAAATATCGGAGTAATTCTCTTA

TTCGCCGTAATAGCCACTGCATTCATAGGATATGTCCTACCATGAGGACAGATATCTTTC

TGAGGGGCCACAGTCATTACAAATCTCCTCTCAGCAATCCCACACATTGGCCCAACAATC

GTAGAATGAATTTGAGGAGGCTTCTCAGTAGACAAAGCCACTTTAACACGATTCTTTGCA

TTCCATTTCATCCTTCCCTTTATCATCACAGCTCTTGTCCTAGTCCATTTATTATTTCTC

CACGAAACCGGATCCAACAATCCCCTAGGAATCAACTCTAACGCTGACAAAATCCCCTTC

CACCCTTACTATACAGTAAAAGATTACCTAGGAGTAATTTTACTACTC------------

>ZBSC0058

--AATCACTCATTTATTGAT

CTTCCCACTCCCCCTAACATCTCATCCTGATGAAACTTCGGCTCACTATTAGGAATCTGC

CTAATTATCCAAATCGCCACAGGACTTTTCCTAGCTATACATTATACAGCAGACACAATA

ACAGCATTTTCATCAGTATCCCATATCTGCCGAGATGTAAATTACGGATGATTAATTCGC

TATATACATGCCAACGGAGCCTCGATATTCTTTATCTGTCTATTTATTCACATCGGACGA

GGCATCTACTATGGATCTTATATCTTCCAAGAAACATGGAACATCGGAGTAATCTTATTA

TTCGCCGTTATAGCTACCGCATTCATAGGATATGTCCTACCATGAGGACAAATATCTTTT

TGGGGGGCCACAGTAATTACAAACCTCCTCTCAGCAATTCCATACATTGGCCCAACAATC

GTAGAATGAATCTGAGGAGGCTTCTCAGTAGACAAAGCCACCCTAACACGATTTTTTGCA

TTTCATTTTATTCTCCCTTTTATCATTACAGCTCTAGTCCTAGTTCACCTATTATTCCTC

CACGAAACCGGATCCAACAATCCTTTAGGCATCAACTCCAACGCAGATAAAATTCCATTT

CACCCTTACTATACAGTTAAAGATTTCTTAGGGGTTATCCTACTCCTC------------

>ZBSC0059

--AACCACTCATTCATCGAC

CTCCCCACTCCTCCAAACATCTCATCCTGATGAAACTTTGGCTCACTTCTAGGAATTTGC

TTAGTAATTCAAATTGCTACAGGACTTTTCCTAGCCATACATTATACAGCAGACACAACA

ACAGCATTTTCATCAGTATCCCATATCTGCCGAGACGTAAATTACGGATGACTAATCCGT

TATATACACGCAAACGGAGCCTCACTATTCTTCATCTGCCTATTTATCCATATCGGACGA

GGCATTTACTACGGATCCTACATCTTCCAAGAAACATGAAACATCGGTGTAATCCTCCTA

TTTGCCGTAATAGCTACCGCATTCATAGGGTACGTCCTACCATGAGGACAAATATCCTTC

TGAGGAGCCACAGTGATTACAAATCTCCTCTCAGCAATCCCATATATTGGCCCAACAATT

GTAGAATGAATCTGAGGAGGGTTCTCAGTAGACAAAGCCACCCTAACACGATTTTTCGCA

TTCCACTTTATCCTCCCTTTTATTATTACAGCCCTTGTCCTAGTCCACCTCCTATTTCTT

CACGAAACCGGATCCAATAACCCCCTAGGACTAAACTCTAATGCAGACAAAATCCCCTTT

CACCCCTATTACACAGTAAAAGATTTTCTCGGAGTAATTTTACTACTT------------

>ZBSC0060

---AACCACTCATTCATTGAC

CTCCCCACACCCCCCAATATTTCATCCTGATGAAACTTTGGCTCACTCCTAGGAATTTGT

TTAATAATTCAAATTACTACAGGACTCTTTCTAGCCATACACTACACAGCAGACACAACA

ACAGCATTTTCATCAGTATCTCACATCTGCCGAGATGTAAACTACGGATGACTAATCCGT

TATATACACGCAAACGGAGCCTCAATATTTTTTATTTGCTTATTCATTCACATTGGACGA

GGTATCTACTATGGATCATACATTTTTCAAGAAACATGAAACATCGGTGTAATTCTCCTA

TTCGCTGTGATAGCCACTGCATTCATAGGATATGTTCTACCATGAGGACAAATATCCTTC

TGAGGAGCCACAGTCATCACAAACCTCCTTTCAGCAATTCCATATATTGGCCCAACAATC

GTAGAATGAATCTGAGGAGGCTTCTCAGTAGATAAAGCCACTCTAACACGATTCTTCGCA

TTCCACTTTATTCTTCCCTTTATTATCACAGCCCTTGTCCTAGTCCATCTCTTATTCCTC

CACGAAACTGGATCTAACAATCCCCTAGGACTTAACTCCAACGCAGATAAAATTCCCTTC

CACCCATACTACACAGTAAAAGATTTCCTCGGAGTAATCTTACTACTT------------

>ZBSC0061

---AACCACTCATTCATCGAC

CTCCCCACTCCTCCAAACATCTCATCCTGATGAAACTTTGGCTCACTTCTAGGAATTTGC

TTAGTAATTCAAATTGCTACAGGACTTTTCCTAGCCATACATTATACAGCAGACACAACA

ACAGCATTTTCATCAGTATCCCATATCTGCCGAGACGTAAATTACGGATGACTAATCCGT

TATATACATGCAAACGGAGCCTCACTATTCTTCATCTGCCTATTTATCCATATCGGACGA

GGCATTTACTACGGATCCTACATCTTCCAAGAAACATGAAACATCGGTGTAATCCTCCTA

TTTGCCGTAATAGCTACCGCATTCATAGGGTACGTCCTACCATGAGGACAAATATCCTTC

TGAGGAGCCACAGTGATTACAAATCTCCTCTCAGCAATCCCATATATTGGCCCAACAATT

GTAGAATGAATCTGAGGGGGATTCTCAGTAGACAAAGCCACCCTAACACGATTTTTCGCA

TTCCACTTTATCCTCCCTTTTATTATTACAGCCCTTGTCCTAGTCCACCTCCTATTTCTT

CACGAAACCGGATCCAATAACCCCCTAGGACTAAACTCTAATGCAGACAAAATCCCCTTT

CACCCCTATTACACAGTAAAAGATTTTCTCGGAGTAATTTTACTACTT------------

>ZBSC0062

---AACCACTCATTCATCGAC

CTCCCCACTCCTCCAAACATCTCATCCTGATGAAACTTTGGCTCACTTCTAGGAATTTGC

TTAGTAATTCAAATTGCTACAGGACTTTTCCTAGCCATACATTATACAGCAGACACAACA

ACAGCATTTTCATCAGTATCCCATATCTGCCGAGACGTAAATTACGGATGACTAATCCGT

TATATACACGCAAACGGAGCCTCACTATTCTTCATCTGCCTATTTATCCATATTGGACGA

GGCATTTACTACGGATCCTACATCTTCCAAGAAACATGAAACATCGGTGTAATCCTCCTA

TTTGCCGTAATAGCTACCGCATTCATAGGGTACGTCCTACCATGAGGACAAATATCCTTC

TGAGGAGCCACAGTGATTACAAATCTCCTCTCAGCAATCCCATATATTGGCCCAACAATT

GTAGAATGAATCTGAGGGGGGTTCTCAGTAGACAAAGCCACCCTAACACGATTTTTCGCA

TTCCACTTTATCCTCCCTTTTATTATTACAGCCCTTGTCCTAGTCCACCTCCTATTTCTT

CACGAAACCGGATCCAATAACCCCCTAGGACTAAACTCTAATGCAGACAAAATCCCCTTT

CACCCCTATTACACAGTAAAAGATTTTCTCGGAGTAATTTTACTACTT------------

>ZBSC0067

--AACCACTCATTTATTGAT

CTCCCTACTCCCCCTAACATTTCATCTTGATGAAACTTCGGCTCACTTCTAGGAATCTGC

CTAATAATCCAAATTGCCACAGGATTATTCCTAGCCATACATTACACAGCAGACACAACA

ACAGCATTCTCATCAGTATCACATATTTGCCGAGACGTAAATTACGGATGACTTATCCGC

TACATACACGCCAACGGAGCCTCAATATTTTTTATTTGCTTATTTATTCACATCGGACGA

GGAATTTACTATGGATCCTACATCTTCCAAGAAACATGAAATATCGGTATCATCCTTCTT

TTCGCTGTAATAGCCACCGCATTCATAGGCTATGTACTTCCATGAGGACAGATGTCCTTC

TGAGGAGCTACAGTAATCACTAACCTCCTCTCAGCAATCCCATACATTGGTCCAACAATC

GTAGAGTGAATCTGAGGAGGATTCTCAGTAGACAAAGCCACCCTAACACGATTCTTCGCA

TTCCATTTCATCCTCCCATTCATCATTGCAGCCCTTGTACTTGTCCATCTCTTGTTTCTT

CACGAGACTGGATCTAACAATCCCCTGGGAATCAACTCAAATGCAGACAAAATCCCTTTC

CACCCCTATTACACAGTAAAAGACTTTCTAGGGGTTATTATCCTCATC------------

>ZBSC0068

---AACCACTCATTCATCGAC

CTCCCCACTCCTCCAAACATCTCATCCTGATGAAACTTTGGCTCACTTCTAGGAATTTGC

TTAGTAATTCAAATTGCTACAGGACTTTTCCTAGCCATACATTATACAGCAGATACAACA

ACAGCATTTTCATCAGTATCCCATATCTGCCGAGACGTAAATTACGGATGACTAATCCGT

TATATACACGCAAACGGAGCCTCACTATTCTTCATCTGCCTATTTATCCATATCGGACGA

GGCATTTACTACGGATCCTACATCTTCCAAGAAACATGAAACATCGGTGTAATCCTCCTA

TTTGCCGTAATAGCTACCGCATTCATAGGGTACGTCCTACCATGAGGACAAATATCCTTC

TGAGGAGCCACAGTGATTACAAATCTCCTCTCAGCAATCCCATATATTGGCCCAACAATT

GTAGAATGAATCTGAGGGGGGTTCTCAGTAGACAAAGCCACCCTAACACGATTTTTCGCA

TTCCACTTTATCCTCCCTTTTATTATTACAGCCCTTGTCCTAGTCCACCTCTTATTTCTT

CACGAAACCGGATCCAATAACCCCCTAGGACTAAACTCTAATGCAGACAAAATCCCCTTT

CACCCCTATTACACAGTAAAAGATTTTCTCGGAGTAATTTTACTACTT------------

>ZBSC0071

--AACCACTCATTCATTGAT

CTTCCCACTCCCCCTAATATTTCATCTTGATGAAACTTTGGCTCACTCCTAGGAATTTGC

TTAATAATTCAAATCGCTACAGGACTTTTCCTAGCCATACATTATACAGCAGACACAACA

ACAGCATTCTCATCAGTATCCCATATCTGCCGAGACGTCAATTATGGATGACTAATCCGC

TATATACATGCAAACGGAGCTTCAATATTCTTTATTTGCCTATTCATTCACATCGGACGA

GGAATTTACTACGGATCTTATATCTTTCAAGAAACATGAAACATTGGGGTAATTCTCTTA

TTTGCCGTAATAGCCACCGCATTCATAGGGTATGTACTTCCATGAGGACAAATATCCTTC

TGAGGGGCCACAGTCATTACAAATCTTCTTTCAGCTATTCCATATATTGGCCCAACAATC

GTAGAATGAATTTGAGGAGGATTTTCAGTGGACAAAGCCACTTTAACACGATTTTTCGCA

TTTCACTTCATTCTCCCCTTTATTATCACAGCCTTAGTCCTAGTCCATCTCCTATTCCTT

CACGAAACCGGATCTAATAATCCCCTAGGCCTTAACTCCAACTCAGACAAAATCCCTTTT

CACCCATACTACACAGTAAAAGATTTTCTCGGAGTAATTCTACTACTT------------

>ZBSC0073

--AACCACTCATTCATTGAT

CTTCCCACTCCCCCTAATATTTCATCTTGATGAAACTTTGGCTCACTCCTAGGAATTTGC

TTAATAATTCAAATCGCTACAGGACTTTTCCTAGCCATACATTATACAGCAGACACAACA

ACAGCATTCTCATCAGTATCCCATATCTGCCGAGACGTCAATTATGGATGACTAATCCGC

TATATACATGCAAACGGAGCTTCAATATTCTTTATTTGCCTATTCATTCACATCGGACGA

GGAATTTACTACGGATCTTATATCTTTCAAGAAACATGAAACATTGGAGTAATTCTCTTA

TTTGCCGTAATAGCCACCGCATTCATAGGGTATGTACTTCCATGAGGACAAATATCCTTC

TGAGGGGCCACAGTCATTACAAATCTTCTTTCAGCTATTCCATATATTGGCCCAACAATC

GTAGAATGAATTTGAGGAGGATTTTCAGTGGACAAAGCCACTTTAACACGATTTTTCGCA

TTTCACTTCATTCTCCCCTTTATTATCACAGCCTTAGTCCTAGTCCATCTCCTATTCCTT

CACGAAACCGGATCTAATAATCCCCTAGGCCTTAACTCCAACTCAGACAAAATCCCTTTT

CACCCATACTACACAGTAAAAGATTTTCTCGGAGTAATTCTACTACTC------------

>ZBSC0200

--AACCACTCATTCATTGAT

CTTCCCACTCCCCCTAATATTTCATCTTGATGAAACTTTGGCTCACTCCTAGGAATTTGC
TTAATAATTCAAATCGCTACAGGACTTTTCCTAGCTATACATTATACAGCAGACACAACA
ACAGCATTCTCATCAGTATCTCATATCTGCCGAGACGTCAATTATGGATGACTAATCCGC
TATATACATGCAAACGGAGCTTCAATATTCTTTATTTGCCTATTCATTCACATCGGACGA
GGAATTTACTACGGATCTTATATCTTTCAAGAAACATGAAACATTGGAGTAATTCTCTTA
TTTGCCGTAATAGCCACCGCATTCATAGGGTATGTACTTCCATGAGGACAAATATCCTTC
TGAGGGGCCACAGTCATTACAAATCTTCTTTCAGCTATTCCATATATTGGCCCAACAATC
GTAGAATGAATTTGAGGAGGATTTTCAGTGGACAAAGCCACTTTAACACGATTTTTCGCA
TTTCACTTCATTCTCCCCTTTATTATCACAGCCTTAGTCCTAGTCCATCTCCTATTCCTT
CACGAAACCGGATCTAATAATCCCCTAGGCCTTAACTCCAACTCAGACAAAATCCCTTTT
CACCCATACTACACAGTAAAAGATTTCCTCGGAGTAATTCTACTACTT------------

>ZBSC0201
--AACCACTCATTTATTGAT
CTTCCCACTCCCCCTAATATTTCATCTTGATGAAACTTTGGCTCACTCCTAGGAATTTGC
TTAATAATTCAAATCGCTACAGGACTTTTCCTGGCCATACATTATACAGCAGACACAACA
ACAGCATTCTCATCAGTATCCCATATCTGCCGAGACGTCAATTATGGATGACTAATCCGC
TATATACATGCAAACGGAGCTTCAATATTCTTTATTTGCCTATTCATTCACATCGGACGA
GGAATTTACTACGGATCTTATATCTTTCAAGAAACATGAAACATTGGAGTAATTCTCTTA
TTTGCCGTAATAGCCACCGCATTCATAGGGTATGTACTTCCATGAGGACAAATATCCTTC
TGAGGGGCTACAGTCATTACAAATCTTCTTTCAGCTATTCCATATATTGGCCCAACAATC
GTAGAATGAATTTGAGGAGGATTTTCAGTGGACAAAGCCACTTTAACACGATTTTTCGCA
TTTCACTTCATTCTCCCCTTTATTATCACAGCCTTAGTCCTAGTCCATCTCCTATTCCTT
CACGAAACCGGATCTAATAATCCCCTAGGCCTTAACTCCAACTCAGACAAAATCCCTTTT
CACCCATACTACACAGTAAAAGATTTTCTCGGAGTAATTCTACTACTC------------

>ZBSC0202
--AACCACTCATTCATTGAT
CTTCCCACTCCCCCTAATATTTCATCTTGATGAAACTTTGGCTCACTCCTAGGAATTTGC
TTAATAATTCAAATCGCTACAGGACTTTTCCTAGCCATACATTATACAGCAGACACAACA
ACAGCATTCTCATCAGTATCCCATATCTGCCGAGACGTCAATTATGGATGACTAATCCGC
TATATACATGCAAACGGAGCTTCAATATTCTTTATTTGCCTATTCATTCACATCGGACGA
GGAATTTACTACGGATCTTATATCTTTCAAGAAACATGAAACATTGGAGTAATTCTCTTA
TTTGCCGTAATAGCCACCGCATTCATAGGGTATGTACTTCCATGAGGACAAATATCCTTC
TGAGGGGCTACAGTCATTACAAATCTTCTTTCAGCTATTCCATATATTGGCCCAACAATC
GTAGAATGAATTTGAGGAGGATTTTCAGTGGACAAAGCCACTTTAACACGATTTTTCGCA
TTTCACTTCATTCTCCCCTTTATTATCACAGCCTTAGTCCTAGTCCATCTCCTATTCCTT
CACGAAACCGGATCTAATAATCCCCTAGGCCTTAACTCCAACTCAGACAAAATCCCTTTT
CACCCATACTACACAGTAAAAGATTTTCTCGGAGTAATTCTACTACTC------------

>ZBSC0203
--AACCACTCATTCATTGAT
CTTCCCACTCCCCCTAATATTTCATCTTGATGAAACTTTGGCTCACTCCTAGGAATTTGC
TTAATAATTCAAATCGCTACAGGACTTTTCCTAGCCATACATTATACAGCAGACACAACA
ACAGCATTCTCATCAGTATCCCATATCTGCCGAGACGTCAATTATGGATGACTAATCCGC
TATATACATGCAAACGGAGCTTCAATATTCTTTATTTGCCTATTCATTCACATCGGACGA
GGAATTTACTACGGATCTTATATCTTTCAAGAAACATGAAACATTGGGGTAATTCTCTTA
TTTGCCGTAATAGCCACCGCATTCATAGGGTATGTACTTCCATGAGGACAAATATCCTTC
TGAGGGGCCACAGTCATTACAAATCTTCTTTCAGCTATTCCATATATTGGCCCAACAATC
GTAGAATGAATTTGAGGAGGATTTTCAGTGGACAAAGCCACTTTAACACGATTTTTCGCA
TTTCACTTCATTCTCCCCTTTATTATCACAGCCTTAGTCCTAGTCCATCTCCTATTCCTT
CACGAAACCGGATCTAATAATCCCCTAGGCCTTAACTCCAACTCAGACAAAATCCCTTTT
CACCCATACTACACAGTAAAAGATTTTCTCGGAGTAATTCTACTACTC------------

>ZBSC0204
--AACCACTCATTCATCGAC
CTCCCCACTCCTCCAAACATCTCATCCTGATGAAACTTTGGCTCACTTCTAGGAATTTGC
TTAGTAATTCAAATTGCTACAGGACTTTTCCTAGCCATACATTATACAGCAGACACAACA
ACAGCATTTTCATCAGTATCCCATATCTGCCGAGACGTAAATTACGGATGACTAATCCGT
TATATACACGCAAACGGAGCCTCACTATTCTTCATCTGTCTATTCATCCATATCGGACGA
GGCATTTACTACGGATCCTACATCTTCCAAGAAACATGAAACATCGGTGTAATCCTCCTA
TTTGCCGTAATAGCTACCGCATTCATAGGATACGTCCTACCATGAGGACAAATATCCTTC
TGAGGAGCCACAGTGATTACAAATCTCCTCTCAGCAATCCCGTATATTGGTCCAACAATT
GTAGAATGAATCTGAGGGGGGTTCTCAGTAGACAAAGCCACCCTAACACGATTTTTCGCA
TTCCATTTTATCCTCCCTTTTATTATTACAGCCCTTGTCCTAGTCCACCTCCTATTTCTT
CACGAAACCGGATCCAATAACCCCCTAGGACTAAACTCTAATGCAGACAAAATCCCCTTT
CACCCCTACTACACAGTAAAAGATTTTCTTGGAGTAATTTTACTACTT------------

>ZBSC0206
--AACCACTCATTCATTGAT
CTTCCCACTCCCCCTAATATCTCATCTTGATGAAACTTTGGTTCACTTCTAGGAATTTGC
TTAATTATTCAAATTGCTACAGGACTTTTCCTAGCCATACATTATACAGCAGACACAACA

ACAGCATTCTCATCAGTATCCCATATCTGCCGAGACGTCAATTATGGATGACTAATCCGC

TATATACATGCAAACGGAGCTTCAATATTCTTTATTTGCcTATTCATTCACATTGGACGA

GGAATTTACTACGGATCTTACATCTTTCAAGAAACATGAAACATTGGAGTAATTCTCTTA

TTTGCCGTAATAGCCACCGCATTCATAGGATATGTACTTCCATGAGGACAAATATCCTTC

TGAGGGGCCACAGTCATTACAAATCTTCTTTCAGCTATTCCATATATTGGCCCAACAATC

GTAGAATGAATTTGAGGAGGATTTTCAGTGGACAAAGCCACTTTAACACGATTTTTCGCA

TTCCACTTCATTCTCCCCTTTATTATCACAGCCCTAGTCCTAGTCCATCTTCTATTCCTT

CACGAAACCGGATCTAATAATCCCCTAGGCCTTAACTCCAACTCAGACAAAATCCCTTTT

CACCCATACTACACAGTAAAAGATTTTCTCGGAGTAATTCTACTACTT-----------

>ZBSC0208

--AACCACTCATTCATTGAT

CTTCCCACTCCCCCTAATATTTCATCTTGATGAAACTTTGGCTCACTCCTAGGAATTTGC

TTAATAATTCAAATCGCTACAGGACTTTTCCTAGCCATACATTATACAGCAGACACAACA

ACAGCATTCTCATCAGTATCCCATATCTGCCGAGACGTCAATTATGGATGACTAATCCGC

TATATACATGCAAACGGAGCTTCAATATTCTTTATTTGCCTATTCATTCACATCGGACGA

GGAATTTACTACGGATCTTATATCTTTCAAGAAACATGAAACATTGGAGTAATTCTCTTA

TTTGCCGTAATAGCCACCGCATTCATAGGGTATGTACTTCCATGAGGACAAATATCCTTC

TGAGGGGCCACAGTCATTACAAATCTTCTTTCAGCTATTCCATATATTGGCCCAACAATC

GTAGAATGAATTTGAGGAGGATTTTCAGTGGACAAAGCCACTTTAACACGATTTTTCGCA

TTTCACTTCATTCTCCCCTTTATTATCACAGCCTTAGTCCTAGTCCATCTCCTATTCCTT

CACGAAACCGGATCTAATAATCCCCTAGGCCTTAACTCCAACTCAGACAAAATCCCTTTT

CACCCATACTACACAGTAAAAGATTTTCTCGGAGTAATTCTACTACTT-----------

>ZBSC0209

--AACCACTCATTCATTGAT

CTTCCCACTCCCCCTAATATCTCATCTTGATGAAACTTTGGTTCACTTCTAGGAATTTGC

TTAATTATTCAAATTGCTACAGGACTTTTCCTAGCCATACATTATACAGCAGACACAACA

ACAGCATTCTCATCAGTATCCCATATCTGCCGAGACGTCAATTATGGATGACTAATCCGC

TATATACATGCAAACGGAGCTTCAATATTCTTTATTTGCCTATTCATTCACATTGGACGA

GGAATTTACTACGGATCTTACATCTTTCAAGAAACATGAAACATTGGAGTAATTCTCTTA

TTTGCCGTAATAGCCACCGCATTCATAGGATATGTACTTCCATGAGGACAAATATCCTTC

TGAGGGGCCACAGTCATTACAAATCTTCTTTCAGCTATTCCATATATTGGCCCAACAATC

GTAGAATGAATTTGAGGAGGATTTTCAGTGGACAAAGCCACTTTAACACGATTTTTCGCA

TTCCACTTCATTCTCCCCTTCATTATCACAGCCCTAGTCCTAGTCCATCTTCTATTCCTT

CACGAAACCGGATCTAATAATCCCCTAGGCCTTAACTCCAACTCAGACAAAATCCCTTTT

CACCCATACTACACAGTAAAAGACTTTCTCGGAGTAATTCTACTACTT-----------

>ZBSC0212

--AACCACTCATTCATTGAT

CTTCCCACTCCCCCTAATATCTCATCTTGATGAAACTTTGGTTCACTTCTAGGAATTTGC

TTAATCATTCAAATTGCTACAGGACTTTTCCTAGCCATACATTATACAGCAGACACAACA

ACAGCATTCTCATCAGTATCCCATATCTGCCGAGACGTCAATTATGGATGACTAATCCGC

TATATACATGCAAACGGAGCTTCAATATTCTTTATTTGCCTATTCATTCACATTGGACGA

GGAATTTACTACGGATCTTACATCTTTCAAGAAACATGAAACATTGGAGTAATTCTCTTA

TTTGCCGTAATAGCCACCGCATTCATAGGATATGTACTTCCATGAGGACAAATATCCTTC

TGAGGGGCCACAGTCATTACAAATCTTCTTTCAGCTATTCCATATATTGGCCCAACAATC

GTAGAATGAATTTGAGGAGGATTTTCAGTGGACAAAGCCACTTTAACACGATTTTTCGCA

TTCCACTTCATTCTCCCCTTCATTATCACAGCCCTAGTCCTAGTCCATCTTCTATTCCTT

CACGAAACCGGATCTAATAATCCCCTAGGCCTTAACTCCAACTCAGACAAAATCCCTTTT

CACCCATACTACACAGTAAAAGACTTTCTCGGAGTAATTCTACTACTT------------

>ZBSC0213

--AACCATTCATTCATTGAT

CTCCCCACTCCCCCCAACATCTCATCCTGATGAAACTTCGGCTCACTCCTAGGAATTTGC

TTAGTTATTCAAATCACCACAGGACTTTTCCTAGCTATACATTACACAGCAGACACAACA

ACAGCATTCTCATCAGTCTCACACATTTGCCGAGACGTAAATTACGGATGACTAATCCGC

TATATACACGCAAACGGAGCCTCAATATTTTTTATCTGTTTATTCATCCACATCGGACGA

GGGATTTATTATGGATCCTATATCTTTCAAGAAACATGAAACATCGGTGTAATTCTCTTA

TTCGCTGTAATAGCCACTGCATTTATAGGCTACGTTCTACCATGAGGACAAATATCTTTC

TGAGGAGCTACAGTAATCACTAACCTCCTCTCAGCAATCCCATACATTGGCCCAACAATT

GTAGAATGAATTTGAGGGGGTTTCTCAGTAGACAAAGCCACCCTAACACGATTCTTCGCA

TTCCACTTTATCCTCCCATTCATCATTGCAGCTCTTGTACTAGTCCATCTCTTGTTCCTA

CACGAGACCGGATCTAATAATCCCCTGGGAATCAACTCAAACGCAGACAAAATCCCTTTT

CACCCCTACTATACAGTAAAAGACTTCCTAGGGGTTATTATCTTTATT------------

>ZBSC0215

--AACCACTCATTCATCGAC

CTCCCCACTCCTCCAAACATCTCATCCTGATGAAACTTTGGCTCACTTCTAGGAATTTGC

TTAGTAATTCAAATTGCTACAGGACTTTTCCTAGCCATACATTATACAGCAGACACAACA

ACAGCATTTTCATCAGTATCCCATATCTGCCGAGACGTAAATTACGGATGACTAATCCGT

TATATACACGCAAACGGAGCCTCACTATTCTTCATCTGCCTATTTATCCATATCGGACGA

GGCATTTACTACGGATCCTACATCTTCCAAGAAACATGAAACATCGGTGTAATCCTCCTA

TTTGCCGTAATAGCTACCGCATTCATAGGGTATGTCCTACCATGAGGACAAATATCCTTC

TGAGGAGCCACAGTGATTACAAATCTCCTCTCAGCAATCCCATATATTGGCCCAACAATT

GTAGAATGAATCTGAGGAGGGTTCTCAGTAGACAAAGCCACCCTAACACGATTTTTCGCA

TTCCACTTTATCCTCCCTTTTATTATTACAGCCCTTGTCCTAGTCCACCTCCTATTTCTT

CACGAAACCGGATCCAATAACCCCCTAGGACTAAACTCTAATGCAGACAAAATCCCCTTT

CACCCCTATTACACAGTAAAAGATTTTCTCGGAGTAATTTTACTACTT------------

>ZBSC0216

--AACCACTCATTCATTGAC

CTTCCCACTCCCCCCAACATTTCATCCTGATGAAACTTTGGCTCACTCTTAGGAATTTGC

CTAGTAATTCAAATCGCCACAGGACTCTTTTTAGCCATACATTACACAGCAGATACAACA

ACAGCATTTTCATCAGTATCTCACATCTGCCGAGACGTAAATTACGGATGACTAATCCGT

TATATACATGCAAACGGAGCCTCAATATTCTTTATCTGCTTATTCATCCACATTGGACGA

GGAATCTACTATGGATCCTACATCTTCCACGAAACATGAAATATCGGAGTAATTCTCTTA

TTCGCCGTAATAGCCACTGCATTCATAGGATATGTCCTACCATGAGGACAAATATCTTTC

TGAGGGGCCACAGTCATTACAAATCTCCTCTCAGCAATCCCATACATTGGCCCAACAATC

GTAGAATGAATTTGAGGGGGCTTCTCAGTAGACAAAGCCACTTTAACACGATTCTTTGCA

TTCCATTTCATCCTTCCCTTTATCATCACAGCTCTTGTCCTAGTCCATTTATTATTTCTC

CACGAAACCGGATCCAACAATCCCCTAGGAATCAACTCCAACGCTGACAAAATCCCCTTC

CACCCTTACTATACAGTAAAAGATTACCTAGGAGTAATTTTACTACTC------------

>ZBSC0217

--AACCACTCATTCATTGAC

CTTCCCACTCCCCCCAACATTTCATCCTGATGAAACTTTGGCTCACTCTTAGGAATTTGC

CTAGTAATTCAAATCGCCACAGGACTCTTTTTAGCCATACATTACACAGCAGATACAACA

ACAGCATTTTCATCAGTATCTCACATCTGCCGAGACGTAAATTACGGATGACTAATCCGT

TATATACATGCAAACGGAGCCTCAATATTCTTTATCTGCTTATTCATCCACATTGGACGA

GGAATCTACTATGGATCCTACATCTTCCACGAAACATGAAATATCGGAGTAATTCTCTTA

TTCGCCGTAATAGCCACTGCATTCATAGGATATGTCCTACCATGAGGACAAATATCTTTC

TGAGGGGCCACAGTCATTACAAATCTCCTCTCAGCAATCCCATACATTGGCCCAACAATC

GTAGAATGAATTTGAGGGGGCTTCTCAGTAGACAAAGCCACTTTAACACGATTCTTTGCA

TTCCATTTCATCCTTCCCTTTATCATCACAGCTCTTGTCCTAGTCCATTTATTATTTCTC

CACGAAACCGGATCCAACAATCCCCTAGGAATCAACTCCAACGCTGACAAAATCCCCTTC

CACCCTTACTATACAGTAAAAGATTACCTAGGAGTAATTTTACTACTC------------

>ZBSC0207

--AACCACTCATTCATTGAT

CTTCCCACTCCCCCTAACATTTCATCTTGATGAAACTTTGGCTCACTCCTAGGAATTTGC

TTAATAATTCAAATCGCTACAGGACTTTTCCTAGCCATACATTATACAGCAGACACAACA

ACAGCATTCTCATCAGTATCCCATATCTGCCGAGACGTCAATTATGGATGACTGATCCGC

TATATACATGCAAACGGAGCTTCAATATTCTTTATTTGCCTATTCATTCACATCGGACGA

GGAATTTACTACGGATCTTATATCTTTCAAGAAACATGAAACATTGGGGTAATTCTCTTA

TTTGCCGTAATAGCCACCGCATTCATAGGGTATGTACTTCCATGAGGACAAATATCCTTC

TGAGGGGCCACAGTCATTACAAATCTTCTTTCAGCTATTCCATATATTGGCCCAACAATC

GTAGAATGAATTTGAGGAGGATTTTCAGTGGACAAAGCCACTTTAACACGATTTTTCGCA

TTTCACTTCATTCTCCCCTTTATTATCACAGCCTTAGTCCTAGTCCATCTCCTATTCCTT

CACGAAACCGGATCTAATAATCCCCTAGGCCTTAACTCCAACTCAGACAAAATCCCTTTT

CACCCATACTACACAGTAAAAGATTTTCTCGGAGTAATTCTACTACTC------------

>ZBSC0225

--AACCATTCATTCATTGAC

CTTCCCACTCCCCCCAACATTTCATCCTGATGAAACTTTGGCTCGCTCTTAGGAATTTGC

CTAATAATCCAAATCGCCACAGGACTCTTTTTAGCCATACATTACACAGCAGATACAACA

ACAGCATTTTCATCAGTAGCTCACATCTGCCGAGACGTAAATTACGGATGACTAATCCGT

TATATACATGCAAACGGAGCCTCAATATTCTTTATCTGCTTATTCATCCACATTGGACGA

GGAATCTACTATGGATCCTACATCTTCCACGAGACATGAAATATCGGAGTAATTCTCTTA

TTCGCCGTAATAGCCACTGCATTCATAGGATATGTCCTACCATGAGGACAGATATCTTTC

TGAGGAGCCACAGTCATTACAAATCTCCTCTCAGCAATCCCATACATTGGCCCAACAATC

GTAGAATGAATTTGAGGGGGCTTCTCAGTAGACAAAGCCACTTTAACACGATTCTTTGCA

TTCCACTTCATCCTTCCCTTTATCATCACAGCTCTTGTCCTAGTCCATTTATTATTTCTC

CACGAAACCGGATCCAACAATCCCCTAGGAATCAACTCTAACGCTGACAAAATCCCCTTC

CATCCTTACTATACAGTAAAAGATTACCTAGGAGTAATTTTACTACTC------------

>ZBSC0227

--AACCACTCATTCATTGAT

CTTCCCACTCCCCCTAATATCTCATCTTGATGAAACTTTGGTTCACTTCTAGGAATTTGC

TTAATTATTCAAATTGCTACAGGACTTTTCCTAGCCATACATTATACAGCAGACACAACA

ACAGCATTCTCATCAGTATCCCATATCTGCCGAGACGTCAATTATGGATGACTAATCCGC

TATATACATGCAAACGGAGCTTCAATATTCTTTATTTGCCTATTCATTCACATTGGACGA

GGAATTTACTACGGATCTTACATCTTTCAAGAAACATGAAACATTGGAGTAATTCTCTTA

TTTGCCGTAATAGCCACCGCATTCATAGGATATGTACTTCCATGAGGACAAATATCCTTC

TGAGGGGCCACAGTCATTACAAATCTTCTTTCAGCTATTCCATATATTGGCCCAACAATC

GTAGAATGAATTTGAGGAGGATTTTCAGTGGACAAAGCCACTTTAACACGATTTTTCGCA

TTCCACTTCATTCTCCCCTTTATTATCACAGCCCTAGTCCTAGTCCATCTTCTATTCCTT

CACGAAACCGGATCTAATAATCCCCTAGGCCTTAACTCCAACTCAGACAAAATCCCTTTT

CACCCATACTACACAGTAAAAGATTTTCTCGGAGTAATTCTACTACTT------------

>ZBSC0228

--AACCATTCATTCATTGAT

CTCCCCACTCCCCCCAACATCTCATCCTGATGAAACTTCGGCTCACTCCTAGGAATCTGC

TTAGTTATTCAAATCACCACAGGACTTTTCCTAGCTATACATTACACAGCAGACACAACA

ACAGCATTCTCATCAGTCTCACACATTTGCCGAGACGTAAATTACGGATGACTAATCCGC

TATATACACGCAAACGGAGCCTCAATATTTTTTATCTGTTTATTTATCCACATCGGACGA

GGGATTTATTATGGATCCTATATCTTTCAAGAAACATGAAACATCGGTGTAATTCTCTTA

TTCGCTGTAATAGCCACTGCATTTATAGGCTATGTTCTACCATGAGGACAAATATCTTTC

TGAGGAGCTACAGTAATCACTAATCTCCTCTCAGCAATCCCATACATTGGCCCAACAATT

GTAGAATGAATTTGAGGGGGTTTCTCAGTAGACAAAGCCACCCTAACACGATTCTTCGCA

TTCCACTTTATCCTCCCATTCATCATTGCAGCTCTTGTACTAGTTCATCTCTTGTTCCTA

CACGAGACCGGATCTAATAATCCCCTGGGAATCAACTCAAACGCAGACAAAATCCCTTTT

CACCCCTACTATACAGTAAAAGACTTCCTAGGGGTTATTATCTTTATT------------

>ZBSC0229

--AACCATTCATTCATTGAT

CTCCCCACTCCCCCCAACATCTCATCCTGATGAAACTTCGGCTCACTCCTAGGAATCTGC

TTAGTTATTCAAATCACCACAGGACTTTTCCTAGCTATACATTACACAGCAGACACAACA

ACAGCATTCTCATCAGTCTCACACATTTGCCGAGACGTAAATTACGGATGACTAATCCGC

TATATACACGCAAACGGAGCCTCAATATTTTTTATCTGTTTATTTATCCACATCGGACGA

GGGATTTATTATGGATCCTATATCTTTCAAGAAACATGAAACATCGGTGTAATTCTCTTA

TTCGCTGTAATAGCCACTGCATTTATAGGCTATGTTCTACCATGAGGACAAATATCTTTC

TGAGGAGCTACAGTAATCACTAATCTCCTCTCAGCAATCCCATACATTGGCCCAACAATT

GTAGAATGAATTTGAGGAGGTTTCTCAGTAGACAAAGCCACCCTAACACGATTCTTCGCA

TTCCACTTTATCCTCCCATTCATCATTGCAGCTCTTGTACTAGTCCATCTCTTGTTCCTA

CACGAGACCGGATCTAATAATCCCCTGGGAATCAACTCAAACGCAGACAAAATCCCTTTT

CACCCCTACTATACAGTAAAAGACTTCCTAGGGGTTATTATCTTTATT------------

>ZBSC0230

---AACCACTCATTCATCGAC

CTCCCCACTCCTCCAAACATCTCATCCTGATGAAACTTTGGCTCACTTCTAGGAATTTGC

TTAGTAATTCAAATTGCTACAGGACTTTTCCTAGCCATACATTATACAGCAGACACAACA

ACAGCATTTTCATCAGTATCCCATATCTGCCGAGACGTAAATTACGGATGACTAATCCGT

TATATACACGCAAACGGAGCCTCACTATTCTTCATCTGCCTATTTATCCATATCGGACGA

GGCATTTACTACGGATCCTACATCTTCCAAGAAACATGAAACATCGGTGTAATCCTCCTA

TTTGCCGTAATAGCTACCGCATTCATAGGGTACGTCCTACCATGAGGACAAATATCCTTC

TGAGGAGCCACAGTGATTACAAATCTCCTCTCAGCAATCCCGTATATTGGTCCAACAATT

GTAGAATGAATCTGAGGAGGGTTCTCAGTAGACAAAGCCACCCTAACACGATTTTTCGCA

TTCCATTTTATCCTCCCTTTTATTATTACAGCCCTTGTCCTAGTCCACCTCCTATTTCTT

CACGAAACCGGATCCAATAACCCCCTAGGACTAAACTCTAATGCAGACAAAATCCCCTTT

CACCCCTATTACACAGTAAAAGATTTTCTCGGAGTAATCTTACTACTT------------

>ZBSC0232

---AACCACTCATTCATCGAC

CTCCCCACTCCTCCAAACATCTCATCCTGATGAAACTTTGGCTCACTTCTAGGAATTTGC

TTAGTAATTCAAATTGCTACAGGACTTTTCCTAGCCATACATTATACAGCAGACACAACA

ACAGCATTTTCATCAGTATCCCATATCTGCCGAGACGTAAATTACGGATGACTAATCCGT

TATATACACGCAAACGGAGCCTCACTATTCTTCATCTGCCTATTTATCCATATCGGACGA

GGCATTTACTACGGATCCTACATCTTCCAAGAAACATGAAACATCGGTGTAATCCTCCTA

TTTGCCGTAATAGCTACCGCATTCATAGGGTACGTCCTACCATGAGGACAAATATCCTTC

TGAGGAGCCACAGTGATTACAAACCTCCTCTCAGCAATCCCATATATTGGCCCAACAATT

GTAGAATGAATCTGAGGGGGGTTCTCAGTAGACAAAGCCACCCTAACACGATTTTTCGCA

TTCCACTTTATCCTCCCTTTTATTATTACAGCCCTTGTCCTAGTCCACCTCCTATTTCTT

CACGAAACCGGATCCAATAACCCCCTAGGACTAAACTCTAATGCAGACAAAATCCCCTTT

CACCCCTATTACACAGTAAAAGATTTTCTCGGAGTAATTTTACTACTT------------

>ZBSC0233

---AACCACTCATTCATTGAC

CTTCCCACTCCCCCCAACATTTCATCCTGATGAAACTTTGGCTCACTCTTAGGAATTTGC

CTAGTAATTCAAATCGCCACAGGACTCTTTTTAGCCATACATTACACAGCAGATACAACA

ACAGCATTTTCATCAGTATCTCACATCTGCCGAGACGTAAATTACGGATGACTAATCCGT

TATATACATGCAAACGGAGCCTCAATATTCTTTATCTGCTTATTCATCCACATTGGACGA

GGAATCTACTATGGATCCTACATCTTCCACGAAACATGAAATATCGGAGTAATTCTCTTA

TTCGCCGTAATAGCCACTGCATTCATAGGATATGTCCTACCATGAGGACAAATATCTTTC

TGAGGAGCCACAGTCATTACAAATCTCCTCTCAGCAATCCCATACATTGGCCCAACAATC

GTAGAATGAATTTGAGGAGGCTTCTCAGTAGACAAAGCCACTTTAACACGATTCTTTGCA

TTCCATTTCATCCTTCCCTTTATCATCACAGCTCTTGTCCTAGTCCATTTATTATTTCTC

CACGAAACCGGATCCAACAATCCCCTAGGAATCAACTCCAACGCTGACAAAATCCCCTTC

CACCCTTACTATACAGTAAAAGATTACCTAGGAGTAATTTTACTACTC------------

>ZBSC0234

--AACCACTCATTCATTGAC

CTCCCTACTCCTCCAAACATCTCATCCTGATGAAACTTTGGCTCACTCCTAGGAATTTGC

TTAGTAATTCAAATTACTACAGGACTCTTCCTAGCCATACACTATACAGCAGATACAACA

ACAGCATTTTCATCAGTATCCCACATCTGCCGAGACGTAAATTACGGATGACTAATTCGT

TATATACACGCAAACGGAGCCTCAATATTCTTCATCTGCCTATTTATCCATATCGGACGA

GGAATTTACTACGGATCCTACATCTTTCAAGAAACATGAAACATCGGCGTAATCCTCCTA

TTTGCCGTAATAGCTACCGCATTCATGGGTTATGTCCTACCATGAGGACAAATATCCTTC

TGAGGCGCCACAGTCATTACAAACCTCCTCTCAGCAATCCCATACATTGGCCCAACAATT

GTAGAATGAATCTGAGGAGGCTTCTCAGTAGACAAAGCCACTCTAACACGATTCTTCGCA

TTCCACTTTATCCTTCCCTTTATTATTGCAGCTCTCGTCCTAGTCCACCTCTTATTTCTC

CACGAAACTGGATCCAATAACCCTCTAGGACTAAACTCCAACGCAGACAAAATCCCCTTT

CACCCCTATTATACAGTAAAAGATTTTCTCGGAGTAATCTTACTACTC------------

>ZBSC0235

--AACCACTCATTCATTGAC

CTCCCTACTCCTCCAAACATCTCATCCTGATGAAACTTTGGCTCACTCCTAGGAATTTGC

CTAGTAATTCAAATTACTACAGGACTCTTCCTAGCCATACACTATACAGCAGATACAACA

ACAGCATTTTCATCAGTATCCCACATCTGCCGAGACGTAAATTACGGATGACTAATTCGT

TATATACACGCAAACGGAGCCTCAATATTCTTCATCTGCCTATTTATCCATATCGGACGA

GGAATTTACTACGGATCCTACATCTTTCAAGAAACATGAAACATCGGTGTAATCCTCCTA

TTTGCCGTAATAGCTACCGCATTCATGGGTTACGTCCTACCATGAGGACAAATATCCTTC

TGAGGCGCCACAGTCATTACAAACCTCCTCTCAGCAATCCCATACATTGGCCCAACAATT

GTAGAATGAATCTGAGGAGGCTTCTCAGTAGACAAAGCCACTCTAACACGATTCTTCGCA

TTCCACTTTATCCTTCCCTTTATTATTACAGCTCTCGTCCTAGTCCACCTCTTATTTCTC

CACGAAACTGGATCCAATAACCCTCTAGGACTAAACTCCAACGCAGACAAAATCCCCTTT

CACCCCTATTATACAGTAAAAGATTTTCTCGGAGTAATCTTACTACTT------------

>ZBSC0238

--AACCACTCATTCATTGAC

CTTCCCACTCCCCCCAACATTTCATCCTGATGAAACTTTGGCTCACTCTTAGGAATTTGC

CTAGTAATTCAAATCGCCACAGGACTCTTTTTAGCCATACATTACACAGCAGATACAACA

ACAGCATTTTCATCAGTATCTCACATCTGCCGAGACGTAAATTACGGATGACTAATCCGT

TATATACATGCAAACGGAGCCTCAATATTCTTTATCTGCTTATTCATCCACATTGGACGA

GGAATCTACTATGGATCCTACATCTTCCACGAAACATGAAATATCGGAGTAATTCTCTTA

TTCGCCGTAATAGCCACTGCATTCATAGGATATGTCCTACCATGAGGACAAATATCTTTC

TGAGGGGCCACAGTCATTACAAATCTCCTCTCAGCAATCCCATACATTGGCCCAACAATC

GTAGAATGAATTTGAGGGGGCTTCTCAGTAGACAAAGCCACTTTAACACGATTCTTTGCA

TTCCATTTCATCCTTCCCTTTATCATCACAGCTCTTGTCCTAGTCCATTTATTATTTCTC

CACGAAACCGGATCCAACAATCCCCTAGGAATCAACTCCAACGCTGACAAAATCCCCTTC

CACCCTTACTATACAGTAAAAGATTACCTAGGAGTAATTTTACTACTC------------

>ZBSC0239

--AACCATTCATTCATTGAT

CTCCCCACTCCCCCCAACATCTCATCCTGATGAAACTTCGGCTCACTCCTAGGAATCTGC

TTAGTTATTCAAATCACCACAGGACTTTTCCTAGCTATACATTACACAGCAGACACAACA

ACAGCATTCTCATCAGTCTCACACATTTGCCGAGACGTAAATTACGGATGACTAATCCGC

TATATACACGCAAACGGAGCCTCAATATTTTTTATCTGTTTATTTATCCACATCGGACGA

GGGATTTATTATGGATCCTATATCTTTCAAGAAACATGAAACATCGGTGTAATTCTCTTA

TTCGCTGTAATAGCCACTGCATTTATAGGCTATGTTCTACCATGAGGACAAATATCTTTC

TGAGGAGCTACAGTAATCACTAACCTCCTCTCAGCAATCCCATACATTGGCCCAACAATT

GTAGAATGAATTTGAGGGGGTTTCTCAGTAGACAAAGCCACCCTAACACGATTCTTCGCA

TTCCACTTTATCCTCCCATTCATCATTGCAGCTCTTGTACTAGTTCATCTCTTGTTCCTA

CACGAGACCGGATCTAATAATCCCCTGGGAATCAACTCAAACGCAGACAAAATCCCTTTT

CACCCCTACTATACAGTAAAAGACTTCCTAGGGGTTATTATCTTTATT------------

>ZBSC0248

--AACCACTCATTCATTGAC

CTCCCCACACCTCCCAATATTTCATCCTGATGAAACTTTGGCTCACTCCTAGGAATTTGT

TTAATAATTCAAATTGCTACAGGACTCTTTCTAGCCATACACTACACAGCAGACACAACA

ACAGCATTTTCATCAGTATCTCACATCTGCCGAGATGTAAACTACGGATGACTAATCCGT

TATATACACGCAAACGGAGCCTCAATATTCTTTATTTGCTTATTCATTCACATTGGACGA

GGTATCTACTATGGATCATACATTTTTCAAGAAACATGAAACATCGGTGTAATTCTCCTA

TTCGCTGTGATAGCCACTGCATTCATAGGATATGTTCTACCATGAGGACAAATATCCTTC

TGAGGAGCCACAGTCATCACAAAcCTTCTTTCAGCAATTCCATATATTGGCCCAACAATT

GTAGAATGAATCTGAGGAGGCTTCTCAGTAGATAAAGCCACTCTAACACGATTCTTCGCA

TTCCACTTTATTCTTCCCTTTATCATCACAGCCCTTGTCCTAGTCCATCTCTTATTCCTC

CACGAAACTGGATCTAACAATCCCCTAGGACTTAACTCCAACGCAGATAAAATTCCCTTC

CACCCATACTACACAGTAAAAGATTTTCTTGGAGTAATCTTACTACTT------------

>ZBSC0249

--AACCACTCATTCATTGAT
CTTCCCACTCCCCCTAATATTTCATCTTGATGAAACTTTGGCTCACTCCTAGGAATTTGC
TTAATAATTCAAATCGCTACAGGACTTTTCCTAGCCATACATTATACAGCAGACACAACA
ACAGCATTCTCATCAGTATCCCATATCTGCCGAGACGTCAATTATGGATGACTAATCCGC
TATATACATGCAAACGGAGCTTCAATATTCTTTATTTGCCTATTCATTCACATCGGACGA
GGAATTTACTACGGATCTTATATCTTTCAAGAAACATGAAACATTGGAGTAATTCTCTTA
TTTGCCGTAATAGCCACCGCATTCATAGGGTATGTACTTCCATGAGGACAAATATCCTTC
TGAGGGGCCACAGTCATTACAAATCTTCTTTCAGCTATTCCATATATTGGCCCAACAATC
GTAGAATGAATTTGAGGAGGATTTTCAGTGGACAAAGCCACTTTAACACGATTTTTCGCA
TTTCACTTCATTCTCCCCTTTATTATCACAGCCTTAGTCCTAGTCCATCTCCTATTCCTT
CACGAAACCGGATCTAATAATCCCCTAGGCCTTAACTCCAACTCAGACAAAATCCCTTTT
CACCCATACTACACAGTAAAAGATTTTCTCGGAGTAATTCTACTACTC------------

>ZBSC0250

--AACCACTCATTCATTGAT
CTTCCCACTCCCCCTAATATCTCATCTTGATGAAACTTTGGCTCACTTCTAGGAATCTGC
TTAATAATTCAAATCGCTACAGGACTTTTCCTAGCCATACATTATACAGCAGACACAACA
ACAGCATTCTCATCAGTATCCCATATCTGCCGAGACGTCAATTATGGATGACTAATCCGC
TATATACATGCAAACGGAGCTTCAATATTCTTTATTTGCCTATTCATTCACATTGGACGA
GGAATTTACTACGGATCTTATATCTTTCAAGAAACATGAAACATTGGAGTAATTCTCTTA
TTTGCCGTAATAGCCACCGCATTCATAGGATATGTACTTCCATGAGGACAAATATCCTTC
TGAGGGGCCACAGTCATTACAAATCTTCTTTCAGCTATTCCATATATTGGCCCAACAATC
GTAGAATGAATTTGAGGAGGATTTTCAGTGGACAAAGCCACTTTAACACGATTTTTCGCA
TTTCACTTCATTCTCCCCTTTATTATCACAGCCTTAGTCCTAGTCCATCTTCTATTCCTT
CACGAAACCGGATCTAATAATCCCCTAGGCCTTAACTCCAACTCAGACAAAATCCCTTTT
CACCCATACTACACAGTAAAAGATTTTCTCGGAGTAATTCTACTACTT------------

>ZBSC0251

--AACCACTCATTCATTGAT
CTTCCCACTCCCCCTAATATCTCATCTTGATGAAACTTTGGTTCACTTCTAGGAATTTGC

TTAATCATTCAAATCGCTACAGGACTTTTCCTAGCCATACATTATACAGCAGACACAACA

ACAGCATTCTCATCAGTATCCCATATCTGCCGAGACGTCAATTATGGATGACTAATCCGC

TATATACATGCAAACGGAGCTTCAATATTCTTTATTTGCTTATTCATTCACATTGGACGA

GGAATTTACTACGGATCTTACATCTTTCAAGAAACATGAAACATTGGAGTAATTCTCTTA

TTTGCCGTAATAGCCACCGCATTCATAGGATATGTACTTCCATGAGGACAAATATCCTTC

TGAGGGGCCACAGTCATTACAAATCTTCTTTCAGCTATTCCATATATTGGCCCAACAATC

GTAGAATGAATTTGAGGAGGATTTTCAGTAGACAAAGCCACTTTAACACGATTTTTCGCA

TTTCACTTCATTCTCCCCTTTATTATCACAGCCCTAGTCCTAGTCCATCTTCTATTCCTT

CACGAAACCGGATCTAATAATCCCCTAGGCCTTAACTCCAACTCAGACAAAATCCCTTTT

CACCCATACTACACAGTAAAAGATTTTCTCGGAGTAATTCTACTACTT------------

>ZBSC0254

--AACCACTCATTCATCGAC

CTCCCCACTCCCCCAAACATCTCATCCTGATGAAACTTTGGCTCACTTCTAGGAATTTGC

TTAGTAATTCAAATTGCTACAGGACTTTTCCTAGCCATACATTATACAGCAGACACAACA

ACAGCATTTTCATCAGTATCCCATATCTGCCGAGACGTAAATTACGGATGACTAATCCGT

TATATACACGCAAACGGAGCCTCACTATTCTTCATCTGCCTATTCATCCATATCGGACGA

GGCATTTACTACGGATCCTACATCTTCCAAGAAACATGAAACATCGGTGTAATTCTCCTA

TTTGCCGTAATAGCTACCGCATTCATAGGATACGTCCTACCATGAGGACAAATATCCTTC

TGAGGAGCCACAGTGATTACAAATCTCCTCTCAGCAATCCCGTATATTGGTCCAACAATT

GTAGAATGAATCTGAGGGGGGTTCTCAGTAGACAAAGCCACCCTAACACGATTTTTCGCA

TTCCATTTTATCCTCCCTTTTATTATTACAGCCCTTGTCCTAGTCCACCTCCTATTTCTT

CACGAAACCGGATCCAATAACCCCCTAGGACTAAACTCTAATGCAGACAAAATCCCCTTT

CACCCCTATTACACAATAAAAGATTTTCTCGGAGTAATTTTACTACTT------------

>ZBSC0259

--AACCACTCATTCATTGAC

CTTCCCACTCCCCCCAACATTTCATCCTGATGAAACTTTGGCTCACTCTTAGGAATTTGC

CTAGTAATTCAAATCGCCACAGGACTCTTTTTAGCCATACATTACACAGCAGATACAACA

ACAGCATTTTCATCAGTATCTCACATCTGCCGAGACGTAAATTACGGATGACTAATCCGC

TATATACATGCAAACGGAGCCTCAATATTCTTTATCTGCTTATTCATCCACATTGGACGA

GGAATCTACTATGGATCCTACATCTTCCACGAAACATGAAATATCGGAGTAATTCTCTTA

TTCGCCGTAATAGCCACTGCATTCATAGGATATGTCCTACCATGAGGACAAATATCTTTC

TGAGGAGCCACAGTCATTACAAATCTCCTCTCAGCAATCCCATACATTGGCCCAACAATC

GTAGAATGAATTTGAGGGGGCTTCTCAGTAGACAAAGCCACTTTAACACGATTCTTTGCA

TTCCATTTCATCCTTCCCTTTATCATCACAGCTCTTGTCCTAGTCCATTTATTATTTCTC

CACGAAACCGGATCCAACAATCCCCTAGGAATCAACTCCAACGCTGACAAAATCCCCTTC

CACCCTTACTATACAGTAAAAGATTTCCTAGGAGTAATTTTACTACTC------------

>ZBSC0264
--AACCACTCATTCATTGAC
CTTCCCACTCCCCCCAACATTTCATCCTGATGAAACTTTGGCTCACTCTTAGGAATTTGC
CTAGTAATTCAAATCGCCACAGGACTCTTTTTAGCCATACATTACACAGCAGATACAACA
ACAGCATTTTCATCAGTATCTCACATCTGCCGAGACGTAAATTACGGATGACTAATCCGT
TATATACATGCAAACGGAGCCTCAATATTCTTTATCTGCTTATTCATCCACATTGGACGA
GGAATCTACTATGGATCCTACATCTTCCACGAAACATGAAATATCGGAGTAATTCTCTTA
TTCGCCGTAATAGCCACTGCATTCATAGGATATGTCCTACCATGAGGACAAATATCTTTC
TGAGGAGCCACAGTCATTACAAATCTCCTCTCAGCAATCCCATACATTGGCCCAACAATC
GTAGAATGAATTTGAGGGGGCTTCTCAGTAGACAAAGCCACTTTAACACGATTCTTTGCA
TTCCATTTCATCCTTCCCTTTATCATCACAGCTCTTGTCCTAGTCCATTTATTATTTCTC
CACGAAACCGGATCCAACAATCCCCTAGGAATCAACTCCAACGCTGACAAAATCCCCTTC
CACCCTTACTATACAGTAAAAGATTTCCTAGGAGTAATTTTACTACTC------------

>ZBSC0282
--AACCACTCATTCATCGAC
CTCCCCACTCCTCCAAACATCTCATCCTGATGAAACTTTGGCTCACTTCTAGGAATTTGC
TTAGTAATTCAAATTGCTACAGGACTTTTCCTAGCCATACATTATACAGCAGACACAACA
ACAGCATTTTCATCAGTATCCCATATCTGCCGAGACGTAAATTACGGATGACTAATCCGT
TATATACACGCAAACGGAGCCTCACTATTCTTCATCTGCCTATTTATCCATATCGGACGA
GGCATTTACTACGGATCCTACATCTTCCAAGAAACATGAAACATCGGTGTAATCCTCCTA
TTTGCCGTAATAGCTACCGCATTCATAGGGTACGTCCTACCATGAGGACAAATATCCTTC
TGAGGAGCCACAGTGATTACAAATCTCCTCTCAGCAATCCCATATATTGGCCCAACAATT
GTAGAATGAATCTGAGGGGGGTTCTCAGTAGACAAAGCCACCCTAACACGATTTTTCGCA
TTCCACTTTATCCTCCCTTTTATTATTACAGCCCTTGTCCTAGTCCACCTCCTATTTCTT
CACGAAACCGGATCCAATAACCCCCTAGGACTAAACTCTAATGCAGACAAAATCCCCTTT
CACCCCTATTACACAGTAAAAGATTTTCTCGGAGTAATTTTACTACTT------------

>ZBSC0284
--AACCACTCATTCATCGAC
CTCCCCACTCCTCCAAACATCTCATCCTGATGAAACTTTGGCTCACTTCTAGGAATTTGC
TTAGTAATTCAAATTGCTACAGGACTTTTCCTAGCCATACATTATACAGCAGACACAACA
ACAGCATTTTCATCAGTATCCCATATCTGCCGAGACGTAAATTACGGATGACTAATCCGT

TATATACACGCAAACGGAGCCTCACTATTCTTCATCTGCCTATTTATCCATATCGGACGA

GGCATTTACTACGGATCCTACATCTTCCAAGAAACATGAAACATCGGTGTAATCCTCCTA

TTTGCCGTAATAGCTACCGCATTCATAGGATACGTCCTACCATGAGGACAAATATCCTTC

TGAGGAGCCACAGTGATTACAAATCTCCTCTCAGCAATCCCATATATTGGCCCAACAATT

GTAGAATGAATCTGAGGGGGGTTCTCAGTAGACAAAGCCACCCTAACACGATTTTTCGCA

TTCCACTTTATCCTCCCTTTTATTATTACAGCCCTTGTCCTAGTCCACCTCCTATTTCTT

CACGAAACCGGATCCAATAACCCCCTAGGACTAAACTCTAATGCAGACAAAATCCCCTTT

CACCCCTATTACACAGTAAAAGATTTTCTCGGAGTAATTTTACTACTT------------

>ZBSC0285

--AACCACTCATTCATCGAC

CTCCCCACTCCTCCAAACATCTCATCCTGATGAAACTTTGGCTCACTTCTAGGAATTTGC

TTAGTAATTCAAATTGCTACAGGACTTTTCCTAGCCATACATTATACAGCAGACACAACA

ACAGCATTTTCATCAGTATCCCATATCTGCCGAGACGTAAATTACGGATGACTAATCCGT

TATATACACGCAAACGGAGCCTCACTATTCTTCATCTGCCTATTTATCCATATCGGACGA

GGCATTTACTACGGATCCTACATCTTCCAAGAAACATGAAACATCGGTGTAATCCTCCTA

TTTGCCGTAATAGCTACCGCATTCATAGGGTACGTCCTACCATGAGGACAAATATCCTTC

TGAGGAGCCACAGTGATTACAAATCTCCTCTCAGCAATCCCATATATTGGCCCAACAATT

GTAGAATGAATCTGAGGGGGGTTCTCAGTAGACAAAGCCACCCTAACACGATTTTTCGCA

TTCCACTTTATCCTCCCTTTTATTATTACAGCCCTTGTCCTAGTCCACCTCCTATTTCTT

CACGAAACCGGATCCAATAACCCCCTAGGACTAAACTCTAATGCAGACAAAATCCCCTTT

CACCCCTATTACACAGTAAAAGATTTTCTCGGAGTAATTTTACTACTT------------

>ZBSC0287

--AACCATTCATTCATTGAT

CTCCCCACTCCCCCCAACATCTCATCCTGATGAAACTTCGGCTCACTCCTAGGAATTTGC

TTAGTTATTCAAATCACCACAGGACTTTTCCTAGCTATACATTACACAGCAGACACAACA

ACAGCATTCTCATCAGTCTCACACATTTGCCGAGACGTAAATTACGGATGACTAATCCGC

TATATACACGCAAACGGAGCCTCAATATTTTTTATCTGTTTATTCATCCACATCGGACGA

GGGATTTATTATGGATCCTATATCTTTCAAGAAACATGAAACATCGGTGTAATTCTCTTA

TTCGCTGTAATAGCCACTGCATTTATAGGCTACGTTCTACCATGAGGACAAATATCTTTC

TGAGGAGCTACAGTAATCACTAACCTCCTCTCAGCAATCCCATACATTGGCCCAACAATT

GTAGAATGAATTTGAGGGGGTTTCTCAGTAGACAAAGCCACCCTAACACGATTCTTCGCA

TTCCACTTTATCCTCCCATTCATCATTGCAGCTCTTGTACTAGTCCATCTCTTGTTCCTA

CACGAGACCGGATCTAATAATCCCCTGGGAATCAACTCAAACGCAGACAAAATCCCTTTT

CACCCCTACTATACAGTAAAAGACTTCCTAGGGGTTATTATCTTTATT------------

>ZBSC0288
--AACCACTCATTCATCGAC
CTCCCCACTCCTCCAAACATCTCATCCTGATGAAACTTTGGCTCACTTCTAGGAATTTGC
TTAGTAATTCAAATTGCTACAGGACTTTTCCTAGCCATACATTATACAGCAGACACAACA
ACAGCATTTTCATCAGTATCCCATATCTGCCGAGACGTAAATTACGGATGACTAATCCGT
TATATACACGCAAACGGAGCCTCACTATTCTTCATCTGCCTATTTATCCATATCGGACGA
GGCATTTACTACGGATCCTACATCTTCCAAGAAACATGAAACATCGGTGTAATCCTCCTA
TTTGCCGTAATAGCTACCGCATTCATAGGATACGTCCTACCATGAGGACAAATATCCTTC
TGAGGAGCCACAGTGATTACAAATCTCCTCTCAGCAATCCCATATATTGGCCCAACAATT
GTAGAATGAATCTGAGGGGGGTTCTCAGTAGACAAAGCCACCCTAACACGATTTTTCGCA
TTCCACTTTATCCTCCCTTTTATTATTACAGCCCTTGTCCTAGTCCACCTCCTATTTCTT
CACGAAACCGGATCCAATAACCCCCTAGGACTAAACTCTAATGCAGACAAAATCCCCTTT
CACCCCTATTACACAGTAAAAGATTTTCTCGGAGTAATTTTACTACTT------------

>ZBSC0297
--AACCACTCATTCATTGAT
CTTCCCACTCCCCCTAATATTTCATCTTGATGAAACTTTGGCTCACTCCTAGGAATTTGC
TTAATAATTCAAATCGCTACAGGACTTTTCCTAGCCATACATTATACAGCAGACACAACA
ACAGCATTCTCATCAGTATCCCATATCTGCCGAGACGTCAATTATGGATGACTAATCCGC
TATATACATGCAAACGGAGCTTCAATATTCTTTATTTGCCTATTCATTCACATCGGACGA
GGAATTTATTACGGATCTTATATCTTTCAAGAAACATGAAACATTGGAGTAATTCTCTTA
TTTGCCGTAATAGCCACCGCATTCATAGGATATGTACTTCCATGAGGACAAATATCCTTC
TGAGGGGCCACAGTCATTACAAATCTTCTTTCAGCTATTCCATATATTGGCCCAACAATC
GTAGAATGAATTTGAGGAGGATTTTCAGTGGACAAAGCCACTTTAACACGATTTTTCGCA
TTTCACTTCATCCTCCCCTTTATTATCACAGCCTTAGTCCTAGTCCATCTCCTATTCCTT
CACGAAACCGGATCTAATAATCCCCTAGGCCTCAACTCCAACTCAGACAAAATCCCTTTT
CACCCATACTACACAGTAAAAGATTTTCTCGGAGTAATTCTACTACTC------------

>ZBSC0298
--AACCACTCATTCATTGAT
CTTCCCACTCCCCCTAATATTTCATCTTGATGAAACTTTGGCTCACTCCTAGGAATTTGC
TTAATAATTCAAATCGCTACAGGACTTTTCCTAGCCATACATTATACAGCAGACACAACA
ACAGCATTCTCATCAGTATCCCATATCTGCCGAGACGTCAATTATGGATGACTAATCCGC
TATATACATGCAAACGGAGCTTCAATATTCTTTATTTGCCTATTCATTCACATCGGACGA
GGAATTTACTACGGATCTTATATCTTTCAAGAAACATGAAACATTGGAGTAATTCTCTTA

TTTGCCGTAATGGCCACCGCATTCATAGGGTATGTACTTCCATGAGGACAAATATCCTTC
TGAGGGGCCACAGTCATCACAAATCTTCTTTCAGCTATTCCATATATTGGCCCAACAATC
GTAGAATGAATTTGAGGAGGATTTTCAGTGGACAAAGCCACTTTAACACGATTTTTCGCA
TTTCACTTCATTCTCCCCTTTATTATCACAGCCTTAGTCCTAGTCCATCTCCTATTCCTT
CACGAAACCGGATCTAATAATCCCCTAGGCCTTAACTCCAACTCAGACAAAATCCCTTTT
CACCCATACTACACAGTAAAAGATTTTCTCGGAGTAATTCTACTACTC------------

>ZBSC0299
--AACCATTCATTCATTGAT
CTCCCCACTCCCCCCAACATCTCATCCTGATGAAACTTCGGCTCACTCCTAGGAATCTGC
TTAGTTATTCAAATCACCACAGGACTTTTCCTAGCTATACATTACACAGCAGACACAACA
ACAGCATTCTCATCAGTCTCACACATTTGCCGAGACGTAAATTACGGATGACTAATCCGC
TATATACACGCAAACGGAGCCTCAATATTTTTATCTGTTTATTTATCCACATCGGACGA
GGGATTTATTATGGATCCTATATCTTTCAAGAAACATGAAACATCGGTGTAATTCTCTTA
TTCGCTGTAATAGCCACTGCATTTATAGGCTATGTTCTACCATGAGGACAAATATCTTTC
TGAGGAGCTACAGTAATCACTAACCTCCTCTCAGCAATCCCATACATTGGCCCAACAATT
GTAGAATGAATTTGAGGGGGTTTCTCAGTAGACAAAGCCACCCTAACACGATTCTTCGCA
TTCCACTTTATCCTCCCATTCATCATTGCAGCTCTTGTACTAGTTCATCTCTTGTTCCTA
CACGAGACCGGATCTAATAATCCCCTGGGAATCAACTCAAACGCAGACAAAATCCCTTTT
CACCCCTACTATACAGTAAAAGACTTCCTAGGGGTTATTATCTTTATT------------

>ZBSC0300
--AACCACTCATTCATTGAT
CTTCCCACTCCCCCTAATATTTCATCTTGATGAAACTTTGGCTCACTCCTAGGAATTTGC
TTAATAATTCAAATCGCTACAGGACTTTTCCTAGCCATACATTATACAGCAGACACAACA
ACAGCATTCTCATCAGTATCCCATATCTGCCGAGACGTCAATTATGGATGACTAATCCGC
TATATACATGCAAACGGAGCTTCAATATTCTTTATTTGCCTATTCATTCACATCGGACGA
GGAATTTACTACGGATCTTATATCTTTCAAGAAACATGAAACATTGGAGTAATTCTCTTA
TTTGCCGTAATAGCCACCGCATTCATAGGGTATGTACTTCCATGAGGACAAATATCCTTC
TGAGGGGCCACAGTCATTACAAATCTTCTTTCAGCTATTCCATATATTGGCCCAACAATC
GTAGAATGAATTTGAGGAGGATTTTCAGTGGACAAAGCCACTTTAACACGATTTTTCGCA
TTTCACTTCATTCTCCCCTTTATTATCACAGCCTTAGTCCTAGTCCATCTCCTATTCCTT
CACGAAACCGGATCTAATAATCCCCTAGGCCTTAACTCCAACTCAGACAAAATCCCTTTT
CACCCATACTACACAGTAAAAGATTTTCTCGGAGTAATTCTACTACTC------------

>ZBSC0301

--AACCACTCATTCATTGAT

CTTCCCACTCCCCCTAATATTTCATCTTGATGAAACTTTGGCTCACTCCTAGGAATTTGC

TTAACAATTCAAATCGCTACAGGACTTTTCCTAGCCATACATTATACAGCAGACACAACA

ACAGCATTCTCATCAGTATCCCATATCTGCCGAGACGTCAATTATGGATGACTAATCCGC

TATATACATGCAAACGGAGCTTCAATATTCTTTATTTGCCTATTCATTCACATCGGACGA

GGAATTTACTACGGATCTTATATCTTTCAAGAAACATGAAACATTGGAGTAATTCTCTTA

TTTGCCGTAATAGCCACCGCATTCATAGGGTATGTACTTCCATGAGGACAAATATCCTTC

TGAGGGGCCACAGTCATTACAAATCTTCTTTCAGCTATTCCATATATTGGCCCAACAATC

GTAGAATGAATTTGAGGAGGATTTTCAGTGGACAAAGCCACTTTAACACGATTTTTCGCA

TTTCACTTCATTCTCCCCTTTATTATCACAGCCTTAGTCCTAGTCCATCTCCTATTCCTT

CACGAAACCGGATCTAATAATCCCCTAGGCCTTAACTCCAACTCAGACAAAATCCCTTTT

CACCCATACTACACAGTAAAAGATTTTCTCGGAGTAATTCTACTACTC------------

>ZBSC0321

--AACCACTCATTCATTGAC

CTTCCCACACCTCCTAATATTTCATCCTGATGAAACTTTGGCTCCCTCCTAGGAATTTGT

TTAGTAATCCAAATTGCTACAGGACTCTTTTTAGCCATACACTACACAGCAGACACAACA

ACAGCATTTTCATCAGTATCTCACATCTGCCGAGATGTAAACTACGGATGACTAATCCGT

TACATACACGCAAACGGAGCCTCAATATTCTTCATTTGCTTATTCATTCACATTGGACGA

GGTATCTACTATGGATCATACATTTTTCAAGAAACATGAAACATCGGTGTAATTCTCCTA

TTCGCTGTGATAGCCACTGCATTCATAGGATATGTTCTACCATGAGGACAAATATCCTTC

TGAGGAGCTACAGTCATCACAAATCTTCTTTCAGCAATTCCATATATTGGCCCAACAATT

GTAGAATGAATCTGAGGAGGCTTCTCAGTAGATAAAGCTACCCTAACACGATTCTTTGCA

TTCCACTTCATTCTTCCCTTTATTATCACAGCCCTTGTCCTAGTCCATCTCTTATTCCTC

CACGAAACTGGGTCTAACAATCCCCTAGGACTTAACTCCAACGCAGATAAAATTCCCTTC

CACCCATACTACACAGTAAAAGATTTTCTTGGAGTAATTTTACTACTT------------

>ZBSC0322

--AATCACTCATTCATTGAC

CTTCCCACACCTCCTAATATTTCATCCTGATGAAACTTTGGCTCCCTCCTAGGAGTTTGT

TTAGTAATCCAAATTGCTACAGGACTCTTTTTAGCCATACACTACACAGCAGACACAACA

ACAGCATTTTCATCAGTATCTCACATCTGCCGAGATGTAAACTACGGATGACTAATCCGT

TATATACACGCAAACGGAGCCTCAATATTCTTCATTTGCTTATTCATTCATATTGGACGA

GGAATCTACTATGGATCATATATTTTTCAAGAAACATGAAACATCGGTGTAATTCTCTTA

TTCGCTGTGATAGCCACTGCATTCATAGGGTATGTTCTACCATGAGGACAAATATCCTTC

TGAGGCGCCACAGTTATCACAAATCTTCTTTCAGCAATTCCATATATTGGCCCAACAGTT

GTAGAATGAATCTGAGGAGGCTTTTCAGTAGATAAAGCCACTCTAACACGATTCTTTGCA

TTCCACTTTATTCTCCCCTTTATTATCACAGCCCTTGTCCTAGTCCATCTCTTATTCCTC

CACGAAACTGGATCTAACAATCCCCTAGGACTTAACTCCAACGCAGATAAAATTCCCTTC

CACCCATACTACACAGTAAAAGATTTTCTTGGAGTAATTTTACTACTT------------

>ZBSC0338

--AACCACTCATTCATTGAC

CTCCCCACACCTCCCAATATTTCATCCTGATGAAACTTTGGCTCACTCCTAGGAATTTGT

TTAGTAATTCAAATTGCTACAGGACTCTTTCTAGCCATACACTACACAGCAGACACAACA

ACAGCATTTTCATCAGTATCTCACATCTGCCGAGATGTAAACTATGGATGACTAATCCGT

TATATACACGCAAACGGAGCCTCAATATTCTTTATTTGCTTATTCATTCACATTGGACGA

GGtATCTACTATGGATCATACATTTTTCAAGAAACATGAAACATCGGTGTAATTCTCCTA

TTCGCCGTGATAGCCACTGCATTCATAGGATATGTTCTACCATGAGGACAAATATCCTTC

TGAGGAGCCACAGTCATCACAAACCTTCTTTCAGCAATTCCATATATTGGCCCAACAATC

GTAGAATGAATCTGAGGAGGCTTCTCAGTAGATAAAGCCACTCTAACACGATTCTTCGCA

TTCCACTTTATTCTTCCCTTTATTATCACAGCCCTTGTCCTAGTCCATCTCTTATTCCTC

CACGAAACTGGATCTAACAATCCCCTAGGACTTAACTCCAACGCAGATAAAATTCCCTTC

CACCCATACTACACAGTAAAAGATTTTCTTGGAGTAATTTTACTACTT------------

>ZBSC0339

--AATCACTCATTCATTGAC

CTTCCCACACCTCCTAATATTTCATCCTGATGAAACTTTGGGTCCCTCCTAGGAATTTGT

TTAGTAATCCAAATTGCTACAGGACTCTTTTTAGCCATACACTACACAGCAGACACAACA

ACAGCATTTTCATCAGTATCTCACATCTGCCGAGATGTAAACTACGGATGACTAATCCGT

TATATACACGCAAACGGAGCCTCAATATTCTTCATTTGCTTATTCATTCATATTGGGCGA

GGAATCTACTATGGATCATATATTTTTCAAGAAACATGAAACATCGGTGTAATCCTCCTA

TTCGCTGTGATAGCCACTGCATTCATAGGATATGTTCTACCATGAGGACAAATATCCTTC

TGAGGAGCCACAGTTATCACAAATCTTCTTTCAGCAATTCCATATATTGGCCCAACAATT

GTAGAATGAATCTGAGGAGGCTTTTCAGTAGATAAAGCCACTCTAACACGATTCTTTGCA

TTCCACTTTATTCTTCCCTTTATTATCACAGCCCTTGTCCTAGTCCATCTCTTATTCCTC

CACGAAACTGGATCTAACAATCCCCTAGGACTTAACTCCAACGCAGATAAAATTCCCTTC

CACCCATACTACACAGTAAAAGATTTTCTTGGAGTAATTTTACTACTT------------

>ZBSC0344

--AATCACTCATTCATTGAC

CTTCCCACACCTCCTAATATTTCATCCTGATGAAACTTTGGGTCCCTCCTAGGAATTTGT

TTAGTAATCCAAATTGCTACAGGACTCTTTTTAGCCATACACTACACAGCAGACACAACA

ACAGCATTTTCATCAGTATCTCACATCTGCCGAGATGTAAACTACGGATGACTAATCCGT

TATATACACGCAAACGGAGCCTCAATATTCTTCATTTGCTTATTCATTCATATTGGACGA

GGAATCTACTATGGGTCATATATTTTTCAAGAAACATGAAACATCGGTGTAATCCTCCTA

TTCGCTGTGATAGCCACTGCATTCATAGGGTATGTTCTACCATGAGGACAAATATCCTTC

TGAGGAGCCACAGTTATCACAAATCTTCTTTCAGCAATTCCATATATTGGCCCAACAATT

GTAGAATGAATCTGAGGAGGCTTTTCAGTAGATAAAGCCACTCTAACACGATTCTTTGCA

TTCCACTTTATTCTTCCCTTTATTATCACAGCCCTTGTCCTAGTCCATCTCTTATTCCTC

CACGAAACTGGATCTAACAACCCCCTAGGACTTAACTCCAACGCAGATAAAATTCCCTTC

CACCCATACTACACAGTAAAAGATTTTCTTGGAGTAATTTTACTACTT------------

>ZBSC0348

--AACCACTCATTCATTGAC

CTCCCCACTCCCCCCAACATTTCATCCTGATGAAACTTTGGCTCACTCTTAGGAATTTGC

CTAGTAATTCAAATCGCCACAGGACTCTTTTTAGCCATACATTACACAGCAGATACAACA

ACAGCATTTTCATCAGTATCTCACATCTGCCGAGACGTAAATTACGGATGACTAATCCGT

TATATACATGCAAACGGAGCCTCAATATTCTTTATCTGCTTATTCATCCACATTGGACGA

GGAATCTACTATGGATCCTACATCTTCCACGAAACATGAAATATCGGAGTAATTCTCTTA

TTCGCCGTAATAGCCACTGCATTCATAGGATATGTCCTACCATGAGGACAGATATCTTTC

TGAGGGGCCACAGTCATTACAAATCTCCTCTCAGCAATCCCATACATCGGCCCAACAATC

GTAGAATGAATTTGAGGAGGCTTCTCAGTAGACAAAGCCACTTTAACACGATTCTTTGCA

TTCCATTTCATCCTTCCCTTTATCATCACAGCTCTTGTCCTAGTCCATTTATTATTTCTC

CACGAAACCGGATCCAACAATCCCCTAGGAATCAACTCTAACGCTGACAAAATCCCCTTC

CACCCTTACTATACAGTAAAAGATTACCTAGGAGTAATTTTACTACTC------------

>ZBSC0369

--AACCACTCATTTATTGAT

CTCCCTACTCCCCCTAACATTTCATCTTGATGAAACTTCGGCTCACTTCTAGGAATCTGC

CTAATAATCCAAATTGCCACAGGATTATTCCTAGCCATACATTACACAGCAGACACAACA

ACAGCATTCTCATCAGTATCACATATTTGCCGAGACGTAAATTACGGATGACTTATCCGC

TACATACACGCCAACGGAGCCTCAATATTTTTTATTTGCTTATTTATTCACATCGGACGA

GGAATTTACTATGGATCCTACATCTTCCAAGAAACATGAAATATCGGTATCATCCTTCTT

TTCGCTGTAATAGCCACCGCATTCATAGGCTATGTACTTCCATGAGGACAGATGTCCTTC

TGAGGAGCTACAGTAATCACTAACCTCCTCTCAGCAATCCCATACATTGGTCCAACAATC

GTAGAGTGAATCTGAGGAGGATTCTCAGTAGACAAAGCCACCCTAACACGATTCTTCGCA

TTCCATTTCATCCTCCCATTCATCATTGCAGCCCTTGTACTTGTCCATCTCTTGTTTCTT

CACGAGACCGGATCTAACAATCCCCTGGGAATCAACTCAAATGCAGACAAAATCCCTTTC

CACCCCTATTACACAGTAAAAGACTTTCTAGGGGTTATTATCCTCATC------------

>ZBSC0370

--AATCACTCATTCATTGAC

CTTCCCACACCTCCTAATATTTCATCCTGATGAAACTTTGGGTCCCTCCTAGGAATTTGT

TTAGTAATCCAAATTGCTACAGGACTCTTTTTAGCCATACACTACACAGCAGACACAACA

ACAGCATTTTCATCAGTATCTCACATCTGCCGAGACGTAAACTACGGATGACTAATCCGT

TATATACACGCAAACGGAGCCTCAATATTCTTCATTTGCTTATTCATTCATATTGGACGA

GGAATCTACTATGGATCATATATTTTTCAAGAAACATGAAACATCGGTGTAATCCTCCTA

TTCGCTGTGATAGCCACTGCATTCATAGGATATGTTCTACCATGAGGACAAATATCCTTC

TGAGGAGCCACAGTTATCACAAATCTTCTTTCAGCAATTCCATATATTGGCCCAACAATT

GTAGAATGAATCTGAGGAGGCTTTTCAGTAGATAAAGCCACTCTAACACGATTCTTTGCA

TTCCACTTTATTCTTCCCTTTATTATCACAGCCCTTGTCCTAGTCCATCTCTTATTCCTC

CACGAAACTGGATCTAACAATCCCCTAGGACTTAACTCCAACGCAGATAAAATTCCCTTC

CACCCATACTACACAGTAAAAGATTTTCTTGGAGTAATTTTACTACTT------------

>15189 /transl_table=2

----------------------CAATATTCTTTATCTGCTTATTCATCCACATCGGACGA

GGAATCTACTATGGATCCTACATCTTCCACGAAACATGAAATATCGGAGTAATTCTCTTA

TTCGCCGTAATAGCCACTGCATTCATAGGATATGTCCTACCATGAGGACAAATATCTTTC

TGAGGGGCCACAGTCATTACAAATCTCCTCTCAGCAATCCCATACATTGGCCCAACAA--

>25768 /transl_table=2

----------------------CAATATTCTTTATCTGCTTATTCATCCACATTGGACGA
GGAATCTACTATGGATCCTACATCTTCCACGAAACATGAAATATCGGAGTAATTCTCTTA
TTCGCCGTAATAGCCACTGCATTCATAGGATATGTCCTACCATGAGGACAGATATCTTTC
TGAGGGGCCACAGTCATTACAAATCTCCTCTCAGCAATCCCATACATTGGCCCAACAA--

>8249 /transl_table=2

----------------------CAATATTCTTCATCTGCCTATTTATCCATATCGGACGA
GGAATTTACTACGGATCCTACATCTTTCAAGAAACATGAAACATCGGCGTAATCCTCCTA
TTTGCCGTAATAGCTACCGCATTCATGGGTTATGTCCTACCATGAGGACAAATATCCTTC
TGAGGCGCCACAGTCATTACAAACCTCCTCTCAGCAATCCCATACATTGGCCCAACAA--

>8248 /transl_table=2

----------------------CAATATTCTTCATCTGCCTATTTATCCATATCGGACGA
GGAATTTACTACGGATCCTACATCTTTCAAGAAACATGAAACATCGGCGTAATCCTCCTA
TTTGCCGTAATAGCTACCGCATTCATGGGTTATGTCCTACCATGAGGACAAATATCCTTC
TGAGGCGCCACAGTCATTACAAACCTCCTCTCAGCAATCCCATACATTGGCCCAACAA--

>25754 /transl_table=2

----------------------CAATATTCTTTATTTGCCTATTCATTCACATCGGACGA

GGAATTTACTACGGATCTTATATCTTTCAAGAAACATGAAACATTGGAGTAATTCTCTTA

TTTGCCGTAATAGCCACCGCATTCATAGGGTATGTACTTCCATGAGGACAAATATCCTTC

TGAGGGGCCACAGTCATTACAAATCTTCTTTCAGCTATTCCATATATTGGCCCAACAA--

>37777 /transl_table=2

----------------------CAATATTCTTTATCTGCTTATTCATCCACATTGGACGA

GGAATCTACTAtGGATCCTAcATCTTcCAcGAAACATGAAATATCGGAGTAATTCTCCTA

TTCGCCGTAATAGCCACTGCATTCATAGGATATGTCCTACCATGAGGACAGATATCTTTC

TGAGGGGCCACAGTCATTACAAATCTCCTCTCAGCAATCCCATACnTTGGCCCAACAA--

>37799 /transl_table=2

----------------------CAATATTCTTTATTTGCCTATTCATTCACATCGGACGA

GGAATTTACTACGGATCTTATATCTTTCAAGAAACATGAAACATTGGAGTAATTCTCTTA

TTTGCCGTAATAGCCACCGCATTCATAGGGTATGTACTTCCATGAGGACAAATATCCTTC

TGAGGGGCCACAGTCATTACAAATCTTCTTTCAGCTATTCCATATATTGGCCCAACAA--

>5044 /transl_table=2

----------------------CAATATTCTTTATTTGCCTATTCATTCACATTGGACGA

GGAATTTACTACGGATCTTACATCTTTCAAGAAACATGAAACATTGGAGTAATTCTCTTA

TTTGCCGTAATAGCCACCGCATTCATAGGATATGTACTTCCATGAGGACAAATATCCTTC

TGAGGGGCCACAGTCATTACAAATCTTCTTTCAGCTATTCCATATATTGGCCCAACAA--

>37796 /transl_table=2

----------------------CAATATTCTTTATTTGCCTATTCATTCACATTGGACGA

GGAATTTACTACGGATCTTACATCTTTCAAGAAACATGAAACATTGGAGTAATTCTCTTA

TTTGCCGTAATAGCCACCGCATTCATAGGATATGTACTTCCATGAGGACAAATATCCTTC

TGAGGGGCCACAGTCATTACAAATCTTCTTTCAGCTATTCCATATATTGGCCCAACAA--

>5035 /transl_table=2

-----------------------CAATATTTTTATCTGTTTATTCATTCACATCGGACGA

GGAATTTATTACGGATCCTATATCTTTCAAGAAACATGAAACATCGGTGTAATTCTCTTA

TTCGCTGTAATAGCCACTGCGTTCATAGGCTATGTTCTACCATGAGGACAAATATCTTTC

TGAGGAGCTACAGTAATCACTAATCTCCTTTCAGCAATCCCATACATTGGCCCAACAA--

>5050 /transl_table=2

----------------------CAATATTCTTCATCTGCCTATTCATCCACATTGGACGA

GGAATTTACTATGGATCCTATATCTTCCAAGAAACATGAAACATTGGTGTAATTCTTCTA

TTCGCCGTAATAGCTACCGCATTCATAGGATACGTCCTACCATGAGGACAAATATCCTTC

TGAGGGGCCACAGTTATTACAAACCTCCTCTCAGCAATCCCATACATTGGCCCAACAA--

>91-090-M-288 /transl_table=2

----------------------CAATATTCTTTATCTGCTTATTCATCCACATTGGACGA

GGAATCTACTATGGATCCTACATCTTCCACGAAACATGAAATATCGGAGTAATTCTCTTA
TTCGCCGTAATAGCCACTGCATTCATAGGATATGTCCTACCATGAGGACAAATATCTTTC
TGAGGGGCCACAGTCATTACAAATCTCCTCTCAGCAATCCCATACATTGGCCCAACAA--

>17927 /transl_table=2

----------------------CAAnnTTTATCATCTGCCTATTTATCCATATCGGACGA
GGCAtnTACTACGGATCCTACATCTTCCAAGAAACATGAAACATCGGAGTAATCCTCCTA
TTTGCCGTAATAGCTACCGCATTCATAGGGTATGTCCTACCATGAGGACAAATnTCCTTC
TGAGGAGCTACAGTAATTACGAATCTCCTCTCAGCAATCCCTTATATTGCACCAACAA--

>17240 /transl_table=2

----------------------CAATATTCTTTATCTGCTTATTCATCCACATTGGACGA
GGAATCTACTATGGATCCTACATCTTCCACGAAACATGAAATATCGGAGTAATTCTCTTA
TTCGCCGTAATAGCCACTGCATTCATAGGATATGTCCTACCATGAGGACAGATATCCTTC
TGAGGGGCCACAGTCATTACAAATCTCCTCTCAGCAATCCCATACATTGGCCCAACAA--

>21983 /transl_table=2

----------------------CAATATTCTTTATCTGCTTATTCATCCACATTGGACGA

GGAATCTACTATGGATCCTACATCTTCCACGAAACATGAAATATCGGAGTAATTCTCTTA

TTCGCCGTAATAGCCACTGCATTCATAGGATATGTCCTACCATGAGGACAAATATCTTTC

TGAGGGGCCACAGTCATTACAAATCTCCTCTCAGCAATCCCATACATTGGCCCAACAA--

>17002 /transl_table=2

----------------------CAATATTCTTTATCTGCTTATTCATCCACATTGGACGA

GGAATCTACTATGGATCCTACATCTTCCACGAAACATGAAATATCGGAGTAATTCTCTTA

TTCGCCGTAATAGCCACTGCATTCATAGGATATGTCCTACCATGAGGACAGATATCTTTC

TGAGGGGCCACAGTCATTACAAATCTCCTCTCAGCAATCCCATACATTGGCCCAACAA--

>22135 /transl_table=2

----------------------CAATATTTTTTATCTGTTTATTTATCCACATCGGACGA

GGAATTTATTATGGATCCTATATCTTTCAAGAAACATGAAACATCGGTGTAATTCTCCTA

TTCGCTGTAATAGCCACTGCATTTATAGGCTATGTTCTACCATGAGGACAAATATCTTTC

TGAGGAGCTACAGTAATTACTAATCTCCTCTCAGCAATCCCATACATTGGCccAACAA--

>M/9701/90 /transl_table=2

----------------------CAATATTTTTATCTGCCTATTCATCCATATCGGACGA
GGaATCTACTACGGATCCTACATCTTCCAAGAAACATGaAAcATCGGCGTAATCCTCCTA
TTCGCCGTAATAGCTACCGCATTCATAGGGTATGTCCTACCATGAGGACAAATATCCTTT
TGAGGAGcCACAGTTATCACAAACCTCCTCTCAGCAATCCCATATATTGGCCCAACAA--

>M/9041/89 /transl_table=2

----------------------CAATATTCTTTATCTGCTTATTCATCCACATCGGACGA
GGAATCTACTATGGATCCTACATCTTCCACGAAACATGAAATATCGGAGTAATTCTCTTA
TTCGCCGTAATAGCCACTGCATTCATAGGATATGTCCTACCATGAGGACAAATATCTTTC
TGAGGGGCCACAGTCATTACAAATCTCCTCTCAGCAATCCCATACATTGGCCCAACAA--

>M/9069/89 /transl_table=2

----------------------CAATATTTTTTATCTGCTTATTnATCCACATCGGACGA

GGaATCTACTATGGATCCTACATCTTCCAnGaAACATGAAATATCGGAGTAATTCTCTTA

TTCGCCGTAATAGCCACTGCATTCATAGGgTATGTCCTACCATGAGGACAgATATCTTTC

TGAGGnGCCACAGTCATTACAAATCTCCTCTCAGCnATCCCATACnTTGGCCCAACAA--

>M/9120/89 /transl_table=2

----------------------CAATATTCTTTATCTGCTTATTCATCCACATTGGACGA

GGAATCTACTATGGATCCTACATTTTCCACGAAACATGAAATATCGGAGTAATTCTCTTA

TTCGCCGTAATAGCCACTGCATTCATAGGATATGTCCTACCATGAGGACAGATATCTTTC

TGAGGGGCCACAGTCATTACAAATCTCCTCTCAGCAATCCCATACATTGGCCCAACAA--

>M/9192/89 /transl_table=2

----------------------CAATATTCTTTAttTGCCTATTCATTCACATCGGACGA

GGAATTTACTACGGATCTTATATCTTTCAAGAAACATGAAACATTGGAGTAATTCTCTTA

TTTGCCGTAATAGCCACCGCATTCATAGGATATGTACTTCCATGAGGACAAATATCCTTC

TGAGGGGCCACAGTCATTACAAATCTTCTTTCAGCTATTCCATATATTGGCCCAACAA--

>M/9703/90 /transl_table=2

----------------------CAATATTCTTTATTTGCTTATTTATTCACATCGGACGA

GGAATTTATTATGGATCCTATATTTTCCAAGAAACATGAAACATCGGTATCATCCTTCTT

TTCGCTGTAATAGCCACCGCATTCATAGGCTACGTACTTCCATGAGGACAGATGTCCTTC

TGAGGGGCTACAGTAATCACTAATCTCCTCTCAGCAATTCCATACATTGGCCCAACAA--

>M/9816/90 /transl_table=2

----------------------CAATATTTTTTATCTGTTTATTCATCCACATCGGACGA

GGAATTTATTATGGATCCTATATCTTTCAAGAAACATGAAACATCGGTGTAATTCTCTTA

TTCGCTGTAATAGCCACTGCATTTATAGGCTATGTTCTACCATGAGGACAAATATCTTTC

TGAGGAGCTACAGTAATCACTAATCTCCTCTCAGCAATCCCATACATTGGCCCAACAA--

>M/10016/90 /transl_table=2

----------------------CAATATTCTTCATCTGCCTATTCATCCACATCGGAnGA

GGAAGATACTATGGATCCTATATCTTCCAAGAAACATGAAACATTGGAGTAATTCTTCTA

TTCGCCGTAATAGCTACCGCATTCATAGGATACGTcCTACCATGAGGACAAATATCCTTC

TGAGGAGCCACAGCTATTACAAACCTCCTCTCAnCnnTCCCATACATTGCACCAACAA--

>M/1011/90 /transl_table=2

----------------------CAATATTTTTCATCTGCCTATTCATCCATATCGGACGA

GGGAGATACTACGGATCCTACATCTTTCAAGAAACATGAAACATCGGCGTAATCCTCCTA

TTTGCCGTAATAGCTACCGCATTCATAGGtTATGTCCTACCATGAGGACAAATATCCTTC

TGAGG-GCCACAGTCATTACAAACCTCCTCTCAGCAATCCCATACATTGGCCCAACAA--

>M5936

ATGACAAACATCCGAAAAATTCACCCCCTAATAAAAATAGTAAACCACTCATTCATTGAC

CTCCCTACTCCTCCAAACATCTCATCCTGATGAAACTTTGGCTCACTCCTAGGAATTTGC

TTAGTAATTCAAATTACTACAGGACTCTTCCTAGCCATACACTATACAGCAGATACAACA

ACAGCATTTTCATCAGTATCCCACATCTGCCGAGACGTAAATTACGGATGACTAATTCGT

TATATACACGCAAACGGAGCCTCAATATTCTTCATCTGCCTATTTATCCATATCGGACGA

GGAATTTACTACGGATCCTACATCTTTCAAGAAACATGAAACATCGGTGTAATCCTCCTA

TTTGCCGTAATAGCTACCGCATTCATGGGTTACGTCCTACCATGAGGACAAATATCCTTC

TGAGGCGCCACAGTCATTACAAACCTCCTCTCAGCAATCCCATACATTGGCCCAACAATT

GTAGAATGAATCTGAGGAGGCTTCTCAGTAGACAAAGCCACTCTAACACGATTCTTCGCA

TTCCACTTTATCCTTCCCTTTATTATTACAGCTCTCGTCCTAGTCCACCTCTTATTTCTC

CACGAAACTGGATCCAATAACCCTCTAGGACTAAACTCCAACGCAGACAAAATCCCCTTT

CACCCCTATTATACAGTAAAAGATTTTCTCGGAGTAATCTTACTACTTCTATTCTTCACA
ATCTTAGTCCTCTTCTTTCCCGACCTACTTGGAGACCCAGACAATTACACACCCGCCAAC
CCACTTAATACTCCCCCTCATATTAAACCCGAATGATATTTCTTATTTGCCTACGCTATT
CTCCGCTCTATTCCCAATAAACTAGGTGGAGTACTAGCCCTAGTACTTTCAATCCTCATT
CTAATTTTCCTACCACTAATCCATACATCAAAACAACGAAGCCTAATATTCCGACCAATT
TCACAAATACTCTACTGAATTTTAATCGCCAACCTACTTATCCTCACATGAATTGGAGGT
CAACCAGTAGAACACCCATTTATCATTATTGGCCAACTAGCCTCAATTAGCTATTTCTCC
ATCATCCTAATCTTTCTACCAATCGCAGGGATCATCGAAGACAAAATATTAAAATGATAT
>M5963
ATGACAAACATCCGAAAAATTCACCCCCTAATAAAAATAGTAAACCACTCATTCATTGAC
CTCCCTACTCCTCCAAACATCTCATCCTGATGAAACTTTGGCTCACTCCTAGGAATTTGC
TTAGTAATTCAAATTACTACAGGACTCTTCCTAGCCATACACTATACAGCAGATACAACA
ACAGCATTTTCATCAGTATCCCACATCTGCCGAGACGTAAATTACGGATGACTAATTCGT
TATATACACGCAAACGGAGCCTCAATATTCTTCATCTGCCTATTTATCCATATCGGACGA
GGAATTTACTACGGATCCTACATCTTTCAAGAAACATGAAACATCGGTGTAATCCTCCTA
TTTGCCGTAATAGCTACCGCATTCATGGGTTACGTCCTACCATGAGGACAAATATCCTTC
TGAGGCGCCACAGTCATTACAAACCTCCTCTCAGCAATCCCATACATTGGCCCAACAATT
GTAGAATGAATCTGAGGAGGCTTCTCAGTAGACAAAGCCACTCTAACACGATTCTTCGCA
TTCCACTTTATCCTTCCCTTTATTATTACAGCTCTCGTCCTAGTCCACCTCTTATTTCTC
CACGAAACTGGATCCAATAACCCTCTAGGACTAAACTCCAACGCAGACAAAATCCCCTTT
CACCCCTATTATACAGTAAAAGATTTTCTCGGAGTAATCTTACTACTTCTATTCTTCACA
ATCTTAGTCCTCTTCTTTCCCGACCTACTTGGAGACCCAGACAATTACACACCCGCCAAC
CCACTTAATACTCCCCCTCATATTAAACCCGAATGATATTTCTTATTTGCCTACGCTATT
CTCCGCTCTATTCCCAATAAACTAGGTGGAGTACTAGCCCTAGTACTTTCAATCCTCATT
CTAATTTTCCTACCACTAATCCATACATCAAAACAACGAAGCCTAATATTCCGACCAATT
TCACAAATACTCTACTGAATTTTAATCGCCAACCTACTTATCCTCACATGAATTGGAGGT
CAACCAGTAGAACACCCATTTATCATTATTGGCCAACTAGCCTCAATTAGCTATTTCTCC
ATCATCCTAATCTTTCTACCAATCGCAGGGATCATCGAAGACAAAATATTAAAATGATAT
>M5964
ATGACAAACATCCGAAAAATTCACCCCCTAATAAAAATAGTAAACCACTCATTCATTGAC
CTCCCTACTCCTCCAAACATCTCATCCTGATGAAACTTTGGCTCACTCCTAGGAATTTGC
TTAGTAATTCAAATTACTACAGGACTCTTCCTAGCCATACACTATACAGCAGATACAACA
ACAGCATTTTCATCAGTATCCCACATCTGCCGAGACGTAAATTACGGATGACTAATTCGT
TATATACACGCAAACGGAGCCTCAATATTCTTCATCTGCCTATTTATCCATATCGGACGA
GGAATTTACTACGGATCCTACATCTTTCAAGAAACATGAAACATCGGTGTAATCCTCCTA
TTTGCCGTAATAGCTACCGCATTCATGGGTTACGTCCTACCATGAGGACAAATATCCTTC
TGAGGCGCCACAGTCATTACAAACCTCCTCTCAGCAATCCCATACATTGGCCCAACAATT
GTAGAATGAATCTGAGGAGGCTTCTCAGTAGACAAAGCCACTCTAACACGATTCTTCGCA
TTCCACTTTATCCTTCCCTTTATTATTACAGCTCTCGTCCTAGTCCACCTCTTATTTCTC
CACGAAACTGGATCCAATAACCCTCTAGGACTAAACTCCAACGCAGACAAAATCCCCTTT
CACCCCTATTATACAGTAAAAGATTTTCTCGGAGTAATCTTACTACTTCTATTCTTCACA
ATCTTAGTCCTCTTCTTTCCCGACCTACTTGGAGACCCAGACAATTACACACCCGCCAAC
CCACTTAATACTCCCCCTCATATTAAACCCGAATGATATTTCTTATTTGCCTACGCTATT
CTCCGCTCTATTCCCAATAAACTAGGTGGAGTACTAGCCCTAGTACTTTCAATCCTCATT
CTAATTTTCCTACCACTAATCCATACATCAAAACAACGAAGCCTAATATTCCGACCAATT
TCACAAATACTCTACTGAATTTTAATCGCCAACCTACTTATCCTCACATGAATTGGAGGT
CAACCAGTAGAACACCCATTTATCATTATTGGCCAACTAGCCTCAATTAGCTATTTCTCC
ATCATCCTAATCTTTCTACCAATCGCAGGGATCATCGAAGACAAAATATTAAAATGATAT
>M-ANE1
ATGACAAACATCCGAAAAATTCACCCCCTACTAAAAATAGTAAACCACTCATTCATCGAC
CTCCCCACTCCTCCAAACATCTCATCCTGATGAAACTTTGGCTCACTTCTAGGAATTTGC

TTAGTAATTCAAATTGCTACAGGACTTTTCCTAGCCATACATTATACAGCAGACACAACA

ACAGCATTTTCATCAGTATCCCATATCTGCCGAGACGTAAATTACGGATGACTAATCCGT

TATATACACGCAAACGGAGCCTCACTATTCTTCATCTGCCTATTCATCCATATCGGACGA

GGCATTTACTACGGATCCTACATCTTCCAAGAAACATGAAACATCGGTGTAATCCTCCTA

TTTGCCGTAATAGCTACCGCATTCATAGGATACGTCCTACCATGAGGACAAATATCCTTC

TGAGGAGCCACAGTGATTACAAATCTCCTCTCAGCAATCCCGTATATTGGTCCAACAATT

GTAGAATGAATCTGAGGGGGGTTCTCAGTAGACAAAGCCACCCTAACACGATTTTTCGCA

TTCCATTTTATCCTCCCTTTTATTATTTCAGCCCTTGTCCTAGTCCACCTCCTATTTCTT

CACGAAACCGGATCCAATAACCCCCTAGGACTAAACTCTAATGCAGACAAAATCCCCTTT

CACCCCTATTACACAGTAAAAGATTTTCTCGGAGTAATTTTACTACTTCTATTCTTCATA

ATCCTAGTTCTCTTCTTTCCTGACCTACTTGGAGACCCAGACAATTACACACCTGCTAAC

CCACTCAACACACCTCCCCATATTAAACCCGAATGATATTTCTTATTTGCCTACGCTATT

CTCCGTTCCATCCCCAATAAACTAGGTGGAGTACTAGCCTTAGTATTATCAATCCTCATT

CTAATTTTCCTACCACTAATCCATACATCAAAACAACGAAGCCTAATATTCCGACCAATT

TCACAAATACTTTACTGAATTTTAATCGCTAACCTGCTTATCCTTACATGAATCGGGGGC

CAACCAGTAGAACACCCATTTATCATTATTGGCCAACTAGCCTCAATTAGCTATTTTTCT

ATCATCCTAATCTTTCTACCAATCGCAGGAATCATCGAAGACAAAATATTAAAATTATAT

>M-INA19

ATGACAAACATCCGAAAAATTCACCCCCTAATAAAAATAGTAAACCACTCATTCATTGAC

CTCCCTACTCCTCCAAACATCTCATCCTGATGAAACTTTGGCTCACTCCTAGGAATTTGC

TTAGTAATTCAAATTACTACAGGACTCTTCCTAGCCATACACTATACAGCAGATACAACA

ACAGCATTTTCATCAGTATCCCACATCTGCCGAGACGTAAATTACGGATGACTAATTCGT

TATATACACGCAAACGGAGCCTCAATATTCTTCATCTGCCTATTTATCCATATCGGACGA

GGAATTTACTACGGATCCTACATCTTTCAAGAAACATGAAACATCGGCGTAATCCTCCTA

TTTGCCGTAATAGCTACCGCATTCATGGGTTATGTCCTACCATGAGGACAAATATCCTTC

TGAGGCGCCACAGTCATTACAAACCTCCTCTCAGCAATCCCATACATTGGCCCAACAATT

GTAGAATGAATCTGAGGAGGCTTCTCAGTAGACAAAGCCACTCTAACACGATTCTTCGCA

TTCCACTTTATCCTTCCCTTTATTATTGCAGCTCTCGTCCTAGTCCACCTCTTATTTCTC

CACGAAACTGGATCCAATAACCCTCTAGGACTAAACTCCAACGCAGACAAAATCCCCTTT

CACCCCTATTATACAGTAAAAGATTTTCTCGGAGTAATCTTACTACTTCTATTCTTCACA

ATTTTAGTCCTCTTCTTCCCCGACCTACTTGGAGACCCAGACAATTACACACCCGCCAAC

CCACTTAATACTCCCCCTCATATTAAACCCGAATGATATTTCTTATTTGCCTACGCTATT

CTCCGCTCTATTCCCAATAAACTAGGTGGAGTACTAGCCCTAGTACTTTCAATCCTCATT

CTAATTTTCCTACCACTAATCCATACATCAAAACAACGAAGCCTAATATTCCGACCAATT

TCACAAATACTCTACTGAATTTTAATCGCCAACCTACTTATCCTCACATGAATTGGAGGT

CAACCAGTAGAACACCCATTTATCATTATTGGCCAACTAGCCTCAATTAGCTACTTCTCC

ATCATCCTAATCTTTCTACCAATCGCAGGGATCATCGAAGACAAAATATTAAAATGATAT

>M5939_M_INA2

ATGACAAACATTCGAAAAATTCACCCACTACTAAAAATAGTTAACCACTCATTCATTGAT

CTTCCCACTCCCCCTAATATTTCATCTTGATGAAACTTTGGCTCACTCCTAGGAATTTGC

TTAATAATTCAAATCGCTACAGGACTTTTCCTAGCCATACATTATACAGCAGACACAACA

ACAGCATTCTCATCAGTATCCCATATCTGCCGAGACGTCAATTATGGATGACTAATCCGC

TATATACATGCAAACGGAGCTTCAATATTCTTTATTTGCCTATTCATTCACATCGGACGA

GGAATTTACTACGGATCTTACATCTTTCAAGAAACATGAAACATTGGAGTAATCCTCTTA

TTTGCCGTAATAGCCACCGCATTCATAGGGTATGTACTTCCATGAGGACAAATATCCTTC

TGAGGGGCCACAGTCATTACAAATCTTCTTTCAGCTATTCCATATATTGGCCCAACAATC

GTAGAATGAATTTGAGGAGGATTTTCAGTGGACAAAGCCACTTTAACACGATTTTTCGCA

TTTCACTTCATTCTCCCCTTTATTATCACAGCCTTAGTCCTAGTCCATCTCCTATTCCTT

CACGAAACCGGATCTAATAATCCCCTAGGCCTTAACTCCAACTCAGACAAAATCCCTTTT

CACCCATACTACACAGTAAAAGATTTTCTCGGAGTAATTCTACTACTTCTATTTTTCACA

ATTTTAGTCCTCTTCTTCCCTGACTTACTTGGAGATCCAGACAACTACACACCCGCTAAC

CCCCTTAACACTCCCCCCCACATTAAACCCGAATGGTATTTCTTATTTGCCTATGCTATC

CTACGTTCAATTCCTAACAAACTAGGAGGAGTCCTAGCCCTAGTACTTTCAATCCTTATC

CTAATTTTTTTACCACTCATTCACACATCAAAACAACGAAGCCTAATATTCCGACCTATT

TCCCAAATACTTTACTGAATCTTAATTGCCAACCTACTTATCCTCACATGAATCGGAGGC

CAACCAGTAGAACACCCATTTATTATCATTGGCCAACTAGCCTCAATCAGTTACTTTTCC

ATTATCCTAATTCTTTTACCAATCGCAGGAATCATTGAAGATAATATATTAAAATGATAT

>M-TAD10

ATGACAAACATCCGAAAAATTCACCCCCTACTAAAAATAGTAAACCACTCATTCATCGAC

CTCCCCACTCCTCCAAACATCTCATCCTGATGAAACTTTGGCTCACTTCTAGGAATTTGC

TTAGTAATTCAAATTGCTACAGGACTTTTCCTAGCCATACATTATACAGCAGACACAACA

ACAGCATTTTCATCAGTATCCCATATCTGCCGAGACGTAAATTACGGATGACTAATCCGT

TACATACACGCAAACGGAGCCTCACTATTCTTCATCTGCCTATTTATCCATATCGGACGA

GGCATTTACTACGGATCCTACATCTTCCAAGAAACATGAAACATCGGTGTAATCCTCCTA

TTTGCCGTAATAGCTACCGCATTCATAGGGTACGTCCTACCATGAGGACAAATATCCTTC

TGAGGAGCCACAGTGATTACAAATCTCCTCTCAGCAATCCCGTATATTGGTCCAACAATT

GTAGAATGAATCTGAGGGGGGTTCTCAGTAGACAAAGCCACCCTAACACGATTTTTCGCA

TTCCATTTTATCCTCCCTTTTATTATTACAGCCCTTGTCCTAGTCCACCTCCTATTTCTT

CACGAAACCGGATCCAATAACCCCCTAGGACTAAACTCTAATGCAGACAAAATCCCCTTT

CACCCCTATTACACAGTAAAAGATTTTCTCGGAGTAATCTTACTACTTCTATTCTTCATA

ATCCTAGTTCTCTTCTTTCCTGACCTACTTGGAGACCCAGACAATTACACACCCGCTAAC

CCACTCAACACACCTCCCCATATTAAACCCGAATGATATTTCTTATTTGCCTACGCTATT

CTCCGTTCCATCCCCAATAAACTAGGTGGAGTACTAGCCTTAGTATTATCAATCCTCATT

CTAATTTTCCTACCACTAATCCATACATCAAAACAACGAAGCCTAATATTCCGACCAATT

TCACAAATACTTTACTGAATTTTAATCGCTAACCTGCTTATCCTTACATGAATCGGGGGC

CAACCAGTAGAACACCCATTTATCATTATTGGTCAACTAGCCTCAATTAGCTATTTTTCT

ATCATCCTAATCTTTCTACCAATCGCAGGAATCATCGAAGACAAAATATTAAAATTATAT

>M-TES11

ATGACAAACTTCCGAAAAACTCACCCCCTACTAAAAATAGTAAACCATTCATTCATTGAT

CTCCCCACTCCCCCCAACATCTCATCCTGATGAAACTTCGGCTCACTCCTAGGAATTTGC

TTAGTTATCCAAATCACCACAGGACTTTTCCTAGCTATACATTACACAGCAGACACAACA

ACAGCATTCTCATCAGTCTCACACATTTGCCGAGACGTAAATTACGGATGACTAATCCGC

TATATACACGCAAACGGAGCCTCAATATTTTTTATCTGTTTATTCATCCACATCGGACGA

GGGATTTATTATGGATCCTATATCTTTCAAGAAACATGAAACATCGGTGTAATTCTCTTA

TTCGCTGTAATAGCCACTGCATTTATAGGCTATGTTCTACCATGAGGACAAATATCTTTC

TGAGGAGCTACAGTAATCACTAATCTCCTCTCAGCGATCCCATACATTGGCCCAACAATT

GTAGAATGAATTTGAGGAGGTTTCTCAGTAGACAAAGCCACCCTAACACGATTCTTCGCA

TTCCACTTTATCCTCCCATTCATCATTGCAGCTCTTGTACTAGTCCATCTCTTGTTCCTA

CACGAGACCGGATCTAATAATCCCCTGGGAATCAACTCAAACGCAGACAAAATCCCTTTT

CACCCCTACTATACAGTAAAAGACTTCCTAGGGGTTATTATCTTTATTTTATTCTTCACA

ATAATAGTCCTATTTTTCCCAGACTTACTTGGAGACCCAGACAACTACACACCCGCCAAT

CCACTTAACACCCCTCCTCACATTAAACCCGAATGATACTTTCTATTTGCCTACGCCATC

CTACGCTCAATTCCCAACAAACTAGGAGGAGTCCTAGCCCTAGTACTTTCAATCCTCATC

CTAGCCCTCCTACCACTTATTCATACATCAAAACAACGAAGCCTGATATTCCGACCGATC

TCACAAATACTATACTGAATTTTAATCGCTAACCTACTTATCCTAACATGAATTGGAGGT

CAACCAGTAGAACACCCATTTATTATCATTGGCCAACTAGCTTCAATCAGTTACTTCTCT

ATTATCCTCATCCTACTTCCAATTGCAGGAATTATCGAAGACAAAATATTGAAATGATAT

>M5309

ATGACAAACTTCCGAAAAACTCACCCTCTACTAAAAATAGTAAACCATTCATTCATTGAT

CTCCCCACTCCCCCCAACATCTCATCCTGATGAAACTTCGGCTCACTCCTAGGAATTTGC

TTAGTTATCCAAATCACCACAGGACTTTTCCTAGCTATACATTACACAGCAGACACAACA

ACAGCATTCTCATCAGTCTCACACATTTGCCGAGACGTAAATTACGGATGACTAATCCGC

TATATACACGCAAACGGAGCCTCAATATTTTTATCTGTTTATTCATCCACATCGGACGA

GGGATTTATTATGGATCCTATATCTTTCAAGAAACATGAAACATCGGTGTAATTCTCTTA

TTCGCTGTAATAGCCACTGCATTTATAGGCTATGTTCTACCATGAGGACAAATATCTTTC

TGAGGAGCTACAGTAATCACTAATCTCCTCTCAGCGATCCCATACATTGGCCCAACAATT

GTAGAATGAATTTGAGGAGGTTTCTCAGTAGACAAAGCCACCCTAACACGATTCTTCGCA

TTCCACTTTATCCTCCCATTCATCATTGCAGCTCTTGTACTAGTCCATCTCTTGTTCCTA

CACGAGACCGGATCTAATAATCCCCTGGGAATCAACTCAAACGCAGACAAAATCCCTTTT

CACCCCTACTATACAGTAAAAGACTTCCTAGGGGTTATTATCTTTATTTTATTCTTCACA

ATAATAGTCCTATTTTTCCCAGACTTACTTGGAGATCCAGACAACTACACACCCGCCAAT

CCACTTAACACCCCTCCCCACATTAAACCCGAATGATACTTTCTATTTGCCTACGCCATC

CTACGCTCAATTCCCAACAAACTAGGAGGAGTCCTAGCCCTAGTACTTTCAATCCTCATC

CTAGCCCTCCTACCACTTATTCATACATCAAAACAACGAAGCCTAATATTCCGACCGATC

TCACAAATACTGTACTGAATTTTAATCGCTAACCTACTTATCCTAACATGAATTGGAGGT

CAACCAGTAGAACACCCATTTATCATCATTGGCCAACTAGCTTCAATCAGTTACTTCTCT

ATTATCCTCATCCTTCTTCCAATTGCAGGAATTATCGAAGACAAAATATTGAAATGATAT

>M5925

ATGACAAACTTCCGAAAAACTCACCCTCTACTAAAAATAGTAAACCATTCATTCATTGAT

CTCCCCACTCCCCCCAACATCTCATCCTGATGAAACTTCGGCTCACTCCTAGGAATCTGC

TTAGTTATTCAAATCACCACAGGACTTTTCCTAGCTATACATTACACAGCAGACACAACA

ACAGCATTCTCATCAGTCTCACACATTTGCCGAGACGTAAATTACGGATGACTAATCCGC

TATATACACGCAAACGGAGCCTCAATATTTTTATCTGTTTATTTATCCACATCGGACGA

GGGATTTATTATGGATCCTATATCTTTCAAGAAACATGAAACATCGGTGTAATTCTCTTA

TTCGCTGTAATAGCCACTGCATTTATAGGCTATGTTCTACCATGAGGACAAATATCTTTC

TGAGGAGCTACAGTAATCACTAATCTCCTCTCAGCAATCCCATACATTGGCCCAACAATT

GTAGAATGAATTTGAGGGGGTTTCTCAGTAGACAAAGCCACCCTAACACGATTCTTCGCA

TTCCACTTTATCCTCCCATTCATCATTGCAGCTCTTGTACTAGTTCATCTCTTGTTCCTA

CACGAGACCGGATCTAATAATCCCCTGGGAATCAACTCAAACGCAGACAAAATCCCTTTT

CACCCCTACTATACAGTAAAAGACTTCCTAGGGGTTATTATCTTTATTTTATTCTTCACA

ATAATAGTCCTATTTTTCCCAGACTTACTTGGAGACCCAGACAACTACACACCCGCCAAT

CCACTTAACACCCCTCCTCACATTAAACCCGAATGATACTTTCTATTTGCCTACGCCATC

CTACGCTCAATTCCCAACAAACTAGGAGGAGTCCTAGCCCTAGTACTTTCAATCCTCATC

CTAGCCCTCCTACCACTTATTCATACATCAAAACAACGAAGCCTAATATTCCGACCAATC

TCACAAATACTGTACTGAATTTTAATCGCTAACCTACTCATCCTAACATGAATTGGAGGT

CAACCAGTAGAACACCCATTTATCATCATTGGCCAACTAGCTTCAATCAGTTACTTCTCT

ATTATCCTCATCCTACTTCCAATTGCAGGAATTATCGAAGACAAAATATTGAAATGATAT

>M5929

ATGACAAACATCCGAAAAATTCACCCCCTACTAAAAATAGTAAACCACTCATTCATCGAC

CTCCCCACTCCTCCAAACATCTCATCCTGATGAAACTTTGGCTCACTTCTAGGAATTTGC

TTAGTAATTCAAATTGCTACAGGACTTTTCCTAGCCATACATTATACAGCAGACACAACA

ACAGCATTTTCATCAGTATCCCATATCTGCCGAGACGTAAATTACGGATGACTAATCCGT

TATATACACGCAAACGGAGCCTCACTATTCTTCATCTGCCTATTTATCCATATCGGACGA

GGCATTTACTACGGATCCTACATCTTCCAAGAAACATGAAACATCGGTGTAATCCTCCTA

TTTGCCGTAATAGCTACCGCATTCATAGGGTACGTCCTACCATGAGGACAAATATCCTTC

TGAGGAGCCACAGTGATTACAAATCTCCTCTCAGCAATCCCGTATATTGGTCCAACAATT

GTAGAATGAATCTGAGGGGGGTTCTCAGTAGACAAAGCCACCCTAACACGATTTTTCGCA

TTCCATTTTATTCTTCCTTTTATTATTACAGCCCTTGTCCTAGTCCACCTCCTATTTCTT

CACGAAACCGGATCTAATAACCCCCTAGGACTAAACTCTAATGCAGACAAAATCCCCTTT

CACCCCTATTACACAGTAAAAGATTTTCTCGGAGTAATTTTACTACTTCTATTCTTCATA

ATCCTAGTTCTCTTCTTTCCTGACCTACTTGGAGACCCAGACAATTACACACCCGCTAAC

CCACTCAACACACCTCCCCATATTAAACCCGAATGATATTTCTTATTTGCCTACGCTATT

CTCCGTTCCATCCCCAATAAACTAGGTGGAGTACTAGCCCTAGTATTATCAATCCTCATT

CTAATTTTCCTACCACTAATCCATACATCAAAACAACGAAGCCTAATATTCCGACCAATT
TCACAAATACTTTACTGAATTTTAATCGCTAACCTACTTATCCTTACATGAATCGGGGGC
CAACCAGTAGAACACCCATTTATCATTATTGGCCAACTAGCCTCAATTAGCTATTTTTCT
ATTATCCTAATCTTTCTACCAATCGCAGGAATCATCGAAGACAAAATATTAAAATTATAT
>M5931
ATGACAAACATCCGAAAAATTCACCCCCTAATAAAAATAGTAAACCACTCATTCATTGAC
CTCCCTACTCCTCCAAACATCTCATCCTGATGAAACTTTGGCTCACTCCTAGGAATTTGC
TTAGTAATTCAAATTACTACAGGACTCTTCCTAGCCATACACTATACAGCAGATACAACA
ACAGCATTTTCATCAGTATCCCACATCTGCCGAGACGTAAATTACGGATGACTAATTCGT
TATATACACGCAAACGGAGCCTCAATATTCTTCATCTGCCTATTTATCCATATCGGACGA
GGAATTTACTACGGATCCTACATCTTTCAAGAAACATGAAACATCGGTGTAATCCTCCTA
TTTGCCGTAATAGCTACCGCATTCATGGGTTACGTCCTACCATGAGGACAAATATCCTTC
TGAGGCGCCACAGTCATTACAAACCTCCTCTCAGCAATCCCATACATTGGCCCAACAATT
GTAGAATGAATCTGAGGAGGCTTCTCAGTAGACAAAGCCACTCTAACACGATTCTTCGCA
TTCCACTTTATCCTTCCCTTTATTATTACAGCTCTCGTCCTAGTCCACCTCTTATTTCTC
CACGAAACTGGATCCAATAACCCTCTAGGACTAAACTCCAACGCAGACAAAATCCCCTTT
CACCCCTATTATACAGTAAAAGATTTTCTCGGAGTAATCTTACTACTTCTATTCTTCACA
ATCTTAGTCCTCTTCTTTCCCGACCTACTTGGAGACCCAGACAATTACACACCCGCCAAC
CCACTTAATACTCCCCCTCATATTAAACCCGAATGATATTTCTTATTTGCCTACGCTATT
CTCCGCTCTATTCCCAATAAACTAGGTGGAGTACTAGCCCTAGTACTTTCAATCCTCATT
CTAATTTTCCTACCACTAATCCATACATCAAAACAACGAAGCCTAATATTCCGACCAATT
TCACAAATACTCTACTGAATTTTAATCGCCAACCTACTTATCCTCACATGAATTGGAGGT
CAACCAGTAGAACACCCATTTATCATTATTGGCCAACTAGCCTCAATTAGCTATTTCTCC
ATCATCCTAATCTTTCTACCAATCGCAGGGATCATCGAAGACAAAATATTAAAATGATAT
>M5932a /transl_table=2
ATGACAAACATCCGAAAAATTCACCCCCTACTAAAAATAGTAAACCACTCATTCATCGAC
CTCCCCACTCCTCCAAACATCTCATCCTGATGAAACTTTGGCTCACTTCTAGGAATTTGC
TTAGTAATTCAAATTGCTACAGGACTTTTCCTAGCCATACATTATACAGCAGACACAACA
ACAGCATTTTCATCAGTATCCCATATCTGCCGAGACGTAAATTACGGATGACTAATCCGT
TATATACACGCAAACGGAGCCTCACTATTCTTCATCTGCCTATTTATCCATATCGGACGA
GGCATTTACTACGGATCCTACATCTTCCAAGAAACATGAAACATCGGTGTAATCCTCCTA
TTTGCCGTAATAGCTACCGCATTCATAGGATACGTCCTACCATGAGGACAAATATCCTTC
TGAGGAGCCACAGTGATTACAAATCTCCTCTCAGCAATCCCATATATTGGCCCAACAATT
GTAGAATGAATCTGAGGGGGGTTCTCAGTAGACAAAGCCACCCTAACACGATTTTTCGCA
TTCCACTTTATCCTACCTTTTATTATTACAGCCCTTGTCCTAGTCCACCTCCTATTTCTT
CACGAAACCGGATCCAATAACCCCCTAGGACTAAACTCTAATGCAGACAAAATCCCCTTT
CACCCCTATTACACAGTAAAAGATTTTCTCGGAGTAATTTTACTACTTCTATTCTTCATA
ATCCTAGTTCTCTTCTTCCCTGACCTACTTG-----------------------------

>M5934
ATGACAAACATTCGAAAAATTCACCCACTACTAAAAATAGTTAACCACTCATTCATTGAT
CTTCCCACTCCCCCTAATATTTCATCTTGATGAAACTTTGGCTCACTCCTAGGAATTTGC
TTAATAATTCAAATCGCTACAGGACTTTTCCTAGCCATACATTATACAGCAGACACAACA
ACAGCATTCTCATCAGTATCCCATATCTGCCGAGACGTCAATTATGGATGACTAATCCGC
TATATACATGCAAACGGAGCTTCAATATTCTTTATTTGCCTATTCATTCACATCGGACGA
GGAATTTACTACGGATCTTATATCTTTCAAGAAACATGAAACATTGGAGTAATTCTCTTA

TTTGCCGTAATAGCCACCGCATTCATAGGGTATGTACTTCCATGAGGACAAATATCCTTC
TGAGGGGCCACAGTCATTACAAATCTTCTTTCAGCTATTCCATATATTGGCCCAACAATC
GTAGAATGAATTTGAGGAGGATTTTCAGTGGACAAAGCCACTTTAACACGATTTTTCGCA
TTTCACTTCATTCTCCCCTTTATTATCACAGCCTTAGTCCTAGTCCATCTCCTATTCCTT
CACGAAACCGGATCTAATAATCCCCTAGGCCTTAACTCCAACTCAGACAAAATCCCTTTT
CACCCATACTACACAGTAAAAGATTTTCTCGGAGTAATTCTACTACTCCTATTTTTCACA
ATTCTAGTCCTCTTCTTCCCTGACTTACTTGGAGATCCAGACAACTACACACCCGCTAAC
CCCCTTAACACTCCCCCCCACATTAAACCCGAATGGTATTTCTTATTTGCCTATGCTATC
CTACGTTCAATTCCTAACAAACTAGGAGGAGTCCTAGCCCTAGTACTTTCAATCCTTATC
CTAATTTTTTTACCACTCATTCACACATCAAAACAACGAAGCCTAATATTCCGACCTATT
TCCCAAATACTTTACTGAATCTTAATTGCCAACCTACTTATCCTCACATGAATCGGAGGC
CAACCAGTAGAACACCCATTTATTATCATTGGCCAACTAGCCTCAATCAGTTACTTTTCC
ATTATCCTAATTCTTTTACCAATCGCAGGAATCATTGAAGATAATATATTAAAATGATAT
>M5937
ATGACAAACATCCGAAAAATTCACCCCCTAATAAAAATACTAAACCACTCATTCATTGAC
CTCCCTACTCCTCCAAACATCTCATCCTGATGAAACTTTGGCTCACTCCTAGGAATTTGC
TTAGTAATTCAAATTACTACAGGACTCTTCCTAGCCATACACTATACAGCAGATACAACA
ACAGCATTTTCATCAGTATCCCACATCTGCCGAGACGTAAATTACGGATGACTAATTCGT
TATATACACGCAAACGGAGCCTCAATATTCTTCATCTGCCTATTTATCCATATCGGACGA
GGAATTTACTACGGATCCTACATCTTTCAAGAAACATGAAATATCGGCGTAATCCTCCTA
TTTGCCGTAATAGCTACCGCATTCATGGGTTATGTCCTACCATGAGGACAAATATCCTTC
TGAGGCGCCACAGTCATTACAAACCTCCTCTCAGCAATCCCATACATTGGCCCAACAATT
GTAGAATGAATCTGAGGAGGCTTCTCAGTAGACAAAGCCACTCTAACACGATTCTTCGCA
TTCCACTTTATCCTTCCCTTTATTATTGCAGCTCTCGTCCTAGTCCACCTCTTATTTCTC
CACGAAACTGGATCCAATAACCCTCTAGGACTAAACTCCAACGCAGACAAAATCCCCTTT
CACCCCTATTATACAGTAAAAGATTTTCTCGGAGTAATCTTACTACTCCTATTCTTCACA
ATTTTAGTCCTCTTCTTCCCCGACCTACTTGGAGACCCAGACAATTACACACCCGCCAAC
CCACTTAATACTCCCCCTCATATTAAACCCGAATGATATTTCTTATTTGCCTACGCTATT
CTCCGCTCTATTCCCAATAAACTAGGTGGAGTACTAGCCCTAGTACTTTCAATCCTCATT
CTAATTTTCCTACCACTAATCCATACATCAAAACAACGAAGCCTAATATTCCGACCAATT
TCACAAATACTCTACTGAATTTTAATCGCCAACCTACTTATCCTCACATGAATTGGAGGT
CAACCAGTAGAACACCCATTTATCATTATTGGCCAACTAGCCTCAATTAGCTATTTCTCC
ATCATCCTAATCTTTCTACCAATCGCAGGAATCATCGAAGACAAAATATTAAAATGATAT
>M5938
ATGACAAACATTCGAAAAATTCACCCACTACTAAAAATAGTTAACCACTCATTCATTGAT
CTTCCCACTCCCCCTAATATTTCATCTTGATGAAACTTTGGCTCACTCCTAGGAATTTGC
TTAATAATTCAAATCGCTACAGGACTTTTCCTAGCCATACATTATACAGCAGACACAACA
ACAGCATTCTCATCAGTATCCCATATCTGCCGAGACGTCAATTATGGATGACTAATCCGC
TATATACATGCAAACGGAGCTTCAATATTCTTTATTTGCCTATTCATTCACATCGGACGA
GGAATTTACTACGGATCTTATATCTTTCAAGAAACATGAAACATTGGAGTAATTCTCTTA
TTTGCCGTAATAGCCACCGCATTCATAGGGTATGTACTTCCATGAGGACAAATATCCTTC
TGAGGGGCCACAGTCATTACAAATCTTCTTTCAGCTATTCCATATATTGGCCCAACAATC
GTAGAATGAATTTGAGGAGGATTTTCAGTGGACAAAGCCACTTTAACACGATTTTTCGCA
TTTCACTTCATTCTCCCCTTTATTATCACAGCCTTAGTCCTAGTCCATCTCCTATTCCTT
CACGAAACCGGATCTAATAATCCCCTAGGCCTTAACTCCAACTCAGACAAAATCCCTTTT
CACCCATACTACACAGTAAAAGATTTTCTCGGAGTAATTCTACTACTTCTATTTTTCACA
ATTTTAGTCCTCTTCTTCCCTGACTTACTTGGAGATCCAGACAACTACACACCCGCTAAC
CCCCTTAACACTCCCCCCCACATTAAACCCGAATGGTATTTCTTATTTGCCTATGCTATT
CTACGTTCAATTCCTAACAAACTAGGAGGAGTCCTAGCCCTAGTACTTTCAATCCTTATC
CTAATTTTTTTACCACTCATTCACACATCAAAACAACGAAGCCTAATATTCCGACCTATT
TCCCAAATACTTTACTGAATCTTAATTGCCAACCTACTTATTCTCACATGAATCGGAGGC

CAACCAGTAGAACACCCATTTATTATCATTGGCCAACTAGCCTCAATCAGTTACTTTTCC
ATTATCCTGATTCTTTTACCAATCGCAGGAATTATTGAAGATAATATATTAAAATGATAT
>M5939_M_INA2
ATGACAAACATTCGAAAAATTCACCCACTACTAAAAATAGTTAACCACTCATTCATTGAT
CTTCCCACTCCCCCTAATATTTCATCTTGATGAAACTTTGGCTCACTCCTAGGAATTTGC
TTAATAATTCAAATCGCTACAGGACTTTTCCTAGCCATACATTATACAGCAGACACAACA
ACAGCATTCTCATCAGTATCCCATATCTGCCGAGACGTCAATTATGGATGACTAATCCGC
TATATACATGCAAACGGAGCTTCAATATTCTTTATTTGCCTATTCATTCACATCGGACGA
GGAATTTACTACGGATCTTACATCTTTCAAGAAACATGAAACATTGGAGTAATCCTCTTA
TTTGCCGTAATAGCCACCGCATTCATAGGGTATGTACTTCCATGAGGACAAATATCCTTC
TGAGGGGCCACAGTCATTACAAATCTTCTTTCAGCTATTCCATATATTGGCCCAACAATC
GTAGAATGAATTTGAGGAGGATTTTCAGTGGACAAAGCCACTTTAACACGATTTTTCGCA
TTTCACTTCATTCTCCCCTTTATTATCACAGCCTTAGTCCTAGTCCATCTCCTATTCCTT
CACGAAACCGGATCTAATAATCCCCTAGGCCTTAACTCCAACTCAGACAAAATCCCTTTT
CACCCATACTACACAGTAAAAGATTTTCTCGGAGTAATTCTACTACTTCTATTTTTCACA
ATTTTAGTCCTCTTCTTCCCTGACTTACTTGGAGATCCAGACAACTACACACCCGCTAAC
CCCCTTAACACTCCCCCCCACATTAAACCCGAATGGTATTTCTTATTTGCCTATGCTATC
CTACGTTCAATTCCTAACAAACTAGGAGGAGTCCTAGCCCTAGTACTTTCAATCCTTATC
CTAATTTTTTTACCACTCATTCACACATCAAAACAACGAAGCCTAATATTCCGACCTATT
TCCCAAATACTTTACTGAATCTTAATTGCCAACCTACTTATCCTCACATGAATCGGAGGC
CAACCAGTAGAACACCCATTTATTATCATTGGCCAACTAGCCTCAATCAGTTACTTTTCC
ATTATCCTAATTCTTTTACCAATCGCAGGAATCATTGAAGATAATATATTAAAATGATAT
>M5944
ATGACAAACATCCGAAAAATTCACCCCCTACTAAAAATAGTAAACCACTCATTCATCGAC
CTCCCCACTCCTCCAAACATCTCATCCTGATGAAACTTTGGCTCACTTCTAGGAATTTGC
TTAGTAATTCAAATTGCTACAGGACTTTTCCTAGCCATACATTATACAGCAGACACAACA
ACAGCATTTTCATCAGTATCCCATATCTGCCGAGACGTAAATTACGGATGACTAATCCGT
TATATACACGCAAACGGAGCCTCACTATTCTTCATCTGCCTATTCATCCATATCGGACGA
GGCATTTACTACGGATCCTACATCTTCCAAGAAACATGAAACATCGGTGTAATCCTCCTA
TTTGCCGTAATAGCTACCGCATTCATAGGATACGTCCTACCATGAGGACAAATATCCTTC
TGAGGAGCCACAGTGATTACAAATCTCCTCTCAGCAATCCCGTATATTGGTCCAACAATT
GTAGAATGAATCTGAGGGGGGTTCTCAGTAGACAAAGCCACCCTAACACGATTTTTCGCA
TTCCATTTTATCCTCCCTTTTATTATTACAGCCCTTGTCCTAGTCCACCTCCTATTTCTT
CACGAAACCGGATCCAATAACCCCCTAGGACTAAACTCTAATGCAGACAAAATCCCCTTT
CACCCCTATTACACAGTAAAAGATTTCCTCGGAGTAATTTTACTACTTTTATTCTTCATA
ATCCTAGTTCTCTTCTTTCCTGACCTACTTGGAGACCCAGACAATTACACACCTGCTAAC
CCACTCAACACACCTCCCCATATTAAACCCGAATGATATTTCTTATTTGCCTACGCTATT
CTCCGTTCCATCCCCAATAAACTAGGTGGAGTACTAGCCTTAGTATTATCAATCCTCATT
CTAATTTTCCTACCACTAATCCATACATCAAAACAACGAAGCCTAATATTCCGACCAATT
TCACAAATACTTTACTGAATTTTAATCGCTAACCTGCTTATCCTTACATGAATCGGGGGC
CAACCAGTAGAACACCCATTTATCATTATTGGCCAACTAGCCTCAATTAGTTATTTTTCT
ATCATCCTAATCTTTCTACCAATCGCAGGAATCATCGAAGACAAAATATTAAAATTATAT
>M5945
ATGACAAACATCCGAAAAATTCACCCCCTACTAAAAATAGTAAACCACTCATTCATCGAC
CTCCCCACTCCTCCAAACATCTCATCCTGATGAAACTTTGGCTCACTTCTAGGAATTTGC
TTAGTAATTCAAATTGCTACAGGACTTTTCCTAGCCATACATTATACAGCAGACACAACA
ACAGCATTTTCATCAGTATCCCATATCTGCCGAGACGTAAATTACGGATGACTAATCCGT
TATATACACGCAAACGGAGCCTCACTATTCTTCATCTGCCTATTTATCCATATCGGACGA
GGTATTTACTACGGATCCTACATCTTCCAAGAAACATGAAACATCGGTGTAATCCTCCTA
TTTGCCGTAATAGCTACCGCATTCATAGGGTACGTCCTACCATGAGGACAAATATCCTTC
TGAGGAGCCACAGTGATTACAAATCTCCTCTCAGCAATCCCATATATTGGCCCAACAATT

GTAGAATGGATCTGGGGGGGGTTCTCAGTAGACAAAGCCACCCTAACACGATTTTTCGCA
TTCCACTTTATCCTCCCTTTTATTATTACAGCCCTTGTCCTAGTCCACCTCCTATTTCTT
CACGAAACCGGATCCAATAACCCCCTAGGACTAAACTCTAATGCAGACAAAATCCCCTTT
CACCCCTATTATACAGTAAAAGATTTTCTCGGAGTAATTTTACTACTTCTATTCTTCATA
ATCCTAGTTCTCTTCTTTCCTGACCTACTTGGAGACCCAGACAATTACACACCTGCTAAC
CCACTCAACACACCTCCCCATATTAAACCCGAATGATATTTCTTATTTGCCTACGCTATT
CTCCGTTCCATCCCCAATAAACTAGGTGGAGTACTAGCCTTAGTATTATCAATCCTCATT
CTAATTTTCCTACCACTAATCCATACATCAAAACAACGAAGCCTAATATTCCGACCAATT
TCACAAATACTTTACTGAATTTTAATCGCTAACCTGCTTATCCTTACATGAATCGGGGGC
CAACCAGTAGAACACCCATTTATCATTATTGGCCAACTAGCCTCAATTAGCTATTTTTCT
ATCATCCTAATCTTTCTACCAATCGCAGGAATCATCGAAGACAAAATATTAAAATTATAT
>M5947
ATGACAAACATTCGAAAAATTCACCCACTACTAAAAATAGTTAACCACTCATTCATTGAT
CTTCCCACTCCCCCTAATATCTCATCTTGATGAAACTTTGGTTCACTTCTAGGAATTTGC
TTAATTATTCAAATCGCTACAGGACTTTTCCTAGCCATACATTATACAGCAGACACAACA
ACAGCATTCTCATCAGTATCCCATATCTGCCGAGACGTCAATTATGGATGACTAATCCGC
TATATACATGCAAACGGAGCTTCAATATTCTTTATTTGCCTATTCATTCACATTGGACGA
GGAATTTACTACGGATCTTACATCTTTCAAGAAACATGAAACATTGGAGTAATTCTCTTA
TTTGCCGTAATAGCCACCGCATTCATAGGATATGTACTTCCATGAGGACAAATATCCTTC
TGAGGGGCCACAGTCATTACAAATCTTCTTTCAGCTATTCCATATATTGGCCCAACAATC
GTAGAATGAATTTGAGGAGGATTTTCAGTGGACAAAGCCACTTTAACACGATTTTTCGCA
TTTCACTTCATTCTCCCCTTTATTATCACAGCCCTAGTCCTAGTCCATCTTCTATTCCTT
CACGAAACCGGATCTAATAATCCCCTAGGCCTTAACTCCAACTCAGACAAAATCCCTTTT
CACCCATACTACACAGTAAAAGATTTTCTCGGAGTAATTCTACTACTTCTATTTTTCACA
ATTTTAGTCCTCTTCTTCCCTGACTTACTTGGAGATCCAGACAACTACACACCCGCTAAC
CCCCTTAACACTCCCCCCCACATTAAACCCGAATGATATTTCTTATTTGCCTATGCTATC
CTACGTTCAATTCCTAACAAACTAGGAGGAGTCCTAGCCCTAGTACTTTCAATCCTTATC
CTAATTTTCTTACCACTCATTCACACATCAAAACAACGAAGCCTAATATTCCGACCTATT
TCCCAAATACTTTACTGAATCTTAATTGCCAACCTACTTATCCTCACATGAATCGGAGGC
CAACCAGTAGAACACCCATTTATTATCATTGGCCAACTAGCCTCAATCAGTTACTTTTCC
ATTATCCTAATTCTTTTACCAATCGCAGGAATCATTGAAGATAATATATTAAAATGATAT
>M5948
ATGACAAACATCCGAAAAATTCACCCCCTACTAAAAATAGTAAACCACTCATTCATCGAC
CTCCCCACTCCTCCAAACATCTCATCCTGATGAAACTTTGGCTCACTTCTAGGAATTTGC
TTAGTAATTCAAATTGCTACAGGACTTTTCCTAGCCATACATTATACAGCAGACACAACA
ACAGCATTTTCATCAGTATCCCATATCTGCCGAGACGTAAATTACGGATGACTAATCCGT
TATATACACGCAAACGGAGCCTCACTATTCTTCATCTGCTTATTTATCCATATCGGACGA
GGCATTTACTACGGATCCTACATCTTCCAAGAAACATGAAACATCGGTGTAATCCTCCTA
TTTGCCGTAATAGCTACCGCATTCATAGGGTACGTCCTACCATGAGGACAAATATCCTTC
TGAGGAGCCACAGTGATTACAAATCTCCTCTCAGCAATCCCATATATTGGCCCAACAATT
GTAGAATGAATCTGAGGAGGGTTCTCAGTAGACAAAGCCACCCTAACACGATTTTTCGCA
TTCCACTTTATCCTCCCTTTTATTATTACAGCCCTTGTCCTAGTCCACCTCCTATTTCTT
CACGAAACCGGATCCAATAACCCCCTAGGACTAAACTCTAATGCAGACAAAATCCCCTTT
CACCCCTATTACACAGTAAAAGATTTTCTCGGAGTAATTTTACTACTTCTATTCTTCATA
ATCCTAGTTCTCTTCTTTCCTGACCTACTTGGAGACCCAGACAATTACACACCTGCTAAC
CCACTCAACACACCTCCCCATATTAAACCCGAATGATATTTCTTATTTGCCTACGCTATT
CTCCGTTCCATCCCCAATAAACTAGGTGGAGTACTAGCCTTAGTATTATCAATCCTCATT
CTAATTTTCCTACCACTAATCCATACATCAAAACAACGAAGCCTAATATTCCGACCAATT
TCACAAATACTTTACTGAATTTTAATCGCTAACCTGCTTATCCTTACATGAATCGGGGGC
CAACCAGTAGAACACCCATTTATCATTATTGGCCAACTAGCCTCAATTAGCTATTTTTCT
ATCATTCTAATCTTTCTACCAATCGCAGGAATCATCGAAGACAAAATATTAAAATTATAT

>M5949

ATGACAAACATCCGAAAAATTCACCCCCTACTAAAAATAGTAAACCACTCATTCATCGAC

CTCCCCACTCCTCCAAACATCTCATCCTGATGAAACTTTGGCTCACTTCTAGGAATTTGC

TTAGTAATTCAAATTGCTACAGGACTTTTCCTAGCCATACATTATACAGCAGACACAACA

ACAGCATTTTCATCAGTATCCCATATCTGCCGAGACGTAAATTACGGATGACTAATCCGT

TATATACACGCAAACGGAGCCTCACTATTCTTCATCTGCCTATTTATCCATATCGGACGA

GGCATTTACTACGGATCCTACATCTTCCAAGAAACATGAAACATCGGTGTAATCCTCCTA

TTTGCCGTAATAGCTACCGCATTCATAGGGTACGTCCTACCATGAGGACAAATATCCTTC

TGAGGAGCCACAGTGATTACAAATCTCCTCTCAGCAATCCCGTATATTGGTCCAACAATT

GTAGAATGAATCTGAGGGGGGTTCTCAGTAGACAAAGCCACCCTAACACGATTTTTCGCA

TTCCATTTTATTCTTCCTTTTATTATTACAGCCCTTGTCCTAGTCCACCTCCTATTTCTT

CACGAAACCGGATCTAATAACCCCCTAGGACTAAACTCTAATGCAGACAAAATCCCCTTT

CACCCCTATTACACAGTAAAAGATTTTCTCGGAGTAATTTTACTACTTCTATTCTTCATA

ATCCTAGTTCTCTTCTTTCCTGACCTACTTGGAGACCCAGACAATTACACACCCGCTAAC

CCACTCAACACACCTCCCCATATTAAACCCGAATGATATTTCTTATTTGCCTACGCTATT

CTCCGTTCCATCCCCAATAAACTAGGTGGAGTACTAGCCCTAGTATTATCAATCCTCATT

CTAATTTTCCTACCACTAATCCATACATCAAAACAACGAAGCCTAATATTCCGACCAATT

TCACAAATACTTTACTGAATTTTAATCGCTAACCTACTTATCCTTACATGAATCGGGGGC

CAACCAGTAGAACACCCATTTATCATTATTGGCCAACTAGCCTCAATTAGCTATTTTTCT

ATTATCCTAATCTTTCTACCAATCGCAGGAATCATCGAAGACAAAATATTAAAATTATAT

>M5950

ATGACAAACATCCGAAAAATTCACCCCCTACTAAAAATAGTAAACCACTCATTCATCGAC

CTCCCCACTCCTCCAAACATCTCATCCTGATGAAACTTTGGCTCACTTCTAGGAATTTGC

TTAGTAATTCAAATTGCTACAGGACTTTTCCTAGCCATACATTATACAGCAGACACAACA

ACAGCATTTTCATCAGTATCCCATATCTGCCGAGACGTAAATTACGGATGACTAATCCGT

TATATACACGCAAACGGAGCCTCACTATTCTTCATCTGCCTATTTATCCATATCGGACGA

GGCATTTACTACGGATCCTACATCTTCCAAGAAACATGAAACATCGGTGTAATCCTCCTA

TTTGCCGTAATAGCTACCGCATTCATAGGGTACGTCCTACCATGAGGACAAATATCCTTC

TGAGGAGCCACAGTGATTACAAATCTCCTCTCAGCAATCCCGTATATTGGTCCAACAATT

GTAGAATGAATCTGAGGGGGGTTCTCAGTAGACAAAGCCACCCTAACACGATTTTTCGCA

TTCCATTTTATCCTTCCTTTTATTATTACAGCCCTTGTCCTAGTCCACCTCCTATTTCTT

CACGAAACCGGATCCAATAACCCCCTAGGACTAAACTCTAATGCAGACAAAATCCCCTTT

CACCCCTATTACACAGTAAAAGATTTTCTCGGAGTAATTTTACTACTTCTATTCTTCATA

ATCCTAGTTCTCTTCTTTCCTGACCTACTTGGAGACCCAGACAATTACACACCCGCTAAC

CCACTCAACACACCTCCCCATATTAAACCCGAATGATACTTCTTATTTGCCTACGCTATT

CTCCGTTCCATCCCCAATAAACTAGGTGGAGTACTAGCCTTAGTATTATCAATCCTCATT

CTAATTTTCCTACCACTAATCCATACATCAAAACAACGAAGCCTAATATTCCGACCAATT

TCACAAATACTTTACTGAATTTTAATCGCTAACCTACTTATCCTTACATGAATCGGGGGC

CAACCAGTAGAACACCCATTTATCATTATTGGCCAACTAGCCTCAATTAGCTATTTTTCT

ATTATCCTAATCTTTCTACCAATCGCAGGAATCATCGAAGACAAAATATTAAAATTATAT

>M5952

ATGACAAACATCCGAAAAATTCACCCCCTAATAAAAATAGTAAACCACTCATTCATTGAC

CTCCCTACTCCTCCAAACATCTCATCCTGATGAAACTTTGGCTCACTCCTAGGAATTTGC

TTAGTAATTCAAATTACTACAGGACTCTTCCTAGCCATACACTATACAGCAGATACAACA

ACAGCATTCTCATCAGTATCCCACATCTGCCGAGACGTAAATTACGGATGACTAATTCGT

TATATACACGCAAACGGAGCCTCAATATTCTTCATCTGCCTATTTATCCATATCGGACGA

GGAATTTACTACGGATCCTACATCTTTCAAGAAACATGAAACATCGGCGTAATCCTCCTA

TTCGCCGTAATAGCTACCGCATTCATGGGTTATGTCCTACCATGAGGACAAATATCCTTC

TGAGGCGCCACAGTCATTACAAACCTCCTCTCAGCAATCCCATACATTGGCCCAACAATT

GTAGAATGAATCTGAGGAGGCTTCTCAGTAGACAAAGCCACTCTAACACGATTCTTCGCA

TTCCACTTTATCCTTCCCTTTATTATTGCAGCTCTCGTCCTAGTCCACCTCTTATTTCTC

CACGAAACTGGATCCAATAACCCTCTAGGACTAAACTCCAACGCAGACAAAATCCCCTTT

CACCCCTATTATACAGTAAAAGATTTTCTCGGAGCAATCTTACTACTTCTATTCTTCACA

ATTTTAGTCCTCTTCTTCCCCGACCTACTTGGAGACCCAGACAATTACACACCCGCCAAC

CCACTTAATACTCCCCCTCATATTAAACCCGAATGATATTTCTTATTTGCCTACGCTATT

CTCCGCTCTATTCCCAATAAACTAGGTGGAGTACTAGCCCTAGTACTTTCAATCCTCATT

CTAATTTTCCTACCACTAATCCATACATCAAAACAACGAAGCCTAATATTCCGACCAATT

TCACAAATACTCTACTGAATTTTAATCGCCAACCTACTTATCCTCACATGAATTGGAGGT

CAACCAGTAGAACACCCATTTATCATTATTGGCCAACTAGCCTCAATTAGCTATTTCTCC

ATCATCCTAATCTTTCTACCAATCGCAGGGATCATCGAAGACAAAATATTAAAATGATAT

>M5953

ATGACAAACATTCGAAAAATTCACCCACTACTAAAAATAGTTAACCACTCATTCATTGAT

CTTCCCACTCCCCCTAATATTTCATCTTGATGAAACTTTGGCTCACTCCTAGGAATTTGC

TTAATAATTCAAATCGCTACAGGACTTTTCCTAGCCATACATTATACAGCAGACACAACA

ACAGCATTCTCATCAGTATCCCATATCTGCCGAGACGTCAATTATGGATGACTAATCCGC

TATATACATGCAAACGGAGCTTCAATATTCTTTATTTGCCTATTCATTCACATCGGACGA

GGAATTTACTACGGATCTTATATCTTTCAAGAAACATGAAACATTGGAGTAATTCTCTTA

TTTGCCGTAATAGCCACCGCATTCATAGGGTATGTACTTCCATGAGGACAAATATCCTTC

TGAGGGGCCACAGTCATTACAAATCTTCTTTCAGCTATTCCATATATTGGCCCAACAATC

GTAGAATGAATTTGAGGAGGATTTTCAGTGGACAAAGCCACTTTAACACGATTTTTCGCA

TTTCACTTCATTCTCCCCTTTATCATCACAGCCTTAGTCCTAGTCCATCTCCTATTCCTT

CACGAAACCGGATCTAATAATCCCCTAGGCCTTAACTCCAACTCAGACAAAATCCCTTTT

CACCCATACTACACAGTAAAAGATTTTCTCGGAGTAATTCTACTACTCCTATTTTTCACA

ATTTTAGTCCTCTTCTTCCCTGACTTACTTGGAGATCCAGACAACTACACACCCGCTAAC

CCCCTTAACACTCCCCCCCACATTAAACCCGAATGATATTTCTTATTTGCCTATGCTATC

CTACGTTCAATTCCTAACAAACTAGGAGGAGTCCTAGCCCTAGTACTTTCAATCCTTATC

CTAATTTTTTTACCACTCATTCACACATCAAAACAACGAAGCCTAATATTCCGACCTATT

TCCCAAATACTTTACTGAATCTTAATTGCCAACCTACTTATCCTCACATGAATCGGAGGC

CAACCAGTAGAACACCCATTTATTATCATTGGCCAACTAGCCTCAATCAGTTACTTTTCC

ATTATCCTAATTCTTTTACCAATCGCAGGAATCATTGAAGATAATATATTAAAATGATAT

>M5957

ATGACAAACATTCGAAAAATTCACCCACTACTAAAAATAGTTAACCACTCATTCATTGAT

CTTCCCACTCCCCCTAATATTTCATCTTGATGAAACTTTGGCTCACTCCTAGGAATTTGC

TTAATAATTCAAATCGCTACAGGACTTTTCCTAGCCATACATTATACAGCAGACACAACA

ACAGCATTCTCATCAGTATCCCATATCTGCCGAGACGTCAATTATGGATGACTAATCCGC

TATATACATGCAAACGGAGCTTCAATATTCTTTATTTGCCTATTCATTCACATCGGACGA

GGAATTTACTACGGATCTTATATCTTTCAAGAAACATGAAACATTGGAGTAATTCTCTTA

TTTGCCGTAATAGCCACCGCATTCATAGGGTATGTACTTCCATGAGGACAAATATCCTTT

TGAGGGGCCACAGTCATTACAAATCTTCTTTCAGCTATTCCATATATTGGCCCAACAATC

GTAGAATGAATTTGAGGAGGATTTTCAGTGGACAAAGCCACTTTAACACGATTTTTCGCA

TTTCACTTCATTCTCCCCTTTATTATCACAGCCTTAGTCCTAGTCCATCTCCTATTCCTT

CACGAAACCGGATCTAATAATCCCCTAGGCCTTAACTCCAACTCAGACAAAATCCCTTTT

CACCCATACTACACAGTAAAAGATTTTCTCGGAGTAATTCTACTACTTCTATTTTTCACA

ATTTTAGTCCTCTTCTTCCCTGACTTACTTGGAGATCCAGACAACTACACACCCGCTAAC

CCCCTTAACACTCCCCCCCACATTAAACCCGAATGATATTTCTTATTTGCCTATGCTATC

CTACGTTCAATTCCTAACAAACTAGGAGGAGTCCTAGCCCTAGTACTTTCAATCCTTATC

CTAATTTTTTTACCACTCATTCACACATCAAAACAACGAAGCCTAATATTCCGACCTATT

TCCCAAATACTTTACTGAATCTTAATTGCCAACCTACTTATCCTCACATGAATCGGAGGC

CAACCAGTAGAACACCCATTTATTATCATTGGCCAACTAGCCTCAATCAGTTACTTTTCC

ATTATCCTAATTCTTTTACCAATCGCAGGAATCATTGAAGATAATATATTAAAATGATAT

>M5958

ATGACAAACATTCGAAAAATTCACCCACTACTAAAAATAGTTAACCACTCATTCATTGAT

CTTCCCACTCCCCCTAATATTTCATCTTGATGAAACTTTGGCTCACTCCTAGGAATTTGC
TTAATAATTCAAATCGCTACAGGACTTTTCCTAGCCATACATTATACAGCAGACACAACA
ACAGCATTTTCATCAGTATCCCATATCTGCCGAGACGTCAATTATGGATGACTAATCCGC
TATATACATGCAAACGGAGCTTCAATATTCTTTATTTGCCTATTCATTCACATCGGACGA
GGAATTTACTACGGATCTTATATCTTTCAAGAAACATGAAACATTGGGGTAATTCTCTTA
TTTGCCGTAATAGCCACCGCATTCATAGGGTATGTACTTCCATGAGGACAAATATCCTTC
TGAGGGGCCACAGTCATTACAAATCTTCTTTCAGCTATTCCATATATTGGCCCAACAATC
GTAGAGTGAATTTGAGGAGGATTTTCAGTGGACAAAGCCACTTTAACACGATTTTTCGCA
TTTCACTTCATTCTCCCCTTTATTATCACAGCCTTAGTCCTAGTCCATCTCCTATTCCTT
CACGAAACCGGATCTAATAATCCCCTAGGCCTTAACTCCAACTCAGACAAAATCCCTTTT
CACCCATACTACACAGTAAAAGATTTTCTCGGAGTAATTCTACTACTTCTATTTTTCACA
ATTTTAGTCCTCTTCTTCCCTGACTTACTTGGAGATCCAGACAACTACACACCCGCTAAC
CCCCTTAACACTCCCCCCCACATTAAACCCGAATGATATTTCTTATTTGCCTATGCTATC
CTACGTTCAATTCCTAACAAACTAGGAGGAGTCCTAGCCCTAGTACTTTCAATCCTTATC
CTAATTTTTTTACCACTCATTCACACATCAAAACAACGAAGCCTAATATTCCGACCTATT
TCCCAAATACTTTACTGAATCTTAATTGCCAACCTACTTATCCTCACATGAATCGGAGGC
CAACCAGTAGAACACCCATTTATTATCATTGGCCAACTAGCCTCAATCAGTTACTTTTCC
ATTATCCTAATCCTTTTACCAATCGCAGGAATCATTGAAGATAATATATTAAAATGATAT
>M5960
ATGACAAACTTCCGAAAAACTCACCCTCTACTAAAAATAGTAAACCATTCATTCATTGAT
CTCCCCACTCCCCCCAACATCTCATCCTGATGAAACTTCGGCTCACTCCTAGGAATTTGC
TTAGTTATCCAAATCACCACAGGACTTTTCCTAGCTATACATTACACAGCAGACACAACA
ACAGCATTCTCATCAGTCTCACACATTTGCCGAGACGTAAATTACGGATGACTAATCCGC
TATATACACGCAAACGGAGCCTCAATATTTTTTATCTGTTTATTCATCCACATCGGACGA
GGAATTTATTATGGATCCTATATCTTTCAAGAAACATGAAACATCGGTGTAATTCTCTTA
TTCGCTGTAATAGCCACTGCATTTATAGGCTATGTTCTACCATGAGGACAAATATCTTTC
TGAGGAGCCACAGTAATCACTAATCTCCTCTCAGCAATCCCATACATTGGCCCAACAATT
GTAGAATGAATTTGAGGAGGTTTCTCAGTAGACAAAGCCACCCTAACACGATTCTTCGCA
TTCCACTTTATCCTCCCATTCATCATTGCAGCTCTTGTACTAGTCCACCTCTTGTTCCTA
CANGAGACCGGATCTAATAATCCCCTGGGAATCAACTCAAACGCAGACAAAATCCCTTTT
CACCCCTACTATACAGTAAAAGACTTCCTAGGGGTTATTATCTTTATTTTATTCTTCACA
ATAATAGTCCTATTTTTCCCAGACTTACTTGGAGACCCAGACAACTACACACCCGCCAAT
CCACTTAACACCCCTCCTCACATTAAACCCGAATGATACTTTCTATTTGCCTACGCCATC
CTACGCTCAATTCCCAACAAACTAGGAGGAGTCCTAGCCCTAGTACTTTCAATCCTCATC
CTAGCCCTCCTACCACTTATTCATACATCAAAACAACGAAGCCTAATATTCCGACCAATC
TCACAAATACTGTACTGAATTTTAATCGCTAACCTACTCATCCTAACATGAATTGGAGGT
CAACCAGTAGAACACCCATTTATCATCATTGGCCAACTAGCTTCAATCAGTTACTTCTCT
ATTATCCTCATCCTACTTCCAATTGCAGGAATTATCGAAGACAAAATATTGAAATGATAT
>M5962
ATGACAAACATCCGAAAAATTCACCCCCTACTAAAAATAGTAAACCACTCATTCATCGAC
CTCCCCACTCCTCCAAACATCTCATCCTGATGAAACTTTGGCTCACTTCTAGGAATTTGC
TTAGTAATTCAAATTGCTACAGGACTTTTCCTAGCCATACATTATACAGCAGACACAACA
ACAGCATTTTCATCAGTATCCCATATCTGCCGAGACGTAAATTACGGATGACTAATCCGT
TATATACACGCAAACGGAGCCTCACTATTCTTCATCTGCCTATTTATCCATATCGGACGA
GGCATTTACTACGGATCCTACATCTTCCAAGAAACATGAAACATCGGTGTAATCCTCCTA
TTTGCCGTAATAGCTACCGCATTCATAGGGTACGTCCTACCATGAGGACAAATATCCTTC
TGAGGAGCCACAGTGATTACAAATCTCCTCTCAGCAATCCCATATATTGGCCCAACAATT
GTAGAATGAATCTGAGGGGGATTCTCAGTAGACAAAGCCACCCTAACACGATTTTTCGCA
TTCCACTTTATCCTCCCTTTTATTATTACAGCCCTTGTCCTAGTCCACCTCCTATTTCTT
CACGAAACCGGATCCAATAACCCCCTAGGACTAAACTCTAATGCAGACAAAATCCCCTTT
CACCCCTATTACACAGTAAAAGATTTTCTCGGAGTAATTTTACTACTTCTATTCTTCATA

ATCCTAGTTCTCTTCTTTCCTGACCTACTTGGAGACCCAGACAATTACACACCTGCTAAC
CCACTCAACACACCTCCCCATATTAAACCCGAATGATATTTCTTATTTGCCTACGCTATT
CTCCGTTCCATCCCCAATAAACTAGGTGGAGTACTAGCCTTAGTATTATCAATCCTCATT
CTAATTTTCCTACCACTAATCCATACATCAAAACAACGAAGCCTAATATTCCGACCAATT
TCACAAATACTTTACTGAATTTTAATCGCTAACCTGCTTATCCTTACATGAATCGGGGGC
CAACCAGTAGAACACCCATTTATCATTATTGGCCAACTAGCCTCAATTAGCTATTTTTCT
ATCATCCTAATCTTTCTACCAATCGCAGGAATCATCGAAGACAAAATATTAAAATTATAT
>M5965
ATGACAAACTTCCGAAAAACTCACCCTCTACTAAAAATAGTAAACCATTCATTCATTGAT
CTCCCCACTCCCCCCAACATCTCATCCTGATGAAACTTCGGCTCACTCCTAGGAATTTGC
TTAGTTATTCAAATCACCACAGGACTTTTTCTAGCCATACATTACACAGCAGACACAACA
ACAGCATTCTCATCAGTCTCACACATTTGCCGAGACGTAAATTACGGATGACTAATCCGC
TATATACACGCAAACGGAGCCTCAATATTTTTTATCTGTTTATTCATCCACATCGGACGA
GGAATTTATTATGGATCCTATATCTTTCAAGAAACATGAAACATCGGTGTAATTCTCTTA
TTCGCTGTAATAGCCACTGCATTTATAGGCTATGTTCTACCATGAGGACAAATATCTTTC
TGAGGAGCTACAGTAATTACTAATCTCCTCTCAGCAATCCCATACATTGGCCCAACAATT
GTAGAATGAATTTGAGGGGGTTTCTCAGTAGATAAAGCCACCCTAACACGATTCTTCGCA
TTCCACTTTATCCTCCCATTCATCATTGCAGCTCTTGTACTAGTCCATCTCTTGTTCCTA
CACGAAACCGGATCTAATAATCCCCTGGGAATCAACTCAAACGCAGACAAAATCCCTTTT
CACCCCTACTATACAGTAAAAGACTTCCTAGGGGTTATTATCTTTATTTTATTCTTCACA
ATAATAGTCCTATTTTTCCCAGACTTACTTGGAGATCCAGACAACTACACACCCGCCAAT
CCACTTAACACCCCTCCCCACATTAAACCCGAATGATACTTTCTATTTGCCTACGCCATC
CTACGCTCAATTCCCAACAAACTAGGAGGAGTCCTAGCCCTAGTACTTTCAATCCTCATC
CTAGCCCTCCTACCACTTATTCATACATCAAAACAACGAAGCCTAATATTCCGACCGATC
TCACAAATACTGTACTGAATTTTAATCGCTAACCTACTTATCCTAACATGAATTGGAGGT
CAACCAGTAGAACACCCATTTATCATCATTGGCCAACTAGCTTCAATCAGTTACTTCTCT
ATTATCCTCATCCTTCTTCCAATTGCAGGAATTATCGAAGACAAAATATTGAAATGATAT
>M5966a. 1166 nucleotides.
ATGACAAACTTCCGAAAAACTCACCCTCTACTAAAAATAGTAAACCATTCATTCATTGAT
CTCCCCACTCCCCCCAACATCTCATCCTGATGAAACTTCGGCTCACTCCTAGGAATCTGC
TTAGTTATTCAAATCACCACAGGACTTTTCCTAGCTATACATTACACAGCAGACACAACA
ACAGCATTCTCATCAGTCTCACACATTTGCCGAGACGTAAATTACGGATGACTAATCCGC
TATATACACGCAAACGGAGCCTCAATATTTTTTATCTGTTTATTTATCCACATCGGACGA
GGGATTTATTATGGATCCTATATCTTTCAAGAAACATGAAACATCGGTGTAATTCTCTTA
TTCGCTGTAATAGCCACTGCATTTATAGGCTATGTTCTACCATGAGGACAAATATCTTTC
TGAGGGGCTACAGTAATCACTAATCTCCTCTCAGCAATCCCATACATTGGCCCAACAATT
GTAGAATGAATTTGAGGGGGTTTCTCAGTAGACAAAGCCACCCTAACACGATTCTTCGCA
TTCCACTTTATCCTCCCATTCATCATTGCAGCTCTTGTACTAGTTCATCTCTTGTTCCTA
CACGAGACCGGATCTAATAATCCCCTGGGAATCAACTCAAACGCAGACAAAATCCCTTTT
CACCCCTACTATACAGTAAAAGACTTCCTAGGGGTTAATATCTTTATTTTATTCTTCACA
ACAATAGTCCTATTTTTCCCAGACTTACTTGGAGACC-----------------------

>M5970
ATGACAAACATTCGAAAAATTCACCCACTACTAAAAATAGTTAACCACTCATTCATTGAT
CTTCCCACTCCCCCTAATATCTCATCTTGATGAAACTTTGGTTCACTTCTAGGAATTTGC
TTAATTATTCAAATTGCTACAGGACTTTTCCTAGCCATACATTATACAGCAGACACAACA

ACAGCATTCTCATCAGTATCCCATATCTGCCGAGACGTCAATTATGGATGACTAATCCGC
TATATACATGCAAACGGAGCTTCAATATTCTTTATTTGCCTATTCATTCACATTGGACGA
GGAATTTACTACGGATCTTACATCTTTCAAGAAACATGAAACATTGGAGTAATTCTCTTA
TTTGCCGTAATAGCCACCGCATTCATAGGATATGTACTTCCATGAGGACAAATATCCTTC
TGAGGGGCCACAGTCATTACAAATCTTCTTTCAGCTATTCCATATATTGGCCCAACAATC
GTAGAATGAATTTGAGGAGGATTTTCAGTGGACAAAGCCACTTTAACACGATTTTTCGCA
TTCCACTTCATTCTCCCCTTCATTATCACAGCCCTAGTCCTAGTCCATCTTCTATTCCTT
CACGAAACCGGATCTAATAATCCCCTAGGCCTTAACTCCAACTCAGACAAAATCCCTTTT
CACCCATACTACACAGTAAAAGACTTTCTCGGAGTAATTCTACTACTTCTATTTTTCACA
ATTTTAGTCCTCTTCTTCCCTGACTTACTTGGAGATCCAGACAACTACACACCCGCTAAC
CCCCTTAACACTCCCCCCCACATTAAACCCGAATGATATTTCTTATTTGCCTATGCTATC
CTACGTTCAATTCCTAACAAACTAGGAGGAGTCCTAGCCCTAGTACTTTCAATCCTTATC
CTAATTTTCTTACCACTCATTCACACATCAAAACAACGAAGCCTAATATTCCGACCTATT
TCCCAAATACTTTACTGAATCTTAATTGCCAACCTACTTATCCTCACATGAATCGGAGGC
CAACCAGTAGAACACCCATTTATTATCATTGGCCAACTAGCCTCAATCAGTTACTTTTCC
ATTATCCTAATTCTTTTACCAATCGCAGGAATCATTGAAGATAATATATTAAAATGATAT
>M5971
ATGACAAACATCCGAAAAATTCACCCCCTACTAAAAATAGTAAACCACTCATTCATCGAC
CTCCCCACTCCTCCAAACATCTCATCCTGATGAAACTTTGGCTCACTTCTAGGAATTTGC
TTAGTAATTCAAATTGCTACAGGACTTTTCCTAGCCATACATTATACAGCAGACACAACA
ACAGCATTTTCATCAGTATCCCATATCTGCCGAGACGTAAATTACGGATGACTAATCCGT
TATATACACGCAAACGGAGCCTCACTATTCTTCATCTGCCTATTCATCCATATCGGACGA
GGTATTTACTACGGATCCTACATCTTCCAAGAAACATGAAACATCGGTGTAATCCTCCTA
TTTGCCGTAATAGCTACCGCATTCATAGGATACGTCCTACCATGAGGACAAATATCCTTC
TGAGGAGCCACAGTGATTACAAATCTCCTCTCAGCAATCCCGTATATTGGTCCAACAATT
GTAGAATGAATCTGAGGGGGGTTCTCAGTAGACAAAGCCACCCTAACACGATTTTTCGCA
TTCCATTTTATCCTCCCTTTTATTATTACAGCCCTTGTCCTAGTCCACCTCCTATTTCTT
CACGAAACCGGATCCAATAACCCCCTAGGACTAAACTCTAATGCAGACAAAATCCCCTTT
CACCCCTATTACACAGTAAAAGATTTTCTCGGAGTAATTTTACTACTTCTATCCTTCATA
ATCCTAGTTCTCTTCTTTCCTGACCTACTTGGAGACCCAGACAATTACACACCTGCTAAC
CCACTCAACACACCTCCCCATATTAAACCCGAATGATATTTCTTATTTGCCTACGCTATT
CTCCGTTCCATCCCCAATAAACTAGGTGGAGTACTAGCCTTAGTATTATCAATCCTCATT
CTAATTTTCCTACCACTAATCCATACATCAAAACAACGAAGCCTAATATTCCGACCAATT
TCACAAATACTTTACTGAATTTTAATCGCTAACCTGCTTATCCTTACATGAATCGGGGGC
CAACCAGTAGAACACCCATTTATCATTATTGGCCAACTAGCCTCAATTAGCTATTTTTCT
ATCATCCTAATCTTTCTACCAATCGCAGGAATCATCGAAGACAAAATATTAAAATTATAT
>M5973
ATGACAAACTTCCGAAAAACTCACCCTCTACTAAAAATAGTAAACCATTCATTCATTGAT
CTCCCCACTCCCCCCAACATCTCATCCTGATGAAACTTCGGCTCACTCCTAGGAATTTGC
TTAGTTATTCAAATCACCACAGGACTTTTCCTAGCCATACATTACACAGCAGACACAACA
ACAGCATTCTCATCAGTCTCACACATTTGCCGAGACGTAAATTACGGATGACTAATCCGC
TATATACACGCAAACGGAGCCTCAATATTTTTTATCTGTTTATTCATCCACATCGGACGA
GGAATTTATTATGGATCCTATATCTTTCAAGAAACATGAAACATCGGTGTAATTCTCTTA
TTCGCTGTAATAGCCACTGCATTTATAGGCTATGTTCTACCATGAGGACAAATATCTTTC
TGAGGAGCTACAGTAATTACTAATCTCCTCTCAGCAATCCCATACATTGGCCCAACAATT
GTAGAATGAATTTGAGGGGGTTTCTCAGTAGATAAAGCCACCCTAACACGATTCTTCGCA
TTCCACTTTATCCTCCCATTCATCATTGCAGCTCTTGTACTAGTCCATCTCTTGTTCCTA
CACGAGACCGGATCTAATAATCCCCTGGGAATCAACTCAAACGCAGACAAAATCCCTTTT
CACCCCTACTATACAGTAAAAGACTTCCTAGGGGTTATTATCTTTATTTTATTCTTCACA
ATAATAGTCCTATTTTTTCCAGACTTACTTGGAGATCCAGACAACTACACACCCGCCAAT
CCACTTAACACCCCTCCCCACATTAAACCCGAATGATACTTTCTATTTGCCTACGCCATC

CTACGCTCAATTCCCAACAAACTAGGAGGAGTCCTAGCCCTAGTACTTTCAATCCTCATC

CTAGCCCTCCTACCACTTATTCATACATCAAAACAACGAAGCCTAATATTCCGACCGATC

TCACAAATACTGTACTGAATTTTAATCGCTAACCTACTTATCCTAACATGAATTGGAGGT

CAACCAGTAGAACACCCATTTATCATCATTGGCCAACTAGCTTCAATCAGTTACTTCTCT

ATTATCCTCATCCTTCTTCCAATTGCAGGAATTATCGAAGACAAAATATTGAAATGATAT

>M5979

ATGACAAACATCCGAAAAATTCACCCCCTACTAAAAATAGTAAACCACTCATTCATCGAC

CTCCCCACTCCTCCAAACATCTCATCCTGATGAAACTTTGGCTCACTTCTAGGAATTTGC

TTAGTAATTCAAATTGCTACAGGACTTTTCCTAGCCATACATTATACAGCAGACACAACA

ACAGCATTTTCATCAGTATCCCATATCTGCCGAGACGTAAATTACGGATGACTAATCCGT

TATATACACGCAAACGGAGCCTCACTATTCTTCATCTGCCTATTCATCCATATCGGACGA

GGCATTTACTACGGATCCTACATCTTCCAAGAAACATGAAACATCGGTGTAATCCTCCTA

TTTGCCGTAATAGCTACCGCATTCATAGGATACGTCCTACCATGAGGACAAATATCCTTC

TGAGGAGCCACAGTGATTACAAATCTCCTCTCAGCAATCCCATACATTGGCCCAACAATT

GTAGAATGAATCTGAGGAGGCTTCTCAGTAGACAAAGCCACTCTAACACGATTCTTCGCA

TTCCACTTTATCCTTCCCTTTATTATTACAGCCCTCGTCCTAGTCCACCTCCTATTTCTC

CACGAAACCGGATCCAATAACCCCCTAGGACTAAACTCCAACGCAGACAAAATCCCCTTT

CACCCCTACTACACAGTAAAAGATTTTCTCGGAGTAATCTTACTACTTCTATTCTTCACA

ATTTTAGTCCTCTTCTTCCCCGACCTACTTGGAGACCCAGACAATTACACACCCGCCAAC

CCACTTAATACTCCCCCTCATATTAAACCCGAATGATATTTCTTATTTGCCTACGCTATT

CTCCGCTCTATTCCCAATAAACTAGGTGGAGTACTAGCCCTAGTACTTTCAATCCTCATT

CTAATTTTCCTACCACTAATCCATACATCAAAACAACGAAGCCTAATATTCCGACCAATT

TCACAAATACTCTACTGAATTTTAATCGCCAACCTACTTATCCTCACATGAATTGGAGGT

CAACCAGTAGAACACCCATTTATCATTATTGGCCAACTAGCCTCAATTAGCTATTTCTCC

ATCATCCTAATCTTTCTACCAATCGCAGGGATCATCGAAGACAAAATATTAAAATGATAT

>M5980

ATGACAAACTTCCGAAAAACTCACCCTCTACTAAAAATAGTAAACCATTCATTCATTGAT

CTCCCCACTCCCCCCAACATCTCATCCTGATGAAACTTCGGCTCACTCCTAGGAATCTGC

TTAGTTATTCAAATCACCACAGGACTTTTCCTAGCTATACATTACACAGCAGACACAACA

ACAGCATTCTCATCAGTCTCACACATTTGCCGAGACGTAAATTACGGATGACTAATCCGC

TATATACACGCAAACGGAGCCTCAATATTTTTATCTGTTTATTTATCCACATCGGACGA

GGGATTTATTATGGATCCTATATCTTTCAAGAAACATGAAACATCGGTGTAATTCTCTTA

TTCGCTGTAATAGCCACTGCATTTATAGGCTATGTTCTACCATGAGGACAAATATCTTTC

TGAGGAGCTACAGTAATTACTAATCTCCTCTCAGCAATCCCATACATTGGCCCAACAATT

GTAGAATGAATTTGAGGGGGTTTCTCAGTAGACAAAGCCACCCTAACACGATTCTTCGCA

TTCCACTTTATCCTCCCATTCATCATTGCAGCTCTTGTACTAGTCCATCTCTTGTTCCTA

CACGAGACCGGATCTAATAATCCCCTGGGAATCAACTCAAACGCAGACAAAATCCCTTTT

CACCCCTACTATACAGTAAAAGACTTCCTAGGGGTTATTATCTTTATTTTATTCTTCACA

ATAATAGTCCTATTTTTCCCAGACTTACTTGGAGATCCAGACAACTACACACCCGCCAAT

CCACTTAACACCCCTCCTCACATTAAACCCGAATGATACTTTCTATTTGCCTACGCCATC

CTACGCTCAATTCCCAACAAACTAGGAGGAGTCCTAGCCCTAGTACTTTCAATCCTCATC

CTAGCCCTCCTACCACTTATTCATACATCAAAACAACGAAGCCTAATATTCCGACCGATC

TCACAAATACTGTACTGAATTTTAATCGCTAACCTACTTATCCTAACATGAATTGGAGGT

CAACCAGTAGAACACCCATTTATCATCATTGGCCAACTAGCTTCAATCAGTTACTTCTCT

ATTATCCTCATCCTACTTCCAATTGCAGGAATTATCGAAGACAAAATATTGAAATGATAT

>M5985

ATGACAAACATTCGAAAAATTCACCCACTACTAAAAATAGTTAACCACTCATTCATTGAT

CTTCCCACTCCCCCTAATATTTCATCTTGATGAAACTTTGGCTCACTCCTAGGAATTTGC

TTAATAATTCAAATCGCTACAGGACTTTTCCTAGCCATACATTATACAGCAGACACAACA

ACAGCATTCTCATCAGTATCCCATATCTGCCGAGACGTCAATTATGGATGACTAATCCGC

TATATACATGCAAACGGAGCTTCAATATTCTTTATTTGCCTATTCATTCACATCGGACGA

GGAATTTACTACGGATCTTATATCTTTCAAGAAACATGAAACATTGGAGTAATTCTCTTA
TTTGCCGTAATAGCCACCGCATTCATAGGGTATGTACTTCCATGAGGACAAATATCCTTC
TGAGGGGCCACAGTCATTACAAATCTTCTTTCAGCTATTCCATATATTGGCCCAACAATC
GTAGAATGAATTTGAGGAGGATTTTCAGTGGACAAAGCCACTTTAACACGATTTTTCGCA
TTTCACTTCATTCTCCCCTTTATTATCACAGCCTTAGTCCTAGTCCATCTCCTATTCCTT
CACGAAACCGGATCTAATAATCCCCTAGGCCTTAACTCCAACTCAGACAAAATCCCTTTT
CACCCATACTACACAGTAAAAGATTTTCTCGGAGTAATTCTACTACTCCTATTTTTCACA
ATTTTAGTCCTCTTCTTCCCTGACTTACTTGGAGATCCAGACAACTACACACCCGCTAAC
CCCCTTAACACTCCCCCCCACATTAAACCCGAATGGTATTTCTTATTTGCCTATGCTATC
CTACGTTCAATTCCTAACAAACTAGGAGGAGTCCTAGCCCTAGTACTTTCAATCCTTATC
CTAATTTTTTTACCACTCATTCACACATCAAAACAACGAAGCCTAATATTCCGACCTATT
TCCCAAATACTTTACTGAATCTTAATTGCCAACCTACTTATCCTCACATGAATCGGAGGC
CAACCAGTAGAACACCCATTTATTATCATTGGCCAACTAGCCTCAATCAGTTACTTTTCC
ATTATCCTAATTCTTTTACCAATCGCAGGAATCATTGAAGATAATATATTAAAATGATAT
>LG102
ATGACAAACATCCGAAAAATTCACCCCCTACTAAAAATAGTAAACCACTCATTCATCGAC
CTCCCCACTCCTCCAAACATCTCATCCTGATGAAACTTTGGCTCACTTCTAGGAATTTGC
TTAGTAATTCAAATCGCTACAGGACTTTTCCTAGCCATACATTATACAGCAGACACAACA
ACAGCATTTTCATCAGTATCCCATATCTGCCGAGACGTAAATTACGGATGACTAATTCGT
TATATACACGCAAACGGAGCCTCACTATTCTTCATCTGCCTATTTATCCATATCGGACGA
GGCATTTACTACGGATCCTACATCTTCCAAGAAACATGAAACATCGGTGTAATCCTCCTA
TTTGCCGTAATAGCTACCGCATTCATAGGGTACGTCCTACCATGAGGACAAATATCCTTC
TGAGGAGCCACAGTGATTACAAATCTCCTCTCAGCAATCCCATATATTGGTCCAACAATT
GTAGAATGAATCTGAGGGGGATTCTCAGTAGACAAAGCCACCCTAACACGATTTTTCGCA
TTCCATTTTATCCTCCCTTTTATTATTACAGCCCTCGTCCTAGTCCACCTCCTATTTCTC
CACGAAACCGGATCCAATAACCCCCTAGGACTAAACTCTAATGCAGACAAAATCCCCTTT
CACCCCTATTACACAGTAAAAGATTTTCTCGGAGTAATTTTACTACTTCTATTTTTCATA
ATCCTAGTTCTCTTCTTTCCTGACTTACTTGGAGACCCAGACAATTACACACCTGCTAAC
CCACTCAACACACCTCCCCATATTAAACCCGAATGATATTTCTTATTTGCCTACGCTATT
CTCCGTTCCATCCCCAATAAACTAGGTGGAGTACTAGCCTTAGTATTATCAATCCTCATT
CTAATTTTTCTACCACTAATCCACACATCAAAACAACGAAGCCTAATATTCCGACCAATT
TCACAAATACTTTACTGAATTTTAATCGCTAACCTGCTTATCCTTACATGAATCGGAGGC
CAACCAGTAGAACACCCGTTTATCATTATTGGCCAACTAGCCTCAATTAGCTATTTTTCT
ATCATCCTAATCTTTCTACCAATCGCAGGAATCATCGAAGACAAAATATTAAAATGATAT
>N3008
ATGACAAACATCCGAAAAATTCACCCCCTAATAAAAATAGTAAACCACTCATTCATTGAC
CTCCCTACTCCTCCAAACATCTCATCCTGATGAAACTTTGGCTCACTCCTAGGAATTTGC
TTAGTAATTCAAATTACTACAGGACTCTTCCTAGCCATACACTATACAGCAGATACAACA
ACAGCATTTTCATCAGTATCCCACATCTGCCGAGACGTAAATTACGGATGACTAATTCGT
TATATACACGCAAACGGAGCCTCAATATTCTTCATCTGCCTATTTATCCATATCGGACGA
GGGATTTACTACGGATCCTACATCTTTCAAGAAACATGAAACATCGGCGTAATCCTCCTA
TTTGCCGTAATAGCTACCGCATTCATAGGTTATGTCCTACCATGAGGACAAATATCCTTC
TGAGGCGCCACAGTCATTACAAACCTCCTCTCAGCAATCCCATACATTGGCCCAACAATT
GTAGAATGAATCTGAGGAGGCTTCTCAGTAGACAAAGCCACTCTAACACGATTCTTCGCA
TTCCACTTTATCCTTCCCTTTATTATTGCAGCTCTCGTCCTAGTCCACCTCTTATTTCTC
CACGAAACTGGATCCAATAACCCTCTAGGACTAAACTCCAACGCAGACAAAATCCCCTTT
CACCCCTATTATACAGTAAAAGATTTTCTCGGAGTAATCTTACTACTTCTATTCTTCACA
ATTTTAGTCCTCTTCTTCCCCGACCTACTTGGAGACCCAGACAATTACACACCCGCCAAC
CCACTTAATACTCCCCCTCATATTAAACCCGAATGATATTTCTTATTTGCCTACGCTATT
CTCCGCTCTATTCCCAATAAACTAGGTGGAGTACTAGCCCTAGTACTTTCAATCCTCATT
CTAATTTTCCTACCACTAATCCATACATCAAAACAACGAAGCCTAATATTCCGACCAATT

TCACAAATACTCTACTGAATTTTAATCGCCAACCTACTTATCCTCACATGAATTGGAGGT

CAACCAGTAGAACACCCATTTATCATTATTGGCCAACTAGCCTCAATTAGCTATTTCTCC

ATCATCCTAATCTTTCTACCAATCGCAGGGATCATCGAAGACAAAATATTAAAATGATAT

>N3013

ATGACAAACATCCGAAAAATTCACCCCCTACTAAAAATAGTAAACCACTCATTCATCGAC

CTCCCCACTCCTCCAAACATCTCATCCTGATGAAACTTTGGCTCACTTCTAGGAATTTGC

TTAGTAATTCAAATTGCTACAGGACTTTTCCTAGCCATACATTATACAGCAGACACAACA

ACAGCATTTTCATCAGTATCCCATATCTGCCGAGACGTAAATTACGGATGACTAATCCGT

TATATACACGCAAACGGAGCCTCACTATTCTTCATCTGCCTATTTATCCATATCGGACGA

GGCATTTACTACGGATCCTACATCTTCCAAGAAACATGAAACATCGGTGTAATCCTCCTA

TTTGCCGTAATAGCTACCGCATTCATAGGGTACGTCCTACCATGAGGACAAATATCCTTC

TGAGGAGCCACAGTGATTACAAATCTCCTCTCAGCAATCCCATATATTGGCCCAACAATT

GTAGAATGAATCTGAGGAGGGTTCTCAGTAGACAAAGCCACCCTAACACGATTTTTCGCA

TTCCACTTTATCCTCCCTTTTATTATTACAGCCCTTGTCCTAGTCCACCTCCTATTTCTT

CACGAAACCGGATCCAATAACCCCCTAGGACTAAACTCTAATGCAGACAAAATCCCCTTT

CACCCCTATTACACAGTAAAAGATTTTCTCGGAGTAATTTTACTACTTCTATTCTTCATA

ATCCTAGTTCTCTTCTTTCCTGACCTACTTGGAGACCCAGACAATTACACACCTGCTAAC

CCACTCAACACACCTCCCCATATTAAACCCGAATGATATTTCTTATTTGCCTACGCTATT

CTCCGTTCCATCCCCAATAAACTAGGTGGAGTACTAGCCTTAATATTATCAATCCTCATT

CTAATTTTCCTACCACTAATCCATACATCAAAACAACGAAGCCTAATATTCCGACCAATT

TCACAAATACTTTACTGAATTCTAATCGCTAACCTGCTTATCCTTACATGAATCGGGGGC

CAACCAGTAGAACACCCATTTATCATTATTGGCCAACTAGCCTCAATTAGCTATTTTTCT

ATCATTCTAATCTTTCTACCAATCGCAGGAATCATCGAAGACAAAATATTAAAATTATAT

>N3015

ATGACAAACATCCGAAAAATTCACCCCCTAATAAAAATAGTAAACCACTCATTCATTGAC

CTCCCTACTCCTCCAAACATCTCATCCTGATGAAACTTTGGCTCACTCCTAGGAATTTGC

TTAGTAATTCAAATTACTACAGGACTCTTCCTAGCCATACACTATACAGCAGATACAACA

ACAGCATTTTCATCAGTATCCCACATCTGCCGAGACGTAAATTACGGATGACTAATTCGT

TATATACACGCAAACGGAGCCTCAATATTCTTCATCTGCCTATTTATCCATATCGGACGA

GGAATTTACTACGGATCCTACATCTTTCAAGAAACATGAAACATCGGCGTAATCCTCCTA

TTTGCCGTAATAGCTACCGCATTCATGGGTTATGTCCTACCATGAGGACAAATATCCTTC

TGAGGCGCCACAGTCATTACAAACCTCCTCTCAGCAATCCCATACATTGGCCCAACAATT

GTAGAATGAATCTGAGGAGGCTTCTCAGTAGACAAAGCCACTCTAACACGATTCTTCGCA

TTCCACTTTATCCTTCCCTTTATTATTGCAGCTCTCGTCCTAGTCCACCTCTTATTTCTC

CACGAAACTGGATCCAATAACCCTCTAGGACTAAACTCCAACGCAGACAAAATCCCCTTT

CACCCCTATTATACAGTAAAAGATTTTCTCGGAGTAATCTTACTACTTCTATTCTTCACA

ATTTTAGTCCTCTTCTTCCCCGACCTACTTGGAGACCCAGACAATTACACACCCGCCAAC

CCACTTAATACTCCCCCTCATATTAAACCCGAATGATATTTCTTATTTGCCTACGCTATT

CTCCGCTCTATTCCCAATAAACTAGGTGGAGTACTAGCCCTAGTACTTTCAATCCTCATT

CTAATTTTCCTACCACTAACCCATACATCAAAACAACGAAGCCTAATATTCCGACCAATT

TCACAAATACTCTACTGAATTTTAATCGCCAACCTACTTATCCTCACATGAATTGGAGGT

CAACCAGTAGAACACCCATTTATCATTATTGGCCAACTAGCCTCAATTAGCTATTTCTCC

ATCATCCTAATCTTTCTACCAATCGCAGGGATCATCGAAGACAAAATATTAAAATGATAT

>N3016

ATGACAAACATCCGAAAAATTCACCCCCTACTAAAAATAGTAAACCACTCATTCATCGAC

CTCCCCACTCCTCCAAACATCTCATCCTGATGAAACTTTGGCTCACTTCTAGGAATTTGC

TTAGTAATTCAAATTGCTACAGGACTTTTCCTAGCCATACATTATACAGCAGACACAACA

ACAGCATTTTCATCAGTATCCCATATCTGCCGAGACGTAAATTACGGATGACTAATCCGT

TATATACACGCAAACGGAGCCTCACTATTCTTCATCTGCCTATTTATCCATATCGGACGA

GGCATTTACTACGGATCCTACATCTTCCAAGAAACATGAAACATCGGTGTAATCCTCCTA

TTTGCCGTAATAGCTACCGCATTCATAGGGTACGTCCTACCATGAGGACAAATATCCTTC

TGAGGAGCCACAGTGATTACAAATCTCCTCTCAGCAATCCCATATATTGGCCCAACAATT

GTAGAATGAATCTGAGGGGGATTCTCAGTAGACAAAGCCACCCTAACACGATTTTTCGCA

TTCCACTTTATCCTCCCTTTTATTATTACAGCCCTTGTCCTAGTCCACCTCCTATTTCTT

CACGAAACCGGATCCAATAACCCCCTAGGACTAAACTCTAATGCAGACAAAATCCCCTTT

CACCCCTATTACACAGTAAAAGATTTTCTCGGAGTAATTTTACTACTTCTATTCTTCATA

ATCCTAGTTCTCTTCTTTCCTGACCTACTTGGAGACCCAGACAATTACACACCTGCTAAC

CCACTCAACACACCTCCCCATATTAAACCCGAATGATATTTCTTATTTGCCTACGCTATT

CTCCGTTCCATCCCCAATAAACTAGGTGGAGTACTAGCCTTAGTATTATCAATCCTCATT

CTAATTTTCCTACCACTAATCCATACATCAAAACAACGAAGCCTAATATTCCGACCAATT

TCACAAATACTTTACTGAATTTTAATCGCTAACCTGCTTATCCTTACATGAATCGGGGGC

CAACCAGTAGAACACCCATTTATCATTATTGGCCAACTAGCCTCAATTAGCTATTTTTCT

ATCATCCTAATCTTTCTACCAATCGCAGGAATCATCGAAGACAAAATATTAAAATTATAT

>N3029

ATGACAAACATCCGAAAAATTCACCCCCTACTAAAAATAGTAAACCACTCATTCATCGAC

CTCCCCACTCCTCCAAACATCTCATCCTGATGAAACTTTGGCTCACTTCTAGGAATTTGC

TTAGTAATTCAAATTGCTACAGGACTTTTCCTAGCCATACATTATACAGCAGACACAACA

ACAGCATTTTCATCAGTATCCCATATCTGCCGAGACGTAAATTACGGATGACTAATCCGT

TATATACACGCAAACGGAGCCTCACTATTCTTCATCTGCCTATTTATCCATATCGGACGA

GGCATTTACTACGGATCCTACATCTTCCAAGAAACATGAAACATCGGTGTAATCCTCCTA

TTTGCCGTAATAGCTACCGCATTCATAGGGTACGTCCTACCATGAGGACAAATATCCTTC

TGAGGAGCCACAGTGATTACAAATCTCCTCTCAGCAATCCCATATATTGGCCCAACAATT

GTAGAATGAATCTGAGGAGGGTTCTCAGTAGACAAAGCCACCCTAACACGATTTTTCGCA

TTCCACTTTATCCTCCCTTTTATTATTACAGCCCTTGTCCTAGTCCACCTCCTATTTCTT

CACGAAACCGGATCCAATAACCCCCTAGGACTAAACTCTAATGCAGACAAAATCCCCTTT

CACCCCTATTACACAGTAAAAGATTTTCTCGGAGTAATTTTACTACTTCTATTCTTCATA

ATCCTAGTTCTCTTCTTTCCTGACCTACTTGGAGACCCAGACAATTACACACCTGCTAAC

CCACTCAACACACCTCCCCATATTAAACCCGAATGATATTTCTTATTTGCCTACGCTATT

CTCCGTTCCATCCCCAATAAACTAGGTGGAGTACTAGCCTTAATATTATCAATCCTCATT

CTAATTTTCCTACCACTAATCCATACATCAAAACAACGAAGCCTAATATTCCGACCAATT

TCACAAATACTTTACTGAATTCTAATCGCTAACCTGCTTATCCTTACATGAATCGGGGGC

CAACCAGTAGAACACCCATTTATCATTATTGGCCAACTAGCCTCAATTAGCTATTTTTCT

ATCATTCTAATCTTTCTACCAATCGCAGGAATCATCGAAGACAAAATATTAAAATTATAT

>N3035

ATGACAAACATTCGAAAAATTCACCCACTACTAAAAATAGTTAACCACTCATTCATTGAT

CTTCCCACTCCCCCTAATATTTCATCTTGATGAAACTTTGGCTCACTCCTAGGAATTTGC

TTAATAATTCAAATCGCTACAGGACTTTTCCTAGCCATACATTATACAGCAGACACAACA

ACAGCATTCTCATCAGTATCCCATATCTGCCGAGACGTCAATTATGGATGACTAATCCGC

TATATACATGCAAACGGAGCTTCAATATTCTTTATTTGCCTATTCATTCACATCGGACGA

GGAATTTACTACGGATCTTATATCTTTCAAGAAACATGAAACATTGGAGTAATTCTCTTA

TTTGCCGTAATAGCCACCGCATTCATAGGGTATGTACTTCCATGAGGACAAATATCCTTC

TGAGGGGCCACAGTCATTACAAATCTTCTTTCAGCTATTCCATATATTGGCCCAACAATC

GTAGAATGAATTTGAGGAGGATTTTCAGTGGACAAAGCCACTTTAACACGATTTTTCGCA

TTTCACTTCATTCTCCCCTTTATTATCACAGCCTTAGTCCTAGTCCATCTCCTATTCCTT

CACGAAACCGGATCTAATAATCCCCTAGGCCTTAACTCCAACTCAGACAAAATCCCTTTT

CACCCATACTACACAGTAAAAGATTTTCTCGGAGTAATTCTACTACTTCTATTTTTCACA

ATTTTAGTCCTCTTCTTCCCTGACTTACTTGGAGATCCAGACAACTACACACCCGCTAAC

CCCCTTAACACTCCCCCCCACATTAAACCCGAATGGTATTTCTTATTTGCCTATGCTATC

CTACGTTCAATTCCTAACAAACTAGGAGGAGTCCTAGCCCTAGTACTTTCAATCCTTATC

CTAATTTTTTTACCACTCATTCACACATCAAAACAACGAAGCCTAATATTCCGACCTATT

TCCCAAATACTTTACTGAATCTTAATTGCCAACCTACTTATCCTCACATGAATCGGAGGC

CAACCAGTAGAACACCCATTTATTATCATTGGCCAACTAGCCTCAATCAGTTACTTTTCC

ATTATCCTAATTCTTTTACCAATCGCAGGAATCATTGAAGATAATATATTAAAATGATAT

>N3036

ATGACAAACATCCGAAAAATTCACCCCCTAATAAAAATAGTAAACCACTCATTCATTGAC

CTCCCTACTCCTCCAAACATCTCATCCTGATGAAACTTTGGCTCACTCCTAGGAATTTGC

TTAGTAATTCAAATTACTACAGGACTCTTCCTAGCCATACACTATACAGCAGATACAACA

ACAGCATTTTCATCAGTATCCCACATCTGCCGAGACGTAAATTACGGATGACTAATTCGT

TATATACACGCAAACGGAGCCTCAATATTCTTCATCTGCCTATTTATCCATATCGGACGA

GGAATTTACTACGGATCCTACATCTTTCAAGAAACATGAAACATCGGTGTAATCCTCCTA

TTTGCCGTAATAGCTACCGCATTCATGGGTTACGTCCTACCATGAGGACAAATATCCTTC

TGAGGCGCCACAGTCATTACAAACCTCCTCTCAGCAATCCCATACATTGGCCCAACAATT

GTAGAATGAATCTGAGGAGGCTTCTCAGTAGACAAAGCCACTCTAACACGATTCTTCGCA

TTCCATTTTATCCTTCCCTTTATTATTACAGCTCTCGTCCTAGTCCACCTCTTATTTCTC

CACGAAACTGGATCCAATAACCCTCTAGGACTAAACTCCAACGCAGACAAAATCCCCTTT

CACCCCTATTATACAGTAAAAGATTTTCTCGGAGTAATCTTACTACTTCTATTCTTCACA

ATTTTAGTCCTCTTCTTCCCCGACCTACTTGGAGACCCAGACAATTACACACCCGCCAAC

CCACTTAATACTCCCCCTCATATTAAACCCGAATGATATTTCTTATTTGCCTACGCTATT

CTCCGCTCTATTCCCAATAAACTAGGTGGAGTACTAGCCCTAGTACTTTCAATCCTCATT

CTAATTTTCCTACCACTAATCCATACATCAAAACAACGAAGCCTAATATTCCGACCAATT

TCACAAATACTCTACTGAATTTTAATCGCCAACCTACTTATCCTCACATGAATTGGAGGT

CAACCAGTAGAACACCCATTTATCATTATTGGCCAACTAGCCTCAATTAGCTATTTCTCC

ATCATCCTAATCTTTCTACCAATCGCAGGGATCATCGAAGACAAAATATTAAAATGATAT

>N3039

ATGACAAACATCCGAAAAATTCACCCCCTAATAAAAATAGTAAACCACTCATTCATTGAC

CTCCCTACTCCTCCAAACATCTCATCCTGATGAAACTTTGGCTCACTCCTAGGAATTTGC

TTAGTAATTCAAATTACTACAGGACTCTTCCTAGCCATACACTATACAGCAGATACAACA

ACAGCATTTTCATCAGTATCCCACATCTGCCGAGACGTAAATTACGGATGACTAATTCGT

TATATACACGCAAACGGAGCCTCAATATTCTTCATCTGCCTATTTATCCATATCGGACGA

GGGATTTACTACGGATCCTACATCTTTCAAGAAACATGAAACATCGGCGTAATCCTCCTA

TTTGCCGTAATAGCTACCGCATTCATAGGTTATGTCCTACCATGAGGACAAATATCCTTC

TGAGGCGCCCCAGTCATTACAAACCTCCTCTCAGCAATCCCATACATTGGCCCAACAATT

GTAGAATGAATCTGAGGAGGCTTCTCAGTAGACAAAGCCACTCTAACACGATTCTTCGCA

TTCCACTTTATCCTTCCCTTTATTATTGCAGCTCTCGTCCTAGTCCACCTCTTATTTCTC

CACGAAACTGGATCCAATAACCCTCTAGGACTAAACTCCAACGCAGACAAAATCCCCTTT

CACCCCTATTATACAGTAAAAGATTTTCTCGGAGTAATCTTACTACTTCTATTCTTCACA

ATTTTAGTCCTCTTCTTCCCCGACCTACTTGGAGACCCAGACAATTACACACCCGCCAAC

CCACTTAATACTCCCCCTCATATTAAACCCGAATGATATTTCTTATTTGCCTACGCTATT

CTCCGCTCTATTCCCAATAAACTAGGTGGAGTACTAGCCCTAGTACTTTCAATCCTCATT

CTAATTTTCCTACCACTAATCCATACATCAAAACAACGAAGCCTAATATTCCGACCAATT

TCACAAATACTCTACTGAATTTTAATCGCCAACCTACTTATCCTCACATGAATTGGAGGT

CAACCAGTAGAACACCCATTTATCATTATTGGCCAACTAGCCTCAATTAGCTATTTCTCC

ATCATCCTAATCTTTCTACCAATCGCAGGGATCATCGAAGACAAAATATTAAAATGATAT

>N3040

ATGACAAACATCCGAAAAATTCACCCCTTACTAAAAATAGTAAACCACTCATTCATCGAC

CTCCCCACTCCTCCAAACATCTCATCCTGATGAAACTTTGGCTCACTTCTAGGAATTTGC

TTAGTAATTCAAATTGCTACAGGACTTTTCCTAGCCATACATTATACAGCAGACACAACA

ACAGCATTTTCATCAGTATCCCATATCTGCCGAGACGTAAATTACGGATGACTAATCCGT

TATATACACGCAAACGGAGCCTCACTATTCTTCATCTGCCTATTTATCCATATCGGACGA

GGCATTTACTACGGATCCTACATCTTCCAAGAAACATGAAACATCGGTGTAATCCTCCTA

TTTGCCGTAATAGCTACCGCATTCATAGGATACGTCCTGCCATGAGGACAAATATCCTTC

TGAGGAGCCACAGTGATTACAAATCTCCTCTCAGCAATCCCGTATATTGGTCCAACAATT

GTAGAATGAATCTGAGGAGGGTTCTCAGTAGACAAAGCCACCCTAACACGATTTTTCGCA

TTCCATTTTATCCTCCCTTTTATTATTACAGCTCTTGTCCTAGTCCACCTCCTATTTCTT
CACGAAACCGGATCCAATAACCCCCTAGGACTAAACTCTAATGCAGACAAAATCCCCTTT
CACCCCTATTACACAGTAAAAGATTTTCTCGGAGTAATTTTACTACTTCTATTCTTCATA
ATCCTAGTTCTCTTCTTTCCTGACCTACTTGGAGACCCAGACAATTACACACCTGCTAAC
CCACTCAACACACCTCCCCATATTAAACCCGAATGATATTTCTTATTTGCCTACGCTATT
CTCCGTTCCATCCCCAATAAACTAGGTGGAGTACTAGCCTTAGTATTATCAATCCTCATT
CTAATTTTCCTACCACTAATCCATACATCAAAACAACGAAGCCTAATATTCCGACCAATT
TCACAAATACTTTACTGAATTTTAATCGCTAACCTGTTTATCCTTACATGAATCGGAGGC
CAACCAGTAGAACACCCATTTATCATTATTGGCCAACTAGCCTCAATTAGCTACTTTTCT
ATCATCCTAATCTTTCTACCAATCGCAGGAATCATCGAAGACAAAATATTAAAATTATAT
>N3133
ATGACAAACATCCGAAAAATTCACCCCCTACTAAAAATAGTAAACCACTCATTCATCGAC
CTCCCCACTCCTCCAAACATCTCATCCTGATGAAACTTTGGCTCACTTCTAGGAATTTGC
TTAGTAATTCAAATTGCTACAGGACTTTTCCTAGCCATACATTATACAGCAGACACAACA
ACAGCATTTTCATCAGTATCCCATATCTGCCGAGACGTAAATTACGGATGACTAATCCGT
TATATACACGCAAACGGAGCCTCACTATTCTTCATCTGCCTATTCATCCATATCGGACGA
GGCATTTACTACGGATCCTACATCTTCCAAGAAACATGAAACATCGGTGTAATCCTCCTA
TTTGCCGTAATAGCTACCGCATTCATAGGATACGTCCTACCATGAGGACAAATATCCTTC
TGAGGAGCCACAGTGATTACAAATCTCCTCTCAGCAATCCCGTATATTGGTCCAACAATT
GTAGAATGAATCTGAGGGGGGTTCTCAGTAGACAAAGCCACCCTAACACGATTTTTTGCA
TTCCATTTTATCCTCCCTTTTATTATTACAGCCCTTGTCCTAGTCCACCTCCTATTTCTT
CACGAAACCGGATCCAATAACCCCCTAGGACTAAACTCTAACGCAGACAAAATCCCCTTT
CACCCCTATTACACAGTAAAAGATTTCCTCGGAGTAATTTTACTACTTTTATTCTTCATA
ATCCTAGTTCTCTTCTTTCCTGACCTACTTGGAGACCCAGACAATTACACACCTGCTAAC
CCACTCAACACACCTCCCCATATTAAACCCGAATGATATTTCTTATTTGCCTACGCTATT
CTCCGTTCCATCCCCAATAAACTAGGTGGAGTACTAGCCTTAGTATTATCAATCCTCATT
CTAATTTTCCTACCACTAATCCATACATCAAAACAACGAAGCCTAATATTCCGACCAATT
TCACAAATACTTTACTGAATTTTAATCGCTAACCTGCTTATCCTTACATGAATCGGGGGC
CAACCAGTAGAACACCCATTTATCATTATTGGCCAACTAGCCTCAATTAGCTATTTTTCT
ATCATCCTAATCTTTCTACCAATCGCAGGAATCATCGAAGACAAAATATTAAAATTATAT
>N3134
ATGACAAACATCCGAAAAATTCACCCCCTACTAAAAATAGTAAACCACTCATTCATCGAC
CTCCCCACTCCTCCAAACATCTCATCCTGATGAAACTTTGGCTCACTTCTAGGAATTTGC
TTAGTAATTCAAATTGCTACAGGACTTTTCCTAGCCATACATTATACAGCAGACACAACA
ACAGCATTTTCATCAGTATCCCATATCTGCCGAGACGTAAATTACGGATGACTAATCCGT
TATATACATGCAAACGGAGCCTCACTATTCTTCATCTGCCTATTCATCCATATCGGACGA
GGCATTTACTACGGATCCTACATCTTCCAAGAAACATGAAACATCGGTGTAATTCTCCTA
TTTGCCGTAATAGCTACCGCATTCATAGGATACGTCCTACCATGAGGACAAATATCCTTC
TGAGGAGCCACAGTGATTACAAATCTCCTCTCAGCAATCCCGTATATTGGTCCAACAATT
GTAGAATGAATCTGAGGGGGGTTCTCAGTAGACAAAGCCACCCTAACACGATTTTTCGCA
TTCCATTTTATCCTCCCTTTTATTATTACAGCCCTTGTCCTAGTCCACCTCCTATTTCTT
CACGAAACCGGATCCAATAACCCCCTAGGACTAAACTCTAATGCAGACAAAATCCCCTTT
CACCCCTATTACACAGTAAAAGATTTTCTCGGAGTAATTTTACTACTTCTATTCTTCATA
ATCCTAGTTCTCTTCTTTCCTGACCTACTTGGAGACCCAGACAATTACACACCTGCTAAC
CCACTCAACACACCTCCCCATATTAAACCCGAATGATATTTCTTATTTGCCTACGCTATT
CTCCGTTCCATCCCCAATAAACTAGGTGGAGTACTAGCCTTAGTATTATCAATCCTCATT
CTAATTTTCCTACCACTAATCCATACATCAAAACAACGAAGCCTAATATTCCGACCAATT
TCACAAATACTTTACTGAATTTTAATCGCTAACCTGCTTATCCTTACATGAATCGGGGGC
CAACCAGTAGAACACCCATTTATCATTATTGGCCAACTAGCCTCAATTAGCTATTTTTCT
ATCATCCTAATCTTTCTACCAATCGCAGGAATCATCGAAGACAAAATATTAAAATTATAT
>N3139

ATGACAAACATCCGAAAAATTCACCCCCTACTAAAAATAGTAAACCACTCATTCATCGAC
CTCCCCACTCCTCCAAACATCTCATCCTGATGAAACTTTGGCTCACTTCTAGGAATTTGC
TTAGTAATTCAAATTGCTACAGGACTTTTCCTAGCCATACATTATACAGCAGACACAACA
ACAGCATTTTCATCAGTATCCCATATCTGCCGAGACGTAAATTACGGATGACTAATCCGT
TATATACACGCAAACGGAGCCTCACTATTCTTCATCTGCCTATTCATCCATATCGGACGA
GGCATTTACTACGGATCCTACATCTTCCAAGAAACATGAAACATCGGTGTAATCCTCCTA
TTTGCCGTAATAGCTACCGCATTCATAGGATACGTCCTACCATGAGGACAAATATCCTTC
TGAGGAGCCACAGTGATTACAAATCTCCTCTCAGCAATCCCGTATATTGGTCCAACAATT
GTAGAATGAATCTGAGGGGGGTTCTCAGTAGACAAAGCCACCCTAACACGATTTTTCGCA
TTCCACTTTATCCTCCCTTTTATTATTACAGCCCTTGTCCTAGTCCACCTCCTATTTCTT
CACGAAACCGGATCCAATAACCCCCTAGGACTAAACTCTAATGCAGACAAAATCCCCTTT
CACCCCTACTACACAGTAAAAGATTTTCTTGGAGTAATTTTACTACTTCTATTCTTCATA
ATCCTAGTTCTCTTCTTTCCTGACCTACTTGGAGACCCAGACAATTACACACCTGCTAAC
CCACTCAACACACCTCCCCATATTAAACCCGAATGATATTTCTTATTTGCCTACGCTATT
CTCCGTTCCATCCCCAATAAACTAGGTGGAGTACTAGCCTTAGTATTATCAATCCTCATT
CTAATTTTCCTACCACTAATCCATACATCAAAGCAACGAAGCCTAATATTCCGACCAATT
TCACAAATACTTTACTGAATTTTAATCGCTAACCTGCTTATCCTTACATGAATCGGGGGC
CAACCAGTAGAACACCCATTTATCATTATTGGCCAACTAGCCTCAATTAGCTATTTTTCT
ATCATCCTAATCTTTCTACCAATCGCAGGAATCATTGAAGACAAAATATTAAAATTATAT
>N3158
ATGACAAACATCCGAAAAATTCACCCCCTACTAAAAATAGTAAACCACTCATTCATCGAC
CTCCCCACTCCTCCAAACATCTCATCCTGATGAAACTTTGGCTCACTTCTAGGCATTTGC
TTAGTAATTCAAATTGCTACAGGACTTTTCCTAGCCATACATTATACAGCAGACACAACA
ACAGCATTTTCATCAGTATCACATATCTGCCGAGACGTAAATTACGGATGACTAATCCGT
TATATACACGCAAACGGAGCCTCACTATTCTTCATCTGCCTATTCATCCATATCGGACGA
GGCATTTACTACGGATCCTACATCTTCCAAGAAACATGAAACATCGGTGTAATCCTCCTA
TTTGCCGTAATAGCTACCGCATTCATAGGATACGTCCTACCATGAGGACAAATATCCTTC
TGAGGAGCCACAGTAATTACAAATCTCCTCTCAGCAATCCCGTATATTGGTCCAACAATT
GTAGAATGAATCTGAGGGGGATTCTCAGTAGACAAAGCCACCCTAACACGATTTTTCGCA
TTCCATTTTATCCTCCCTTTTATTATTACAGCCCTTGTCCTAGTCCACCTCCTATTTCTT
CACGAAACCGGATCCAATAACCCCCTAGGACTAAACTCTAATGCAGACAAAATCCCCTTT
CACCCCTATTACACAGTAAAAGATTTTCTCGGAGTAATTTTACTACTTCTATTTTTCATA
ATCCTAGTTCTCTTCTTCCCCGACCTACTTGGAGACCCAGACAATTACACACCTGCTAAC
CCACTCAACACACCTCCCCATATTAAACCCGAATGATATTTCTTATTTGCCTACGCTATT
CTCCGTTCCATCCCCAATAAACTAGGCGGAGTACTAGCCTTAGTATTATCAATCCTCATT
CTAATTTTCCTACCACTAATCCATACATCAAAACAACGAAGCCTAATATTCCGACCAATT
TCACAAATACTTTACTGAATTTTAATCGCTAACCTGCTTATCCTTACATGAATCGGGGGC
CAACCAGTAGAACACCCATTTATCATTATTGGCCAACTAGCCTCAATTAGCTATTTTTCT
ATCATCCTAATCTTTCTACCAATCGCAGGAATCATCGAAGACAAAATATTAAAATTATAT
>N3168
ATGACAAACTTCCGAAAAACTCACCCTCTACTAAAAATAGTAAACCATTCATTCATTGAT
CTCCCCACTCCCCCCAACATCTCATCCTGATGAAACTTCGGCTCACTCCTAGGAATTTGC
TTAGTTATCCAAATCACCACAGGACTTTTCCTAGCTATACATTACACAGCAGACACAACA
ACAGCATTCTCATCAGTCTCACACATTTGCCGAGACGTAAATTACGGATGACTAATCCGC
TATATACACGCAAACGGAGCCTCAATATTTTTTATCTGTTTATTCATCCACATCGGACGA
GGAATTTATTATGGATCCTATATCTTTCAAGAAACATGAAACATCGGTGTAATTCTCTTA
TTCGCTGTAATAGCCACTGCATTTATAGGCTATGTTCTACCATGAGGACAAATATCATTC
TGAGGAGCTACAGTAATCACTAATCTCCTCTCAGCAATCCCATATATTGGCCCAACAATT
GTAGAATGAATTTGAGGAGGTTTCTCAGTAGACAAAGCCACCCTAACACGATTCTTCGCA
TTCCACTTTATCCTCCCATTCATCATTACAGCTCTTGTACTAGTCCATCTCTTGTTCCTA
CACGAGACCGGATCTAATAATCCCCTGGGAATCAACTCAAACGCAGACAAAATCCCTTTT

CACCCCTACTATACAGTAAAAGACTTCCTAGGGGTTATTATCTTTATTTTATTCTTCACA
ATAATAGTCCTATTTTTCCCAGACTTACTTGGAGACCCAGACAACTACACACCCGCCAAT
CCACTTAACACCCCTCCTCACATTAAACCCGAATGATACTTTCTATTTGCCTACGCCATC
CTACGCTCAATTCCCAACAAACTAGGAGGAGTCCTAGCCCTAGTACTTTCAATCCTCATC
CTAGCCCTCCTACCACTTATTCATACATCAAAACAACGAAGCCTAATATTCCGACCGATC
TCACAAATACTATACTGAATTTTAATCGCTAACCTACTTATCCTAACATGAATTGGAGGT
CAACCAGTAGAACACCCATTTATCATCATTGGCCAACTAGCTTCAATCAGTTACTTCTCT
ATTATCCTCATCCTACTTCCAATTGCAGGAATTATCGAAGACAAAATATTGAAATGATAT
>N3180
ATGACAAACATCCGAAAAATTCACCCCTTACTAAAAATAGTAAACCACTCATTCATCGAC
CTCCCCACTCCTCCAAACATCTCATCCTGATGAAACTTTGGCTCACTTCTAGGAATTTGC
TTAGTAATTCAAATTGCTACAGGACTTTTCCTAGCCATACATTATACAGCAGACACAACA
ACAGCATTTTCATCAGTATCCCATATCTGCCGAGACGTAAATTACGGATGACTAATCCGT
TATATACACGCAAACGGAGCCTCACTATTCTTCATCTGCCTATTTATCCATATCGGACGA
GGCATTTACTACGGATCCTACATCTTTCAAGAAACATGAAACATCGGTGTAATCCTCCTA
TTTGCCGTAATAGCTACCGCATTCATAGGATACGTCCTGCCATGAGGACAAATATCCTTC
TGAGGAGCCACAGTGATTACAAATCTCCTCTCAGCAATCCCGTATATTGGTCCAACAATT
GTAGAATGAATCTGAGGGGGGTTCTCAGTAGACAAAGCCACCCTAACACGATTTTTCGCA
TTCCATTTTATCCTCCCTTTTATTATTACAGCCCTTGTCCTAGTCCACCTCCTATTTCTT
CACGAAACCGGATCCAATAACCCCCTAGGACTAAACTCTAATGCAGACAAAATCCCCTTT
CACCCCTATTACACAGTAAAAGATTTTCTCGGAGTAATTTTACTACTTCTATTTTTCATA
ATCCTAGTTCTCTTCTTTCCTGACCTACTTGGAGACCCAGACAATTACACACCTGCTAAC
CCACTCAACACACCTCCCCATATTAAACCCGAATGATATTTCTTATTTGCCTACGCTATT
CTCCGTTCCATCCCCAATAAACTAGGTGGAGTACTAGCCTTAGTATTATCAATCCTCATT
CTAATTTTCCTACCACTAATCCATACATCAAAACAACGAAGCCTAATATTCCGACCAATT
TCACAAATACTTTACTGAATTTTAATCGCTAACCTGCTTATCCTTACATGAATCGGAGGC
CAACCAGTAGAACACCCATTTATCATTATTGGCCAACTAGCCTCAATTAGCTACTTTTCT
ATCATCCTAATCTTTCTACCAATCGCAGGAATCATCGAAGACAAAATATTAAAATTATAT
>N3195
ATGACAAACATCCGAAAAATTCACCCCTTACTAAAAATAGTAAACCACTCATTCATCGAC
CTCCCCACTCCTCCAAACATCTCATCCTGATGAAACTTTGGCTCACTTCTAGGAATTTGC
TTAGTAATTCAAATTGCTACAGGACTTTTCCTAGCCATACATTATACAGCAGACACAACA
ACAGCATTTTCATCAGTATCCCATATCTGCCGAGACGTAAATTACGGATGACTAATCCGT
TATATACACGCAAACGGAGCCTCACTATTCTTCATCTGCCTATTTATCCATATCGGACGA
GGCATTTACTACGGATCCTACATCTTCCAAGAAACATGAAACATCGGTGTAATCCTCCTA
TTTGCCGTAATAGCTACCGCATTCATAGGGTACGTCCTGCCATGAGGACAAATATCCTTC
TGAGGAGCCACAGTGATTACAAATCTCCTCTCAGCAATCCCGTATATTGGTCCAACAATT
GTAGAATGAATCTGAGGGGGGTTCTCAGTAGACAAAGCCACCCTAACACGATTTTTCGCA
TTCCATTTTATCCTCCCTTTTATTATTACAGCCCTTGTCCTAGTCCACCTCCTATTTCTT
CACGAAACCGGATCCAATAACCCCCTAGGACTAAACTCTAATGCAGACAAAATCCCCTTT
CACCCCTATTACACAGTAAAAGATTTTCTCGGAGTAATTTTACTACTTCTATTTTTCATA
ATCCTAGTTCTCTTCTTTCCCGACCTACTTGGAGACCCAGACAATTACACACCTGCTAAC
CCACTCAACACACCTCCCCATATTAAACCCGAATGATATTTCTTATTTGCCTACGCTATT
CTCCGTTCCATCCCCAATAAACTAGGTGGAGTACTAGCCTTAGTATTATCAATCCTCATT
CTAATTTTCCTACCACTAATCCATACATCAAAACAACGAAGCCTAATATTCCGACCAATT
TCACAAATACTTTACTGAATTTTAATCGCTAACCTGCTTATCCTTACATGAATCGGAGGC
CAACCAGTAGAACACCCATTTATCATTATTGGCCAACTAGCCTCAATTAGCTACTTTTCT
ATCATCCTAATCTTTCTACCAATCGCAGGAATCATCGAAGACAAAATATTAAAATTATAT
>N3223
ATGACAAACATTCGAAAAATTCACCCCCTAATAAAAATAGTAAACCACTCATTCATTGAC
CTCCCTACTCCTCCAAACATCTCATCCTGATGAAACTTTGGCTCACTCCTAGGAATTTGC

TTAGTAATTCAAATTACTACAGGACTCTTCCTAGCCATACACTATACAGCAGATACAACA
ACAGCATTTTCATCAGTATCCCACATCTGCCGAGACGTAAATTACGGATGACTAATTCGT
TATATACACGCAAACGGAGCCTCAATATTCTTCATCTGCCTATTTATCCATATCGGACGA
GGAATTTACTACGGATCCTACATCTTTCAAGAAACATGAAACATCGGTGTAATCCTCCTA
TTTGCCGTAATAGCTACCGCATTCATGGGTTACGTCCTACCATGAGGACAAATATCCTTC
TGAGGCGCCACAGTCATTACAAACCTCCTCTCAGCAATCCCATACATTGGCCCAACAATT
GTAGAATGAATCTGAGGAGGCTTCTCAGTAGACAAAGCCACTCTAACACGATTCTTCGCA
TTCCACTTTATCCTTCCCTTTATTATTACAGCTCTCGTCCTAGTCCACCTCTTATTTCTC
CACGAAACTGGATCCAATAACCCTCTAGGACTAAACTCCAACGCAGACAAAATCCCCTTT
CACCCCTATTATACAGTAAAAGATTTTCTCGGAGTAATCTTACTACTTCTATTCTTCACA
ATTTTAGTCCTCTTCTTCCCCGACCTACTTGGAGACCCAGACAATTACACACCCGCCAAC
CCACTTAATACTCCCCCTCATATTAAACCCGAATGATATTTCTTATTTGCCTACGCTATT
CTCCGCTCTATTCCCAATAAACTAGGTGGAGTACTAGCCCTAGTACTTTCAATCCTCATT
CTAATTTTCCTACCACTAATCCATACATCAAAACAACGAAGCCTAATATTCCGACCAATT
TCACAAATACTCTACTGAATTTTAATCGCCAACCTACTTATCCTCACATGAATTGGAGGT
CAACCAGTAGAACACCCATTTATCATTATTGGCCAACTAGCCTCAATTAGCTATTTCTCC
ATCATCCTAATCTTTCTACCAATCGCAGGGATCATCGAAGACAAAATATTAAAATGATAT
>N3312
ATGACAAACTTCCGAAAAACTCACCCTCTACTAAAAATAGTAAACCATTCATTCATTGAT
CTCCCCACTCCCCCCAACATCTCATCCTGATGAAACTTCGGCTCACTCCTAGGAATTTGC
TTAGTTATCCAAATCACCACAGGACTTTTCCTAGCTATACATTACACAGCAGACACAACA
ACAGCATTCTCATCAGTCTCACACATTTGCCGAGACGTAAATTACGGATGACTAATCCGC
TATATACACGCAAACGGAGCCTCAATATTTTTATCTGTTTATTCATCCACATCGGACGA
GGAATTTATTATGGATCCTATATCTTTCAAGAAACATGAAACATCGGTGTAATTCTCTTA
TTCGCTGTAATAGCCACTGCATTTATAGGCTATGTTCTACCATGAGGACAAATATCTTTC
TGAGGAGCTACAGTAATCACTAATCTCCTCTCAGCGATCCCATACATTGGCCCAACAATT
GTAGAATGAATTTGAGGAGGTTTCTCAGTAGACAAAGCCACCCTAACACGATTCTTCGCA
TTCCACTTTATCCTCCCATTCATCATTGCAGCTCTTGTACTAGTCCATCTCTTGTTCCTA
CACGAGACCGGATCTAATAATCCCCTGGGAATCAACTCAAACGCAGACAAAATCCCTTTT
CACCCCTACTATACAGTAAAAGACTTCCTAGGGGTTATTATCTTTATTTTATTCTTCACA
ATAATAGTCCTATTTTTCCCAGACTTACTTGGAGACCCAGACAACTACACACCCGCCAAT
CCACTTAACACCCCTCCTCACATTAAACCCGAATGATACTTTCTATTTGCCTACGCCATC
CTACGCTCAATTCCCAACAAACTAGGAGGAGTCCTAGCCCTAGTACTTTCAATCCTCATC
CTAGCCCTCCTACCACTTATTCATACATCAAAACAACGAAGCCTAATATTCCGACCGATC
TCACAAATACTATACTGAATTTTAATCGCTAACCTACTTATCCTAACATGAATTGGAGGT
CAACCAGTAGAACACCCATTTATCATCATTGGCCAACTAGCTTCAATCAGTTACTTCTCT
ATTATCCTCATCCTACTTCCAATTGCAGGAATTATCGAAGACAAAATATTGAAATGATAT
>N3289_N3313
ATGACAAACTTCCGAAAAACTCACCCTCTACTAAAAATAGTAAACCATTCATTCATTGAT
CTCCCCACTCCCCCCAACATCTCATCCTGATGAAACTTCGGCTCACTCCTAGGAATTTGC
TTAGTTATCCAAATCACCACAGGACTTTTCCTAGCTATACATTACACAGCAGACACAACA
ACAGCATTCTCATCAGTCTCACACATTTGCCGAGACGTAAATTACGGATGACTAATCCGC
TATATACACGCAAACGGAGCCTCAATATTTTTATCTGTTTATTCATCCACATCGGACGA
GGAATTTATTATGGATCCTATATCTTTCAAGAAACATGAAACATCGGTGTAATTCTCTTA
TTCGCTGTAATAGCCACTGCATTTATAGGCTATGTTCTACCATGAGGACAAATATCATTC
TGAGGAGCTACAGTAATCACTAATCTCCTCTCAGCGATCCCATACATTGGCCCAACAATT
GTAGAATGAATTTGAGGAGGTTTCTCAGTAGACAAAGCCACCCTAACACGATTCTTCGCA
TTCCACTTTATCCTCCCATTCATCATTGCAGCTCTTGTACTAGTCCATCTCTTGTTCCTA
CACGAGACCGGATCTAATAATCCCCTGGGAATCAACTCAAACGCAGACAAAATCCCTTTT
CACCCCTACTATACAGTAAAAGACTTCCTAGGGGTTATTATCTTTATTTTATTCTTCACA
ATAATAGTCCTATTTTTCCCAGACTTACTTGGAGACCCAGACAACTACACACCCGCCAAT

CCACTTAACACCCCTCCTCACATTAAACCCGAATGATACTTTCTATTTGCCTACGCCATC
CTACGCTCAATTCCCAACAAACTAGGAGGAGTCCTAGCCCTAGTACTTTCAATCCTCATC
CTAGCCCTCCTACCACTTATTCATACATCAAAACAACGAAGCCTAATATTCCGACCGATC
TCACAAATACTATACTGAATTTTAATCGCTAACCTACTTATCCTAACATGAATTGGAGGT
CAACCAGTAGAACACCCATTTATCATCATTGGCCAACTAGCTTCAATCAGTTACTTCTCT
ATTATCCTCATCCTACTTCCAATTGCAGGAATTATCGAAGACAAAATATTGAAATGATAT
>N3327
ATGACAAACATCCGAAAAATTCACCCCCTAATAAAAATAGTAAACCACTCATTCATTGAC
CTCCCTACTCCTCCAAACATCTCATCCTGATGAAACTTTGGCTCACTCCTAGGAATTTGC
TTAGTAATTCAAATTACTACAGGACTCTTCCTAGCCATACACTATACAGCAGATACAACA
ACAGCATTTTCATCAGTATCCCACATCTGCCGAGACGTAAATTACGGATGACTAATTCGT
TATATACACGCAAACGGAGCCTCAATATTCTTCATCTGCCTATTTATCCATATCGGACGA
GGAATTTACTACGGATCCTACATCTTTCAAGAAACATGAAACATCGGTGTAATCCTCCTA
TTTGCCGTAATAGCTACCGCATTCATGGGTTACGTCCTACCATGAGGACAAATATCCTTC
TGAGGCGCCACAGTCATTACAAACCTCCTCTCAGCAATCCCATACATTGGCCCAACAATT
GTAGAATGAATCTGAGGAGGCTTCTCAGTAGACAAAGCCACTCTAACACGATTCTTCGCA
TTCCACTTTATCCTTCCCTTTATTATTACAGCTCTCGTCCTAGTCCACCTCTTATTTCTC
CACGAAACTGGATCCAATAACCCTCTAGGACTAAACTCCAACGCAGACAAAATCCCCTTT
CACCCCTATTATACAGTAAAAGATTTTCTCGGAGTAATCTTACTACTTCTATTCTTCACA
ATTTTAGTCCTCTTCTTCCCCGACCTACTTGGAGACCCAGACAATTACACACCCGCCAAC
CCACTTAATACTCCCCCTCATATTAAACCCGAATGATATTTTTTATTTGCCTACGCTATT
CTCCGCTCTATTCCCAATAAACTAGGTGGAGTACTAGCCCTAGTACTTTCAATCCTCATT
CTAATTTTCCTACCACTAATCCATACATCAAAACAACGAAGCCTAATATTCCGACCAATT
TCACAAATACTCTACTGAATTTTAATCGCCAACCTACTTATCCTCACATGAATTGGAGGT
CAACCAGTAGAACACCCATTTATCATTATTGGCCAACTAGCCTCAATTAGCTATTTCTCC
ATCATCCTAATCTTTCTACCAATCGCAGGGATCATCGAAGACAAAATATTAAAATGATAT
>N3329
ATGACAAACATCCGAAAAATTCACCCCCTAATAAAAATAGTAAACCACTCATTCATTGAC
CTCCCTACTCCTCCAAACATCTCATCCTGATGAAACTTTGGCTCACTCCTAGGAATTTGC
TTAGTAATTCAAATTACTACAGGACTCTTCCTAGCCATACACTATACAGCAGATACAACA
ACAGCATTTTCATCAGTATCCCACATCTGCCGAGACGTAAATTACGGATGACTAATTCGT
TATATACACGCAAACGGAGCCTCAATATTCTTCATCTGCCTATTTATCCATATCGGACGA
GGTATTTACTACGGATCCTACATCTTTCAAGAAACATGAAACATCGGCGTAATCCTCCTA
TTTGCCGTAATAGCTACAGCATTCATGGGTTATGTCCTACCATGAGGACAAATATCCTTC
TGAGGCGCCACAGTCATTACAAACCTCCTCTCAGCAATCCCATACATTGGCCCAACAATT
GTAGAATGAATCTGAGGGGGCTTCTCAGTAGACAAAGCCACTCTAACACGATTCTTCGCA
TTCCACTTTATCCTTCCCTTTATTATTGCAGCTCTCGTCCTAGTCCACCTCTTATTTCTC
CACGAAACTGGATCCAATAACCCTCTAGGACTAAACTCCAACGCAGACAAAATCCCCTTT
CACCCCTATTATACAGTAAAAGATTTTCTCGGAGTAATCTTACTACTTCTATTCTTCACA
ATTTTAGTCCTCTTCTTCCCCGACCTACTTGGAGACCCAGACAATTACACACCCGCCAAC
CCACTTAATACTCCCCCTCATATTAAACCCGAATGATATTTCTTATTTGCCTACGCTATT
CTCCGCTCTATTCCCAATAAACTAGGTGGAGTACTAGCCCTAGTACTTTCAATCCTCATT
CTAATTTTCCTACCACTAATCCATACATCAAAACAACGAAGCCTAATATTCCGACCAATT
TCACAAGTACTCTACTGAATTTTAATCGCCAACCTACTTATCCTCACATGAATTGGAGGT
CAACCAGTAGAACACCCATTTATCATTATTGGCCAACTAGCCTCAATTAGCTATTTCTCC
ATCATCCTAATCTTTCTACCAATCGCAGGGATCATCGAAGACAAAATATTAAAATGATAT
>M-AZA14
NNNNNNNNNNNNNNNAAAATTCACCCCCTACTAAAAATAGTAAACCACTCATTCATCGAC
CTCCCCACTCCTCCAAACATCTCATCCTGATGAAACTTTGGCTCACTTCTAGGAATTTGC
TTAGTAATTCAAATTGCTACAGGACTTTTCCTAGCCATACATTATACAGCAGACACAACA
ACAGCATTTTCATCAGTATCCCATATCTGCCGAGACGTAAATTACGGATGACTAATCCGT

TATATACACGCAAACGGAGCCTCACTATTCTTCATCTGTCTATTCATCCATATCGGACGA

GGCATTTACTACGGATCCTACATCTTCCAAGAAACATGAAACATCGGTGTAATCCTCCTA

TTTGCCGTAATAGCTACCGCATTCATAGGATACGTCCTACCATGAGGACAAATATCCTTC

TGAGGAGCCACAGTGATTACAAATCTCCTCTCAGCAATCCCGTATATTGGTCCAACAATT

GTAGAATGAATCTGAGGGGGGTTCTCAGTAGACAAAGCCACCCTAACACGATTTTTCGCA

TTCCATTTTATCCTCCCTTTTATTATTACAGCCCTTGTCCTAGTCCACCTCCTATTTCTT

CACGAAACCGGATCCAATAACCCCCTAGGACTAAACTCTAATGCAGACAAAATCCCCTTT

CACCCCTACTACACAGTAAAAGATTTTCTTGGAGTAATTTTACTACTTCTATTCTTCATA

ATCCTAGTTCTCTTCTTTCCTGACCTACTTGGAGACCCAGACAATTACACACCTGCTAAC

CCACTCAACACACCTCCCCATATTAAACCCGAATGATATTTCTTATTTGCCTACGCTATT

CTCCGTTCCATCCCCAATAAACTAGGTGGAGTACTAGCCTTAGTATTATCAATCCTCATT

CTAATTTTCCTACCACTAATCCATACATCAAAACAACGAAGCCTAATATTCCGACCAATT

TCACAAATACTTTACTGAATTTTAATCGCTAACCTGCTTATCCTTACATGAATCGGGGGC

CAACCAGTAGAACACCCATTTATCATTATTGGCCAACTAGCCTCAATTAGCTATTTTTCT

ATCATCCTAATCTTTCTACCAATCGCAGGAATCATCGAAGA-------------------

>M-AZA13

NNNNNNNNNNNNNNNNAAAATTCACCCCCTACTAAAAATAGTAAACCACTCATTCATCGAC

CTCCCCACTCCTCCAAACATCTCATCCTGATGAAACTTTGGCTCACTTCTAGGAATTTGC

TTAGTAATTCAAATTGCTACAGGACTTTTCCTAGCCATACATTATACAGCAGACACAACA

ACAGCATTCTCATCAGTATCCCATATCTGCCGAGACGTAAATTACGGATGACTAATCCGT

TATATACACGCAAACGGAGCCTCACTATTCTTCATCTGCCTATTTATCCATATCGGACGA

GGCATTTACTACGGATCCTACATCTTCCAAGAAACATGAAACATCGGTGTAATCCTCCTA

TTTGCCGTAATAGCTACCGCATTCATAGGGTACGTCCTACCATGAGGACAAATATCCTTC

TGAGGAGCCACAGTGATTACAAATCTCCTCTCAGCAATCCCATATATTGGCCCAACAATT

GTAGAATGAATCTGAGGGGGGTTCTCAGTAGACAAAGCCACCCTAACACGATTTTTCGCA

TTCCACTTTATCCTCCCTTTTATTATTACAGCCCTTGTCCTAGTCCACCTCCTATTTCTT

CACGAAACCGGATCCAATAACCCCCTAGGACTAAACTCTAATGCAGACAAAATCCCCTTT

CACCCCTATTACACAGTAAAAGATTTTCTCGGAGTAATTTTACTACTTCTATTCTTCATA

ATCCTAGTTCTCTTCTTTCCTGACCTACTTGGAGACCCAGACAATTACACACCTGCTAAC

CCACTCAACACACCTCCCCATATTAAACCCGAATGATATTTCTTATTTGCCTACGCTATT

CTCCGTTCCATCCCCAATAAACTAGGTGGAGTACTAGCCTTAGTATTATCAATCCTCATT

CTAATTTTCCTACCACTAATCCATACATCAAAACAACGAAGCCTAATATTCCGACCAATT

TCACAAATACTTTACTGAATTTTAATCGCTAACCTGCTTATCCTTACATGAATCGGGGGC

CAACCAGTAGAACACCCATTTATCATTATTGGCCAACTAGCCTCAATTAGCTATTTTTCT

ATCATCCTAATCTTTCTACCAATCGCAGGAATCATCGAAGA-------------------

>M-CHA5

NNNNNNNNNNNNNNNNAAAATTCACCCCCTAATAAAAATAGTAAACCACTCATTCATTGAC

CTCCCTACTCCTCCCAACATCTCATCCTGATGAAACTTTGGATCACTCCTAGGAATTTGC

TTAGTAATTCAAATTGCTACAGGATTCTTTTTAGCCATACATTATACAGCAGATACAACA

ACAGTTTTTTCATCAGTATCCCACATCTGCCGAGACGTAAATTGGGGAAGACTAATTCGT

TATATACGCGCAAGCGGAGCCTCAATATTCTTCATCTGCCTATTTATCCATATCGGAAGA

GGAATTTACTGGGGATCCTACCTCTTTCAAGAAACATGAAACATCGGTGTAATCCTCCTA

TTTGCCGTAATAGCTACCGCATTCATGGGTTACGTCCTACCATGAGGACAAATATCCTTA

AGAGGCGCCACAGTCATTACAAACCTCCTCTCAGCAATCCCATACATTGGCCCAACAATT

GTAGAATGAATCTGAGGAGGCTTCTCAGTAGACAAAGCCACTCTAACACGATTCTTCGCA

TTCCACTTTATCCTTCCCTTTATTATTACAGCTCTCGTCCTAGTCCACCTCTTATTTCTC

CACGAAACTGGATCCAATAACCCTCTAGGACTAAACTCCAACGCAGACAAAATCCCCTTT

CACCCCTATTATACAGTAAAAGATTTTCTCGGAGTAATCTTACTACTTCTATTCTTCACA

ATTTTAGTCCTCTTCTTCCCCGACCTACTTGGAGACCCAGACAATTACACACCCGCCAAC

CCACTTAATACTCCCCCTCATATTAAACCCGAATGATATTTCTTATTTGCCTACGCTATT

CTCCGCTCTATTCCCAATAAACTAGGTGGAGTACTAGCCCTAGCACTTTCAATCCTCATT

CTAATTTTCCTACCACTAATCCATACATCAAAACAACGAAGCCTAATATTCCGACCAATT
TCACAAATACTATACTGAATTTTAATCGCCAACCTACTTATCCTCACATGAATTGGAGGT
CAACCAGTAGAACACCCATTTATCATCATTGGCCAACTAGCCTCAATCAGCTATTTCTCC
ATCATCCTAATCTTCCTACCAATCGCAGGGATCATCGAAGA------------------
>M5314
NNNNNNNNNNNNNNNNAAAATGCACCCCCTACTAAAAATAGTAAACCACTCATTCATCGAC
CTCCCCACTCCTCCAAACATCTCATCCTGATGAAACTTTGGATCACTTCTAGGAATTTGC
TTAGTAATTCAAATTGCTACAGGATTTTTCTTAGCCATACATTATACAGCAGACACAACA
ACAGTTTTTTCATCAGTATCCCATATTTGCCGAGACGTAAATTGGGGAAGACTAATCCGT
TATATACGCGCAAGGGGAGCCTCACTATTCTTCATCTGTCTATTCATCCATATGGGACGA
GGCATTTACTACGGATCCTACATCTTCCAAGAAACATGAAACATCGGTGTAATCCTCCTA
TTTGCCGTAATAGCTAGCGCATTCATAGGATATGTCCTACCATGAGGACAAATATCCTTC
TGAGGAGCCACAGTGATTACAAATCTCCTCTCAGCAATCCCGTATATTGGTCCAACAATT
GTAGAATGAATCTGAGGGGGGTTCTCAGTAGACAAAGCCACCCTAACACGATTTTTCGCA
TTCCATTTTATCCTCCCTTTTATTATTACAGCCCTTGTCCTAGTCCACCTCCTATTTCTT
CGCGAAACCGGATCCAATAACCCCCTAGGACTAAACTCTAATGCAGACAAAATCCCCTTT
CACCCCTACTACACAGTAAAAGATTTTCTTGGAGTAATTTTACTACTTCTATTCTTCATA
ATCCTAGTTCTCTTCTTTCCTGACCTACTTGGAGACCCAGACAATTACACACCTGCTAAC
CCACTCAACACACCTCCCCATATTAAACCCGAATGATATTTCTTATTTGCCTACGCTATT
CTCCGTTCCATCCCCAATAAACTAGGTGGAGTACTAGCCTTAGTATTATCAATCCTCATT
CTAATTTTCCTACCACTAATCCATACATCAAAACAACGAAGCCTAATATTCCGACCAATT
TCACAAATACTATAYTGAATTTTAATCGCTAACCTGATTATCCTTACATGAATCGGGGGT
CAACCAGTAGAACATCCATTTATCATTATTGGCCAACTAGCCTCAATCAGCTATTTTTCT
ATCATCCTAATCTTTCTNCCAATCGCAGGAATCATCGAAGA------------------
>M-INA16
NNNNNNNNNNNNNNNNAAAATTCACCCCCTACTAAAAATAGTAAACCACTCATTCATCGAC
CTCCCCACTCCTCCAAACATCTCATCCTGATGAAACTTTGGCTCACTTCTAGGAATTTGC
TTAGTAATTCAAATTGCTACAGGACTTTTCCTAGCCATACATTACACAGCAGACACAACA
ACAGCATTTTCATCAGTATCCCATATCTGCCGAGACGTAAATTACGGATGACTAATCCGT
TATATACACGCAAACGGAGCCTCACTATTCTTCATCTGCCTATTCATCCATATCGGACGA
GGCATTTACTACGGATCCTACATCTTCCAAGAAACATGAAACATCGGTGTAATCCTCCTA
TTTGCCGTAATAGCTACCGCATTCATAGGATACGTCCTACCATGAGGACAAATATCCTTC
TGAGGAGCCACAGTGATTACAAATCTCCTCTCAGCAATCCCGTATATTGGTCCAACAATT
GTAGAATGAATCTGAGGGGGGTTCTCAGTAGACAAAGCCACCCTAACACGATTTTTCGCA
TTCCATTTTATCCTCCCTTTTATTATTACAGCCCTTGTCCTAGTCCACCTCCTATTTCTT
CACGAAACCGGATCCAATAACCCCCTAGGACTAAACTCTAATGCAGACAAAATCCCCTTT
CACCCCTACTACACAGTAAAAGATTTTCTTGGAGTAATTTTATTACTTCTATTCTTCATA
ATCCTAGTTCTCTTCTTTCCTGACCTACTTGGAGACCCAGACAATTACACACCTGCTAAC
CCACTCAACACACCTCCCCATATTAAACCCGAATGATATTTCTTATTTGCCTACGCTATT
CTCCGTTCCATCCCCAATAAACTAGGTGGAGTACTAGCCTTAGTATTATCAATCCTCATT
CTAATTTTCCTACCACTAATCCATACATCAAAACAACGAAGCCTAATATTCCGACCAATT
TCACAAATACTTTACTGAATTTTAATCGCTAACCTGCTTATCCTTACATGAATCGGGGGC
CAACCAGTAGAACACCCATTTATCATTATCGGCCAACTAGCCTCAATTAGCTATTTTTCT
ATCATCCTAATCTTTCTACCAATCNNAGGAATTATCGAAGA------------------
>M-TAD8
NNNNNNNNNNNNNNNNAAAATTCACCCCCTACTAAAAATAGTAAACCACTCATTCATCGAC
CTCCCCACTCCTCCAANCATCTCATCCTGATGAAACTTTGGCTCACTTCTAGGAATTTGC
CTAGTAATTCAAATTGCTACAGGACTTTTCCTAGCCATACATTATACAGCAGACACAACA
ACAGCATTTTCATCAGTATCCCATATCTGCCGAGACGTAAATTACGGATGACTAATCCGT
TATATACACGCAAACGGAGCCTCACTATTCTTCATCTGCCTATTTATCCATATCGGACGA
GGCATTTACTACGGATCCTACATCTTCCAAGAAACATGAAACATCGGTGTAATCCTCCTA

TTTGCCGTAATAGCTACCGCATTCATAGGGTACGTCCTACCATGAGGACAAATATCCTTC
TGAGGAGCCACAGTAATTACAAATCTCCTCTCAGCAATCCCATATATTGGCCCAACAATT
GTAGAATGAATCTGAGGGGGGTTCTCAGTAGACAAAGCCACCCTAACACGATTTTTCGCA
TTCCACTTTATCCTCCCTTTTATTATTACAGCCCTTGTCCTAGTCCACCTCTTATTTCTC
CACGAAACCGGATCCAATAACCCCCTAGGACTAAACTCTAATGCAGACAAAATCCCCTTT
CACCCCTATTACACAGTAAAAGATTTTCTCGGAGTAATTTTACTACTTCTATTCTTCATA
ATCCTAGTTCTCTTCTTCCCTGACCTACTTGGAGACCCAGACAATTACACACCTGCTAAC
CCACTCAACACACCTCCCCATATTAAACCCGAATGATACTTCTTATTTGCCTACGCTATT
CTCCGTTCCATCCCCAATAAACTAGGTGGAGTACTAGCCTTAGTATTATCAATCCTCATT
CTAATTTTCCTACCACTAATCCATACATCAAAACAACGAAGCCTAATATTCCGACCAATT
TCACAAATACTTTACTGAATTTTAATCGCTAACCTGGTTATCCTTACATGAATCGGGGGC
CAACCAGTAGAACACCCATTTATCATTATTGGCCAACTAGCCTCAATTAGCTATTTTTCT
ATCATCCTAATCTTTCTACCAATCGCAGGAATCATCGAAGA-------------------
>M-TAD7
NNNNNNNNNNNNNNNNAAAATTCACCCCCTACTAAAAATAGTAAACCACTCATTCATCGAC
CTCCCCACTCCTCCAAACATCTCATCCTGATGAAACTTTGGCTCACTTCTAGGAATTTGC
TTAGTAATTCAAATTGCTACAGGACTTTTCCTAGCCATACATTATACAGCAGACACAACA
ACAGCATTTTCATCAGTATCCCATATCTGCCGAGACGTAAATTACGGATGACTAATCCGT
TATATACACGCAAACGGAGCCTCACTATTCTTCATCTGCCTATTCATCCATATCGGACGA
GGCATTTACTACGGATCCTACATCTTCCAAGAAACATGAAACATCGGCGTAATCCTCCTA
TTTGCCGTAATAGCTACCGCATTCATAGGATACGTCCTACCATGAGGACAAATATCCTTC
TGAGGAGCCACAGTGATTACAAATCTCCTCTCAGCAATCCCGTATATTGGTCCAACAATT
GTAGAATGAATCTGAGGGGGGTTCTCAGTAGACAAAGCCACCCTAACACGATTTTTCGCA
TTCCATTTTATCCTCCCTTTTATTATTACAGCCCTTGTCCTAGTCCACCTCCTATTTCTT
CACGAAACCGGATCCAATAACCCCCTAGGACTAAACTCTAATGCAGACAAAATCCCCTTT
CACCCCTATTATACAGTAAAAGATTTTCTCGGAGTAATTTTACTACTTCTATTCTTCATA
ATCCTAGTTCTCTTCTTTCCTGACCTACTTGGAGACCCAGACAATTACACACCTGCTAAC
CCACTCAACACACCTCCCCATATTAAACCCGAATGATATTTCTTATTTGCCTACGCTATT
CTCCGTTCTATCCCCAATAAACTAGGTGGAGTACTAGCCTTAGTATTATCAATCCTCATT
CTAATTTTCCTACCACTAATCCATACATCAAAACAACGAAGCCTAATATTCCGACCAATT
TCACAAATACTTTACTGAATCTTAATCGCTAACCTGCTTATCCTTACATGAATCGGGGGC
CAACCAGTAGAACACCCATTTATCATTATTGGCCAACTAGCCTCAATTAGCTATTTTTCT
ATCATCCTAATCTTTCTACCAATCGCAGGAATCATCGAAGA-------------------
>M-INA22
NNNNNNNNNNNNNNNNAAAATTCACCCCCTGATAAAAATAGTAAACCACTCATTCATTGAC
CTCCCTACTCCTCCAAACATCTCATCCTGATGAAACTTTGGCTCACTCCTAGGAATTTGC
TTAGTAATTCAAATTACTACAGGACTCTTCCTAGCCATACACTATACAGCAGATACAACA
ACAGCATTTTCATCAGTATCCCACATCTGCCGAGACGTAAATTACGGATGACTAATTCGT
TATATGCACGCAAACGGAGCCTCAATATTCTTCATCTGCCTATTTATCCATATCGGACGA
GGAATTTACTACGGATCCTACATCTTTCAAGAAACATGAAACATCGGTGTAATCCTCCTA
TTTGCCGTAATAGCTACCGCATTCATGGGTTACGTCCTACCATGAGGACAAATATCCTTC
TGAGGCGCCACAGTCATTACAAACCTCCTCTCAGCAATCCCATACATTGGCCCAACAATT
GTAGAATGAATCTGAGGAGGCTTCTCAGTAGACAAAGCCACTCTAACACGATTCTTCGCA
TTCCACTTTATCCTTCCCTTTATTATTACAGCTCTCGTCCTAGTCCACCTCTTATTTCTC
CACGAAACTGGATCCAATAACCCTCTAGGACTAAACTCCAACGCAGACAAAATCCCCTTT
CACCCCTATTATACAGTAAAAGATTTTCTTGGAGTAATCTTACTACTTCTATTCTTCACA
ATTTTAGTCCTCTTCTTCCCTGACCTACTTGGAGACCCAGACAATTACACACCCGCCAAC
CCACTTAATACTCCCCCTCATATTAAACCCGAATGATATTTCTTATTTGCCTACGCTATT
CTCCGCTCTATTCCCAATAAACTAGGTGGAGTACTAGCCCTAGTACTTTCAATCCTCATT
CTAATTTTCCTACCACTAATCCATACATCAAAACAACGAAGCCTAATATTCCGACCAATT
TCACAAATACTCTACTGAATTTTAATCGCCAACCTACTTATCCTCACATGAATTGGAGGT

CAACCAGTAGAACACCCATTTATCATTATTGGCCAACTAGCCTCAATTAGCTATTTCTCC

ATCATCCTAATCTTTCTACCAATCGCAGGGATCATCGAAGA------------------

>M-TES28

NNNNNNNNNNNNNNNNAAAATTCACCCCCTAATAAAAATAGTAAACCACTCATTCATTGAC

CTCCCTACTCCTCCAAACATCTCATCCTGATGAAACTTTGGCTCACTCCTAGGAATTTGC

TTAGTAATTCAAATTACTACAGGACTCTTCCTAGCCATACACTATACAGCAGATACAACA

ACAGCATTTTCATCAGTATCCCACATCTGCCGAGACGTAAATTACGGATGACTAATTCGT

TATATACACGCAAACGGAGCCTCAATATTCTTCATCTGCCTATTTATCCATATCGGACGA

GGAATTTACTACGGATCCTACATCTTTCAAGAAACATGAAACATCGGTGTAATCCTCCTA

TTTGCCGTAATAGCTACCGCATTCATGGGTTACGTCCTACCATGAGGACAAATATCCTTC

TGAGGCGCCACAGTCATTACAAACCTCCTCTCAGCAATCCCATACATTGGCCCAACAATT

GTAGAATGAATCTGAGGAGGCTTCTCAGTAGACAAAGCCACTCTAACACGATTCTTCGCA

TTCCACTTTATCCTTCCCTTTATTATTACAGCTCTCGTCCTAGTCCACCTCTTATTTCTC

CACGAAACTGGATCCAATAACCCTCTAGGACTAAACTCCAACGCAGACAAAATCCCCTTT

CACCCCTATTATACAGTAAAAGATTTTCTCGGAGTAATCTTACTACTTCTATTCTTCACA

ATTTTAGTCCTCTTCTTCCCCGACCTACTTGGAGACCCAGACAATTACACACCCGCCAAC

CCACTTAATACTCCCCCTCATATTAAACCCGAATGATATTTCTTATTTGCCTACGCTATT

CTCCGCTCTATTCCCAATAAACTAGGTGGAGTACTAGCCCTAGTACTTTCAATCCTCATT

CTAATTTTCCTACCACTAATCCATACATCAAAACAACGAAGCCTAATATTCCGACCAATT

TCACAAATACTCTACTGAATTTTAATCGCCAACCTACTTATCCTCACATGAATTGGAGGT

CAACCAGTAGAACACCCATTTATCATTATTGGCCAACTAGCCTCAATTAGCTATTTCTCC

ATCATCCTAATCTTTCTACCAATCGCAGGGATCATCGAAGA------------------

>M-TES30

NNNNNNNNNNNNNNNNAAAATTCACCCCCTAATAAAAATAGTAAACCACTCATTCATTGAC

CTCCCTACTCCTCCAAACATCTCATCCTGATGAAACTTTGGCTCACTCCTAGGAATTTGC

TTAGTAATTCAAATTACTACAGGACTCTTCCTAGCCATACACTATACAGCAGATACAACA

ACAGCATTTTCATCAGTATCCCACATCTGCCGAGACGTAAATTACGGATGACTAATTCGT

TATATACACGCAAACGGAGCCTCAATATTCTTCATCTGCCTATTTATCCATATCGGACGA

GGAATTTACTACGGATCCTACATCTTTCAAGAAACATGAAACATCGGTGTAATCCTCCTA

TTTGCCGTAATAGCTACCGCATTCATGGGTTACGTCCTACCATGAGGACAAATATCCTTC

TGAGGCGCCACAGTCATTACAAACCTCCTCTCAGCAATCCCATACATTGGCCCAACAATT

GTAGAATGAATCTGAGGAGGCTTCTCAGTAGACAAAGCCACTCTAACACGATTCTTCGCA

TTCCACTTTATCCTTCCCTTTATTATTACAGCTCTCGTCCTAGTCCACCTCTTATTTCTC

CACGAAACTGGATCCAATAACCCTCTAGGACTAAACTCCAACGCAGACAAAATCCCCTTT

CACCCCTATTATACAGTAAAAGATTTTCTCGGAGTAATCTTACTACTTCTATTCTTCACA

ATTTTAGTCCTCTTCTTCCCCGACCTACTTGGAGACCCAGACAATTACACACCCGCCAAC

CCACTTAATACTCCCCCTCATATTAAACCCGAATGATATTTCTTATTTGCCTACGCTATT

CTCCGCTCTATTCCCAATAAACTAGGTGGAGTACTAGCCCTAGTACTTTCAATCCTCATT

CTAATTTTCCTACCACTAATCCATACATCAAAACAACGAAGCCTAATATTCCGACCAATT

TCACAAATACTCTACTGAATTTTAATCGCCAACCTACTTATCCTCACATGAATTGGAGGT

CAACCAGTAGAACACCCATTTATCATTATTGGCCAACTAGCCTCAATTAGCTATTTCTCC

ATCATCCTAATCTTTCTACCAATCGCAGGGATCATCGAAGA------------------

>M-TOU1

NNNNNNNNNNNNNNNNAAAACTCACNCACTACTAAAAATAGTTAATCACTCATTCATTGAT

CTTCCCACTCCCCCTAATATCTCATCTTGATGAAACTTTGGCTCACTTCTAGGAATTTGC

TTAATAATTCAAATCGCTACAGGACTCTTCCTAGCCATACATTATACAGCAGACACAACA

ACAGCATTCTCATCAGTATCCCATATCTGCCGAGACGTCAATTATGGATGACTAATCCGC

TATATACATGCAAACGGAGCTTCAATATTCTTTATTTGCCTATTCATTCACATTGGACGA

GGAATTTACTACGGATCTTATATCTTTCAAGAAACATGAAACATTGGAGTAATTCTCTTA

TTTGCCGTAATAGCCACCGCATTCATAGGATATGTACTTCCATGAGGACAAATATCCTTC

TGAGGGGCCACAGTCATTACAAATCTTCTTTCAGCTATTCCATATATTGGCCCAACAATC

GTAGAATGAATTTGAGGAGGATTTTCAGTGGACAAAGCCACTTTAACACGATTTTTCGCA
TTTCACTTCATTCTCCCCTTTATTATCACAGCCTTAGTCCTAGTCCATCTTCTATTCCTT
CACGAAACCGGATCTAATAATCCCCTAGGCCTTAACTCCAACTCAGACAAAATCCCTTTT
CACCCATACTACACAGTAAAAGATTTTCTCGGAGTAATTCTACTACTTCTATTTTTCACA
ATTTTAGTCCTCTTCTTCCCTGACTTACTTGGAGATCCAGACAACTACACACCCGCTAAC
CCCCTTAACACTCCCCCCCACATTAAACCCGAATGATATTTCTTATTTGCCTATGCTATC
CTACGTTCAATTCCTAACAAACTAGGAGGAGTCCTAGCCCTAGTACTTTCAATCCTCATC
CTAATTTTTTTACCACTCATTCACACATCAAAACAACGAAGCCTAATATTCCGACCCATC
TCCCAAATACTTTACTGAATCTTAATTGCCAACCTACTTATCCTCACATGAATCGGAGGC
CAACCAGTAGAACACCCATTTATTATCATTGGCCAACTAGCCTCAATCAGTTACTTTTCC
ATTATTCTAATTCTTTTACCAATCGCAGGAATCATTGAAGA------------------
>M-TOU11
NNNNNNNNNNNNNNNNAAAATTCACCCACTACTAAAAATAGTTAACCACTCATTCATTGAT
CTTCCCACTCCCCCTAATATTTCATCTTGATGAAACTTTGGCTCACTCCTAGGAATTTGC
TTAATAATTCAAATCGCTACAGGACTTTTCCTAGCCATACATTATACAGCAGACACAACA
ACAGCATTCTCATCAGTATCCCATATCTGCCGAGACGTCAATTATGGATGACTAATCCGC
TATATACATGCAAACGGAGCTTCAATATTCTTTATTTGCCTATTCATTCACATCGGACGA
GGAATTTACTACGGATCTTATATCTTTCAAGAAACATGAAACATTGGAGTAATTCTCTTA
TTTGCCGTAATAGCCACCGCATTCATAGGGTATGTACTTCCATGAGGACAAATATCCTTC
TGAGGGGCCACAGTCATTACAAATCTTCTTTCAGCTATTCCATATATTGGCCCAACAATC
GTAGAATGAATTTGAGGAGGATTTTCAGTGGACAAAGCCACTTTAACACGATTTTTCGCA
TTTCACTTCATTCTCCCCTTTATTATCACAGCCTTAGTCCTAGTCCATCTCCTATTCCTT
CACGAAACCGGATCTAATAATCCCCTAGGCCTTAACTCCAACTCAGACAAAATCCCTTTT
CACCCATACTACACAGTAAAAGATTTTCTCGGAGTAATTCTACTACTTCTATTTTTCACA
ATTTTAGTCCTCTTCTTCCCTGACTTACTTGGAGATCCAGACAACTACACACCCGCTAAC
CCCCTTAACACTCCCCCCCACATTAAACCCGAATGATATTTCTTATTTGCCTATGCTATC
CTACGTTCAATTCCTAACAAACTAGGAGGAGTCCTAGCCCTAGTACTTTCAATCCTTATC
CTAATTTTTTTACCACTCATTCACACATCAAAACAACGAAGCCTAATATTCCGACCTATT
TCCCAAATACTTTACTGAATCTTAATCGCCAACCTACTTATCCTCACATGAATCGGAGGC
CAACCAGTAGAACACCCATTTATTATCATTGGCCAACTAGCCTCAATCAGTTACTTTTCC
ATTATCCTAATTCTTTTACCAATCGCAGGAATCATTGAAGA------------------
>M-TOU7
NNNNNNNNNNNNNNNNAAAATTCACCCACTACTAAAAATAGTTAACCACTCATTCATTGAT
CTTCCCACTCCCCCTAATATTTCATCTTGATGAAACTTTGGCTCACTCCTAGGAATTTGC
TTAATAATTCAAATCGCTACAGGACTTTTCCTAGCCATACATTATACAGCAGACACAACA
ACAGCATTCTCATCAGTATCCCATATCTGCCGAGACGTCAATTATGGATGACTAATCCGC
TATATACATGCAAACGGAGCTTCAATATTCTTTATTTGCCTATTCATTCACATCGGACGA
GGAATTTACTACGGATCTTATATCTTTCAAGAAACATGAAACATTGGAGTAATTCTCTTA
TTTGCCGTAATAGCCACCGCATTTATAGGGTATGTACTTCCATGAGGACAAATATCCTTC
TGAGGGGCCACAGTCATTACTAATCTTCTCTCAGCTATTCCATATATTGGCCCAACAATC
GTAGAATGAATTTGAGGAGGATTTTCAGTAGACAAAGCCACTTTAACACGATTTTTCGCA
TTTCACTTCATTCTCCCCTTTATTATCACAGCCTTAGTCCTAGTCCATCTCCTATTCCTT
CACGAAACCGGATCTAATAATCCCCTAGGCCTTAACTCCAACTCAGACAAAATCCCCTTT
CACCCATACTACACAATAAAAGATTTTCTCGGAGTAATTCTACTACTTCTATTTTTCACA
ATTTTAGTCCTCTTCTTCCCTGACTTACTTGGAGATCCAGACAACTACACACCCGCTAAC
CCCCTTAACACTCCCCCCCACATTAAACCCGAATGGTATTTCTTATTTGCCTATGCTATC
CTACGTTCAATTCCTAACAAACTAGGAGGAGTCCTAGCCCTAGTACTTTCAATCCTTATC
CTAATTTTTTTACCACTCATTCACACATCAAAACAACGAAGCCTAATATTCCGACCTATT
TCCCAAATACTTTACTGAATCTTAATTGCCAACCTACTTATCCTCACATGAATCGGAGGC
CAACCAGTAGAACACCCATTTATTATCATTGGCCAACTAGCCTCAATCAGTTACTTTTCC
ATTATCCTAATTCTTTTACCAATCGCAGGAATCATTGAAGA------------------

>M-TOU3

NNNNNNNNNNNNNNNNNAGATNNNNNNCTTACTAAAAATAGTTAACCACTCATTCATTGAT

CTTCCCACTCCCCCTAATATTTCATCTTGATGAAACTTTGGCTCACTCCTAGGAATTTGC

TTAATAATTCAAATCGCTACAGGACTTTTCCTAGCCATACATTATACAGCAGACACAACA

ACAGCATTCTCATCAGTATCCCATATCTGCCGAGACGTCAATTATGGATGACTAATCCGC

TATATACATGCAAACGGAGCTTCAATATTCTTTATTTGCCTATTCATTCACATCGGACGA

GGAATTTACTACGGATCTTATATCTTTCAAGAAACATGAAACATTGGAGTAATTCTCTTA

TTTGCCGTAATAGCCACCGCATTCATAGGGTATGTACTTCCATGAGGACAAATATCCTTC

TGAGGGGCCACAGTCATTACAAATCTTCTTTCAGCTATCCCATATATTGGCCCAACAATC

GTAGAATGAATTTGAGGAGGATTTTCAGTGGACAAAGCCACTTTAACACGATTTTTCGCA

TTTCACTTCATTCTCCCCTTTATTATCACAGCCTTAGTCCTAGTCCATCTCCTATTCCTT

CACGAAACCGGATCTAATAATCCCCTAGGCCTTAACTCCAACTCAGACAAAATCCCTTTT

CACCCATACTACACAGTAAAAGATTTTCTCGGAGTAATTCTACTACTTCTATTTTTCACA

ATTTTAGTCCTCTTCTTCCCTGACTTACTTGGAGATCCAGACAACTACACACCCGCTAAC

CCCCTTAACACTCCCCCCCACATTAAACCCGAATGGTATTTCTTATTTGCCTATGCTATC

CTACGTTCAATTCCTAACAAACTAGGAGGAGTCCTAGCCCTAGTACTTTCAATCCTTATC

CTAATTTTTTTACCACTCATTCACACATCAAAACAACGAAGCCTAATATTCCGACCTATT

TCCCAAATACTTTACTGAATCTTAATTGCCAACCTACTTATCCTCACATGAATCGGAGGC

CAACCAGTAGAACACCCATTTATCATCATTGGCCAACTAGCCTCAATCAGTTACTTTTCC

ATTATCCTAATTCTTTTACCAATCGCAGGAATTATTGAAGA------------------

>M-TES23

ATGACAAACTTCCGAAAAACTCACCCTCTACTAAAAATAGTAAACCATTCATTCATTGAT

CTCCCCACTCCCCCCAACATCTCATCCTGATGAAACTTCGGCTCACTCCTAGGAATTTGC

TTAGTTATCCAAATCACCACAGGACTTTTCCTAGCTATACATTACACAGCAGACACAACA

ACAGCATTCTCATCAGTCTCACACATTTGCCGAGACGTAAATTACGGATGACTAATCCGC

TATATACACGCAAACGGAGCCTCAATATTTTTTATCTGTTTATTCATCCACATCGGACGA

GGAATTTATTATGGATCCTATATCTTTCAAGAAACATGAAACATCGGTGTAATTCTCTTA

TTCGCTGTAATAGCCACTGCATTTATAGGCTATGTTCTACCATGAGGACAAATATCTTTC

TGAGGAGCTACAGTAATCACTAATCTCCTCTCAGCAATCCCATACATTGGCCCAACAATT

GTAGAATGAATTTGAGGAGGTTTCTCAGTAGACAAAGCCACCCTAACACGATTCTTCGCA

TTCCACTTTATCCTCCCATTCATCATTGCAGCTCTTGTACTAGTCCATCTCTTGTTCCTA

CACGAGACCGGATCTAATAATCCCCTGGGAATCAACTCAAACGCAGACAAAATCCCTTTT

CACCCCTACTATACAGTAAAAGACTTCCTAGGGGTTATTATCTTTATTTTATTCTTCACA

ATAATAGTCCTATTTTTCCCAGACTTACTTGGAGACCCAGACAACTACACACCCGCCAAT

CCACTTAACACCCCTCCTCACATTAAACCCGAATGATACTTTCTATTTGCCTACGCCATC

CTTCGCTCAATTCCCAACAAACTAGGAGGAGTCCTAGCCCTAGTACTTTCAATCCTCATC

CTAGCCCTCCTACCACTTATTCATACATCAAAACAACGAAGCCTAATATTCCGACCGATC

TCACAAATACTATACTGAATTTTAATCGCTAACCTACTTATCCTAACATGAATTGGAGGT

CAACCAGTAGAACACCCATTTATCATCATTGGCCAACTAGCTTCAATCAGTTACTTCTCT

ATTATCCTCATCCTACTTCCAATTGCAGGAATTATCGAAGACAAAATATTGAAATGATAT

>M5238

NNNNNNNNNNNNNNNNAAANTCCACCCATTACTAAAAATAGTAAATCACTCATTCATTGAC

CTTCCCACACCTCCTAATATTTCATCCTGATGAAACTTTGG-TCCCTCCTAGGAGTTTGT

TTAGTAATCCAAATTGCTACAGGACTCTTTTTAGCCATACACTACACAGCAGACACAACA

ACAGCATTTTCATCAGTATCTCACATCTGCCGAGATGTAAACTAGGGATGACTAATCCGT

TATATACACGCAAACGGAGCCTCAATATTCTTCATTTGCTTATTCATTCATATTGGACGA

GGAATCTACTATGGATCATATATTTTTCAAGAAACATGAAACATCGGTGTAATTCTCTTA

TTCGCTGTGATAGCCACTGCATTCATAGGATATGTTCTACCATGAGGACAAATATCCTTC

TGAGGAGCCACAGTTATCACAAATCTTCTTTCAGCAATTCCATATATTGGCCCAACAATT

GTAGAATGAATCTGAGGAGGTTTTTCAGTAGATAAAGCCACTCTAACACGATTCTTTGCA

TTCCACTTTATTCTTCCCTTTATTATCACAGCCCTTGTTCTAGTCCATCTCTTATTCCTC

CACGAAACTGGATCTAACAATCCCCTAGGACTTAACTCCAACGCAGATAAAATTCCCTTC

CACCCATACTACACAATAAAAGATTTTCTTGGAGTAATTTTACTACTTTTATTCTTTACA

ATCTTAGTTCTCTTCTTTCCTGACTTACTTGGAGACCCAGACAATTATACACCCNCCAAC

CCACTTAACACCCCTCCTCATATTAAACCTGAATGATACTTCTTATTTGCCTACGCTATT

CTTCGCTCTATCCCCAATAAACTAGGCGGAGTACTAGCCCTAGTACTCTCAATTCTTATC

TTAATTTTCTNACCACTAATCCATACATCAAAACAACGAAGTCTAATATTCCGACCAATT

TCACAAATACTTTACTGAATCTTAATCGCTAATTTAATTATTCTTACATGAATTGGAGGG

CAACCAGTAGAACACCCATTCATCATCATCGGTCAACTAGCCTCAATCAGTTATTTTTCC

ATTATTTTAATTCTTCTACCAATCGCAGGCATTATCGAAGA-------------------

>M5927

NNNNNNNNNNNNNNNNAAANTCCACCCCCTATTAAAANTAGTAAACCACTCATTCATTGAC

CTCCCTACTCCTCCCAACATTTCATCCTGATGAAACTTTGGATCACTCCTAGGAATTTGC

TTAGCAATTCAAATTGCAACAGGATTCTTTTTAGCCATACATTATACAGCAGATACAACA

ACAGTTTTTTCATCAGTATCCCACATNTGCCGAGACGTAAATTGGGGAAGACTAATTCGT

TATATACGCGCAAGGGGAGCCTCAATATTCTTCATCTGCCTATTTATCCATAGCGGAAGA

GGAATTTACTGAGGATCCTACCTCTTTCAAGAAACATGAAACATCGGTGTACTCCTCCTA

TTTGCCGTAATAGCTAGCGCATTCATGGGTTACGTCCTACCATGAGGACAAATATCCTTC

AGAGGCGCCACAGTCATTACAAACCTCCTCTCAGCAATCCCATNCACTGGCCCAACAATT

GTAGAATGAATCTGAGGAGGCTTCTCAGTAGACAAAGCCACTCTAACACGATTCTTCGCA

TTCCACTTTATCCTTCCCTTTATTATTACAGCTCTCGTCCTAGTCCACCTCTTATTTCTC

CACGAAACTGGATCCAATAACCCTCTAGGACTAAACTCCAACGCAGACAAAATCCCCTTT

CACCCCTATTATACAGTAAAAGATTTTCTCGGAGTAATCTTACTACTTCTATTCTTCACA

ATTTTAGTCCTCTTCTTCCCCGACCTACTTGGAGACCCAGACAATTACACACCCGCCAAC

CCACTTAATACTCCCCCTCATATTAAACCCGAATGATATTTCTTATTTGCCTACGCTATT

CTCCGCTCCATTCCCAATAAACTAGGAGGAGTACTAGCCCTAGCACTTTCAATCCTCATT

CTAATTTTCCTACCACTAATCCACACATCAAAACAACGAAGTCTAATATTCCGATCAATT

TCACAAATACTATAATGAATTTTAATCGCCNACCTAATTATCCTCACATGAATTGGAGGT

CAACCAGTAGAACATCCATTTATCATCATTGGCCAACTAGCCTCAATCAGCTATTTCTCC

ATCATCCTAATCTTTCTACCAATCGCAGGGATCATCGAAGA-------------------

>M5930

NNNNNNNNNNNNNNNNAAAATGCNNNNCCNACTAAAAATAGTAAACCACTCATTCATCGAC

CTCCCCACTCCTCCCAACATCTCATCCTGATGAAACTTTGGATCACTTCTAGGAATTTGC

TTAGTAATTCAAATTGCTACAGGATTTTTTTTAGCCATACATTATACAGCAGACACAACA

ACAGTTTTTTCATCAGTATCCCATATTTGCCGAGACGTAAATTGGGGAAGACTACTCCGT

TACATACGCGCAAGGGGAGCCTCACTATTCTTCATGTGCCTATTTATCCATAGGGGAAGA

GGCATTTACTGGGGATCCTACATCTTCCAAGAAACATGAAACATCGGTGTAATCCTCCTA

TTTGCCGTAATAGCTAGCGCATNCATAGGGTACGTCCTACCNTGAGGACAAATATCCTTC

AGAGGAGCCACAGTAATTACAAATCTCCTCTCAGCAATCCCATATACTGGCCCAACAATT

GTAGAATGAATCTGAGGGGGGTTCTCAGTAGACAAAGCCACCCTAACACGATTTTTCGCA

TTCCACTTTATCCTCCCTTTTATTATTACAGCCCTTGTCCTAGTCCACCTCCTATTTCTT

CGCGAAACCGGATCCAATAACCCCCTAGGACTAAGCTCTAATGCAGACAAAATCCCCTTT

CACCCCTATTACACAGTAAAAGATTTTCTCGGAGTAATTTTACTACTTCTATTCTTCATA

ATCCTAGTTCTCTTCTTTCCYGACCTACTTGGAGACCCAGACAATTACACACCTGCTAAT

CCACTCAACACACCTCCCCATATTAAACCCGAATGATATTTCTTATTTGCCTACGCTATT

CTCCGTTCCATTCCCAATAAACTAGGAGGAGTACTAGCCTTAGTATTATCAATCCTCATT

CTAATTTTCCTACCACTAATCNACACATCAAAACAACGAAGCCTAATATTCCGACCAATT

TCACAAATACTATATTGAATTTTATTCGCTAACCTAATTATCCAAACATGAATCGGGGGT

CAACCAGTAGAACATCCATTTATCATTATTGGCCAACTAGCCTCAATCAGCTATTTTTCT

ATCATCCTAATCTTTCTACCAATCGCAGGAATCATCGAAGA-------------------

>M5933

NNNNNNNNNNNNNNNNAAAATTCACCCCCTAATAAAAATAGTAAACCACTCATTCATTGAC

CTCCCTACTCCTCCAAACATCTCATCCTGATGAAACTTTGGCTCACTCCTAGGGATTTGC

TTAGTAATTCAAATTACTACAGGACTCTTCCTAGCCATACACTATACAGCAGATACAACA

ACAGCATTTTCATCAGTATCCCACATCTGCCGAGACGTAAATTACGGATGACTAATTCGT

TATATACACGCAAACGGAGCCTCAATATTCTTCATCTGCCTATTTATCCATATCGGACGA

GGAATTTACTACGGATCCTACATCTTTCAAGAAACATGAAACATCGGTGTAATCCTCCTA

TTTGCCGTAATAGCTACCGCATTCATGGGTTACGTCCTACCATGAGGACAAATATCCTTC

TGAGGCGCCACAGTCATTACAAACCTCCTCTCAGCAATCCCATACATTGGCCCAACAATT

GTAGAATGAATCTGAGGAGGCTTCTCAGTAGACAAAGCCACTCTAACACGATTCTTCGCA

TTCCACTTTATCCTTCCCTTTATTATTACAGCTCTCGTCCTAGTCCACCTCTTATTTCTC

CACGAAACTGGATCCAATAACCCTCTAGGACTAAACTCCAACGCAGACAAAATCCCCTTT

CACCCCTATTATACAGTAAAAGATTTTCTCGGAGTAATCTTACTACTTCTATTCTTCACA

ATTTTAGTCCTCTTCTTCCCCGACCTACTTGGAGACCCAGACAATTACACACCCGCCAAC

CCACTTAATACTCCCCCTCATATTAAACCCGAATGATATTTCTTATTTGCCTACGCTATT

CTCCGCTCTATTCCCAATAAACTAGGTGGAGTACTAGCCCTAGTACTTTCAATCCTCATT

CTAATTTTCCTACCACTAATCCATACATCAAAACAACGAAGCCTAATATTCCGACCAATT

TCACAAATACTCTACTGAATTTTAATCGCCAACCTACTTATCCTCACATGAATTGGAGGT

CAACCAGTAGAACACCCATTTATCATTATTGGCCAACTAGCCTCAATTAGCTATTTCTCC

ATCATCCTAATCTTTCTACCAATCGCAGGGNNTCTCGAAGA------------------

>M5935

NNNNNNNNNNNNNNNNAAACTNCNCCCCTTACTAAAAATAGTAAACCACTCATTCATCGAC

CTCCCCACTCCTCCAAACATCTCATCCTGATGAAACTTTGGCTCACTTCTAGGAATTTGC

TTAGTAATTCAAATTGCTACAGGACTTTTCCTAGCCATACATTATACAGCAGACACAACA

ACAGCATTTTCATCAGTATCCCATATCTGCCGAGACGTAAATTACGGAKGACTAATCCGT

TATATACACGCAAACGGAGCCTCACTATTCTTCATCTGCCTATTTATCCATATCGGACGA

GGCATTTACTACGGATCCTACATCTTCCAAGAAACATGAAACATCGGTGTAATCCTCCTA

TTTGCCGTAATAGCTACCGCATTCATAGGGTACGTCCTACCATGAGGACAAATATCCTTC

TGAGGAGCCACAGTAATTACAAATCTCCTCTCAGCAATCCCATATATTGGCCCAACAATT

GTAGAATGAATCTGAGGGGGGTTCTCAGTAGACAAAGCCACCCTAACACGATTTTTCGCA

TTCCACTTTATCCTCCCTTTTATTATTACAGCTCTTGTCCTAGTCCACCTCCTATTTCTT

CACGAAACCGGATCCAATAACCCCCTAGGACTAAACTCTAATGCAGACAAAATCCCCTTT

CACCCCTATTACACAGTAAAAGATTTTCTCGGAGTAATTTTACTACTTCTATTCTTCATA

ATCCTAGTTCTCTTCTTTCCTGACCTACTTGGAGACCCAGACAATTACACACCTGCTAAC

CCACTCAACACACCTCCCCATATTAAACCCGAATGATACTTCTTATTTGCCTACGCTATT

CTCCGTTCCATCCCCAATAAACTAGGTGGAGTACTAGCCTTAGTATTATCAATCCTCATT

CTAATTTTCCTACCACTAATCCATACATCAAAACAACGAAGCCTAATATTCCGACCAATT

TCACAAATACTTTACTGAATTTTAATCGCTAACCTGCTTATCCTTACATGAATCGGGGGC

CAACCAGTAGAACACCCATTTATCATTATTGGCCAACTAGCCTCAATTAGCTATTTTTCT

ATCATCCTAATCTTTCTACCAATCGCAGGAATCATCGAAGA------------------

>M5942

NNNNNNNNNNNNNNNNAAAATTCACCCCCTAATAAAAATAGTAAACCACTCATTCATTGAC

CTCCCTACTCCTCCAAACATCTCATCCTGATGAAACTTTGGCTCACTCCTAGGAATTTGC

TTAGTAATTCAAATTACTTCAGGACTCTTCCTAGCCATACACTATACAGCAGATACAACA

ACAGCATTTTCATCAGTATCCCACATCTGCCGAGACGTAAATTACGGATGACTAATTCGT

TATATACACGCAAACGGAGCCTCAATATTCTTCATCTGCCTATTTATCCATATCGGACGA

GGAATTTACTACGGATCCTACATCTTTCAAGAAACATGAAACATCGGCGTAATCCTCCTA

TTCGCCGTAATAGCTACCGCATTCATGGGTTATGTCCTACCATGAGGACAAATATCCTTC

TGAGGCGCCACAGTCATTACAAACCTCCTCTCAGCAATCCCATACATTGGCCCAACAATT

GTAGAATGAATCTGAGGAGGCTTCTCAGTAGACAAAGCCACTCTAACACGATTCTTCGCA

TTCCACTTTATCCTTCCCTTTATTATTGCAGCTCTCGTCCTAGTCCACCTCTTATTTCTC

CACGAAACTGGATCCAATAACCCTCTAGGACTAAACTCCAACGCAGACAAAATCCCCTTT

CACCCCTATTATACAGTAAAAGATTTTCTCGGAGCAATCTTACTACTTCTATTCTTCACA

ATTTTAGTCCTCTTCTTCCCCGACCTACTTGGAGACCCAGACAATTACACACCCGCCAAC
CCACTTAATACTCCCCCTCATATTAAACCCGAATGATATTTCTTATTTGCCTACGCTATT
CTCCGCTCTATTCCCAATAAACTAGGTGGAGTACTAGCCCTAGTACTTTCAATCCTCATT
CTAATTTTCCTACCACTAATCCATACATCAAAACAACGAAGCCTAATATTCCGACCAATT
TCACAAATACTCTACTGAATTTTAATCGCCAACCTACTTATCCTCACATGAATTGGAGGT
CAACCAGTAGAACACCCATTTATCATTATTGGCCAACTAGCCTCAATTAGCTATTTCTCC
ATCATCCTAATCTTTCTACCAATCGCAGGGAATATCGAAGA------------------
>M5943
NNNNNNNNNNNNNNNAAAATTCACCCCCTATTAAAAATAGTAAACCACTCATTCATCGAC
CTCCCCACTCCTCCAAACATCTCATCCTGATGAAACTTTGGCTCACTTCTAGGAATTTGC
TTAGTAATTCAAATTGCTACAGGACTTTTCCTAGCCATACATTATACAGCAGACACAACA
ACAGCATTTTCATCAGTATCCCATATCTGCCGAGACGTAAATTACGGATGACTAATCCGT
TACATACACGCAAACGGAGCCTCACTATTCTTCATCTGCCTATTTATCCATATCGGACGA
GGCATTTACTACGGATCCTACATCTTCCAAGAAACATGAAACATCGGTGTAATCCTCCTA
TTTGCCGTAATAGCTACCGCATTCATAGGGTACGTCCTACCATGAGGACAAATATCCTTC
TGAGGAGCCACAGTGATTACAAATCTCCTCTCAGCAATCCCGTATATTGGTCCAACAATT
GTAGAATGAATCTGAGGGGGGTTCTCAGTAGACAAAGCCACCCTAACACGATTTTTCGCA
TTCCATTTTATCCTCCCTTTTATTATTACAGCCCTTGTCCTAGTCCACCTCCTATTTCTT
CACGAAACCGGATCCAATAACCCCCTAGGACTAAACTCTAATGCAGACAAAATCCCCTTT
CACCCCTATTACACAGTAAAAGATTTTCTCGGAGTAATCTTACTACTTCTATTCTTCATA
ATCCTAGTTCTCTTCTTTCCTGACCTACTTGGAGACCCAGACAATTACACACCCGCTAAC
CCACTCAACACACCTCCCCATATTAAACCCGAATGATATTTCTTATTTGCCTACGCTATT
CTCCGTTCCATCCCCAATAAACTAGGTGGAGTACTAGCCTTAGCATTATCAATCCTCATT
CTAATTTTCCTACCACTAATCCATACATCAAAACAACGAAGCCTAATATTCCGACCAATT
TCACAAATACTTTACTGAATTTTAATCGCTAACCTGCTTATCCTTACATGAATCGGGGGC
CAACCAGTAGAACACCCATTTATCATTATTGGCCAACTAGCCTCAATTAGCTATTTTTCT
ATCATCCTAATCTTTCTACCAATCGCAGGAATCATCGAAGA------------------
>M5946
NNNNNNNNNNNNNNNAAAATTCACCCCCTAATAAAAATAGTAAACCACTCATTCATTGAC
CTCCCTACTCCTCCAAACATCTCATCCTGATGAAACTTTGGCTCACTCCTAGGAATTTGC
TTAGTAATTCAAATTACTACAGGACTCTTCCTAGCCATACACTATACAGCAGATACAACA
ACAGCATTTTCATCAGTATCCCACATCTGCCGAGACGTAAATTACGGATGACTAATTCGT
TATATACACGCAAACGGAGCCTCAATATTCTTCATCTGCCTATTTATCCATATCGGACGA
GGAATTTACTACGGATCCTACATCTTTCAAGAAACATGAAACATCGGTGTAATCCTCCTA
TTTGCCGTAATAGCTACCGCATTCATGGGTTACGTCCTACCATGAGGACAAATATCCTTC
TGAGGCGCCACAGTCATTACAAACCTCCTCTCAGCAATCCCATACATTGGCCCAACAATT
GTAGAATGAATCTGAGGAGGCTTCTCAGTAGACAAAGCCACTCTAACACGATTCTTCGCA
TTCCACTTTATCCTTCCCTTTATTATTACAGCTCTCGTCCTAGTCCACCTCTTATTTCTC
CACGAAACTGGATCCAATAACCCTCTAGGACTAAACTCCAACGCAGACAAAATCCCCTTT
CACCCCTATTATACAGTAAAAGATTTTCTCGGAGTAATCTTACTACTTCTATTCTTCACA
ATCTTAGTCCTCTTCTTTCCCGACCTACTTGGAGACCCAGACAATTACACACCCGCCAAC
CCACTTAATACTCCCCCTCATATTAAACCCGAATGATATTTCTTATTTGCCTACGCTATT
CTCCGCTCTATTCCCAATAAACTAGGTGGAGTACTAGCCCTAGTACTTTCAATCCTCATT
CTAATTTTCCTACCACTAATCCATACATCAAAACAACGAAGCCTAATATTCCGACCAATT
TCACAAATACTCTACTGAATTTTAATCGCCAACCTACTTATCCTCACATGAATTGGAGGT
CAACCAGTAGAACACCCATTTATCATTATTGGCCAACTAGCCTCAATTAGCTATTTCTCC
ATCATCCTAATCTTTCTACCAATCGCAGGGATCATCGAAGA------------------
>M5951
NNNNNNNNNNNNNNNNNNNNNNNNNNNCTACTAAAAATAGTTAACCACTCATTCATTGAT
CTCCCCACTCCCCCTAATATTTCATCTTGATGAAACTTTGGCTCACTCCTAGGAATTTGC
TTAATAATTCAAATCGCTACAGGACTTTTCCTAGCCATACATTATACAGCAGACACAACA

ACAGCATTCTCATCAGTATCCCATATCTGCCGAGACGTCAATTATGGATGACTAATACGC

TATATACATGCAAACGGAGCCTCAATATTCTTTATTTGCCTATTTATTCATATCGGACGA

GGAATTTACTACGGATCTTATATCTTTCAAGAAACATGAAACATTGGAGTAATTCTCTTA

TTTGCCGTAATAGCCACCGCATTCATAGGGTATGTACTTCCATGAGGACAAATATCCTTC

TGAGGGGCCACAGTCATTACAAATCTTCTTTCAGCTATTCCATATATTGGCCCANCAATC

GTANAATGAATNTGAGGNNGNTTTNCAGNAGACAANGCCACTTTAACACGATTTTTCGCA

TTTCACTTCATTCTCCCCTTTATTATCACAGCCTTAGTCCTAGTCCATCTCCTATTCCTT

CACGAAACCGGATCTAATAATCCCCTAGACCTTAACTCCGACTAAGACAAAATCCCTTTT

CACCCATACTACACAGTAAAAGATTTTCTCGGAGTAATTCTACTACTTCTATTTTTCACA

ATTTTAGTCCTCTTCTTCCCTGACTTACTTGGAGATCCAGACAACTACACACCCGCTAAC

CCCCTTAACACTCCCCCCCACATTAAACCCGAATGATATTTCTTATTTGCCTATGCTATC

CTACGTTCAATTCCTAACAAACTAGGAGGAGTCCTAGCCCTAGTACTTTCAATCCTTATC

CTAATTTTTTTACCACTCATTCACACATCAAAACAACGAAGCCTAATATTCCGACCAATT

TCCCAAATACTTTACTGAATTTTAATCGCCAACCTACTTATCCTCACATGAATCGGAGGC

CAACCAGTAGAACACCCATTTATCATCATTGGCCAACTAGCCTCAATCAGTTATTTTTCN

ATTATCCTAATTTTTTTACCAATCGCAGGAATCATTGAAGN-------------------

>M5968

NNNNNNNNNNNNNNNNAAAATTCACCCCCTAATAAAAATAGTAAACCACTCATTCATTGAC

CTCCCTACTCCTCCAAACATCTCATCCTGATGAAACTTTGGCTCACTCCTAGGAATTTGC

TTAATAATTCAAATTACTACAGGACTCTTCCTAGCCATACACTATACAGCAGATACAACA

ACAGCATTTTCATCAGTATCCCACATCTGCCGAGACGTAAATTACGGATGACTAATTCGT

TATATACACGCAAACGGAGCCTCAATATTCTTCATCTGCCTATTTATCCATATCGGACGA

GGAATTTACTACGGATCCTACATCTTTCAAGAAACATGAAACATCGGTGTAATCCTCCTA

TTTGCCGTAATAGCTACCGCATTCATGGGTTACGTCCTACCATGAGGACAAATATCCTTC

TGAGGCGCCACAGTCATTACAAACCTCCTCTCAGCAATCCCATACATTGGCCCAACAATT

GTAGAATGAATCTGAGGAGGCTTCTCAGTAGACAAAGCCACTCTAACACGATTCTTCGCA

TTCCACTTTATCCTTCCCTTTATTATTACAGCTCTCGTCCTAGTCCACCTCTTATTTCTC

CACGAAACTGGATCCAATAACCCTCTAGGACTAAACTCCAACGCAGACAAAATCCCCTTT

CACCCCTATTATACAGTAAAAGATTTTCTCGGAGTAATCTTACTACTTCTATTCTTCACA

ATTTTAGTCCTCTTCTTCCCCGACCTACTTGGAGACCCAGACAATTACACACCCGCCAAC

CCACTTAATACTCCCCCTCATATTAAACCCGAATGATATTTCTTATTTGCCTACGCTATT

CTCCGCTCTATTCCCAATAAACTAGGTGGAGTACTAGCCCTAGTACTTTCAATCCTCATT

CTAATTTTCCTACCACTAATCCATACATCAAAACAACGAAGCCTAATATTCCGACCAATT

TCACAAATACTCTACTGAATTTTAATCGCCAACCTACTTATCCTCACATGAATTGGAGGT

CAACCAGTAGAACACCCATTTATCATTATTGGCCAACTAGCCTCAATTAGCTATTTCTCC

ATCATCCTAATCTTTCTACCAATCGCAGGGATCATCGAAGA-------------------

>M5972

NNNNNNNNNNNNNNNNNAAAATNNNNNNATTACTAAAAATAGTAAACCACTCTTTCTTTGAT

CTCCCCACCCCCCCCAATATTTCATCCTGATGAAACTTTGGCTCACTCCTAGGAATTTGC

CTAGTAATTCAAATTGCTACAGGATTCTTTCTAGCCATACATTACACAGCAGACACAACA

ACAGTATTCTCATCAGTATCTCACCTNTGCAGAGATGTAAAATGGGGAAGACTAATCCGT

TATATACGCGCCAAAGGAGCCTCAATATTCTTTATTTGTTTATTCATTCACATTGGACGA

AGTATCTACTAGGGATCATACATTTTTCAAGAAACATGAAACATCGGTGTAATTCTCCTA

TTCGCTGAGATACCCACTGCATTCATAGGATATGTTCTACCATGAGGACAAATATCCTTC

AGAGGAGCCACAGTCATCACAAACCTTCTTTCAGCAATACCATATATTGGCCCAACAATT

GTAGAATGAATCTGAGGAGGCTTTTCAGTAGATAAAGCCACTCTAACACGATTCTTCGCA

TTCCACTTTATTCTTCCCTTTATTATCACAGCCCTTGTCCTAGTCCATCTCTTATTCCTC

CACGAAACTGGATCTAACAATCCCCTAGGACTTAACTCCAACGCAGATCAAATTCCCTTC

CACCCATACTACACAGTAAAAGATTTTCTTGGAGTAATCTTACTACTTTTATTCTTTACA

ATCTTAGTTCTCTTCTTTCCTGACTTACTTGGAGACCCAGACAATTAYACACCCNCCAAC

CCACTTAACACCCCTCCTCACATTAAACCTGAATGATATTTCTTATTTGCCTACGCTATT

CTCCGCTCTATCCCCAATAAACTAGGGGGAGTGCTAGCCCTAGTACTCTCAATTCTTATT

TTAATTTTTTTACCACTAATCCACACATCAAAACAACGAAGTCTAATATTCCGACCAATT

TCCCAAATACTTTACTGAATCTTAATCGCCAACCTACTTATTCTCACATGAATTGGAGGT

CAACCAGTAGAACACCCATTCATCATCATTGGCCAACTAGCCTCAATCAGTTATTTTTCC

ATCATCCNTATTTTCCTACCAATCGCAGGCATTATCGAAGG------------------

>M5975

NNNNNNNNNNNNNNNAAANTTCACCCCCTAATAAAAATAGTAAACCACTCATTCATTGAC

CTCCCTACTCCTCCCAACATCTCATCCTGATGAAACTTTGGATCACTCCTAGGAATTTGC

TTAGTAATTCAAATTGCTACAGGACTCTTCCTAGCCATACACTATACAGCAGATACAACA

ACAGTTTTTTCATCAGTATCCCACATCTGCCGAGACGTAAATTAGGGATGACTAATTCGT

TATATACGCGCAAGCGGAGCCTCAATATTCTTCATCTGCCTATTTATCCATAGCGGAAGA

GGAATTTACTRCGGATCCTACATCTTTCAAGAAACATGAAACATCGGTGTAATCCTCCTA

TTTGCCGTAATAGCTACCGCATTCATGGGTTACGTCCTACCATGAGGACAAATATCCTTC

AGAGGCGCCACAGTCATTACAAACCTCCTCTCAGCAATCCCATACATTGGCCCAACAATT

GTAGAATGAATCTGAGGAGGCTTCTCAGTAGACAAAGCCACTCTAACACGATTCTTCGCA

TTCCACTTTATCCTTCCCTTTATTATTACAGCTCTCGTCCTAGTCCACCTCTTATTTCTC

CACGAAACTGGATCCAATAACCCTCTAGGACTAAACTCCAACGCAGACAAAATCCCCTTT

CACCCCTATTATACAGTAAAAGATTTTCTCGGAGTAATCTTACTACTTCTATTCTTCACA

ATTTTAGTCCTCTTCTTCCCCGACCTACTTGGAGACCCAGACAATTACACACCCGCCAAC

CCACTTAATACTCCCCCTCATATTAAACCCGAATGATATTTCTTATTTGCCTACGCTATT

CTCCGCTCTATTCCCAATAAACTAGGTGGAGTACTAGCCCTAGTACTTTCAATCCTCATT

TTAATTTTCCTACCACTAATCCATACATCAAAACAACGAAGCCTAATATTCCGACCAATT

TCACAAATACTATACTGAATTTTAATCGCCAACCTACTTATCCTCACATGAATTGGGGGT

CAACCAGTAGAACACCCATTTATCATTATTGGCCAACTAGCCTCAATTAGCTATTTCTCC

ATCATCCTAATCTTTCTACCAATCGCAGGGATCATCGAAGA------------------

>M5976

NNNNNNNNNNNNNNNNNNAATTCACCCACTATTAAAAATAGTTAACCACTCATTCATTGAT

CTTCCCACTCCCCCTAACATTTCATCTTGATGAAACTTTGGATCACTCCTAGGAATTTGC

TTAACAATTCAAATCGCTACAGGACTTTTCCTAGCCATACATTATACAGCAAACACAACA

ACAGCATTCTCATCAGTATCCCATATCTGCCGAGACGTCAATTATGGAAGACTAATCCGC

TATATACATGCAAACGGAGCTTCAATATTCTTTATTTGCCTATTCATTCACATCGGACGA

GGAATTTACTACGGATCTTATATCTTTCAAGAAACATGAAACATTGGGGTAATTCTCTTA

TTTGCCGTAATAGCCACCGCATTCATAGGGTATGTACTTCCATGAGGACAAATATCCTTC

AGAGGGGCCACAGTCATTACAAATCTTTTTTCAGCTATTCCATATATTGGCCCAACAATC

GTAGAGTGAATTTGAGGAGGATTTNCAGTGGACAAAGCCACTTTAACACGATTTTTCGCA

TTTCACTTCATTCTCCCCTTTATTATCACAGCCTTAGTCCTAGTCCATCTCCTATTCCTT

CACGAAACCGGATCTAATAATCCCCTAGGCCTTAACTCCAACTCAGACAAAATCCCCTTT

CACCCATACTACACAGAAAAAGATTCTCTCGGAGTAATTCTACTACTTCTATTTTTCACA

ATTTTAGTCCTCTTCTTCCCTGACTTACTTGGAGATCCAGACAACTACACACCCGCCAAC

CCCCTTAACACTCCCCCCCACATTAAACCCGAATGATATTTCTTATTTGCCTATGCTATC

CTWCGTTCAATTCCTAACAAACTAGGAGGAGTCCTAGCCCTAGTACTTTCAATCCTTATC

CTAATTTTTTTACCACTCATTCACACATCAAAACAACGAAGCCTAATATTCCGACCTATT

TCCCAAATACTTTACTGAATTTTAATCGCCAACCTACTTATCCTCACATGAATCGGAGGC

CAACCAGTAGAACACCCATTTATCATCATTGGCCAACTAGCCTCAATCAGTTACTTTTCC

ATCATCCTAATCCTTTTACCAATCGCAGGAATCATTGAAGA------------------

>M5977

ATGACAAACATCCGAAAAATTCACCCCCTACTAAAAATAGTAAACCACTCATTCATCGAC

CTCCCCACTCCTCCAAACATCTCATCCTGATGAAACTTTGGCTCACTTCTAGGAATTTGC

TTAGTAATTCAAATTGCTACAGGACTTTTCCTAGCCATACATTATACAGCAGACACAACA

ACAGCATTTTCATCAGTATCCCATATCTGCCGAGACGTAAATTACGGATGACTAATCCGT

TATATACACGCAAACGGAGCCTCACTATTCTTCATCTGCCTATTTATCCATATCGGACGA

GGCATTTACTACGGATCCTACATCTTCCAAGAAACATGAAACATCGGTGTAATCCTCCTA

TTTGCCGTAATAGCTACCGCATTCATAGGGTACGTCCTACCATGAGGACAAATATCCTTC

TGAGGAGCCACAGTAATTACAAATCTCCTCTCAGCAATCCCATATATTGGTCCAACAATT

GTAGAATGAATCTGAGGGGGGTTCTCAGTAGACAAAGCCACCCTAACACGATTTTTCGCA

TTCCATTTTATCCTCCCTTTTATTATTACAGCCCTTGTCCTAGTCCACCTCCTATTTCTT

CACGAAACCGGATCCAATAACCCCCTAGGACTAAACTCTAATGCAGACAAAATCCCCTTT

CACCCCTACTACACAGTAAAAGATTTTCTCGGAGTAATTTTACTACTTCTATTCTTCATA

ATCCTAGTTCTCTTCTTTCCTGACCTACTTGGAGACCCAGACAATTACACACCTGCTAAC

CCACTCAACACACCTCCCCATATTAAACCCGAATGATATTTCTTATTCGCCTACGCTATT

CTCCGTTCCATCCCCAATAAACTAGGTGGAGTACTAGCCTTAGTATTATCAATCCTCATT

CTAATTTTCCTACCACTAATCCATACATCAAAACAACGAAGCCTAATATTCCGACCAATT

TCACAAATACTTTACTGAATTTTAATCGCTAACCTGCTTATCCTTACATGAATCGGGGGC

CAACCAGTAGAACACCCATTTATCATTATTGGCCAACTAGCCTCAATTAGCTATTTTTCT

ATCATCCTAATCTTTCTACCAATCGCAGGAATCATCGAAGACAAAATATTAAAATTATAT

>LG137

NNNNNNNNNNNNNNNNAAATTCCACCCCCTACTAAAAATAGTAAACCACTCATTCATCGAC

CTCCCCACTCCTCCAAACATCTCATCCTGATGAAACTTTGGCTCACTTCTAGGAATTTGC

TTAGTAATTCAAATTGCTACAGGATTTTTTTAGCCATACATTATACAGCAGACACAACA

ACAGCATTTTCATCAGTATCCCATATGTGCCGAGACGTAAATTGGGGATGACTAATCCGT

TATATACACGCAAACGGAGCCTCACTATTCTTCATCTGCCTATTTATCCATATCGGACGA

GGCATTTACTACGGATCCTACATCTTCCAAGAAACATGAAACATCGGTGTAATCCTCCTA

TTTGCCGTAATAGCTACCGCATTCATAGGGTACGTCCTACCATGAGGACAAATATCCTTC

TGAGGAGCCACAGTGATTACAAATCTCCTCTCAGCAATCCCATATATTGGTCCAACAATT

GTAGAATGAATCTGAGGGGGATTCTCAGTAGACAAAGCCACCCTAACACGATTTTTCGCA

TTCCATTTTATCCTCCCTTTTATTATTACAGCCCTCATCCTAGTCCACCTCCTATTTCTC

CACGAAACCGGATCCAATAACCCCCTAGGACTAAACTCTAATGCAGACAAAATCCCCTTT

CACCCCTATTACACAGTAAAAGATTTTCTCGGAGTAATTTTACTACTTCTATTTTTCATA

ATCCTAGTTCTCTTCTTTCCTGACTTACTTGGAGACCCAGACAATTACACACCTGCTAAC

CCACTCAACACACCTCCCCATATTAAACCCGAATGATATTTCTTATTTGCCTACGCTATT

CTCCGTTCCATCCCCAATAAACTAGGTGGAGTACTAGCCTTAGTATTATCAATCCTCATT

CTAATTTTTCTACCACTAATTCACACATCAAAACAACGAAGCCTAATATTCCGACCAATT

TCACAAATACTTTACTGAATTTTAATCGCTAACCTGCTTATCCTTACATGAATCGGAGGC

CAACCAGTAGAACACCCGTTTATCATTATTGGCCAACTAGCCTCAATTAGCTATTTTTCT

ATCATCCTAACTTTCTACCCAATCGCAGGAACTATCGAAGACAAA----------TATAT

>M5983

NNNNNNNNNNNNNNNNAAAATTAACCCCCTATTAAAAATAGTAAACCACTCATTCATCGAC

CTCCCCACTCCTCCAAACATTTCATCCTGATGAAACTTTGGCTCACTTCTAGGAATTTGC

TTAGTAATTCAAATTGNTACAGGACTTTTCCTAGCCATACATTATNCAGCAGACACAACA

MCAGCATTTTCATCAGTATCCCATATCTGCCGAGACGTAAATTACGGATGACTAATCCGT

TACATACACGCAAACGGAGCCTCACTATTCTTCATCTGCCTATTTATCCATATCGGACGA

GGCATTTACTACGGATCCTACATCTTCCAAGAAACATGAAACATCGGTGTAATCCTCCTA

TTTGCCGTAATAGCTACCGCATTCATAGGGTACGTCCTACCATGAGGACAAATATCCTTC

TGAGGAGCCACAGTGATTACAAATCTCCTCTCAGCAATCCCGTATATTGGTCCAACAATT

GTAGAATGAATCTGAGGGGGGTTCTCAGTAGACAAAGCCACCCTAACACGATTTTTCGCA

TTCCATTTTATCCTCCCTTTTATTATTACAGCCCTTGTCCTAGTCCACCTCCTATTTCTT

CACGAAACCGGATCCAATAACCCCCTAGGACTAAACTCTAATGCAGACAAAATCCCCTTT

CACCCCTATTACACAGTAAAAGATTTTCTCGGAGTAATCTTACTACTTCTATTCTTCATA

ATCCTAGTTCTCTTCTTTCCTGACCTACTTGGAGACCCAGACAATTACACACCCGCTAAC

CCACTCAACACACCTCCCCATATTAAACCCGAATGATATTTCTTATTTGCCTACGCTATT

CTCCGTTCCATCCCCAATAAACTAGGTGGAGTACTAGCCTTAGTATTATCAATCCTCATT

CTAATTTTCCTACCACTAATCCATACATCAAAACAACGAAGCCTAATATTCCGACCAATT

TCACAAATACTTTACTGAATTTTAATCGCTAACCTGGTTATCCTTACATGAATCGGGGGC

CAACCAGTAGAACACCCATTTATCATTATTGGTCAACTAGCCTCAATTAGCTATTTTTCT

ATCATCCTAATCTTTCTACCAATCGCAGGAATCATCGAAGA-------------------

>M5984

NNNNNNNNNNNNNNNNAAAATTCACCCCCTACTAAAAATAGTAAACCACTCATTCATCGAC

CTCCCCACTCCTCCAAACATCTCATCCTGATGAAACTTTGGCTCACTTCTAGGAATTTGC

TTAGTAATTCAAATTGCTACAGGACTTTTCCTAGCCATACATTATACAGCAGACACAACA

ACAGCATTTTCATCAGTATCCCATATCTGCCGAGACGTAAATTACGGATGACTAATCCGT

TATATACACGCAAACGGAGCCTCACTATTCTTCATCTGCCTATTCATCCATATCGGACGA

GGCATTTACTACGGATCCTACATCTTCCAAGAAACATGAAACATCGGTGTAATCCTCCTA

TTTGCCGTAATAGCTACCGCATTCATAGGATACGTCCTACCATGAGGACAAATATCCTTC

TGAGGAGCCACAGTGATTACAAATCTCCTCTCAGCAATCCCGTATATTGGTCCAACAATT

GTAGAATGAATCTGAGGGGGGTTCTCAGTAGACAAAGCCACCCTAACACGATTTTTCGCA

TTCCATTTTATCCTCCCTTTTATTATTACAGCCCTTGTCCTAGTCCACCTCCTATTTCTT

CACGAAACCGGATCCAATAACCCCCTAGGACTAAACTCTAATGCAGACAAAATCCCCTTT

CACCCCTATTACACAGTAAAAGATTTCCTCGGAGTAATTTTACTACTTTTATTCTTCATA

ATCCTAGTTCTCTTCTTTCCTGACCTACTTGGAGACCCAGACAATTACACACCTGCTAAC

CCACTCAACACACCTCCCCATATTAAACCCGAATGATATTTCTTATTTGCCTACGCTATT

CTCCGTTCCATCCCCAATAAACTAGGTGGAGTACTAGCCTTAGTATTATCAATCCTCATT

CTAATTTTCCTACCACTAATCCATACATCAAAACAACGAAGCCTAATATTCCGACCAATT

TCACAAATACTTTACTGAATTTTAATCGCTAACCTGCTTATCCTTACATGAATCGGGGGC

CAACCAGTAGAACACCCATTTATCATTATTGGCCAACTAGCCTCAATTAGTTATTTTTCT

ATCATCCTAATCTTTCTACCAATCGCAGGAATCATCGAAGA-------------------

>M6108

NNNNNNNNNNNNNNNNAAAATTCACCCCCTAATAAAAATAGTAAACCACTCATTCATTGAC

CTCCCTACTCCTCCCAACATCTCATCCTGATGAAACTTTGGATCACTCCTAGGAATTTGC

TTAGTAATTCAAATTGCTACAGGACTCTTCCTAGCCATACACTATACAGCAGATACAACA

ACAGTTTTTTCATCAGTATCCCACATCTGCCGAGACGTAAATTACGGATGACTAATTCGT

TATATACGCGCAAACGGAGCCTCAATATTCTTCATCTGCCTATTTATCCATATCGGAAGA

GGAATTTACTACGGATCCTACATCTTTCAAGAAACATGAAACATCGGCGTAATCCTCCTA

TTTGCCGTAATAGCTACCGCATTCATGGGTTATGTCCTACCATGAGGACAAATATCCTTC

TGAGGCGCCACAGTCATTACAAACCTCCTCTCAGCAATCCCATACATTGGCCCAACAATT

GTAGAATGAATCTGAGGAGGCTTCTCAGTAGACAAAGCCACTCTAACACGATTCTTCGCA

TTCCACTTTATCCTTCCCTTTATTATTGCAGCTCTCGTCCTAGTCCACCTCTTATTTCTC

CACGAAACTGGATCCAATAACCCTCTAGGACTAAACTCCAACGCAGACAAAATCCCCTTT

CACCCCTATTATACAGTAAAAGATTTTCTCGGAGTAATCTTACTACTTCTATTCTTCACA

ATTTTAGTCCTCTTCTTCCCCGACCTACTTGGAGACCCAGACAATTACACACCCGCCAAC

CCACTTAATACTCCCCCCCATATTAAACCCGAATGATATTTCTTATTTGCCTACGCTATT

CTCCGCTCTATTCCCAATAAACTAGGTGGAGTACTAGCCCTAGTACTTTCAATCCTCATT

CTAATTTTCCTACCACTAATCCATACATCAAAACAACGAAGCCTAATATTCCGACCAATT

TCACAAATACTATACTGAATTTTAATCGCCAACCTACTTATCCTCACATGAATTGGAGGT

CAACCAGTAGAACACCCATTTATCATTATTGGCCAACTAGCCTCAATCAGCTATTTCTCC

ATCATCCTAATCTTTCTACCAATCGCAGGAATCATCGAAGA-------------------

>M6135

NNNNNNNNNNNNNNNNAAAATTCACCCCCTACTAAAAATAGTAAACCACTCATTTATCGAT

CTCCCCACTCCTCCAAACATCTCATCCTGATGAAACTTTGGCTCACTTCTAGGAATTTGC

TTAGTAATTCAAATTGCTACAGGACTTTTCCTAGCTATACATTATACAGCAGACACAACA

ACAGCATTTTCATCAGTATCCCATATCTGCCGAGACGTAAATTACGGATGACTAATCCGT

TATATACACGCAAACGGAGCCTCACTATTCTTCATCTGCCTATTTATCCATATCGGACGA

GGCATTTACTACGGATCCTACATCTTCCAAGAAACATGAAACATCGGTGTAATCCTCCTA

TTTGCCGTAATAGCTACCGCATTCATAGGGTACGTCCTACCATGAGGACAAATATCCTTC

TGAGGAGCCACAGTGATTACAAATCTCCTCTCAGCAATCCCATATATTGGTCCAACAATT
GTAGAATGAATCTGAGGGGGATTCTCAGTAGACAAAGCCACCCTAACACGATTTTTCGCA
TTCCATTTTATCCTCCCTTTTATTATTACAGCCCTTGTCCTAGTCCACCTCCTATTTCTC
CACGAAACCGGATCCAATAACCCCCTAGGACTAAACTCTAATGCAGACAAAATCCCCTTT
CACCCCTATTACACAGTAAAAGATTTTCTCGGAGTAATTTTATTACTTCTATTCTTCATA
ATCCTAGTTCTCTTCTTTCCTGACCTACTTGGAGACCCAGACAATTACACACCTGCTAAC
CCACTCAACACACCTCCCCATATTAAACCCGAATGATATTTCTTATTTGCCTACGCTATT
CTCCGTTCCATCCCCAATAAACTAGGTGGAGTACTAGCCTTAGTATTATCAATCCTCATT
CTAATTTTCCTACCACTAATCCATACATCAAAACAACGAAGCCTAATATTCCGACCAATT
TCACAAATACTTTACTGAATTTTAATCGCTAACCTGCTTATCCTTACATGAATCGGAGGC
CAACCAGTAGAACACCCATTTATCATTATTGGCCAACTAGCCTCAATTAGCTATTTTTCT
ATCATCCTAATCTTTCTACCAATCGCAGGAATCATCGAAGA------------------
>M6133
NNNNNNNNNNNNNNNAAAATTCACCCCCTAATAAAAATAGTAAACCACTCATTCATTGAC
CTCCCTACTCCTCCCAACATCTCATCCTGATGAAACTTTGGATCACTCCTAGGAATTTGC
TTAGTAATTCAAATTGCTACAGGACTCTTCTTAGCCATACACTATACAGCAGATACAACA
ACAGCATTTTCATCAGTATCCCACATCTGCCGAGACGTAAATTACGGATGACTAATTCGT
TATATACACGCAAACGGAGCCTCAATATTCTTCATCTGCCTATTTATCCATATCGGACGA
GGAATTTACTACGGATCCTACATCTTTCAAGAAACATGAAACATCGGTGTAATCCTCCTA
TTTGCCGTAATAGCTACCGCATTCATGGGTTACGTCCTACCATGAGGACAAATATCCTTC
TGAGGCGCCACAGTCATTACAAACCTCCTCTCAGCAATCCCATACATTGGCCCAACAATT
GTAGAATGAATCTGAGGAGGCTTCTCAGTAGACAAAGCCACTCTAACACGATTCTTCGCA
TTCCACTTTATCCTTCCCTTTATTATTACAGCTCTCGTCCTAGTCCACCTCTTATTTCTC
CACGAAACTGGATCCAATAACCCTCTAGGACTAAACTCCAACGCAGACAAAATCCCCTTT
CACCCCTATTATACAGTAAAAGATTTTCTCGGAGTAATCTTACTACTTCTATTCTTCACA
ATTTTAGTCCTCTTCTTCCCCGACCTACTTGGAGACCCAGACAATTACACACCCGCCAAC
CCACTTAATACTCCCCCTCATATTAAACCCGAATGATATTTCTTATTTGCCTACGCTATT
CTCCGCTCTATTCCCAATAAACTAGGTGGAGTACTAGCCCTAGTACTTTCAATCCTCATT
CTAATTTTCCTACCACTAATCCATACATCAAAACAACGAAGCCTAATATTCCGACCAATT
TCACAAATACTCTACTGAATTTTAATCGCCAACCTACTTATCCTCACATGAATTGGAGGT
CAACCAGTAGAACACCCATTTATCATTATTGGCCAACTAGCCTCAATTAGCTATTTCTCC
ATCATCCTAATCTTTCTACCAATCGCAGGGATCATCGAAGA------------------
>M6134
-NNNNNNNNNNNNNNNNNNNNNNNNNNNNNNNNNNNNNNNNNNNNNCCCTCATTTATCGAT
CTCCCCACTCCTCCAAACATCTCATCCTGATGAAACTTTGGCTCACTTCTAGGAATTTGC
TTAGTAATTCAAATTGCTACAGGACTTTTCCTAGCTATACATTATACAGCAGACACAACA
ACAGCATTTTCATCAGTATCCCATATCTGCCGAGACGTAAATTACGGATGACTAATCCGT
TATATACACGCAAACGGAGCCTCACTATTCTTCATCTGCCTATTTATCCATATCGGACGA
GGCATTTACTACGGATCCTACATCTTCCAAGAAACATGAAACATCGGTGTAATCCTCCTA
TTTGCCGTAATAGCTACCGCATTCATAGGGTACGTCCTACCATGAGGACAAATATCCTTC
TGAGGAGCCACAGTGATTACAAATCTCCTCTCAGCAATCCCATATATTGGTCCAACAATT
GTAGAATGAATCTGAGGGGGATTCTCAGTAGACAAAGCCACCCTAACACGATTTTTCGCA
TTCCATTTTATCCTCCCTTTTATTATTACAGCCCTTGTCCTAGTCCACCTCCTATTTCTC
CACGAAACCGGATCCAATAACCCCCTAGGACTAAACTCTAATGCAGACAAAATCCCCTTT
CACCCCTATTACACAGTAAAAGATTTTCTCGGAGTAATTTTATTACTTCTATTCTTCATA
ATCCTAGTTCTCTTCTTTCCTGACCTACTTGGAGACCCAGACAATTACACACCTGCTAAC
CCACTCAACACACCTCCCCATATTAAACCCGAATGATATTTCTTATTTGCCTACGCTATT
CTCCGTTCCATCCCCAATAAACTAGGTGGAGTACTAGCCTTAGTATTATCAATCCTCATT
CTAATTTTCCTACCACTAATCCATACATCAAAACAACGAAGCCTAATATTCCGACCAATT
TCACAAATACTTTACTGAATTTTAATCGCTAACCTGCTTATCCTTACATGAATCGGAGGC
CAACCAGTAGAACACCCATTTATCATTATTGGCCAACTAGCCTCAATTAGCTATTTTTCT

ATCATCCTAATCTTTCTACCATTCTCAGGTNNTATCCAAGG-------------------

>LG103

ATGACAAACATCCGAAAAATTCACCCCCTACTAAAAATAGTAAACCACTCATTCATCGAC

CTCCCCACTCCTCCAAACATCTCATCCTGATGAAACTTTGGCTCACTTCTAGGAATTTGC

TTAGTAATTCAAATCGCTACAGGACTTTTCCTAGCCATACATTATACAGCAGACACAACA

ACAGCATTTTCATCAGTATCCCATATCTGCCGAGACGTAAATTACGGATGACTAATTCGT

TATATACACGCAAACGGAGCCTCACTATTCTTCATCTGCCTATTTATCCATATCGGACGA

GGCATTTACTACGGATCCTACATCTTCCAAGAAACATGAAACATCGGTGTAATCCTCCTA

TTTGCCGTAATAGCTACCGCATTCATAGGGTACGTCCTACCATGAGGACAAATATCCTTC

TGAGGAGCCACAGTGATTACAAATCTCCTCTCAGCAATCCCATATATTGGTCCAACAATT

GTAGAATGAATCTGAGGGGGATTCTCAGTAGACAAAGCCACCCTAACACGATTTTTCGCA

TTCCATTTTATCCTCCCTTTTATTATTACAGCCCTCGTCCTAGTCCACCTCCTATTTCTC

CACGAAACCGGATCCAATAACCCCCTAGGACTAAACTCTAATGCAGACAAAATCCCCTTT

CACCCCTATTACACAGTAAAAGATTTTCTCGGAGTAATTTTACTACTTCTATTTTTCATA

ATCCTAGTTCTCTTCTTTCCTGACTTACTTGGAGACCCAGACAATTACACACCTGCTAAC

CCACTCAACACACCTCCCCATATTAAACCCGAATGATATTTCTTATTTGCCTACGCTATT

CTCCGTTCCATCCCCAATAAACTAGGTGGAGTACTAGCCTTAGTATTATCAATCCTCATT

CTAATTTTTCTACCACTAATCCACACATCAAAACAACGAAGCCTAATATTCCGACCAATT

TCACAAATACTTTACTGAATTTTAATCGCTAACCTGCTTATCCTTACATGAATCGGAGGC

CAACCAGTAGAACACCCGTTTATCATTATTGGCCAACTAGCCTCAATTAGCTATTTTTCT

ATCATCCTAATCTTTCTACCAATCGCAGGAATCATCGAAGACAAAATATTAAAATGATAT

>LG104

ATGACAAACATCCGAAAAATTCACCCCCTACTAAAAATAGTAAACCACTCATTCATCGAC

CTCCCCACTCCTCCAAACATCTCATCCTGATGAAACTTTGGCTCACTTCTAGGAATTTGC

TTAGTAATTCAAATCGCTACAGGACTTTTCCTAGCCATACATTATACAGCAGACACAACA

ACAGCATTTTCATCAGTATCCCATATCTGCCGAGACGTAAATTACGGATGACTAATTCGT

TATATACACGCAAACGGAGCCTCACTATTCTTCATCTGCCTATTTATCCATATCGGACGA

GGCATTTACTACGGATCCTACATCTTCCAAGAAACATGAAACATCGGTGTAATCCTCCTA

TTTGCCGTAATAGCTACCGCATTCATAGGGTACGTCCTACCATGAGGACAAATATCCTTC

TGAGGAGCCACAGTGATTACAAATCTCCTCTCAGCAATCCCATATATTGGTCCAACAATT

GTAGAATGAATCTGAGGGGGATTCTCAGTAGACAAAGCCACCCTAACACGATTTTTCGCA

TTCCATTTTATCCTCCCTTTTATTATTACAGCCCTCGTCCTAGTCCACCTCCTATTTCTC

CACGAAACCGGATCCAATAACCCCCTAGGACTAAACTCTAATGCAGACAAAATCCCCTTT

CACCCCTATTACACAGTAAAAGATTTTCTCGGAGTAATTTTACTACTCCTATTTTTCATA

ATCCTAGTTCTCTTCTTTCCTGACTTACTTGGAGACCCAGACAATTACACACCTGCTAAC

CCACTCAACACACCTCCCCATATTAAACCCGAATGATATTTCTTATTTGCCTACGCTATT

CTCCGTTCCATCCCCAATAAACTAGGTGGAGTACTAGCCTTAGTATTATCAATCCTCATT

CTAATTTTTCTACCACTAATCCACACATCAAAACAACGAAGCCTAATATTCCGACCAATT

TCACAAATACTTTACTGAATTTTAATCGCTAACCTGCTTATCCTTACATGAATCGGAGGC

CAACCAGTAGAACACCCGTTTATCATTATTGGCCAACTAGCCTCAATTAGCTATTTTTCT

ATCATCCTAATCTTTCTACCAATCGCAGGAATCATCGAAGACAAAATATTAAAATGATAT

>LG107

ATGACAAACATCCGAAAAATTCACCCCCTACTAAAAATAGTAAACCACTCATTCATCGAC

CTCCCCACTCCTCCAAACATCTCATCCTGATGAAACTTTGGCTCACTTCTAGGAATTTGC

TTAGTAATTCAAATCGCTACAGGACTTTTCCTAGCCATACATTATACAGCAGACACAACA

ACAGCATTTTCATCAGTATCCCATATCTGCCGAGACGTAAATTACGGATGACTAATTCGT

TATATACACGCAAACGGAGCCTCACTATTCTTCATCTGCCTATTTATCCATATCGGACGA

GGCATTTACTACGGATCCTACATCTTCCAAGAAACATGAAACATCGGTGTAATCCTCCTA

TTTGCCGTAATAGCTACCGCATTCATAGGGTACGTCCTACCATGAGGACAAATATCCTTC

TGAGGAGCCACAGTGATTACAAATCTCCTCTCAGCAATCCCATATATTGGTCCAACAATT

GTAGAATGAATCTGAGGGGGATTCTCAGTAGACAAAGCCACCCTAACACGATTTTTCGCA

TTCCATTTTATCCTCCCTTTTATTATTACAGCCCTCGTCCTAGTCCACCTCCTATTTCTC
CACGAAACCGGATCCAATAACCCCCTAGGACTAAACTCTAATGCAGACAAAATCCCCTTT
CACCCCTATTACACAGTAAAAGATTTTCTCGGAGTAATTTTACTACTCCTATTTTTCATA
ATCCTAGTTCTCTTCTTTCCTGACTTACTTGGAGACCCAGACAATTACACACCTGCTAAC
CCACTCAACACACCTCCCCATATTAAACCCGAATGATATTTCTTATTTGCCTACGCTATT
CTCCGTTCCATCCCCAATAAACTAGGTGGAGTACTAGCCTTAGTATTATCAATCCTCATT
CTAATTTTTCTACCACTAATCCACACATCAAAACAACGAAGCCTAATATTCCGACCAATT
TCACAAATACTTTACTGAATTTTAATCGCTAACCTGCTTATCCTTACATGAATCGGAGGC
CAACCAGTAGAACACCCGTTTATCATTATTGGCCAACTAGCCTCAATTAGCTATTTTTCT
ATCATCCTAATCTTTCTACCAATCGCAGGAATCATCGAAGACAAAATATTAAAATGATAT
>LG106
ATGACAAACATCCGAAAAATTCACCCCCTACTAAAAATAGTAAACCACTCATTCATCGAC
CTCCCCACTCCTCCAAACATCTCATCCTGATGAAACTTTGGCTCACTTCTAGGAATTTGC
TTAATAATTCAAATCGCTACAGGACTTTTCCTAGCCATACATTATACAGCAGACACAACA
ACAGCATTTTCATCAGTATCCCATATCTGCCGAGACGTAAATTACGGATGACTAATTCGT
TATATACACGCAAACGGAGCCTCACTATTCTTCATCTGCCTATTTATCCATATCGGACGA
GGTATTTACTACGGATCCTACATCTTCCAAGAAACATGAAACATCGGTGTAATCCTCCTA
TTTGCCGTAATAGCTACCGCATTCATAGGGTACGTCCTACCATGAGGACAAATATCCTTC
TGAGGAGCCACAGTGATTACAAATCTCCTCTCAGCAATCCCATATATTGGTCCAACAATT
GTAGAATGAATCTGAGGGGGATTCTCAGTAGACAAAGCCACCCTAACACGATTTTTCGCA
TTCCATTTTATCCTCCCTTTTATTATTACAGCCCTCGTCCTAGTCCACCTCCTATTTCTC
CACGAAACCGGATCCAATAACCCCCTAGGACTAAACTCTAATGCAGACAAAATCCCCTTT
CACCCCTATTACACAGTAAAAGATTTTCTCGGAGTAATTTTACTACTCCTATTTTTCATA
ATCCTAGTTCTCTTCTTTCCTGACTTACTTGGAGACCCAGACAATTACACACCTGCTAAC
CCACTCAACACACCTCCCCATATTAAACCCGAATGATATTTCTTATTTGCCTACGCTATT
CTCCGTTCCATCCCCAATAAACTAGGTGGAGTACTAGCCTTAGTATTATCAATCCTCATT
CTAATTTTTCTACCACTAATCCACACATCAAAACAACGAAGCCTAATATTCCGACCAATT
TCACAAATACTTTACTGAATTTTAATCGCTAACCTGCTTATCCTTACATGAATCGGAGGC
CAACCAGTAGAACACCCATTTATCATTATTGGCCAACTAGCCTCAATTAGCTATTTTTCT
ATCATCCTAATCTTTCTACCAATCGCAGGAATCATCGAAGACAAAATATTAAAATGATAT
>LG108
ATGACAAACATCCGAAAAATTCACCCCCTACTAAAAATAGTAAACCACTCATTCATCGAC
CTCCCCACTCCTCCAAACATCTCATCCTGATGAAACTTTGGCTCACTTCTAGGAATTTGC
TTAGTAATTCAAATCGCTACAGGACTTTTCCTAGCCATACATTATACAGCAGACACAACA
ACAGCATTTTCATCAGTATCCCATATCTGCCGAGACGTAAATTACGGATGACTAATTCGT
TATATACACGCAAACGGAGCCTCACTATTCTTCATCTGCCTATTTATCCATATCGGACGA
GGCATTTACTACGGATCCTACATCTTCCAAGAAACATGAAACATCGGTGTAATCCTCCTA
TTTGCCGTAATAGCTACCGCATTCATAGGGTACGTCCTACCATGAGGACAAATATCCTTC
TGAGGAGCCACAGTGATTACAAATCTCCTCTCAGCAATCCCATATATTGGTCCAACAATT
GTAGAATGAATCTGAGGGGGATTCTCAGTAGACAAAGCCACCCTAACACGATTTTTCGCA
TTCCATTTTATCCTCCCTTTTATTATTACAGCCCTCGTCCTAGTCCACCTCCTATTTCTC
CACGAAACCGGATCCAATAACCCCCTAGGACTAAACTCTAATGCAGACAAAATCCCCTTT
CACCCCTATTACACAGTAAAAGATTTTCTCGGAGTAATTTTACTACTTCTATTTTTCATA
ATCCTAGTTCTCTTCTTTCCTGACTTACTTGGAGACCCAGACAATTACACACCTGCTAAC
CCACTCAACACACCTCCCCATATTAAACCCGAATGATATTTCTTATTTGCCTACGCTATT
CTCCGTTCCATCCCCAATAAACTAGGTGGAGTACTAGCCTTAGTATTATCAATCCTCATT
CTAATTTTTCTACCACTAATCCACACATCAAAACAACGAAGCCTAATATTCCGACCAATT
TCACAAATACTTTACTGAATTTTAATCGCTAACCTGCTTATCCTTACATGAATCGGAGGC
CAACCAGTAGAACACCCGTTTATCATTATTGGCCAACTAGCCTCAATTAGCTATTTTTCT
ATCATCCTAATCTTTCTACCAATCGCAGGAATCATCGAAGACAAAATATTAAAATGATAT
>LG109

ATGACAAACATCCGAAAAATTCACCCCCTACTAAAAATAGTAAACCACTCATTCATCGAC

CTCCCCACTCCTCCAAACATCTCATCCTGATGAAACTTTGGCTCACTTCTAGGAATTTGC

TTAGTAATTCAAATCGCTACAGGACTTTTCCTAGCCATACATTATACAGCAGACACAACA

ACAGCATTTTCATCAGTATCCCATATCTGCCGAGACGTAAATTACGGATGACTAATTCGT

TATATACACGCAAACGGAGCCTCACTATTCTTCATCTGCCTATTTATCCATATCGGACGA

GGCATTTACTACGGATCCTACATCTTCCAAGAAACATGAAACATCGGTGTAATCCTCCTA

TTTGCCGTAATAGCTACCGCATTCATAGGGTACGTCCTACCATGAGGACAAATATCCTTC

TGAGGAGCCACAGTGATTACAAATCTCCTCTCAGCAATCCCATATATTGGTCCAACAATT

GTAGAATGAATCTGAGGGGGATTCTCAGTAGACAAAGCCACCCTAACACGATTTTTCGCA

TTCCATTTTATCCTCCCTTTTATTATTACAGCCCTCGTCCTAGTCCACCTCCTATTTCTC

CACGAAACCGGATCCAATAACCCCCTAGGACTAAACTCTAATGCAGACAAAATCCCCTTT

CACCCCTATTACACAGTAAAAGATTTTCTCGGAGTAATTTTACTACTCCTATTTTTCATA

ATCCTAGTTCTCTTCTTTCCTGACTTACTTGGAGACCCAGACAATTACACACCTGCTAAC

CCACTCAACACACCTCCCCATATTAAACCCGAATGATATTTCTTATTTGCCTACGCTATT

CTCCGTTCCATCCCCAATAAACTAGGTGGAGTACTAGCCTTAGTATTATCAATCCTCATT

CTAATTTTTCTACCACTAATCCACACATCAAAACAACGAAGCCTAATATTCCGACCAATT

TCACAAATACTTTACTGAATTTTAATCGCTAACCTGCTTATCCTTACATGAATCGGAGGC

CAACCAGTAGAACACCCGTTTATCATTATTGGCCAACTAGCCTCAATCAGCTATTTTTCT

ATCATCCTAATCTTTCTACCAATCGCAGGAATCATCGAAGACAAAATATTAAAATGATAT

>LG129

ATGACAAACATCCGAAAAATTCACCCCCTACTAAAAATAGTAAACCACTCATTCATCGAC

CTCCCCACTCCTCCAAACATCTCATCCTGATGAAACTTTGGCTCACTTCTAGGAATTTGC

TTAGTAATTCAAATTGCTACAGGACTTTTCCTAGCCATACATTATACAGCAGACACAACA

ACAGCATTTTCATCAGTATCCCATATCTGCCGAGACGTAAATTACGGATGACTAATCCGT

TATATACACGCAAACGGAGCCTCACTATTCTTCATGTGCCTATTTATCCATATCGGACGA

GGCATTTACTACGGATCCTACATCTTCCAAGAAACATGAAACATCGGTGTAATCCTCCTA

TTTGCCGTAATAGCTACCGCATTCATAGGGTACGTCCTACCATGAGGACAAATATCCTTC

TGAGGAGCCACAGTGATTACAAATCTCCTCTCAGCAATCCCATATATTGGTCCAACAATT

GTAGAATGAATCTGAGGGGGATTCTCAGTAGACAAAGCCACCCTAACACGATTTTTCGCA

TTCCATTTTATCCTCCCTTTTATTATTACAGCCCTCATCCTAGTCCACCTCCTATTTCTC

CACGAAACCGGATCCAATAACCCCCTAGGACTAAACTCTAATGCAGACAAACTCCCCTTC

CACCCCTATTACACAGTAAAAGATTTTCTCGGAGTAATTTTACTACTTCTATTTTTCATA

ATCCTAGTTCTCTTCTTTCCTGACTTACTTGGAGACCCAGACAATTACACACCTGCTAAC

CCACTCAACACACCTCCCCATATTAAACCCGAATGATATTTCTTATTTGCCTACGCTATT

CTCCGTTCCATCCCCAATAAACTAGGTGGAGTACTAGCCTTAGTATTATCAATCCTCATT

CTAATTTTTTTACCACTAATTCACACATCAAAACAACGAAGCCTAATATTCCGACCAATT

TCACAAATACTTTACTGAATTTTAATCGCTAACCTGCTTATCCTTACATGAATCGGAGGC

CAACCAGTAGAACACCCGTTTATCATCATTGGCCAACTAGCCTCAATTAGCTATTTTTCT

ATCATCCTAATCTTTCTACCAATCGCAGGAATCATCGAAGACAAAATATTAAAATGATAT

>LG131

ATGACAAACATCCGAAAAATTCACCCCCTACTAAAAATAGTAAACCACTCATTCATCGAC

CTCCCCACTCCTCCAAACATCTCATCCTGATGAAACTTTGGCTCACTTCTAGGAATTTGC

TTAGTAATTCAAATCGCTACAGGACTTTTCCTAGCCATACATTATACAGCAGACACAACA

ACAGCATTTTCATCAGTATCCCATATCTGCCGAGACGTAAATTACGGATGACTAATCCGT

TATATACACGCAAACGGAGCCTCACTATTCTTCATCTGCCTATTTATCCATATCGGACGA

GGCATTTACTACGGATCCTACATCTTCCAAGAAACATGAAACATCGGTGTAATCCTCCTA

TTTGCCGTAATAGCTACCGCATTCATAGGGTACGTCCTACCATGAGGACAAATATCCTTC

TGAGGAGCCACAGTGATTACAAATCTCCTCTCAGCAATCCCATATATTGGTCCAACAATT

GTAGAATGAATCTGAGGGGGATTCTCAGTAGACAAAGCCACCCTAACACGATTTTTCGCA

TTCCATTTTATCCTCCCTTTTATTATTACAGCCCTCATCCTAGTCCACCTCCTATTTCTC

CACGAAACCGGATCCAATAACCCCCTAGGACTAAACTCTAATGCAGACAAAATCCCCTTC

CACCCCTATTACACAGTAAAAGATTTTCTCGGAGTAATTTTACTACTTCTATTTTTCATA
ATCCTAGTTCTCTTCTTTCCTGACTTACTTGGAGACCCAGACAATTACACACCTGCTAAC
CCACTCAACACACCTCCCCATATTAAACCCGAATGATATTTCTTATTTGCCTACGCTATT
CTCCGTTCCATCCCCAATAAACTAGGTGGAGTACTAGCCTTAGTATTATCAATCCTCATT
CTAATTTTTTTACCACTAATTCACACATCAAAACAACGAAGCCTAATATTCCGACCAATT
TCACAAATACTTTACTGAATTTTAATCGCTAACCTGCTTATCCTTACATGAATCGGAGGC
CAACCAGTAGAACACCCGTTTATCATTATTGGCCAACTAGCCTCAATTAGCTATTTTTCT
ATCATCCTAATCTTTCTACCAATCGCAGGAATCATCGAAGACAAAATATTAAAATGATAT
>LG132
ATGACAAACATCCGAAAAATTCACCCCCTACTAAAAATAGTAAACCACTCATTCATCGAC
CTCCCCACTCCTCCAAACATCTCATCCTGATGAAACTTTGGCTCACTTCTAGGAATTTGC
TTAGTAATTCAAATCGCTACAGGACTTTTCCTAGCCATACATTATACAGCAGACACAACA
ACAGCATTTTCATCAGTATCCCATATCTGCCGAGACGTAAATTACGGATGACTAATCCGT
TATATACACGCAAACGGAGCCTCACTATTCTTCATCTGCCTATTTATCCATATCGGACGA
GGCATTTACTACGGATCCTACATCTTCCAAGAAACATGAAACATCGGTGTAATCCTCCTA
TTTGCCGTAATAGCTACCGCATTCATAGGGTACGTCCTACCATGAGGACAAATATCCTTC
TGAGGAGCCACAGTGATTACAAATCTCCTCTCAGCAATCCCATATATTGGTCCAACAATT
GTAGAATGAATCTGAGGGGGATTCTCAGTAGACAAAGCCACCCTAACACGATTTTTCGCA
TTCCATTTTATCCTCCCTTTTATTATTACAGCCCTCATCCTAGTCCACCTCCTATTTCTC
CACGAAACCGGATCCAATAACCCCCTAGGACTAAACTCTAATGCAGACAAAATCCCCTTT
CACCCCTATTACACAGTAAAAGATTTTCTCGGAGTAATTTTACTACTTCTATTTTTCATA
ATCCTAGTTCTCTTCTTTCCTGACTTACTTGGAGACCCAGACAATTACACACCTGCTAAC
CCACTCAACACACCTCCCCATATTAAACCCGAATGATATTTCTTATTTGCCTACGCTATT
CTCCGTTCCATCCCCAATAAACTAGGTGGAGTACTAGCCTTAGTATTATCAATCCTCATT
CTAATTTTTTTACCACTAATTCACACATCAAAACAACGAAGCCTAATATTCCGACCAATT
TCACAAATACTTTACTGAATTTTAATCGCTAACCTGCTTATCCTTACATGAATCGGAGGC
CAACCAGTAGAACACCCGTTTATCATTATTGGCCAACTAGCCTCAATTAGCTATTTTTCT
ATCATCCTAATCTTTCTACCAATCGCAGGAATCATCGAAGACAAAATATTAAAATGATAT
>LG139
ATGACAAACATCCGAAAAATTCACCCCCTACTAAAAATAGTAAACCACTCATTCATCGAC
CTCCCCACTCCTCCAAACATCTCATCCTGATGAAACTTTGGCTCACTTCTAGGAATTTGC
TTAGTAATTCAAATCGCTACAGGACTTTTCCTAGCCATACATTATACAGCAGACACAACA
ACAGCATTTTCATCAGTATCCCATATCTGCCGAGACGTAAATTACGGATGACTAATCCGT
TATATACACGCAAACGGAGCCTCACTATTCTTCATCTGCCTATTTATCCATATCGGACGA
GGCATTTACTACGGATCCTACATCTTCCAAGAAACATGAAACATCGGTGTAATCCTCCTA
TTTGCCGTAATAGCTACCGCATTCATAGGGTACGTCCTACCATGAGGACAAATATCCTTC
TGAGGAGCCACAGTGATTACAAATCTCCTCTCAGCAATCCCATATATTGGTCCAACAATT
GTAGAATGAATCTGAGGGGGATTCTCAGTAGACAAAGCCACCCTAACACGATTTTTCGCA
TTCCATTTTATCCTCCCTTTTATTATTACAGCCCTCATCCTAGTCCACCTCCTATTTCTC
CACGAAACCGGATCCAATAACCCCCTAGGACTAAACTCTAATGCAGACAAAATCCCCTTT
CACCCCTATTACACAGTAAAAGATTTTCTCGGAGTAATTTTACTACTTCTATTTTTCATA
ATCCTAGTTCTCTTCTTTCCTGACTTACTTGGAGACCCAGACAATTACACACCTGCTAAC
CCACTCAACACACCTCCCCATATTAAACCCGAATGATATTTCTTATTTGCCTACGCTATT
CTCCGTTCCATCCCCAATAAACTAGGTGGAGTACTAGCCTTAGTATTATCAATCCTCATT
CTAATTTTTCTACCACTAATTCACACATCAAAACAACGAAGCCTAATATTCCGACCAATT
TCACAAATACTTTACTGAATTTTAATCGCTAACCTGCTTATCCTTACATGAATCGGAGGC
CAACCAGTAGAACACCCGTTTATCATTATTGGCCAACTAGCCTCAATTAGCTATTTTTCT
ATCATCCTAATCTTTCTACCAATCGCAGGAATCATCGAAGACAAAATATTAAAATGATAT
>LG140
ATGACAAACATCCGAAAAATTCACCCCCTACTAAAAATAGTAAACCACTCATTCATCGAC
CTCCCCACTCCTCCAAACATCTCATCCTGATGAAACTTTGGCTCACTTCTAGGAATTTGC

TTAGTAATTCAAATCGCTACAGGACTTTTCCTAGCCATACATTATACAGCAGACACAACA
ACAGCATTTTCATCAGTATCCCATATCTGCCGAGACGTAAATTACGGATGACTAATCCGT
TATATACACGCAAACGGAGCCTCACTATTCTTCATCTGCCTATTTATCCATATCGGACGA
GGCATTTACTACGGATCCTACATCTTCCAAGAAACATGAAACATCGGTGTAATCCTCCTA
TTTGCCGTAATAGCTACCGCATTCATAGGGTACGTCCTACCATGAGGACAAATATCCTTC
TGAGGAGCCACAGTGATTACAAATCTCCTCTCAGCAATCCCATATATTGGTCCAACAATT
GTAGAATGAATCTGAGGGGGATTCTCAGTAGACAAAGCCACCCTAACACGATTTTTCGCA
TTCCATTTTATCCTCCCTTTTATTATTACAGCCCTCATCCTAGTCCACCTCCTATTTCTC
CACGAAACCGGATCCAATAACCCCCTAGGACTAAACTCTAATGCAGACAAAATCCCCTTT
CACCCCTATTACACAGTAAAAGATTTTCTCGGAGTAATTTTACTACTTCTATTTTTCATA
ATCCTAGTTCTCTTCTTCCCTGACTTACTTGGAGACCCAGACAATTACACACCTGCTAAC
CCACTCAACACACCTCCCCATATTAAACCCGAATGATATTTCTTATTTGCCTACGCTATT
CTCCGTTCCATCCCCAATAAACTAGGTGGAGTACTAGCCTTAGTATTATCAATCCTCATT
CTAATTTTTCTACCACTAATTCACACATCAAAACAACGAAGCCTAATATTCCGACCAATT
TCACAAATACTTTACTGAATTTTAATCGCTAACCTGCTTATCCTTACATGAATCGGAGGC
CAACCAGTAGAACACCCGTTTATCATTATTGGCCAATTAGCCTCAATTAGCTATTTTTCT
ATCATCCTAATCTTTCTACCAATCGCAGGAATCATCGAAGACAAAATATTAAAATGATAT
>LG110
ATGACAAACATCCGAAAAATTCACCCCCTACTAAAAATAGTAAACCACTCATTCATCGAC
CTCCCCACTCCTCCAAACATCTCATCCTGATGAAACTTTGGCTCACTTCTAGGAATTTGC
TTAGTAATTCAAATCGCTACAGGACTTTTCCTAGCCATACATTATACAGCAGACACAACA
ACAGCATTTTCATCAGTATCCCATATCTGCCGAGACGTAAATTACGGATGACTAATACGT
TATATACACGCAAACGGAGCCTCACTATTCTTCATCTGCCTATTTATCCATATCGGACGA
GGCATTTACTACGGATCCTACATCTTCCAAGAAACATGAAACATCGGTGTAATCCTCCTA
TTTGCCGTAATAGCTACCGCATTCATAGGGTACGTCCTACCATGAGGACAAATATCCTTC
TGAGGAGCCACAGTGATTACAAATCTCCTCTCAGCAATCCCATATATTGGTCCAACAATT
GTAGAATGAATCTGAGGGGGATTCTCAGTAGACAAAGCCACCCTAACACGATTTTTCGCA
TTCCATTTTATCCTCCCTTTTATTATTACAGCCCTCGTCCTAGTCCACCTCCTATTTCTC
CACGAAACCGGATCCAATAACCCCCTAGGACTAAACTCTAATGCAGACAAAATCCCCTTT
CACCCCTATTACACAGTAAAAGATTTTCTCGGAGTAATTTTACTACTTCTATTTTTCATA
ATCCTAGTTCTCTTCTTTCCTGACTTACTTGGAGACCCAGACAATTACACACCTGCTAAC
CCACTCAACACACCTCCCCATATTAAACCCGAATGATATTTCTTATTTGCCTACGCTATT
CTCCGTTCCATCCCCAATAAACTAGGTGGAGTACTAGCCTTAGTATTATCAATCCTCATT
CTAATTTTTCTACCACTAATCCACACATCAAAACAACGAAGCCTAATATTCCGACCAATT
TCACAAATACTTTACTGAATTTTAATCGCTAACCTGCTTATCCTTACATGAATCGGAGGC
CAACCAGTAGAACACCCGTTTATCATTATTGGCCAACTAGCCTCAATTAGCTATTTTTCT
ATCATCCTAATCTTTCTACCAATCGCAGGAATCATCGAAGACAAAATATTAAAATGATAT
>LG133
ATGACAAACATCCGAAAAATTCACCCCCTACTAAAAATAGTAAACCACTCATTCATCGAC
CTCCCCACTCCTCCAAACATCTCATCCTGATGAAACTTTGGCTCACTTCTAGGAATTTGC
TTAGTAATTCAAATCGCTACAGGACTTTTCCTAGCCATACATTATACAGCAGACACAACA
ACAGCATTTTCATCAGTATCCCATATCTGCCGAGACGTAAATTACGGATGACTAATCCGT
TATATACACGCAAACGGAGCCTCACTATTCTTCATCTGCCTATTTATCCATATCGGACGA
GGCATTTACTACGGATCCTACATCTTCCAAGAAACATGAAACATCGGTGTAATCCTCCTA
TTTGCCGTAATAGCTACCGCATTCATAGGGTACGTCCTACCATGAGGACAAATATCCTTC
TGAGGAGCCACAGTGATTACAAATCTCCTCTCAGCAATCCCATATATTGGTCCAACAATT
GTAGAATGAATCTGAGGGGGATTCTCAGTAGACAAAGCCACCCTAACACGATTTTTCGCA
TTCCATTTTATCCTCCCTTTTATTATTACAGCCCTCATCCTAGTCCACCTCCTATTTCTC
CACGAAACCGGATCCAATAACCCCCTAGGACTAAACTCTAATGCAGACAAAATCCCCTTT
CACCCCTATTACACAGTAAAAGATTTTCTCGGAGTAATTTTACTACTTCTATTTTTCATA
ATCCTAGTTCTCTTCTTTCCTGACTTACTTGGAGACCCAGACAATTACACACCTGCTAAC

CCACTCAACACACCTCCCCATATTAAACCCGAATGATATTTCTTATTTGCCTACGCTATT
CTCCGTTCCATCCCCAATAAACTAGGTGGAGTACTAGCCTTAGTATTATCAATCCTCATT
CTAATTTTTTTACCACTAATTCACACATCAAAACAACGAAGCCTAATATTCCGACCAATT
TCACAAATACTTTACTGAATTTTAATCGCTAACCTGCTTATCCTTACATGAATCGGAGGC
CAACCAGTAGAACACCCGTTTATCATTATTGGCCAACTAGCCTCAATTAGCTATTTTTCT
ATCATCCTAATCTTTCTTCCAATCGCAGGAATCATCGAAGACAAAATATTAAAATGATAT

>LG134

ATGACAAACATCCGAAAAATTCACCCCCTACTAAAAATAGTAAACCACTCATTCATCGAC
CTCCCCACTCCTCCAAACATCTCATCCTGATGAAACTTTGGCTCACTTCTAGGAATTTGC
TTAGTAATTCAAATCGCTACAGGACTTTTCCTAGCCATACATTATACAGCAGACACAACA
ACAGCATTTTCATCAGTATCCCATATCTGCCGAGACGTAAATTACGGATGACTAATCCGT
TATATACACGCAAACGGAGCCTCACTATTCTTCATCTGCCTATTTATCCATATCGGACGA
GGCATTTACTACGGATCCTACATCTTCCAAGAAACATGAAACATCGGTGTAATCCTCCTA
TTTGCCGTAATAGCTACCGCATTCATAGGGTACGTCCTACCATGAGGACAAATATCCTTC
TGAGGAGCCACAGTGATTACAAATCTCCTCTCAGCAATCCCATATATTGGTCCAACAATT
GTAGAATGAATCTGAGGGGGATTCTCAGTAGACAAAGCCACCCTAACACGATTTTTCGCA
TTCCATTTTATCCTCCCTTTTATTATTACAGCCCTCATCCTAGTCCACCTCCTATTTCTC
CACGAAACCGGATCCAATAACCCCCTAGGACTAAACTCTAATGCAGACAAAATCCCCTTT
CACCCCTATTACACAGTAAAAGATTTTCTCGGAGTAATTTTACTACTTCTATTTTTCATA
ATCCTAGTTCTCTTCTTTCCTGACTTACTTGGAGACCCAGACAATTACACACCTGCTAAC
CCACTCAACACACCTCCCCATATTAAACCCGAATGATATTTCTTATTTGCCTACGCTATT
CTCCGTTCCATCCCCAATAAACTAGGTGGAGTACTAGCCTTAGTATTATCAATCCTCATT
CTAATTTTTCTACCACTAATCCACACATCAAAACAACGAAGCCTAATATTCCGACCAATT
TCACAAATACTTTACTGAATTTTAATCGCTAACCTGCTTATCCTTACATGAATCGGAGGC
CAACCAGTAGAACACCCGTTTATCATTATTGGCCAACTAGCCTCAATTAGCTATTTTTCT
ATTATCCTAATCCTTTTACCAATCGCAGGAATCATTGAAGATAATATATTAAAATGATAT

>LG114

ATGACAAACATCCGAAAAATTCACCCCCTACTAAAAATAGTAAACCACTCATTCATCGAC
CTCCCCACTCCTCCAAACATCTCATCCTGATGAAACTTTGGCTCACTTCTAGGAATTTGC
TTAGTAATTCAAATCGCTACAGGACTTTTCCTAGCCATACATTATACAGCAGACACAACA
ACAGCATTTTCATCAGTATCCCATATCTGCCGAGACGTAAATTACGGATGACTAATCCGT
TATATACACGCAAACGGAGCCTCACTATTCTTCATCTGCCTATTTATCCATATCGGACGA
GGCATTTACTACGGATCCTACATCTTCCAAGAAACATGAAACATCGGTGTAATCCTCCTA
TTTGCCGTAATAGCTACCGCATTCATAGGGTACGTCCTACCATGAGGACAAATATCCTTC
TGAGGAGCCACAGTGATTACAAATCTCCTCTCAGCAATCCCATATATTGGTCCAACAATT
GTAGAATGAATCTGAGGGGGATTCTCAGTAGACAAAGCCACCCTAACACGATTTTTCGCA
TTCCATTTTATCCTCCCTTTTATTATTACAGCCCTCATCCTAGTCCACCTCCTATTTCTC
CACGAAACCGGATCCAATAACCCCCTAGGACTAAACTCTAATGCAGACAAAATCCCCTTT
CACCCCTATTACACAGTAAAAGATTTTCTCGGAGTAATTTTACTACTTCTATTTTTCATA
ATCCTAGTTCTCTTCTTCCCTGACTTACTTGGAGACCCAGACAATTACACACCTGCTAAC
CCACTCAACACACCTCCCCATATTAAACCCGAATGATATTTCTTATTTGCCTACGCTATT
CTCCGTTCCATCCCCAATAAACTAGGTGGAGTACTAGCCTTAGTATTATCAATCCTCATT
CTAATTTTTCTACCACTAATTCACACATCAAAACAACGAAGCCTAATATTCCGACCAATT
TCACAAATACTTTACTGAATTTTAATCGCTAACCTGCTTATCCTTACATGAATCGGAGGC
CAACCAGTAGAACACCCGTTTATCATTATTGGCCAATTAGCCTCAATTAGCTATTTTTCT
ATCATCCTAATCTTTCTACCAATCGCAGGAATCATCGAAGACAAAATATTAAAATGATAT

>LG117

ATGACAAACATCCGAAAAATTCACCCCCTACTAAAAATAGTAAACCACTCATTCATCGAC
CTCCCCACTCCTCCAAACATCTCATCCTGATGAAACTTTGGCTCACTTCTAGGAATTTGC
TTAGTAATTCAAATCGCTACAGGACTTTTCCTAGCCATACATTATACAGCAGACACAACA
ACAGCATTTTCATCAGTATCCCATATCTGCCGAGACGTAAATTACGGATGACTAATCCGT

TATATACACGCAAACGGAGCCTCACTATTCTTCATCTGCCTATTTATCCATATCGGACGA

GGCATTTACTACGGATCCTACATCTTCCAAGAAACATGAAACATCGGTGTAATCCTCCTA

TTTGCCGTAATAGCTACCGCATTCATAGGGTACGTCCTACCATGAGGACAAATATCCTTC

TGAGGAGCCACAGTGATTACAAATCTCCTCTCAGCAATCCCATATATTGGTCCAACAATT

GTAGAATGAATCTGAGGGGGATTCTCAGTAGACAAAGCCACCCTAACACGATTTTTCGCA

TTCCATTTTATCCTCCCTTTTATTATTACAGCCCTCATCCTAGTCCACCTCCTATTTCTC

CACGAAACCGGATCCAATAACCCCCTAGGACTAAACTCTAATGCAGACAAAATCCCCTTT

CACCCCTATTACACAGTAAAAGATTTTCTCGGAGTAATTTTACTACTTCTATTTTTCATA

ATCCTAGTTCTCTTCTTTCCTGACTTACTTGGAGACCCAGACAATTACACACCTGCTAAC

CCACTCAACACACCTCCCCATATTAAACCCGAATGATATTTCTTATTTGCCTACGCTATT

CTCCGTTCCATCCCCAATAAACTAGGTGGAGTACTAGCCTTAGTATTATCAATCCTCATT

CTAATTTTTCTACCACTAATTCACACATCAAAACAACGAAGCCTAATATTCCGACCAATT

TCACAAATACTTTACTGAATTTTAATCGCTAACCTGCTTATCCTTACATGAATCGGAGGC

CAACCAGTAGAACACCCGTTTATCATTATTGGCCAACTAGCCTCAATTAGCTATTTTTCT

ATCATCCTAATCTTTCTACCAATCGCAGGAATCATCGAAGACAAAATATTAAAATGATAT

>LG138

ATGACAAACATCCGAAAAATTCACCCCCTACTAAAAATAGTAAACCACTCATTCATCGAC

CTCCCCACTCCTCCAAACATCTCATCCTGATGAAACTTTGGCTCACTTCTAGGAATTTGC

TTAGTAATTCAAATCGCTACAGGACTTTTCCTAGCCATACATTATACAGCAGACACAACA

ACAGCATTTTCATCAGTATCCCATATCTGCCGAGACGTAAATTACGGATGACTAATCCGT

TATATACACGCAAACGGAGCCTCACTATTCTTCATCTGCCTATTTATCCATATCGGACGA

GGCATTTACTACGGATCCTACATCTTCCAAGAAACATGAAACATCGGTGTAATCCTCCTA

TTTGCCGTAATAGCTACCGCATTCATAGGGTACGTCCTACCATGAGGACAAATATCCTTC

TGAGGAGCCACAGTGATTACAAATCTCCTCTCAGCAATCCCATATATTGGTCCAACAATT

GTAGAATGAATCTGAGGGGGATTCTCAGTAGACAAAGCCACCCTAACACGATTTTTCGCA

TTCCATTTTATCCTCCCTTTTATTATTACAGCCCTCATCCTAGTCCACCTCCTATTTCTC

CACGAAACCGGATCCAATAACCCCCTAGGACTAAACTCTAATGCAGACAAAATCCCCTTT

CACCCCTATTACACAGTAAAAGATTTTCTCGGAGTAATTTTACTACTTCTATTTTTCATA

ATCCTAGTTCTCTTCTTTCCTGACTTACTTGGAGACCCAGACAATTACACACCTGCTAAC

CCACTCAACACACCTCCCCATATTAAACCCGAATGATATTTCTTATTTGCCTACGCTATT

CTCCGTTCCATCCCCAATAAACTAGGTGGAGTACTAGCCTTAGTATTATCAATCCTCATT

CTAATTTTTCTACCACTAATTCACACATCAAAACAACGAAGCCTAATATTCCGACCAATT

TCACAAATACTTTACTGAATTTTAATCGCTAACCTGCTTATCCTTACATGAATCGGAGGC

CAACCAGTAGAACACCCGTTTATCATTATTGGCCAACTAGCCTCAATTAGCTATTTTTCT

ATCATCCTAATCTTTCTACCAATCGCAGGAATCATCGAAGACAAAATATTAAAATGATAT

>LG115

ATGACAAACATCCGAAAAATTCACCCCCTACTAAAAATAGTAAACCACTCATTCATTGAC

CTCCCCACTCCTCCAAACATCTCATCTTGATGAAACTTTGGCTCACTTCTAGGAATTTGC

TTAGTAATTCAAATTGCTACAGGACTTTTCCTAGCCATACATTATACAGCAGACACAACA

ACAGCATTTTCATCAGTATCCCATATCTGCCGAGACGTAAATTACGGATGACTAATCCGT

TATATACACGCAAGCGGAGCCTCACTATTCTTCATCTGCCTATTTATCCATATCGGACGA

GGCATTTACTACGGATCCTACATCTTCCAAGAAACATGAAACATCGGTGTAATCCTCCTA

TTTGCCGTAATAGCTACCGCATTCATAGGGTACGTCCTACCATGAGGACAAATATCCTTC

TGAGGAGCCACAGTGATTACAAATCTCCTCTCAGCAATCCCATATATTGGTCCAACAATT

GTAGAATGAATCTGAGGGGGATTCTCAGTAGACAAAGCCACCCTAACACGATTTTTCCCA

TTCCATTTTATCCTCCCCTTTATTATTACAGCCCTCATCCTAGTCCACCTCCTATTTCTC

CACGAAACCGGATCCAATAACCCCCTAGGACTAAACTCTAATGCAGACAAAATCCCCTTT

CACCCCTATTACACAGTAAAAGATTTTCTAGGAGTAATTTTAATAATTCTATTTTTCATA

ATCCTAGTTCTCTTCTTCCCTGACTTACTTGGAGACCCAGACAATTACACACCTGCTAAC

CCACTCAACACACCTCCCCACATTAAACCCGAATGATATTTCTTATTTGCCTACGCCATT

CTCCGTTCCATCCCCAATAAACTAGGTGGAGTACTAGCCTTAGTATTATCAATCCTCATT

CTAATTTTTTTACCACTAATTCACACATCAAAACAACGAAGCCTAATATTCCGACCAATT

TCACAAATACTTTACTGAATTTTAATCGCTAACCTGCTTATCCTTACATGAATCGGAGGC

CAACCAGTAGAACACCCGTTTATCATCATTGGCCAACTAGCCTCAATCAGCTATTTTTCT

ATTATCCTAATTCTTTTACCAATCGCAGGAATCATTGAAGATAATATATTAAAATGATAT

>LG112

ATGACAAACATTCGAAAAATTCACCCACTACTAAAAATAGTTAACCACTCATTCATTGAT

CTTCCCACTCCCCCTAATATTTCATCTTGATGAAACTTTGGCTCACTCCTAGGAATTTGC

TTAATAATTCAAATCGCTACAGGACTTTTCCTAGCCATACATTATACAGCAGACACAACA

ACAGCATTCTCATCAGTATCCCATATCTGCCGAGACGTCAATTATGGATGACTAATCCGC

TATATACATGCAAACGGAGCTTCAATATTCTTTATTTGCCTATTCATTCACATCGGACGA

GGAATTTACTACGGATCTTATATCTTTCAAGAAACATGAAACATTGGAGTAATTCTCTTA

TTTGCCGTAATAGCCACCGCATTCATAGGATATGTACTTCCATGAGGACAAATATCCTTC

TGAGGGGCCACAGTCATTACAAATCTTCTTTCAGCTATTCCATATATTGGCCCAACAATC

GTAGAATGAATTTGAGGAGGATTTTCAGTGGACAAAGCCACTTTAACACGATTTTTCGCA

TTTCACTTCATTCTCCCCTTTATTATCACAGCCTTAGTCCTAGTCCATCTCCTATTCCTT

CACGAAACCGGATCTAATAATCCCCTAGGCCTTAACTCCAACTCAGACAAAATCCCTTTT

CACCCATACTACACAGTAAAAGATTTTCTCGGAGTAATTCTACTACTCCTATTTTTCACA

ATTTTAGTCCTCTTCTTCCCTGACTTACTTGGAGATCCAGACAACTACACACCCGCTAAC

CCCCTTAACACTCCCCCCCACATTAAACCCGAATGATATTTCTTATTTGCCTATGCTATC

CTACGTTCAATTCCTAACAAACTAGGAGGAGTCCTAGCCCTAGTACTTTCAATCCTTATC

CTAATTTTTTTACCACTCATTCACACATCAAAACAACGAAGCCTAATATTCCGACCTATT

TCCCAAATACTTTACTGAATCTTAATTGCCAACCTACTTATCCTCACATGAATCGGAGGC

CAACCAGTAGAACACCCATTTATTATCATTGGCCAACTAGCCTCAATCAGTTACTTTTCC

ATTATCCTAATTCTTTTACCAATCGCAGGAATCATTGAAGATAATATATTAAAATGATAT

>LG120 /transl_table=2

ATGACAAACATTCGAAAAATTCACCCACTACTAAAAATAGTTAACCACTCATTCATTGAT

CTTCCCACTCCCCCTAATATTTCATCTTGATGAAACTTTGGCTCACTCCTAGGAATTTGC

TTAATAATTCAAATCGCTACAGGACTTTTCCTAGCCATACATTATACAGCAGACACAACA

ACAGCATTCTCATCAGTATCCCATATCTGCCGAGACGTCAATTATGGATGACTAATCCGC

TATATACATGCAAACGGAGCTTCAATATTCTTTATTTGCCTATTCATTCACATCGGACGA

GGAATTTACTACGGATCTTATATCTTTCAAGAAACATGAAACATTGGAGTAATTCTCTTA

TTTGCCGTAATAGCCACCGCATTCATAGGGTATGTACTTCCATGAGGACAAATATCCTTC

TGAGGGGCCACAGTCATTACAAATCTTCTTTCAGCTATTCCATATATTGGCCCAACAATC

GTAGAATGAATTTGAGGAGGATTTTCAGTGGACAAAGCCACTTTAACACGATTTTTCGCA

TTTCACTTCATTCTCCCCTTTATTATCACAGCCTTAGTCCTAGTCCATCTCCTATTCCTT

CACGAAACCGGATCTAATAATCCCCTAGGCCTTAACTCCAACTCAGACAAAATCCCTTTT

CACCCATACTACACAGTAAAAGATTTTCTAGGAGTAATTCTACTACTCCTATTTTTCACA

ATTTTAGTCCTCTTCTTCCCTGACTTACTTGGAGATCCAGACAACTACACACCCGCTAAC

CCCCTTAACACTCCCCCCCACATTAAACCCGAATGATATTTCTTATTTGCCTATGCTATC

CTACGTTCAATTCCTAACAAACTAGGAGGAGTCCTAGCCCTAGTACTTTCAATCCTTATC

CTAATTTTTTTACCACTCATTCACACATCAAAACAACGAAGCCTAATATTTCGACCTATT

TCCCAAATACTTTACTGAATCTTAATTGCCAACCTACTTATCCTCACATGAATCGGAGGC

CAACCAGTAGAACACCCATTTATTATCATTGGCCAACTAGCCTCAATCAGTTACTTTTCC

ATTATCCTAATTCTTTTACCAATCGCAGGAATCATTGAAGATAATATATTAAAATGATAT

>LG122 /transl_table=2

ATGACAAACATTCGAAAAATTCACCCACTACTAAAAATAGTTAACCACTCATTCATTGAT

CTTCCCACTCCCCCTAATATTTCATCTTGATGAAACTTTGGCTCACTCCTAGGAATTTGC

TTAATAATTCAAATCGCTACAGGACTTTTCCTAGCCATACATTATACAGCAGACACAACA

ACAGCATTCTCATCAGTATCCCATATCTGCCGAGACGTCAATTATGGATGACTAATCCGC

TATATACATGCAAACGGAGCTTCAATATTCTTTATTTGCCTATTCATTCACATCGGACGA

GGAATTTACTACGGATCTTATATCTTTCAAGAAACATGAAACATTGGAGTAATCCTCTTA

TTTGCCGTAATAGCCACCGCATTCATAGGGTATGTACTTCCATGGGGACAAATATCCTTC
TGAGGGGCCACAGTCATTACAAATCTTCTTTCAGCTATTCCATATATTGGCCCAACAATC
GTAGAATGAATTTGAGGAGGATTTTCAGTGGACAAAGCCACTTTAACACGATTTTTCGCA
TTTCACTTCATTCTCCCCTTTATTATCACAGCCTTAGTCCTAGTCCATCTCCTATTCCTT
CACGAAACCGGATCTAATAATCCCCTAGGCCTTAACTCCAACTCAGACAAAATCCCTTTT
CACCCATACTACACAGTAAAAGATTTTCTCGGAGTAATTCTACTACTTCTATTTTTCACA
ATTTTAGTCCTCTTCTTCCCTGACTTACTTGGAGATCCAGACAACTACACACCCGCTAAC
CCCCTTAACACTCCCCCCCACATTAAACCCGAATGGTATTTCTTATTTGCCTATGCTATC
CTACGTTCAATTCCTAACAAACTAGGAGGAGTCCTAGCCCTAGTACTTTCAATCCTTATC
CTAATTTTTTTACCACTCATTCACACATCAAAACAACGAAGCCTAATATTCCGACCTATT
TCCCAAATACTTTACTGAATATTAATTGCCAACCTACTTATCCTCACATGAATCGGAGGC
CAACCAGTAGAACACCCATTTATTATCATTGGCCAACTAGCCTCAATCAGTTACTTTTCC
ATTATCCTAATTCTTTTACCAATCGCAGGAATCATTGAAGATAATATATTAAAATGATAT
>LG124 /transl_table=2
ATGACAAACATTCGAAAAATTCACCCACTACTAAAAATAGTTAACCACTCATTCATCGAT
CTTCCCACTCCCCCTAATATTTCATCTTGATGAAACTTTGGCTCACTCCTAGGAATTTGC
TTAATAATTCAAATCGCTACAGGACTTTTCCTAGCCATACATTATACAGCAGACACAACA
ACAGCATTCTCATCAGTATCCCATATCTGCCGAGACGTCAATTATGGATGACTAATCCGC
TATATACATGCAAACGGAGCTTCAATATTCTTTATTTGCCTATTCATTCACATCGGACGA
GGAATTTACTACGGATCTTATATCTTTCAAGAAACATGAAACATTGGAGTAATTCTCTTA
TTTGCCGTAATAGCCACCGCATTTATAGGGTATGTACTTCCATGAGGACAAATATCCTTC
TGAGGGGCCACAGTCATTACTAATCTTCTTTCAGCTATTCCATATATTGGCCCAACAATC
GTAGAATGAATTTGAGGGGGATTTTCAGTGGACAAAGCCACTTTAACACGATTTTTCGCA
TTTCACTTCATTCTCCCCTTTATTATCACAGCCTTAGTCCTAGTCCATCTCCTATTCCTT
CACGAAACCGGATCTAATAATCCCCTAGGCCTTAACTCCAACTCAGACAAAATCCCCTTT
CACCCATACTACACAGTAAAAGATTTTCTCGGAGTAATTCTACTACTTCTATTTTTCACA
ATTTTAGTCCTCTTCTTCCCTGACTTACTTGGAGATCCAGACAACTACACACCCGCTAAC
CCCCTTAACACTCCCCCCCACATTAAACCCGAATGGTATTTCTTATTTGCCTATGCTATC
CTACGTTCAATTCCTAACAAACTAGGAGGAGTCCTAGCCCTAGTACTTTCAATCCTTATC
CTAATTTTTTTACCACTCATTCACACATCAAAACAACGAAGCCTAATATTCCGACCTATT
TCCCAAATACTTTACTGAATCTTAATTGCCAACCTACTTATCCTCACATGAATCGGAGGC
CAACCAGTAGAACACCCATTTATTATCATTGGCCAACTAGCCTCAATCAGTTACTTTTCC
ATTATCCTAATTCTTTTGCCAATCGCAGGAATCATTGAAGATAATATATTAAAATGATAT
>LG126 /transl_table=2
ATGACAAACATTCGAAAAATTCACCCACTACTAAAAATAGTTAACCACTCATTCATTGAT
CTTCCCACTCCCCCTAATATTTCATCTTGATGAAACTTTGGCTCACTCCTAGGAATTTGC
TTAATAATTCAAATCGCTACAGGACTTTTCCTAGCCATACATTATACAGCAGACACAACA
ACAGCATTCTCATCAGTATCCCATATCTGCCGAGACGTCAATTATGGATGACTAATCCGC
TATATACATGCAAACGGAGCTTCAATATTCTTTATTTGCCTATTCATTCACATCGGACGA
GGAATTTACTACGGATCTTATATCTTTCAAGAAACATGAAACATTGGAGTAATTCTCTTA
TTTGCCGTAATAGCCACCGCATTCATAGGATATGTACTTCCATGAGGACAAATATCCTTC
TGAGGGGCCACAGTCATTACAAATCTTCTTTCAGCTATTCCATATATTGGCCCAACAATC
GTAGAATGAATTTGAGGAGGATTTTCAGTGGACAAAGCCACTTTAACACGATTTTTCGCA
TTTCACTTCATTCTCCCCTTTATTATCACAGCCTTAGTCCTAGTCCATCTCCTATTCCTT
CACGAAACCGGATCTAATAATCCCCTAGGCCTTAACTCCAACTCAGACAAAATCCCTTTT
CACCCATACTACACAGTAAAAGATTTTCTCGGAGTAATTCTACTACTTCTATTTTTCACA
ATTTTAGTCCTCTTCTTCCCTGACTTACTTGGAGATCCAGACAACTACACACCCGCTAAC
CCCCTTAACACTCCCCCACACATTAAACCCGAATGGTATTTCTTATTTGCCTATGCTATC
CTACGTTCAATTCCTAACAAACTAGGAGGAGTCCTAGCCCTAGTACTTTCAATCCTTATC
CTAATTTTTTTACCACTCATTCACACATCAAAACAACGAAGCCTAATATTCCGACCTATT
TCCCAAATACTTTACTGAATCTTAATTGCCAACCTACTTATCCTCACATGAATCGGAGGC

CAACCAGTAGAACACCCATTTATTATCATTGGCCAACTAGCCTCAATCAGTTACTTTTCC
ATTATCCTAATTCTTTTACCAATCGCAGGAATCATTGAAGATAATATATTAAAATGATAT
>M5978
ATGACAAACATCCGAAAAATTCACCCCCTAATAAAAATAGTAAACCACTCATTCATTGAC
CTCCCTACTCCTCCAAACATCTCATCCTGATGAAACTTTGGCTCACTCCTAGGAATTTGC
TTAGTAATTCAAATTACTACAGGACTCTTCCTAGCCATACACTATACAGCAGATACAACA
ACAGCATTTTCATCAGTATCCCACATCTGCCGAGACGTAAATTACGGATGACTAATTCGT
TATATACACGCAAACGGAGCCTCAATATTCTTCATCTGCCTATTTATCCATATCGGACGA
GGAATTTACTACGGATCCTACATCTTTCAAGAAACATGAAACATCGGTGTAATCCTCCTA
TTTGCCGTAATAGCTACCGCATTCATGGGTTACGTCCTACCATGAGGACAAATATCCTTC
TGAGGCGCCACAGTCATTACAAACCTCCTCTCAGCAATCCCATACATTGGCCCAACAATT
GTAGAATGAATCTGAGGAGGCTTCTCAGTAGACAAAGCCACTCTAACACGATTCTTCGCA
TTCCACTTTATCCTTCCCTTTATTATTACAGCTCTCGTCCTAGTCCACCTCTTATTTCTC
CACGAAACTGGATCCAATAACCCTCTAGGACTAAACTCCAACGCAGACAAAATCCCCTTT
CACCCCTATTATACAGTAAAAGATTTTCTCGGAGTAATCTTACTACTTCTATTCTTCACA
ATTTTAGTCCTCTTCTTTCCCGACCTACTTGGAGACCCAGACAATTACACACCCGCCAAC
CCACTTAATACTCCCCCTCATATTAAACCCGAATGATATTTCTTATTTGCCTACGCTATT
CTCCGCTCTATTCCCAATAAACTAGGTGGAGTACTAGCCCTAGTACTTTCAATCCTCATT
CTAATTTTCCTACCACTAATCCATACATCAAAACAACGAAGCCTAATATTCCGACCAATT
TCACAAATACTCTACTGAATTTTAATCGCCAACCTACTTATCCTCACATGAATTGGAGGT
CAACCAGTAGAACACCCATTTATCATTATTGGCCAACTAGCCTCAATTAGCTATTTCTCC
ATCATCCTAATCTTTCTACCAATCGCAGGGATCATCGAAGACAAAATATTAAAATGATAT
>M5982
ATGACAAACATCCGAAAAATTCACCCCCTAATAAAAATAGTAAACCACTCATTCATTGAC
CTCCCTACTCCTCCAAACATCTCATCCTGATGAAACTTTGGCTCACTCCTAGGAATTTGC
TTAGTAATTCAAATTACAACAGGACTCTTCCTAGCCATACACTATACAGCAGATACAACA
ACAGCATTTTCATCAGTATCCCACATCTGCCGAGACGTAAATTACGGATGACTAATTCGT
TATATACACGCAAACGGAGCCTCAATATTCTTCATCTGCCTATTTATCCATATCGGACGA
GGAATTTACTACGGATCCTACATCTTTCAAGAAACATGAAACATCGGTGTAATCCTCCTA
TTTGCCGTAATAGCTACCGCATTCATGGGTTACGTCCTACCATGAGGACAAATATCCTTC
TGAGGCGCCACAGTCATTACAAACCTCCTCTCAGCAATCCCATACATTGGCCCAACAATT
GTAGAATGAATCTGAGGAGGCTTCTCAGTAGACAAAGCCACTCTAACACGATTCTTCGCA
TTCCACTTTATCCTTCCCTTTATTATTACAGCTCTCGTCCTAGTCCACCTCTTATTTCTC
CACGAAACTGGATCCAATAACCCTCTAGGACTAAACTCCAACGCAGACAAAATCCCCTTT
CACCCCTATTATACAGTAAAAGATTTTCTCGGAGTAATCTTACTACTTCTATTCTTCACA
ATTTTAGTCCTCTTCTTCCCCGACCTACTTGGAGACCCAGACAATTACACACCCGCCAAC
CCACTTAATACTCCCCCTCATATTAAACCCGAATGATATTTCTTATTTGCCTACGCTATT
CTCCGCTCTATTCCCAATAAACTAGGTGGAGTACTAGCCCTAGTACTTTCAATCCTCATT
CTAATTTTCCTACCACTAATCCATACATCAAAACAACGAAGCCTAATATTCCGACCAATT
TCACAAATACTCTACTGAATTTTAATCGCCAACCTACTTATCCTCACATGAATTGGAGGT
CAACCAGTAGAACACCCATTTATCATTATTGGCCAACTAGCCTCAATTAGCTATTTCTCC
ATCATCCTAATCTTTCTACCAATCGCAGGGATCATCGAAGACAAAATATTAAAATGATAT
>LG65
ATGACAAACATCCGAAAAATTCACCCTCTACTAAAAATAGTAAACCACTCATTCATTGAC
CTCCCTACTCCCCCAAACATCTCATCCTGATGAAACTTTGGTTCACTCCTAGGAATTTGC
TTAATGATTCAAATCGCTACAGGACTCTTCCTAGCCATACACTATACAGCAGACACAACA
ACAGCATTTTCATCAGTATCCCACATCTGTCGAGACGTAAATTACGGATGATTAATCCGT
TATATACACGCAAACGGAGCCTCAATATTCTTCATCTGTCTATTTATCCACATCGGACGA
GGGATTTACTACGGATCCTATGTCTTCCAAGAAACATGAAACATTGGTGTAATTCTTCTA
TTTACCGTAATAGCTACCGCATTCATAGGATATGTCCTACCATGAGGACAAATATCCTTC
TGAGGAGCCACAGTCATTACGAACCTCCTCTCAGCAATCCCATACATTGGTCCAACAATT

GTAGAATGAATCTGAGGAGGCTTTTCAGTAGACAAAGCCACTCTAACACGATTCTTTGCA
TTTCACTTCATTCTCCCCTTTATCATTACAGCCCTAGTCCTAGTCCACCTCTTATTTCTT
CACGAAACTGGATCTAACAACCCCCTAGGACTAAATTCCAACGCAGATAAAATTCCCTTT
CATCCCTACTACACAGTAAAAGATTTCCTCGGAGTAATTTTACTACTTCTATTCTTTATA
ATTTTAGTTCTCTTCTTTCCTGACTTACTTGGAGATCCAGACAATTACACACCTGCCAAC
CCACTTAATACTCCCCCCCACATTAAACCCGAATGATATTTCCTATTTGCCTATGCCATT
CTCCGTTCCATTCCCAATAAATTAGGTGGAGTACTAGCCTTAGTACTCTCAATTCTCATT
CTAATTTTTCTACCACTAACTCATACATCCAAACAGCGAAGCCTAATATTCCGACCAATC
TCACAAATACTCTACTGAATTTTAATTGCCAACCTACTTATCCTCACATGAATTGGAGGA
CAACCAGTAGAACATCCATTTATTATTATCGGCCAACTAGCTTCAATCAGCTATTTTTCT
ATCATCCTAATCTTTCTACCAATCGCAGGAATCATCGAAGACAAAATATTAAAATGATAC
>LG66
ATGACAAACATCCGAAAAATTCACCCTCTACTAAAAATAGTAAACCACTCATTCATTGAC
CTCCCTACTCCCCCAAACATCTCATCCTGATGAAACTTTGGTTCACTCCTAGGAATTTGC
TTAATGATTCAAATCGCTACAGGACTCTTCCTAGCCATACACTATACAGCAGACACAACA
ACAGCATTTTCATCAGTATCCCACATCTGTCGAGACGTAAATTACGGATGATTAATCCGT
TATATACACGCAAACGGAGCCTCAATATTCTTCATCTGTCTATTTATCCACATCGGACGA
GGGATTTACTACGGATCCTATGTCTTCCAAGAAACATGAAACATTGGTGTAATTCTTCTA
TTTACCGTAATAGCTACCGCATTCATAGGATATGTCCTACCATGAGGACAAATATCCTTC
TGAGGAGCCACAGTCATTACGAACCTCCTCTCAGCAATCCCATACATTGGTCCAACAATT
GTAGAATGAATCTGAGGAGGCTTTTCAGTAGACAAAGCCACTCTAACACGATTCTTTGCA
TTTCACTTCATTCTCCCCTTTATCATTACAGCCCTAGTCCTAGTCCACCTCTTATTTCTT
CACGAAACTGGATCTAACAACCCCCTAGGACTAAATTCCAACGCAGATAAAATTCCCTTT
CATCCCTACTACACAGTAAAAGATTTCCTCGGAGTAATTTTACTACTTCTATTCTTTATA
ATTTTAGTTCTCTTCTTTCCTGACTTACTTGGAGATCCAGACAATTACACACCTGCCAAC
CCACTTAATACTCCCCCCCACATTAAACCCGAATGATATTTCCTATTTGCCTATGCCATT
CTCCGTTCCATTCCCAATAAATTAGGTGGAGTACTAGCCTTAGTACTCTCAATTCTCATT
CTAATTTTTCTACCACTAACTCATACATCCAAACAGCGAAGCCTAATATTCCGACCAATC
TCACAAATACTCTACTGAATTTTAATTGCCAACCTACTTATCCTCACATGAATTGGAGGA
CAACCAGTAGAACATCCATTTATTATTATCGGCCAACTAGCTTCAATCAGCTATTTTTCT
ATCATCCTAATCTTTCTACCAATCGCAGGAATCATCGAAGACAAAATATTAAAATGATAC
>LG67
ATGACAAACATCCGAAAAATTCACCCTCTACTAAAAATAGTAAACCACTCATTCATTGAC
CTCCCTACTCCCCCAAACATCTCATCCTGATGAAACTTTGGTTCACTCCTAGGAATTTGC
TTAATGATTCAAATCGCTACAGGACTCTTCCTAGCCATACACTATACAGCAGACACAACA
ACAGCATTTTCATCAGTATCCCACATCTGTCGAGACGTAAATTACGGATGATTAATCCGT
TATATACACGCAAACGGAGCCTCAATATTCTTCATCTGTCTATTTATCCACATCGGACGA
GGGATTTACTACGGATCCTATGTCTTCCAAGAAACATGAAACATTGGTGTAATTCTTCTA
TTTACCGTAATAGCTACCGCATTCATAGGATATGTCCTACCATGAGGACAAATATCCTTC
TGAGGAGCCACAGTCATTACGAACCTCCTCTCAGCAATCCCATACATTGGTCCAACAATT
GTAGAATGAATCTGAGGAGGCTTTTCAGTAGACAAAGCCACTCTAACACGATTCTTTGCA
TTTCACTTCATTCTCCCCTTTATCATTACAGCCCTAGTCCTAGTCCACCTCTTATTTCTT
CACGAAACTGGATCTAACAACCCCCTAGGACTAAATTCCAACGCAGATAAAATTCCCTTT
CATCCCTACTACACAGTAAAAGATTTCCTCGGAGTAATTTTACTACTTCTATTCTTTATA
ATTTTAGTTCTCTTCTTTCCTGACTTACTTGGAGATCCAGACAATTACACACCTGCCAAC
CCACTTAATACTCCCCCCCACATTAAACCCGAATGATATTTCCTATTTGCCTATGCCATT
CTCCGTTCCATTCCCAATAAATTAGGTGGAGTACTAGCCTTAGTACTCTCAATTCTCATT
CTAATTTTTCTACCACTAACTCATACATCCAAACAGCGAAGCCTAATATTCCGACCAATC
TCACAAATACTCTACTGAATTTTAATTGCCAACCTACTTATCCTCACATGAATTGGAGGA
CAACCAGTAGAACATCCATTTATTATTATCGGCCAACTAGCTTCAATCAGCTATTTTTCT
ATCATCCTAATCTTTCTACCAATCGCAGGAATCATCGAAGACAAAATATTAAAATGATAC

>LG72

ATGACAAACATCCGAAAAATTCACCCTCTACTAAAAATAGTAAACCACTCATTCATTGAC

CTCCCTACTCCCCCAAACATCTCATCCTGATGAAACTTTGGTTCACTCCTAGGAATTTGC

TTAACGATTCAAATCGCTACAGGACTCTTCCTAGCCATACACTATACAGCAGACACAACA

ACAGCATTTTCATCAGTATCCCACATCTGTCGAGACGTAAATTACGGATGATTAATCCGT

TATATACACGCAAACGGAGCCTCAATATTCTTCATCTGTCTATTTATCCACATCGGACGA

GGAATTTACTACGGATCCTATGTCTTCCAAGAAACATGAAACATTGGTGTAATTCTTCTA

TTTACCGTAATAGCTACCGCATTCATAGGATATGTCCTACCATGAGGACAAATATCCTTC

TGAGGAGCCACAGTCATTACGAACCTCCTCTCAGCAATCCCATACATTGGTCCAACAATT

GTAGAATGAATCTGAGGAGGCTTTTCAGTAGACAAAGCCACTCTAACACGATTCTTTGCA

TTTCACTTTATTCTCCCCTTTATCATTACAGCCCTAGTCCTAGTCCACCTCTTATTTCTT

CACGAAACTGGATCTAACAACCCCCTAGGACTAAATTCCAACGCAGATAAAATTCCCTTT

CATCCCTACTACACAGTAAAAGATTTCCTCGGAGTAATTTTACTACTTCTATTCTTTATA

ATTTTAGTTCTCTTCTTTCCTGACTTACTTGGAGATCCAGACAATTACACACCTGCCAAC

CCACTTAATACTCCCCCCCACATTAAACCCGAATGATATTTCCTATTTGCCTATGCCATT

CTCCGTTCCATTCCCAATAAATTAGGTGGAGTACTAGCCCTAGTACTCTCAATTCTCATT

CTAATTTTTCTACCACTAACTCATACATCCAAACAGCGAAGCCTAATATTCCGACCAATC

TCACAAATACTCTACTGAATTTTAATTGCCAACCTACTCATCCTCACATGAATTGGAGGA

CAACCAGTAGAACATCCATTTATTATTATTGGCCAACTAGCTTCAATCAGTTATTTTTCT

ATCATCCTAATCTTTCTACCAATCGCAGGAATCATCGAAGACAAAATATTAAAATGATAC

>LG73

ATGACAAACATCCGAAAAATTCACCCTCTACTAAAAATAGTAAACCACTCATTCATTGAC

CTCCCTACTCCCCCAAACATCTCATCCTGATGAAACTTTGGTTCACTCCTAGGAATTTGC

TTAATGATTCAAATCGCTACAGGACTCTTCCTAGCCATACACTATACAGCAGACACAACA

ACAGCATTTTCATCAGTATCCCACATCTGTCGAGACGTAAATTACGGATGATTAATCCGT

TATATACACGCAAACGGAGCCTCAATATTCTTCATCTGTCTATTTATCCACATCGGACGA

GGGATTTACTACGGATCCTACGTCTTCCAAGAAACATGAAACATTGGTGTAATTCTTCTA

TTTACCGTAATAGCTACCGCATTCATAGGATATGTCCTACCATGAGGACAAATATCCTTC

TGAGGAGCCACAGTCATTACGAACCTCCTCTCAGCAATCCCATACATTGGTCCAACAATT

GTAGAATGAATCTGAGGAGGCTTTTCAGTAGACAAAGCCACTCTAACACGATTCTTTGCA

TTTCACTTCATTCTCCCCTTTATCATTACAGCCCTAGTCCTAGTCCACCTCTTATTTCTT

CACGAAACTGGATCTAACAACCCCCTAGGACTAAATTCCAACGCAGATAAAATTCCCTTT

CATCCCTACTACACAGTAAAAGATTTCCTCGGAGTAATTTTACTACTTCTATTCTTTATA

ATTTTAGTTCTCTTCTTTCCTGACTTACTTGGAGATCCAGACAATTACACACCTGCCAAC

CCACTTAATACTCCCCCCCACATTAAACCCGAATGATATTTCCTATTTGCCTATGCCATT

CTCCGTTCCATTCCCAATAAATTAGGTGGAGTACTAGCCTTAGTACTCTCAATTCTCATT

CTAATTTTTCTACCACTAACTCATACATCCAAACAGCGAAGCCTAATATTCCGACCAATC

TCACAAATACTCTACTGAATTTTAATTGCCAACCTACTTATCCTCACATGAATTGGAGGA

CAACCAGTAGAACATCCATTTATTATTATTGGCCAACTAGCTTCAATCAGTTATTTTTCT

ATCATCCTAATCTTTCTACCAATTGCAGGAATCATCGAAGACAAAATATTAAAATGATAC

>LG77

ATGACAAACATCCGAAAAATTCACCCCCTACTAAAAATAGTAAATCACTCATTCATCGAC

CTCCCCACTCCTCCAAACATCTCATCCTGATGAAACTTTGGCTCACTTCTAGGAATTTGC

TTAGTTATTCAAATTGCTACAGGACTCTTCCTAGCCATACATTACACAGCAGATACAACA

ACAGCATTTTCATCAGTATCCCACATCTGCCGAGACGTAAATTACGGATGGCTAATCCGT

TATATACACGCAAACGGAGCCTCACTATTCTTCATCTGCCTATTTATCCATATCGGACGA

GGCATTTACTACGGATCCTATATCTTCCAAGAAACATGAAACATCGGTGTAATCCTCCTA

TTTGCCGTAATAGCTACCGCATTCATAGGGTATGTCCTACCATGAGGGCAAATATCTTTC

TGAGGGGCCACAGTGATCACAAATCTCCTCTCAGCAATCCCATATATTGGCCCAACAATT

GTAGAATGAATCTGAGGGGGATTCTCAGTAGACAAAGCCACCCTAACACGATTTTTCGCA

TTCCACTTTATCCTCCCCTTTATTATCACAGCCCTTGTCCTAGTCCACCTCCTATTTCTC

CACGAAACTGGATCCAATAACCCCCTAGGACTAAACTCTAACGCAGACAAAATCCCCTTT
CACCCCTATTACACAGTAAAAGATTTTCTAGGAGTAATTTTACTACTTCTATTCTTCATA
ATCCTAGTTCTCTTCTTTCCTGACTTACTTGGAGACCCAGACAATTACACACCTGCCAAC
CCACTTAACACACCTCCTCATATTAAACCCGAATGATATTTCTTATTTGCCTACGCTATT
CTCCGTTCTATCCCCAATAAACTAGGTGGAGTACTAGCCTTAGTACTATCAATCCTCATT
CTAATTTTCCTACCCCTAACCCATACATCAAAACAACGAAGCCTAATATTTCGACCAATT
TCACAAATACTTTACTGAATTTTAATCGCCAACCTGCTTATCCTTACATGAATCGGAGGC
CAACCAGTAGAACACCCATTTATCATTATTGGCCAACTAGCCTCAATTAGTTATTTTTCT
ATCATCCTGATCTTTCTACCAATCGCAGGAATCATCGAAGACAAAATATTAAAATGATAC
>LG79
ATGACAAACATCCGAAAAATTCACCCCCTACTAAAAATAGTAAATCACTCATTCATCGAC
CTCCCCACTCCTCCAAACATCTCATCCTGATGAAACTTTGGCTCACTCCTAGGAATTTGC
TTAGTTATTCAAATTGCTACAGGACTCTTCCTAGCCATACATTACACAGCAGATACAACA
ACAGCATTTTCATCAGTATCCCACATCTGCCGAGACGTAAATTACGGATGACTAATCCGT
TATATACACGCAAACGGAGCCTCACTATTCTTCATCTGCCTATTTATCCATATCGGACGA
GGCATTTACTACGGATCCTATATCTTCCAAGAAACATGAAACATCGGTGTAATCCTCCTA
TTTGCCGTAATAGCTACCGCATTCATAGGGTATGTCCTACCATGAGGGCAAATATCTTTC
TGAGGGGCCACAGTGATCACAAATCTCCTCTCAGCAATCCCATATATTGGCCCAACAATT
GTAGAATGAATCTGAGGGGGATTCTCAGTAGACAAAGCTACCCTAACACGATTTTTCGCA
TTCCACTTTATCCTCCCCTTTATTATCACAGCCCTTGTCCTAGTCCACCTCCTATTTCTC
CACGAAACTGGATCCAATAACCCCCTAGGACTAAACTCTAACGCAGACAAAATCCCCTTT
CACCCCTATTACACAGTAAAAGACTTTTTAGGAGTAATTTTACTACTCCTATTCTTCATA
ATCCTAGTTCTCTTCTTTCCTGACTTACTTGGAGACCCAGACAATTACACACCTGCCAAC
CCACTTAACACACCTCCTCATATTAAACCCGAATGATATTTCTTATTTGCCTACGCTATT
CTCCGTTCTATCCCCAATAAACTAGGTGGAGTACTAGCCTTAGTACTATCAATCCTCATT
CTAATTTTCCTACCCCTAATCCATACATCAAAACAACGAAGCCTAATATTTCGACCAATT
TCACAAATACTTTACTGAATTTTAATCGCCAACCTGCTTATCCTTACATGAATCGGAGGC
CAACCAGTAGAACACCCATTTATCATTATTGGCCAACTAGCCTCAATTAGTTATTTTTCT
ATCATCCTAATCTTTCTACCAATCGCAGGAATCATCGAAGACAAAATATTAAAATGATAC
>LG91
ATGACAAACATCCGAAAAATTCACCCCCTACTAAAAATAGTAAATCACTCATTCATCGAC
CTCCCCACTCCTCCAAACATCTCATCCTGATGAAACTTTGGCTCACTTCTAGGAATTTGC
TTAGTTATTCAAATTGCTACAGGACTCTTCCTAGCCATACATTACACAGCAGATACAACA
ACAGCATTTTCATCAGTATCCCACATCTGCCGAGACGTAAATTACGGATGACTAATCCGT
TATATACACGCAAACGGAGCCTCACTATTCTTCATCTGCCTATTTATCCATATCGGACGA
GGCATTTACTACGGATCCTATATCTTCCAAGAAACATGAAACATCGGTGTAATCCTCCTA
TTTGCCGTAATAGCTACCGCATTCATAGGGTATGTCCTACCATGAGGGCAAATATCTTTC
TGAGGGGCCACAGTGATCACAAATCTCCTCTCAGCAATCCCATATATTGGCCCAACAATT
GTAGAATGAATCTGAGGGGGATTCTCAGTAGACAAAGCCACCCTAACACGATTTTTCGCA
TTCCACTTTATCCTCCCCTTTATTATCACAGCCCTTGTCCTAGTCCACCTCCTATTTCTC
CACGAAACTGGATCCAATAACCCCCTAGGACTAAACTCTAACGCAGACAAAATCCCCTTT
CACCCCTATTACACAGTAAAAGATTTTCTAGGAGTAATTTTACTACTTCTATTCTTCATA
ATCCTAGTTCTCTTCTTTCCTGACTTACTTGGAGACCCAGACAATTACACACCTGCCAAC
CCACTTAACACACCTCCTCACATTAAACCCGAATGATATTTCTTATTTGCCTACGCTATT
CTCCGTTCTATCCCCAATAAACTAGGTGGAGTACTAGCCTTAGTACTATCAATCCTCATT
CTAATTTTCCTACCCCTAACCCATACATCAAAACAACGAAGCCTAATATTTCGACCAATT
TCACAAATACTTTACTGAATTTTAATCGCCAACCTGCTTATCCTTACATGAATCGGAGGC
CAACCAGTAGAACACCCATTTATCATTATTGGCCAACTAGCCTCAATTAGTTATTTTTCT
ATCATCCTAATCTTTCTACCAATCGCAGGAATCATCGAAGACAAAATATTAAAATGATAC
>LG93
ATGACAAACATCCGAAAAATTCACCCCCTACTAAAAATAGTAAATCACTCATTCATCGAC

CTCCCCACTCCTCCAAACATCTCATCCTGATGAAACTTCGGCTCACTTCTAGGAATTTGC
TTAGTTATTCAAATTGCTACAGGACTCTTCCTAGCCATACATTACACAGCAGATACAACA
ACAGCATTTTCATCAGTATCCCACATCTGCCGAGACGTAAATTACGGATGGCTAATCCGT
TATATACACGCAAACGGAGCCTCACTATTCTTCATCTGCCTATTTATCCATATCGGACGA
GGCATTTACTACGGATCCTATATCTTCCAAGAAACATGAAACATCGGTGTAATCCTCCTA
TTTGCCGTAATAGCTACCGCATTCATAGGGTATGTCCTACCATGAGGGCAAATATCTTTC
TGAGGGGCCACAGTGATCACAAATCTCCTCTCAGCAATCCCATATATTGGCCCAACAATT
GTAGAATGAATCTGAGGGGGATTCTCAGTAGACAAAGCCACCCTAACACGATTTTTCGCA
TTCCACTTTATCCTCCCCTTTATTATCACAGCCCTTGTCCTAGTCCACCTCCTATTTCTC
CACGAAACTGGATCCAATAACCCCCTAGGACTAAACTCCAACGCAGACAAAATCCCCTTT
CACCCCTATTACACAGTAAAAGATTTTCTAGGAGTAATTTTACTACTTCTATTCTTCATA
ATCCTAGTTCTCTTCTTTCCTGACTTACTTGGAGACCCAGACAATTACACACCTGCCAAC
CCACTTAACACACCTCCTCATATTAAACCCGAATGATATTTCTTATTTGCCTACGCTATT
CTCCGTTCTATCCCCAATAAACTAGGTGGAGTACTAGCCTTAGTACTATCAATCCTCATT
CTAATTTTCCTACCCCTAACCCATACATCAAAACAACGAAGCCTAATATTTCGACCAATT
TCACAAATACTTTACTGAATTTTAATCGCCAACCTGCTTATCCTTACATGAATCGGAGGC
CAACCAGTAGAACACCCATTTATCATTATTGGCCAACTAGCCTCAATTAGTTATTTTTCT
ATCATCCTGATCTTTCTACCAATCGCAGGAATCATCGAAGACAAAATATTAAAATGATAC
>LG96
ATGACAAACATCCGAAAAATTCACCCCCTACTAAAAATAGTAAATCACTCATTCATCGAC
CTCCCCACTCCTCCAAACATCTCATCCTGATGAAACTTCGGCTCACTTCTAGGAATTTGC
TTAGTTATTCAAATTGCTACAGGACTCTTCCTAGCCATACATTACACAGCAGATACAACA
ACAGCATTTTCATCAGTATCCCACATCTGCCGAGACGTAAATTACGGATGGCTAATCCGT
TATATACACGCAAACGGAGCCTCACTATTCTTCATCTGCCTATTTATCCATATCGGACGA
GGCATTTACTACGGATCCTATATCTTCCAAGAAACATGAAACATCGGTGTAATCCTCCTA
TTTGCCGTAATAGCTACCGCATTCATAGGGTATGTCCTACCATGAGGGCAAATATCTTTC
TGAGGGGCCACAGTGATCACAAATCTCCTCTCAGCAATCCCATATATTGGCCCAACAATT
GTAGAATGAATCTGAGGGGGATTCTCAGTAGACAAAGCCACCCTAACACGATTTTTCGCA
TTCCACTTTATCCTCCCCTTTATTATCACAGCCCTTGTCCTAGTCCACCTCCTATTTCTC
CACGAAACTGGATCCAATAACCCCCTAGGACTAAACTCCAACGCAGACAAAATCCCCTTT
CACCCCTATTACACAGTAAAAGATTTTCTAGGAGTAATTTTACTACTTCTATTCTTCATA
ATCCTAGTTCTCTTCTTTCCTGACTTACTTGGAGACCCAGACAATTACACACCTGCCAAC
CCACTTAACACACCTCCTCATATTAAACCCGAATGATATTTCTTATTTGCCTACGCTATT
CTCCGTTCTATCCCCAATAAACTAGGTGGAGTACTAGCCTTAGTACTATCAATCCTCATT
CTAATTTTCCTACCCCTAACCCATACATCAAAACAACGAAGCCTAATATTTCGACCAATT
TCACAAATACTTTACTGAATTTTAATCGCCAACCTGCTTATCCTTACATGAATCGGAGGC
CAACCAGTAGAACACCCATTTATCATTATTGGCCAACTAGCCTCAATTAGTTATTTTTCT
ATCATCCTGATCTTTCTACCAATCGCAGGAATCATCGAAGACAAAATATTAAAATGATAC
>LG75 /transl_table=2
ATGACAAACATCCGAAAAATTCACCCTCTACTAAAAATAGTAAACCACTCATTCATTGAC
CTCCCTACTCCCCCAAACATCTCATCCTGATGAAACTTTGGTTCACTCCTAGGAATTTGC
TTAACGATTCAAATCGCTACAGGACTCTTCCTAGCCATACACTATACAGCAGACACAACA
ACAGCATTTTCATCAGTATCCCACATCTGTCGAGACGTAAATTACGGATGATTAATCCGT
TATATACACGCAAACGGAGCCTCAATATTCTTCATCTGTCTATTTATCCACATCGGACGA
GGAATTTACTACGGATCCTATGTCTTCCAAGAAACATGAAACATTGGTGTAATTCTTCTA
TTTACCGTAATAGCTACCGCATTCATAGGATATGTCCTACCATGAGGACAAATATCCTTC
TGAGGAGCCACAGTCATTACGAACCTCCTCTCAGCAATCCCATACATTGGTCCAACAATT
GTAGAATGAATCTGAGGAGGCTTTTCAGTAGACAAAGCCACTCTAACACGATTCTTTGCA
TTTCACTTTATTCTCCCCTTTATCATTACAGCCCTAGTCCTAGTCCACCTCTTATTTCTT
CACGAAACTGGATCTAACAACCCCCTAGGACTAAATTCCAACGCAGATAAAATTCCCTTT
CATCCCTACTACACAGTAAAAGATTTCCTCGGAGTAATTTTACTACTTCTATTCTTTATA

ATTTTAGTTCTCTTCTTTCCTGACTTACTTGGAGATCCAGACAATTACACACCTGCCAAC

CCACTTAATACTCCCCCCCACATTAAACCCGAATGATATTTCCTATTTGCCTATGCCATT

CTCCGTTCCATTCCCAATAAATTAGGTGGAGTACTAGCCCTAGTACTCTCAATTCTCATT

CTAATTTTTCTACCACTAACTCATACATCCAAACAGCGAAGCCTAATATTCCGACCAATC

TCACAAATACTCTACTGAATTTTAATTGCCAACCTACTCATCCTCACATGAATTGGAGGA

CAACCAGTAGAACATCCATTTATTATTATTGGCCAACTAGCTTCAATCAGTTATTTTTCT

ATCATCCTAATCTTTCTACCAATCGCAGGAATCATCGAAGACAAAATATTAAAATGATAC

>N3073

ATGACAAACATCCGAAAAATTCACCCCCTAATAAAAATAGTAAACCACTCATTCATTGAC

CTCCCTACTCCTCCAAACATCTCATCCTGATGAAACTTTGGCTCACTCCTAGGAATTTGC

TTAGTAATTCAAATTACTACAGGACTCTTCCTAGCCATACACTATACAGCAGATACAACA

ACAGCATTTTCATCAGTATCCCACATCTGCCGAGACGTAAATTACGGATGACTAATTCGT

TATATACACGCAAACGGAGCCTCAATATTCTTCATCTGCCTATTTATCCATATCGGACGA

GGAATTTACTACGGATCCTACATCTTTCAAGAAACATGAAACATCGGCGTAATCCTCCTA

TTTGCCGTAATAGCTACCGCATTCATGGGTTATGTCCTACCATGAGGACAAATATCCTTC

TGAGGCGCCACAGTCATTACAAACCTCCTCTCAGCAATCCCATACATTGGCCCAACAATT

GTAGAATGAATCTGAGGAGGCTTCTCAGTAGACAAAGCCACTCTAACACGATTCTTCGCA

TTCCACTTTATCCTTCCCTTTATTATTGCAGCTCTCGTCCTAGTACACCTCTTATTTCTC

CACGAAACTGGATCCAATAACCCTCTAGGACTAAACTCCAACGCAGACAAAATCCCCTTT

CACCCCTATTATACAGTAAAAGATTTTCTCGGAGTAATCTTACTACTTCTATTCTTCACA

ATTTTAGTCCTCTTCTTCCCCGACCTACTTGGAGACCCAGACAATTACACACCCGCCAAC

CCACTTAATACTCCCCCTCATATTAAACCCGAATGATATTTCTTATTTGCCTACGCTATT

CTCCGCTCTATTCCCAATAAACTAGGTGGAGTACTAGCCCTAGTACTTTCAATCCTCATT

CTAATTTTCCTACCACTAATCCATACATCAAAACAACGAAGCCTAATATTCCGACCAATT

TCACAAATACTCTACTGAATTTTAATCGCCAACCTACTTATCCTCACATGAATTGGAGGT

CAACCAGTAGAACACCCATTTATCATTATTGGCCAACTAGCCTCAATTAGCTATTTCTCC

ATCATCCTAATCTTCCTACCAATCGCAGGGATCATCGAAGACAAAATATTAAAATGATAT

>M5961

ATGACAAACATTCGAAAAATTCACCCACTACTAAAAATAGTTAACCACTCATTTATTGAT

CTTCCCACTCCCCCTAATATCTCATCTTGATGAAACTTTGGCTCACTTCTAGGAATTTGC

TTAATAATTCAAATCGCTACAGGACTTTTCCTAGCCATACATTATACAGCAGACACAACA

ACAGCATTCTCATCAGTATCCCATATCTGCCGAGACGTCAATTATGGATGACTAATCCGC

TATATACATGCAAACGGAGCTTCAATATTCTTTATTTGCCTATTCATTCACATTGGACGA

GGGATTTACTACGGATCTTATATCTTTCAAGAAACATGAAACATTGGAGTAATTCTCTTA

TTTGCCGTAATAGCCACCGCATTCATAGGATATGTACTTCCATGAGGACAAATATCCTTC

TGAGGGGCCACAGTCATTACAAATCTTCTTTCAGCTATTCCATATATTGGCCCAACAATC

GTAGAATGAATTTGAGGAGGATTTTCAGTGGACAAAGCCACTTTAACACGATTTTTCGCA

TTCCACTTCATTCTCCCCTTTATTATCACAGCCTTAGTCCTAGTCCATCTCCTATTCCTT

CACGAAACCGGATCTAATAATCCCCTAGGCCTTAACTCCAACTCAGACAAAATCCCTTTT

CACCCATACTACACAGTAAAAGATTTTCTCGGAGTAATTCTACTACTTCTATTTTTCACA

ATTTTAGTCCTCTTCTTCCCTGACTTACTTGGAGATCCAGACAACTACACACCCGCTAAC

CCCCTTAACACTCCCCCCCACATTAAACCCGAATGATATTTCTTATTTGCCTATGCTATC

CTACGTTCAATTCCTAACAAACTAGGAGGAGTCCTAGCCCTAGTACTCTCAATCCTTATC

CTAATTTTTTTACCACTCATCCACACATCAAAACAACGAAGCCTAATATTCCGACCCATT

TCCCAAATACTTTACTGAATCTTAATTGCCAACCTACTTATCCTCACATGAATCGGAGGC

CAACCAGTAGAACACCCATTTATTATCATTGGCCAACTAGCCTCAATCAGTTACTTTTCC

ATTATCCTAATTCTTTTACCAATCGCAGGAATCATTGAAGATAATATATTAAAATGATAT

>M5967

ATGACAAACATTCGAAAAATTCACCCACTACTAAAAATAGTTAACCACTCATTCATTGAT

CTTCCCACTCCCCCTAATATTTCATCTTGATGAAACTTTGGCTCACTCCTAGGAATTTGC

TTAATAATTCAAATCGCTACAGGACTTTTCCTAGCCATACATTATACAGCAGACACAACA

ACAGCATTCTCATCAGTATCCCATATCTGCCGAGACGTCAATTATGGATGACTAATCCGC
TATATACATGCAAACGGAGCTTCAATATTCTTTATTTGCCTATTCATTCACATCGGACGA
GGAATTTACTACGGATCTTATATCTTTCAAGAAACATGAAACATTGGGGTAATTCTCTTA
TTTGCCGTAATAGCCACCGCATTCATAGGGTATGTACTTCCATGAGGACAAATATCCTTC
TGAGGGGCCACAGTCATTACAAATCTTCTTTCAGCTATTCCATATATTGGCCCAACAATC
GTAGAGTGAATTTGAGGAGGATTTTCAGTGGACAAAGCCACTTTAACACGATTTTTCGCA
TTTCACTTCATTCTCCCCTTTATTATCACAGCCTTAGTCCTAGTCCATCTTCTATTCCTT
CACGAAACCGGATCTAATAATCCCCTAGGCCTTAACTCCAACTCAGACAAAATCCCTTTT
CACCCATACTACACAGTAAAAGATTTTCTCGGAGTAATTCTACTACTTCTATTTTTCACA
ATTTTAGTCCTCTTCTTCCCTGACTTACTTGGAGATCCAGACAAYTACACACCCGCTAAC
CCCCTTAACACTCCCCCCCACATTAAACCCGAATGATATTTCTTATTTGCCTATGCTATC
CTACGTTCAATTCCTAACAAACTAGGAGGAGTCCTAGCCCTAGTACTTTCAATCCTTATC
CTAATTTTTTTACCACTCATTCACACATCAAAACAACGAAGCCTAATATTCCGACCTATT
TCCCAAATACTTTACTGAATCTTAATTGCCAACCTACTTATCCTCACATGAATCGGAGGC
CAACCAGTAGAACACCCATTTATTATCATTGGCCAACTAGCCTCAATCAGTTACTTTTCC
ATTATCCTAATCCTTTTACCAATCGCAGGAATCATTGAAGATAATATATTAAAATGATAT
